# Mask and Release Strategy‐Enabled Diversity‐Oriented Synthesis for DNA‐Encoded Library

**DOI:** 10.1002/advs.202307049

**Published:** 2023-12-03

**Authors:** Silin Zhang, Haiman Zhang, Xiawen Liu, Ping Qi, Tingting Tan, Shengdong Wang, Hui Gao, Hongtao Xu, Zhi Zhou, Wei Yi

**Affiliations:** ^1^ Guangzhou Municipal and Guangdong Provincial Key Laboratory of Molecular Target & Clinical Pharmacology The NMPA and State Key Laboratory of Respiratory Disease School of Pharmaceutical Sciences and the Fifth Affiliated Hospital Guangzhou Medical University Guangzhou 511436 China; ^2^ Guangzhou Institute for Food Inspection Guangzhou 511400 China; ^3^ Shanghai Institute for Advanced Immunochemical Studies & School of Life Science and Technology ShanghaiTech University Shanghai 201210 China

**Keywords:** benzofuran, diversity‐oriented synthesis, DNA‐encoded library, mask and release, sulfiliminyl phenol

## Abstract

An ideal DNA‐encoded library (DEL) selection requires the library to consist of diverse core skeletons and cover chemical space as much as possible. However, the lack of efficient on‐DNA synthetic approaches toward core skeletons has greatly restricted the diversity of DEL. To mitigate this issue, this work disclosed a “Mask & Release” strategy to streamline the challenging on‐DNA core skeleton synthesis. *N*‐phenoxyacetamide is used as a masked phenol and versatile directing group to mediate diversified DNA‐compatible C‐H functionalization, introducing the 1st‐dimensional diversity at a defined site, and simultaneously releasing the phenol functionality, which can facilitate the introduction of the 2nd diversity. This work not only provides a set of efficient syntheses toward DNA‐conjugated drug‐like core skeletons such as *ortho*‐alkenyl/sulfiliminyl/cyclopropyl phenol, benzofuran, dihydrobenzofuran but also provides a paradigm for on‐DNA core skeleton synthetic method development.

## Graphical abstract

A “Mask & Release” strategy is disclosed herein to streamline the challenging on‐DNA core skeleton synthesis including *ortho*‐alkenyl/sulfiliminyl/cyclopropyl phenol, benzofuran, dihydrobenzofuran, in which *N*‐phenoxyacetamide is used as a masked phenol and versatile directing group to mediate diversified DNA‐compatible C–H functionalization, introducing the 1st‐dimensional diversity at a defined site, and simultaneously releasing the phenol functionality to realize the 2nd diversity.

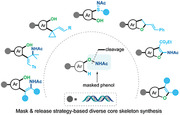

## 1 Introduction

DNA‐encoded chemical library (DEL), conceptually designed by Brenner and Lerner in 1992,^[^ ^1^ ^]^ has become a potent technology for drug discovery.^[^ ^2^, ^3^ ^]^ Combining the advantages of DNA barcoding capacity and combinatorial chemistry, DEL facilitates the rapid generation of an immense number of chemicals via iterative “split‐and‐pool” cycles.^[^ ^4^ ^]^ In the DEL library, each compound is connected to a unique, amplifiable DNA tag, simplifying chemical identification following selection against biomolecular targets.^[^ ^5^ ^]^ So far, a series of hits have been efficiently selected from DELs,^[^ ^6^ ^]^ targeting different types of drug targets such as enzymes,^[^ ^7^, ^8^, ^9^, ^10^, ^11^, ^12^, ^13^ ^]^ kinase,^[^ ^14^ ^]^ chemokines,^[^ ^15^ ^]^ G protein‐coupled receptors (GPCRs),^[^ ^16^, ^17^ ^]^ protein and protein interactions (PPIs),^[^ ^18^, ^19^, ^20^ ^]^ and RNA.^[^ ^21^, ^22^, ^23^, ^24^ ^]^ Furthermore, DEL has emerged became a fundamental platform to bridge chemistry and biology beyond the hit selection activity in innovative drug discovery.^[^ ^25^, ^26^, ^27^, ^28^, ^29^, ^30^, ^31^ ^]^

The success of DEL in identifying binders is due to its exceptional number and diverse chemotypes, introduced via DNA‐compatible chemistry. Rapid advancements in DEL chemistry,^[^ ^32^, ^33^, ^34^ ^]^ mainly including the building block (BB) connection reactions such as diazo‐transfer,^[^ ^35^ ^]^ amide formation,^[^ ^36^ ^]^ diarylether synthesis,^[^ ^37^ ^]^ various cross‐coupling reaction,^[^ ^38^, ^39^, ^40^, ^41^, ^42^, ^43^, ^44^, ^45^ ^]^ C‐H activation and functionalization,^[^ ^46^, ^47^, ^48^, ^49^ ^]^ photo‐promoted reaction,^[^ ^50^, ^51^, ^52^ ^]^ sulfur–fluoride exchange (SuFEx) click chemistry,^[^ ^53^ ^]^ bioinspired click selenylation,^[^ ^54^, ^55^, ^56^ ^]^ and the progresses of on‐DNA privileged heterocycles synthesis have further driven its rapid evolution and applications in basic research and drug discovery.^[^ ^47^, ^48^, ^57^, ^58^, ^59^, ^60^, ^61^, ^62^, ^63^, ^64^, ^65^ ^]^ However, at present, it is well recognized that the diversity of DEL is more dependent on the availability of the core skeletons than the commercially available common BBs.^[^ ^27^ ^]^

**Figure 1:**
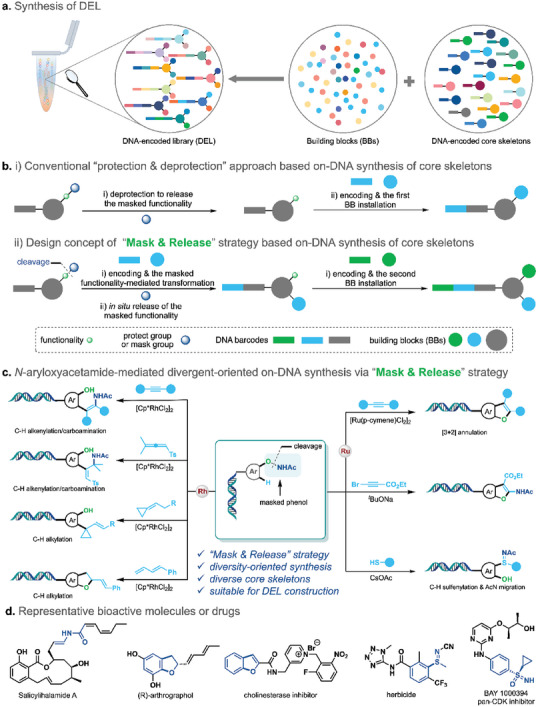
“Mask & Release” strategy‐enabled diversity‐oriented synthesis of DNA‐encoded core skeletons. a) Synthesis of DEL. b) i) Conventional “protection & deprotection” approach based on‐DNA synthesis of core skeletons. ii) Design concept of “Mask & Release” strategy based on DNA synthesis of core skeletons. c) *N*‐phenoxyacetamide‐mediated divergent‐oriented on‐DNA synthesis via “Mask & Release” strategy. d) Representative bioactive molecules or drugs.

Generally, a conventional DEL is synthesized by an interactive encoding and chemical connection of commercially available building blocks (BBs) on a starting DNA‐encoded core skeletons containing two or three functionalities, which was usually synthesized by time‐consuming and laborious off‐DNA synthesis (**Figure** **1**a). In these core skeletons, the functionalities usually need to be protected to make sure the orthogonal assembly of the DNA headpiece and the following 1st and/or 2nd BBs, unavoidable protection, and deprotection procedures have been looped (Figure 1bi). Notably, the deprotection step not only increases the reaction step but also may reduce DNA fidelity. To circumvent such bottleneck, we proposed that a “Mask & Released” strategy would be an ideal solution to the on‐DNA synthesis of multifunctional core skeletons. As illustrated in Figure 1bii, a masked functionality in a DNA‐conjugated core skeleton is designed to serve as a directing group to mediate the on‐DNA diversity‐oriented synthesis (DOS) at a defined site, introducing the 1st diversity, and simultaneously releasing the masked functionality via the cleavage or migration of the mask group, thereby facilitating the introduction of the 2nd diversity via DNA encoding and chemical connection.

After careful evaluation of various functionalities, we envisioned that *N*‐phenoxy amide would be a choice to fulfill the “Mask & Release” on‐DNA core skeleton synthesis. As shown in Figure 1c, *N*‐phenoxyacetamide, a masked phenol in the DNA‐conjugated *N*‐aryloxyacetamide would serve as a versatile directing group to mediate on‐DNA diversification of the aryl skeleton at a defined stie via the Rh(III)‐/Ru(II)‐mediated system^[^ ^66^, ^67^, ^68^ ^]^ or base‐mediated sigmatropic rearrangement process,^[^ ^69^ ^]^ thereby efficiently realized the diversity‐oriented synthesis of DNA‐conjugated core skeletons. Moreover, the in situ released phenol could serve as a valuable anchor to install another dimensional diversity. Herein, this report details an on‐DNA *N*‐aryloxyacetamide mediated diversity‐oriented synthesis of core skeletons via the “Mask & Release” strategy. Diversified on‐DNA reaction modes including on‐DNA C–H alkenylation/carboamination, [3+2] annulation and cyclopropylation, etc. have been well developed, enabling the efficient access to a diverse array of DNA‐conjugated drug‐like core skeletons (Figure 1d).^[^ ^70^, ^71^, ^72^, ^73^, ^74^, ^75^ ^]^ Furthermore, ligation efficiency and DNA integrity are preserved under the established reaction conditions, making these reactions suitable for DEL construction.

## 2 Results and Discussion

Our investigations began by examining the reaction between DNA‐conjugated *N*‐phenoxyacetamide **A1** and diphenylacetylene **B1** using a conventional off‐DNA catalytic system previously described for Rh(III)‐catalyzed C‐H functionalization of *N*‐phenoxyacetamide with alkynes: [Cp*RhCl_2_]_2_/CsOAc.^[^ ^66^, ^76^ ^]^ The desired C–H coupling proceeded smoothly in a PBS (pH 9.4)/THF co‐solvent system, affording the desired DNA‐conjugated *ortho*‐enamine phenols in 34% yield. Further screening of the solvent system, reaction temperature, catalyst loading, additive, and substrate concentration revealed the best reagent blend consisting of 20 equivalents of [CpRhCl_2_]_2_, 1000 equivalents of **B1**, 200 equivalents of CsOAc, and MeOH‐PBS (1:1, pH 9.4), which produced the desired DNA‐conjugated **C1** with an impressive yield of 81%. To the best of our knowledge, this is the first on‐DNA direct C–H functionalization of *N*‐phenoxyacetamide.

**Figure 2:**
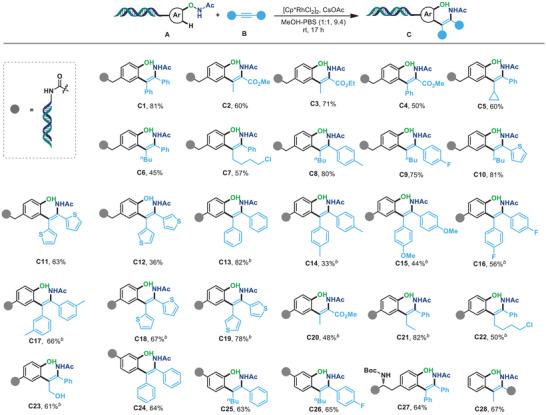
*N*‐phenoxyacetamide‐mediated C–H functionalization with alkynes under Rh promotion for the synthesis of DNA‐conjugated *ortho*‐alkenyl phenols. a) Reaction conditions: (A) (1 equiv, 0.5 mm in *dd*H_2_O), [Cp*RhCl_2_]_2_ (20 equiv, 10 mm in DMA), CsOAc (200 equiv, 200 mm in *dd*H_2_O), (B) (1000 equiv, 500 mm in DMA) in MeOH‐PBS (1:1, pH 9.4, 20 µL) at room temperature for 17 h without exclusion of air or moisture. b) The reaction was conducted at 60 °C for 8 h. The conversion of (**C**) was determined by LC‐MS analysis.

With the optimized reaction conditions established, we further explored the reaction scope of this Rh(III)‐catalyzed on‐DNA C‐H alkenylation/carboamination cascade by designing and successfully synthesizing a variety of DNA‐conjugated *N*‐phenoxyacetamides for DNA‐compatible reaction development. As shown in **Figure** **2**, the electron‐neutral group containing *N*‐phenoxyacetamides proved to be good reactants for this transformation, yielding the desired DNA‐conjugated *ortho*‐alkenyl phenols in moderate to excellent yields (**C1‐C12**). Transformable functional groups such as esters were well tolerated in this reaction (**C2‐C4**). The insertion of aryl‐alkyl disubstituted alkynes resulted in specific regioselectivity, with the aryl‐substituted carbon center installed on the acetamido group (**C5‐C9**). Heterocycle‐substituted alkynes were also proved to be efficient, thus generating the corresponding **C10‐C12** in 36–81% yields. To further probe the generality of this on‐DNA transformation, the electron‐deficient group linked *N*‐phenoxyacetamides were next synthesized and proved as good reactants to couple with diverse symmetrical diaryl acetylenes or unsymmetrical di‐substituted acetylenes, yielding the desired DNA‐conjugated *ortho*‐alkenyl phenols in moderate to excellent yields (**C13‐C23**). Of note, the position of the substituent on the phenyl ring of *N*‐phenoxyacetamides had no significant effect on the reaction outcome, as *meta*‐substituted *N*‐phenoxyacetamides were well compatible and afforded the desired products specifically at the less‐hindered site (**C24‐C26**). Furthermore, the late‐stage C–H functionalization of complex DNA‐conjugated *N*‐phenoxyacetamide containing a tyrosine moiety also demonstrated good reactivity, yielding the corresponding *ortho*‐alkenyaltion phenol **C27** in good yields. Reversely, the DNA‐conjugated alkyne **B21** was also compatible with the reaction conditions to produce the target DNA‐conjugated products **C28** in moderate yield, implying the *N*‐phenoxyacetamide‐mediated C–H functionalization represented a versatile strategy for the construction of DNA‐conjugated drug‐like *ortho*‐enamine phenols.

**Figure 3:**
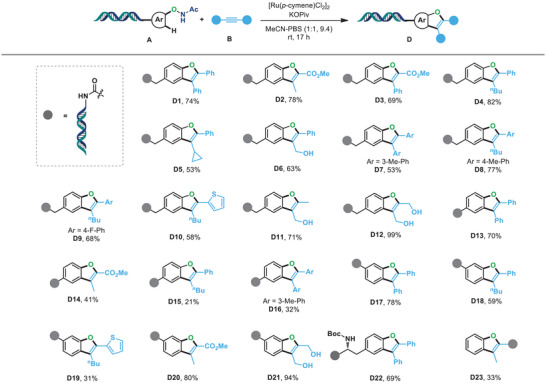
*N*‐phenoxyacetamide mediated on‐DNA synthesis of benzofurans. Reaction conditions: (A) (1 equiv, 0.5 mm in *dd*H_2_O), [Ru(*p*‐cymene)Cl_2_]_2_ (20 equiv, 10 mm in DMA), KOPiv (200 equiv, 200 mm in *dd*H_2_O), (B) (1000 equiv, 500 mm in DMA) in MeCN/PBS (1:1, pH 9.4, 20 µL) at room temperature for 17 h without exclusion of air or moisture. The conversion of product (D) was determined by LC‐MS.

Afterward, to further explore the innovative reactivity of the versatile DNA‐conjugated *N*‐phenoxyacetamide and alkyne substrates, we sought to develop chemodivergent synthetic approaches for the diversified assembly of DNA‐encoded chemicals using a tunable strategy. Through an intensive screening of the corresponding parameters for the reaction optimization, and intriguingly, we found that DNA‐conjugated benzofuran **D1** could be obtained in 74% yield via Ru(II)‐catalyzed redox‐neutral C‐H functionalization under [Ru(*p*‐cymene)Cl_2_]_2_/KOPiv catalytic system by using the MeCN/PBS (1:1, pH 9.4) as the co‐solvent. With the established conditions in hand, we next investigated the compatibility of this system (**Figure** **3**). Initially, we focused on the C‐H functionalization of the electron‐neutral group containing *N*‐phenoxyacetamide with a variety of alkynes. To our delight, the reaction was compatible with either diaryl disubstituted or aryl‐alkyl disubstituted alkynes, producing the corresponding DNA‐conjugated benzofurans in moderate to good yields (**D4**‐**D9**). Furthermore, the catalytic system could be applied to the thiophene‐functionalized substrate, yielding the desired product **D10** in 58% yield. In addition, alkynes bearing different functional groups such as ester and hydroxyl group proceeded smoothly under the optimized reaction conditions, generating the target products in good to excellent yields (**D2**, **D3**, **D11,** and **D12**, 69–99%). Encouraged by the prominent performance of the catalytic system, we further examined the versatility of this transformation by treating the electron‐deficient group containing *N*‐phenoxyacetamide with various alkynes. As a result, diaryl alkynes, aryl‐alkyl substituted alkynes, as well as electron‐deficient alkynes all participated well in this reaction, yielding the corresponding benzofurans smoothly (**D13‐D16**). As expected, *meta*‐substituted *N*‐phenoxyacetamide could also be efficiently converted into the target DNA‐conjugated benzofurans (**D17‐D21**) with excellent regioselectivity. In addition, the late‐stage *N*‐phenoxyacetamide mediated C‐H modification of complex DNA‐conjugated tyrosine derived also demonstrated good reactivity, yielding the corresponding benzofuran **D22** in good yields. Moreover, the DNA‐conjugated alkyne **B21** was also compatible with the reaction conditions to produce the target DNA‐conjugated products **D23** in moderate yield, implying the C–H functionalization of *N*‐phenoxyacetamides represented a versatile strategy for the construction of desired DNA‐conjugated benzofurans.

**Figure 4:**
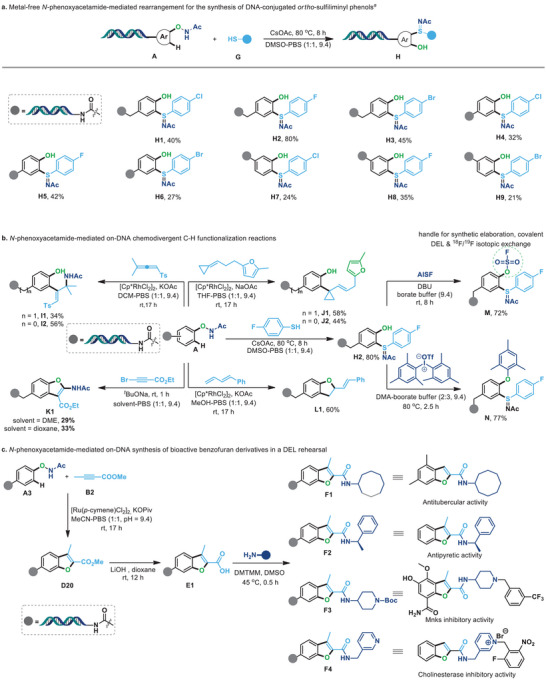
*N*‐phenoxyacetamide‐mediated on‐DNA synthetic application and diversified transformations. a) Metal‐free *N*‐phenoxyacetamide‐mediated rearrangement for the synthesis of DNA‐conjugated *ortho*‐sulfiliminyl phenols. b) *N*‐phenoxyacetamide‐mediated on‐DNA chemodivergent C‐H functionalization reactions & late‐stage functionalization of the released phenol. c) *N*‐phenoxyacetamide‐mediated on‐DNA synthesis of bioactive benzofuran derivatives in a DEL rehearsal.

Considering the well‐defined diversified transformations of *N*‐phenoxyacetamides, further development of novel on‐DNA reaction manifolds should be of significant importance for DNA‐conjugated *N*‐phenoxyacetamide substrates. Consequently, a metal‐free approach has been initially developed for the synthesis of DNA‐conjugated *ortho*‐sulfiliminyl phenols through a C–H sulfenylation/intramolecular rearrangement cascade reaction.^[^ ^69^ ^]^ As demonstrated in **Figure** **4**a, a series of DNA‐conjugated *N*‐phenoxyacetamides and thiophenols were examined to afford the desired products in moderate conversions (**H1**‐**H9**) via cascade C─S and S═N bond formation, and their chemical structures and regioselectivities were further confirmed by the subsequent parallel injection experiment and analysis, in which the product **H5** was employed as an effector (see Figure S3, Supporting Information for details). Of note, other versatile CPs were also compatible to react with DNA‐conjugated *N*‐phenoxyacetamides for the construction of DNA‐conjugated intriguing frameworks, which further demonstrated the generality of such an on‐DNA strategy (Figure 4b). The Rh(III)‐catalyzed C‐H activation/carboamination with allene‐generated phenol‐substituted allylic amines smoothly (**I1** and **I2**), and the C‐H cyclopropylation was realized via Rh(III)‐catalyzed coupling with methylenecyclopropane substrates (**J1** and **J2**). In addition, 2‐amino benzofuran **K1** could be obtained through a metal‐free [3,3]‐sigmatropic rearrangement/[3+2] annulation of DNA‐conjugated *N*‐phenoxyacetamide with ethyl 3‐bromopropiolate. Dihydrobenzofuran **L1** was successfully generated via the Rh(III)‐catalyzed C‐H activation/[3+2] annulation of *N*‐phenoxyacetamide with 1,3‐diene. Notably, the released phenol of **H2** could react with AISF or diaryliodonium salt under our previously reported on‐DNA reaction conditions,^[^ ^37^, ^41^ ^]^ efficiently introducing the privileged diaryl ether pharmacophore (**M**) or fluorosulfate functionality (**N**), which can serve a versatile functionality for various on‐DNA metal‐promoted cross‐coupling reactions,^[^ ^41^, ^42^ ^]^ potential covalent DEL synthesis,^[^ ^77^ ^]^ or radio drug discovery.^[^ ^78^ ^]^

Furthermore, given the importance of benzofuran framework in numerous biologically active molecules,^[^ ^79^, ^80^ ^]^ and to further evaluate the utility potential of the developed on‐DNA *N*‐phenoxyacetamide‐mediated chemodivergent synthesis, we investigated the potential of *N*‐phenoxyacetamide‐mediated on‐DNA synthesis of drug‐like benzofuran derivatives in a DEL rehearsal. As shown in Figure 4c, the *N*‐phenoxyacetamide‐mediated on‐DNA C‐H functionalization of DNA‐conjugated *N*‐phenoxyacetamide with alkyne substrate proceeded smoothly to give DNA‐conjugated benzofuran **D20** under the promotion of [Ru(*p*‐cymene)Cl_2_]_2_/KOPiv. Hydrolysis of **D20** yielded its corresponding acid **E1**, which, upon further reaction with various amines through DMTMM‐promoted amidation, resulted in the formation of the desired DNA‐conjugated and bioactivity‐driven benzofuran‐2‐carboxamides. For example, the condensation of **E1** and cyclooctanamine yielded **F1** bearing a privileged structural motif for targeting the antitubercular activity.^[^ ^81^ ^]^ Additionally, the DNA‐conjugated molecule **F2** for targeting the antipyretic activity^[^ ^82^ ^]^ was generated in 74% yield using this protocol. Finally, this method was also compatible with heterocycle‐substituted amines to produce the corresponding DNA‐tagged compounds **F3** and **F4**, which can be used as the versatile precursor for building the interesting molecule linked with Mnks and cholinesterase inhibitory activities.^[^ ^72^, ^83^ ^]^ These results revealed the compatibility of the developed protocol and provided profound potential for DEL construction.

**Figure 5:**
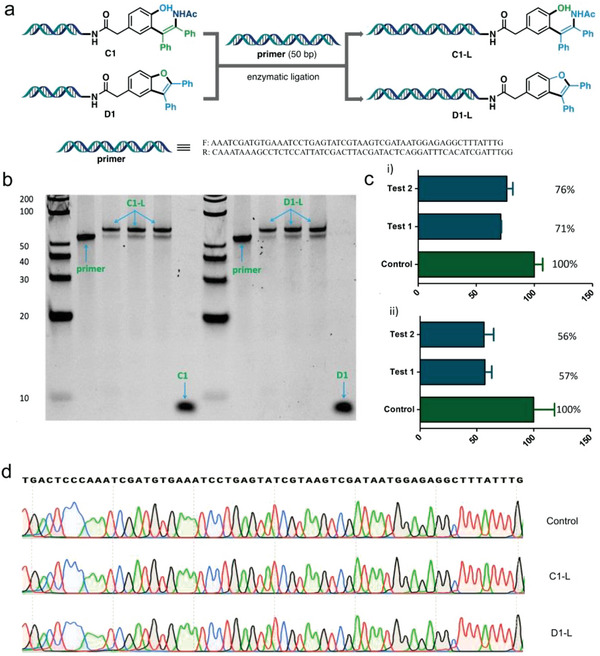
Validation of the integrity of the DNA barcode from the samples of enzymatic ligation. a) DNA ligation. b) DNA ligation analysis of the final products **C1‐L** and **D1‐L**. c) qPCR analysis of residual amplifiable material of an inhouse DEL after exposure to the reaction conditions of i) on‐DNA *ortho*‐enamine phenols synthesis and ii) benzofuran synthesis. d) Sanger sequencing results of **C1‐L** and **D1‐L**.

Preserving the integrity of DNA tags after chemical reactions is crucial during DEL construction, as the DNA barcode is the sole record used to decode the chemical structure.^[^ ^28^, ^30^ ^]^ Consequently, we performed enzymatic ligations of a 50‐mer oligo DNA primer with two conjugates **C1** and **D1**, which contained representative *ortho*‐alkenyl phenols and benzofurans, respectively (**Figure** **5**a). As shown in Figure 5b,d, polyacrylamide gel electrophoresis and Sanger sequencing were respectively employed to analyze these ligation products. In the end, the results confirmed that the resulting sequence aligned with expectations. Subsequently, we assessed DNA damage using qPCR. Encouragingly, the recovery yields of an inhouse DEL reached 71% and 56% under the standard reaction conditions of *N*‐hyoxylacetamide‐mediated on‐DNA *ortho*‐enamine phenols synthesis and benzofuran synthesis (Figure 5c), which are much higher than the threshold of practical DEL synthesis.^[^ ^84^ ^]^ Taken together, these results suggested that on‐DNA integration of functionalized *ortho*‐alkenyl phenols and benzofurans resulted in acceptable DNA damage and was suitable for DEL synthesis.

## 3 Conclusion

In conclusion, to circumvent the bottleneck concerning on‐DNA core skeleton synthesis, a “Mask and Released” strategy was proposed to streamline the diversity‐oriented synthesis of DNA‐conjugated core skeletons. Using *N*‐phenoxyacetamide as a masked phenol and versatile directing group, diversified DNA‐compatible C‐H functionalization has been realized for the synthesis of DNA‐conjugated *ortho*‐alkenyl/sulfiliminyl/cyclopropyl phenols, benzofurans, dihydrobenzofuran, *etc*. with good functional group compatibility, introducing the 1st dimensional diversity at a defined site. Furthermore, the simultaneously released phenol can facilitate the introduction of the 2nd diversity. These reactions can be used for DEL construction as they maintain the ligation efficiency and integrity of DNA barcodes. Further applications of the developed strategy in building the phenol and benzofuran‐focused DELs for the selection of hit compounds via the “split‐and‐pool” strategy are currently underway. Taken together, this work not only provides a set of efficient syntheses toward DNA‐conjugated drug‐like core skeletons but also provides a paradigm for on‐DNA core skeletons synthetic method development.

## Conflict of Interest

The authors declare no conflict of interest.

## Supporting information

Supporting Information

## Data Availability

The data that support the findings of this study are available in the supplementary material of this article.
