# Supplementary material for: Mask and Release Strategy‐Enabled Diversity‐Oriented Synthesis for DNA‐Encoded Library
Source: Adv Sci (Weinh). 2023 Dec 3;11(6):2307049. doi: 10.1002/advs.202307049 (PMC10853742; doi:10.1002/advs.202307049)
Supplement: Supplementary file 1 — Supporting Information [file ADVS-11-2307049-s001.pdf]

ADVANCED SCIENCE
Open Access

Supporting Information

for *Adv. Sci.*, DOI 10.1002/advs.202307049

Mask and Release Strategy-Enabled Diversity-Oriented Synthesis for DNA-Encoded Library

Silin Zhang, Haiman Zhang, Xiawen Liu, Ping Qi, Tingting Tan, Shengdong Wang, Hui Gao, Hongtao Xu, Zhi Zhou* and Wei Yi**

Support Information

Mask and Release Strategy-Enabled Diversity-Oriented Synthesis for DNA-Encoded Library

Silin Zhang,[a,‡] Haiman Zhang,[a,‡] Xiawen Liu,[a,‡] Ping Qi,[b] Tingting Tan,[c] Shengdong Wang,[a] Hui Gao,[a] Hongtao Xu,[c,*] Zhi Zhou[a,*] and Wei Yi[a,*]

[a]Guangzhou Municipal and Guangdong Provincial Key Laboratory of Molecular Target & Clinical Pharmacology, the NMPA and State Key Laboratory of Respiratory Disease, School of Pharmaceutical Sciences and the Fifth Affiliated Hospital, Guangzhou Medical University, Guangzhou, Guangdong 511436, People's Republic of China

[b]Guangzhou Institute for Food Inspection, Guangzhou, 511400, People's Republic of China

[c]Shanghai Institute for Advanced Immunochemical Studies & School of Life Science and Technology, ShanghaiTech University, Shanghai 201210, People's Republic of China

[‡]These authors contributed equally to this work

E-mail: yiwei@gzhmu.edu.cn; zhouzhi@gzhmu.edu.cn; xuht@shanghaitech.edu.cn

Table of Contents

1. General methods 3
2. The structure of DNA headpiece 3
3. General procedure for the synthesis of substrates A 4
4. General procedure for the synthesis of DNA-conjugated *ortho*-alkenyl phenols. 5
5. General procedure for the synthesis of DNA-conjugated benzofurans 7
6. General procedure for the synthesis of DNA-conjugated *ortho*-sulfiliminyl phenols 8
7. General procedure for the synthesis of products F 9
8. General procedure for the synthetic application and diversified transformations of DNA-conjugated *N*-phenoxyacetamides. 10
9. Synthesis of off-DNA products 14
10. Parallel Injection Experiment and Analysis 20
11. Evaluation of the DNA Tags Degradation 25
Copies of NMR spectra 29
LC trace of products 39
Reference 119

1. General Methods

All reagents and DNA headpiece **HP-NH2** (5'/5phos/GAGTCA/iSp9/iUniAmM/iSp9 /TGACTCCC-3', **Figure S1**) were obtained from commercial sources unless otherwise noted and used as received. All on-DNA reactions were performed in 1.5 mL Eppendorf tubes. On-DNA reactions in the studies of reaction condition optimization and substrate scope extension were analyzed by UPLC-MS. Typically, samples were dissolved in an appropriate amount of distilled and deionized water (ddH_2O) and injected into a reverse-phase chromatography column (Xbridge Oligonucleotide BEH C18 column, 1.7 μm, 2.1×50 mm). The elution was carried out as followings: 5-95% solvent B over 10 min, 0.4 mL/min, λ = 260 nm; solvent A: 0.75% v/v hexafluoroisopropanol/ 0.038% v/v triethylamine in methanol/water = 5/95; solvent B: 0.75% v/v hexafluoroisopropanol/ 0.038% v/v triethylamine in methanol/water = 90/10. The effluents were analyzed by Xevo G2-XS Q-TOF with electrospray ionization source. Water was purified with a Millipore Milli-Q system. NMR spectra were recorded on Bruker AM-500 instruments using solvent peaks as the internal standard (^{1}H NMR, Me_4Si at 0 ppm, Chloroform-*d* at 7.26 ppm, DMSO-d_6 at 2.50 ppm; ^{13}C NMR, Me_4Si at 0 ppm, Chloroform-*d* at 77.16 ppm, DMSO-d_6 at 39.52 ppm). ^{1}H NMR multiplicity data are denoted by s (singlet), d (doublet), t (triplet), and m (multiplet). High-resolution mass spectra (HRMS-ESI) were obtained on an AB Sciex 4600 Q-TOF MS instrument.

2. The Structure of DNA Headpiece

DNA headpiece **HP-NH2** (5′/5Phos/GAGTCA/iSp9/iUniAmM/iSp9/TGACTCCC-3′, MW = 4937.23, **Figure S1**).

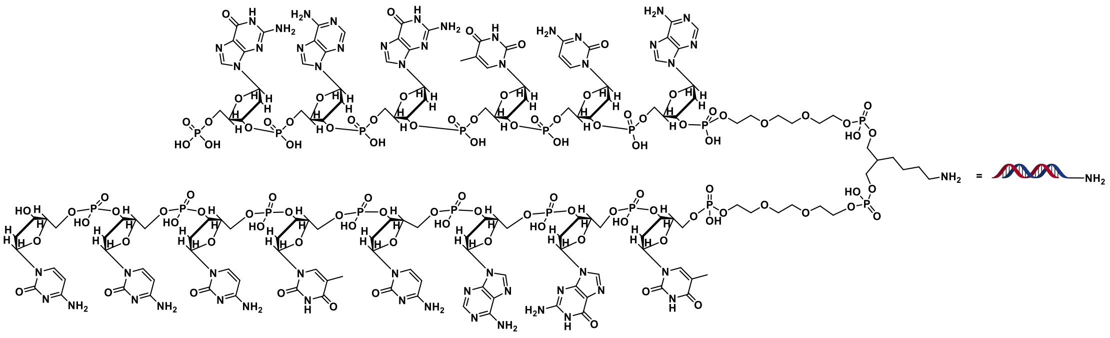

Figure S1. The structure of DNA headpiece

3. General Procedure for the Synthesis of Substrates A

Scheme S1. Synthesis of substrates A

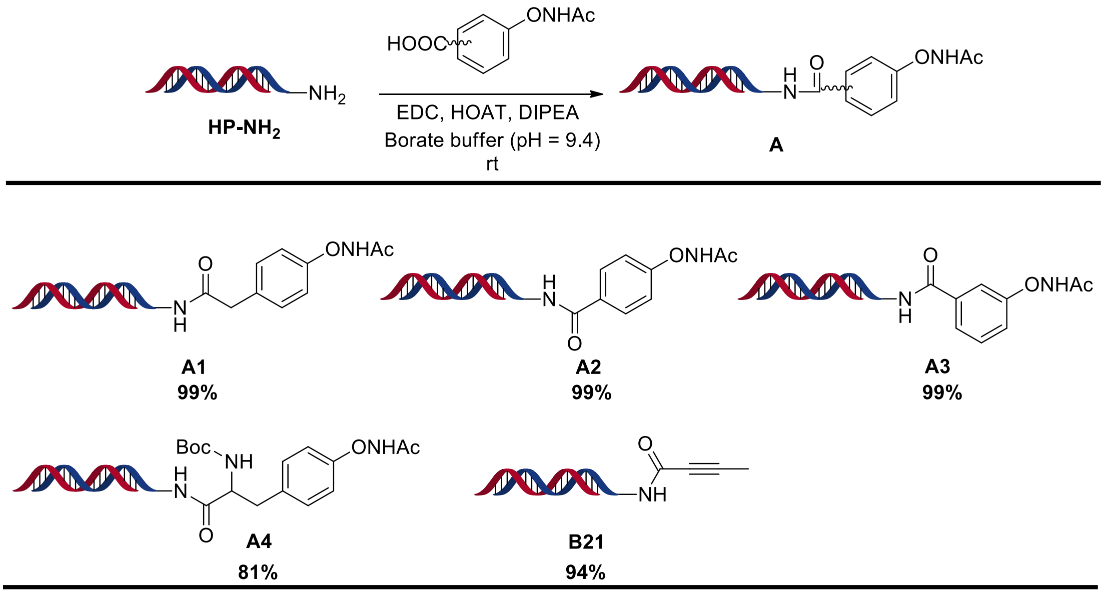

General procedure for the synthesis of A: The carboxyl group substituted *N*-phenoxyacetamides were prepared according to a known procedure.[S1] Mix acid (126 μL, 120 mM in DMSO), HOAT (2400 μL, 120 mM in DMSO), EDC (2400 μL, 600 mM in DMSO) and DIPEA (2400 μL, 600 mM in DMSO) together and incubate 1 h at room temperature, after that the solution of DNA headpiece (300 nmol, dissolved in 600 μL of borate buffer (pH = 9.4) was added to the mixture. The resulted mixture was vortexed and stood at 25 ^{o}C overlight. Add aqueous 5 M NaCl solution (10% by volume) and cold ethanol (2.5 times by volume, ethanol stored at -20 °C) to the resultant supernatant, and stored at -80 ^{o}C for at least 1 h. The mixture

was centrifuged at 4 °C for 30 min at 12000 rpm to remove the supernatant. The resulting pellet was re-dissolved in ddH_2O (600 μL), which was used in following reaction without further purification.

4. General Procedure for the Synthesis of DNA-Conjugated *ortho*-Alkenyl Phenols

To the **A1** or **A2** (4 μL, 0.5 mM in ddH_2O) was added 1000 equiv of **B1** (4 μL, 500 mM in DMA), 20 equiv of [Cp*RhCl_2]$_2$ (4 μL, 10 mM in DMA) and 200 equiv of CsOAc (2 μL, 200 mM in ddH_2O) in solvent-PBS (20 μL, 1:1). The mixture was vortexed and stood at rt for 17 h. After the reaction was completed, added scavenger sodium diethyldithiocarbamic acid (3.2 μL, 500 mM in ddH_2O) to the mixture, and heated the reaction mixture at 60 °C for at least 30 min. Then the mixture was centrifuged at 4 °C for 30 min at 4800 rpm, and the resultant supernatant was collected. Add aqueous 5 N NaCl solution (10% by volume) and cold ethanol (2.5 times by volume, ethanol stored at -20 °C) to the resultant supernatant. The mixture was vortexed and stored at a -80 °C freezer for more than 1 h. The sample was centrifuged for around 30 min at 4 °C in a microcentrifuge at 12000 rpm. The above supernatant was removed and the pellet (precipitate) was dissolved in deionized water for LC-MS detection.

Table S1. Screening of the reaction conditions using A2.[a]

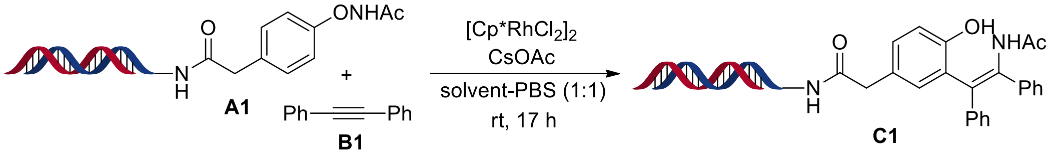

Entry	Solvent	pH of PBS	Additive	Yield of **C1**
1	DCM	4.2	/	NR
2	DCM	5.9	/	NR
3	DCM	7.0	/	NR
4	^{t}BuOH	9.4	HOAc	NR
5	^{t}BuOH	7.0	HOAc	NR
6	^{t}BuOH	4.2	HOAc	NR

7[b]	^{t}BuOH	4.2	HOAc	NR
8	DCM	4.2	TBAC	trace
9	DCM	4.2	TBAC+HOAc	trace
10	MeOH	4.2	HOAc	trace
11	^{t}BuOH	4.2	/	trace
12	MeOH	7.0	HOAc	21%
13	MeOH	9.4	HOAc	18%
14	DCM	7.0	TBAC	57%
15	DCM	9.4	TBAC	61%
16	EtOH	4.2	/	54%
17	^{i}PrOH	4.2	/	28%
18	TFE	4.2	/	4%
19	THF	4.2	/	34%
20	DMA	4.2	/	41%
21	DCM	4.2	TBAC+HOAc	trace
22	DCM	4.2	TBAC	<5%
23	MeOH	4.2	/	56%
24	MeOH	7.0	/	78%
25	MeOH	9.4	/	81%
26[c]	MeOH	9.4	/	70%
27[d]	MeOH	9.4	/	60%
28[e]	MeOH	9.4	/	57%
29[f]	MeOH	9.4	/	75%

[a]Reaction conditions: **A1** (1 equiv, 0.5 mM in ddH_2O), $[Cp^*RhCl_2]_2$ (20 equiv, 10 mM in DMA), CsOAc (200 equiv, 200 mM in ddH_2O), **B1** (1000 equiv, 500 mM in DMA) in solvent-PBS (1:1, 20 μL) at rt for 17 h without exclusion of air or moisture. [b]^{t}BuOH-PBS (2:1, pH = 4.2, 20 μL). [c]MeOH-PBS (1:1, pH = 9.4, 10 μL). [d]MeOH-PBS (2:1, pH = 9.4, 50 μL). [e]MeOH-PBS (1:1, pH = 9.4, 25 μL). [f]$[Cp^*RhCl_2]_2$, CsOAc and **B1** are dissolved in MeOH. The yield of **C1** was determined by LC-MS.

Table S2. Screening of the reaction conditions using A2.[a]

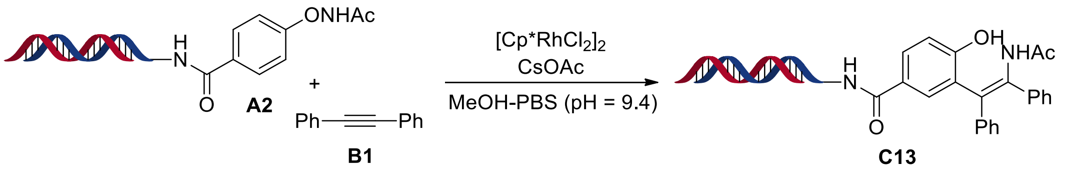

Entry	MeOH:PBS	T（°C）	Time（h）	Yield of **C13**
1	1:1	rt	17	76%
2[b]	1:1	rt	17	69%
3	1:1	40	8	80%
4	1:1	60	8	82%
5	2:1	rt	17	77%

[a]Reaction conditions: **A2** (1 equiv, 0.5 mM in ddH_2O), $[Cp^*RhCl_2]_2$ (20 equiv, 10 mM in DMA),

CsOAc (200 equiv, 200 mM in ddH_2O), **B1** (1000 equiv, 500 mM in DMA) in MeOH-PBS (pH = 9.4, 20 μL) without exclusion of air or moisture. [b]$[Cp*RhCl_2]_2$, CsOAc and **B1** are dissolved in MeOH. The yield of **C13** was determined by LC-MS.

5. General Procedure for the Synthesis of DNA-Conjugated Benzofurans

To the **A1** (4 μL, 0.5 mM in ddH_2O) was added 1000 equiv of **B2** (4 μL, 500 mM in DMA), 20 equiv of [Ru(*p*-cymene)Cl_2]$_2$ (4 μL, 10 mM in DMA) and 200 equiv of base (2 μL, 200 mM in ddH_2O) in solvent-PBS (20 μL, 1:1, pH = 9.4). The mixture was vortexed and stood at rt for 17 h. After the reaction was completed, added scavenger sodium diethyldithiocarbamic acid (3.2 μL, 500 mM in ddH_2O) to the mixture, and heated the reaction mixture at 60 °C for at least 30 min. Then the mixture was centrifuged at 4 °C for 30 min at 4800 rpm, and the resultant supernatant was collected. Add aqueous 5 N NaCl solution (10% by volume) and cold ethanol (2.5 times by volume, ethanol stored at -20°C) to the resultant supernatant. The mixture was vortexed and stored at a -80 °C freezer for more than 1 h. The sample was centrifuged for around 30 min at 4 °C in a microcentrifuge at 12000 rpm. The above supernatant was removed and the pellet (precipitate) was dissolved in deionized water for LC-MS detection.

Table S3. Screening of the reaction conditions.[a]

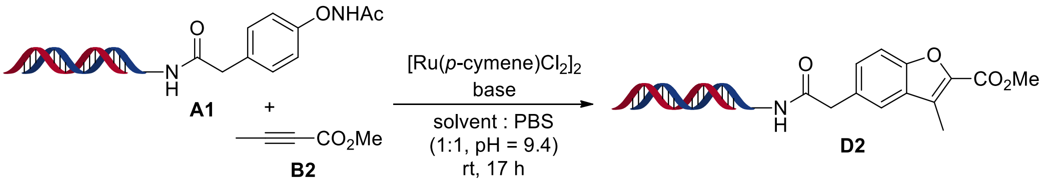

Entry	Solvent	Base	Result
1	DCM+TBAC	K_2CO_3	61%
2	MeCN	K_2CO_3	72%
3	MeCN	CsOAc	71%
4	MeCN	KOAc	73%
5	MeCN	Cs_2CO_3	62%
6	MeCN	DBU	54%
7	Dioxane	K_2CO_3	50%

8	MeCN	K_2CO_3	72%
9	MeCN	K_2CO_3	6%
10	DCM+TBAC	KOAc	71%
11[b]	MeCN	KOAc	73%
12	MeOH	KOAc	71%
13	TFE	KOAc	71%
14	^{t}BuOH	KOAc	ND
15	THF	KOAc	66%
16	MeCN	KOPiv	77%
17	MeCN	$Cu(OAc)_2$	41%
18	MeCN	NaOAc	70%
19[c]	MeCN	KOPiv	70%
20[d]	MeCN	KOPiv	76%
21[e]	MeCN	KOPiv	73%
22[f]	MeCN	KOPiv	82%

[a]Reaction conditions: **A1** (1 equiv, 0.5 mM in ddH_2O), $[Ru(p\text{-cymene})Cl_2]_2$ (20 equiv, 10 mM in DMA), base (200 equiv, 200 mM in ddH_2O), **B2** (1000 equiv, 500 mM in DMA) in solvent-PBS (1:1, pH = 9.4, 20 μL) at rt for 17 h without exclusion of air or moisture. [b]MeCN-PBS (2:1, pH = 9.4, 20 μL). [c]The pH of PBS is 4.2. [d]The pH of PBS is 7.0. [e]The reaction was conducted at 40 ^{o}C for 8 hours. [f]The reaction was conducted at 60 ^{o}C for 8 hours. The yield of **D2** was determined by LC-MS.

6. General Procedure for the Synthesis of DNA-Conjugated *ortho*-Sulfiliminyl Phenols

To the **A1** (4 μL, 0.5 mM in ddH_2O) was added 1000 equiv of **G1** (4 μL, 500 mM in DMA) and 200 equiv of CsOAc (2 μL, 200 mM in ddH_2O) in DMSO-PBS (10 μL, 1:1, pH = 9.4). The mixture was vortexed and stood at 80 ^{o}C for 8 h. After the reaction was completed, added scavenger sodium diethyldithiocarbamic acid (3.2 μL, 500 mM in ddH_2O) to the mixture, and heated the reaction mixture at 60 °C for at least 30 min. Then the mixture was centrifuged at 4 °C for 30 min at 4800 rpm, and the resultant supernatant was collected. Add aqueous 5 N NaCl solution (10% by volume) and cold ethanol (2.5 times by volume, ethanol stored at -20°C) to the resultant supernatant. The mixture was vortexed and stored at a -80 °C freezer for more than 1 h. The sample was centrifuged for around 30 min at 4 °C in a microcentrifuge at 12000 rpm. The above supernatant was removed and the pellet (precipitate) was dissolved in deionized water for LC-MS detection.

Table S4. Screening of the reaction conditions.[a]

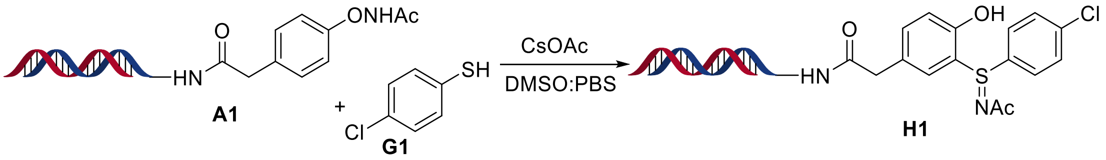

Entry	Solvent:PBS	pH of solvent	T(°C)	Time(h)	Yield
1	1:0	/	rt	17	trace
2	1:1	9.4	rt	17	21%
3	1:1	9.4	40	8	trace
4	1:1	9.4	60	8	13%
5	1:1	9.4	80	8	40%

[a]Reaction conditions: **A1** (1 equiv, 0.5 mM in ddH$_2$O), CsOAc (200 equiv, 200 mM in ddH$_2$O), **G1** (1000 equiv, 500 mM in DMA) in DMSO-PBS (1:1, pH = 9.4, 10 μL) at 80 °C for 8 h without exclusion of air or moisture. Conversion of **A1** was determined by LC-MS.

7. General Procedure for the Synthesis of Products F

To **E1** (4 μL, 2 nmol, 0.5 mM in ddH$_2$O) was added 13.5 μL of MOPS buffer (pH = 7, 50 mM in ddH$_2$O) and 3 μL of DMT-MM (500 mM in MOPS buffer). The resulting mixture was vortexed and stirred at 45 °C for 30 minutes. Then 7.5 μL of desired amine (100 mM in DMSO) was added to the mixture and stirred at 45 °C for 12 hours. After that 5 M NaCl solution (10% by volume) and cold ethanol (2.5 times by volume, ethanol stored at -20°C) were added, vortexed, and incubated at -80 °C for at least 30 minutes. The sample was centrifuged for 30 minutes at 4 °C in a microcentrifuge at 12,000 rpm to remove the supernatant. The resulting pellet (precipitate) was re-dissolved in ddH$_2$O (10 μL) for LC-MS detection.

Scheme S2. Synthesis of DNA-conjugated bioactive benzofuran derivatives

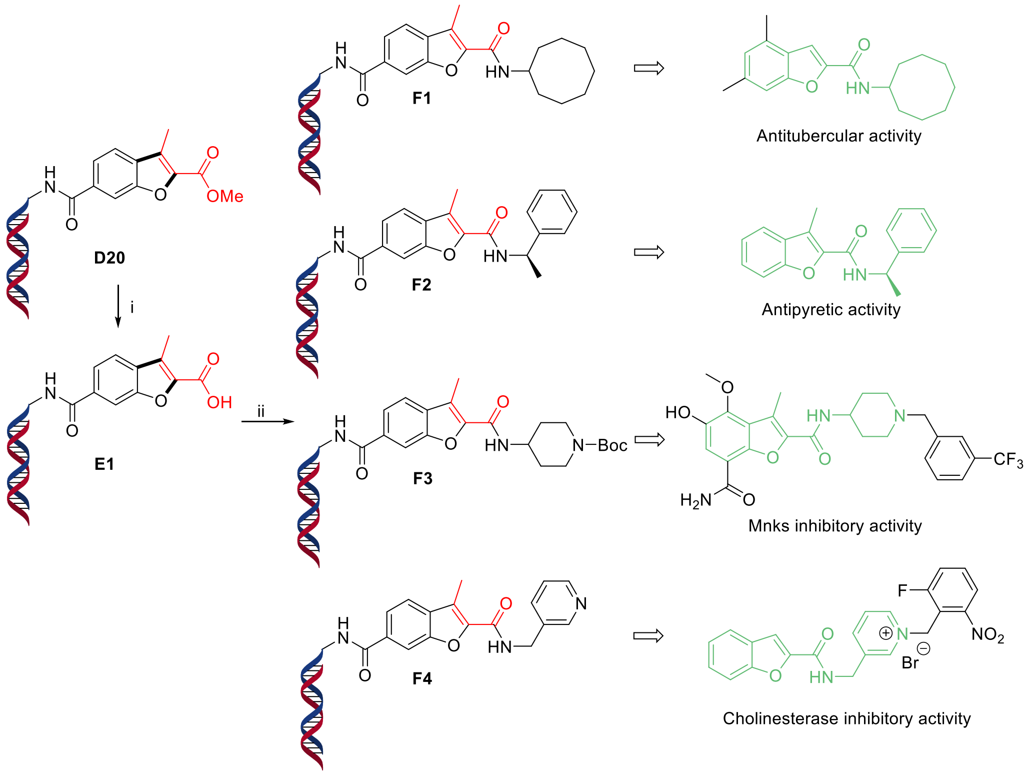

8. General Procedure for the Synthetic Application and Diversified Transformations of DNA-Conjugated *N*-Phenoxyacetamides

All reactions are reacted according to the conditions under the reaction formula. Then the mixture was centrifuged at 4 °C for 30 min at 4800 rpm, and the resultant supernatant was collected. Add aqueous 5 N NaCl solution (10% by volume) and cold ethanol (2.5 times by volume, ethanol stored at -20°C) to the resultant supernatant. The mixture was vortexed and stored at a -80 °C freezer for more than 1 h. The sample was centrifuged for around 30 min at 4 °C in a microcentrifuge at 12000 rpm. The above supernatant was removed and the pellet (precipitate) was dissolved in deionized water for LC-MS detection.

Scheme S3. Synthesis of compound C27 and D22

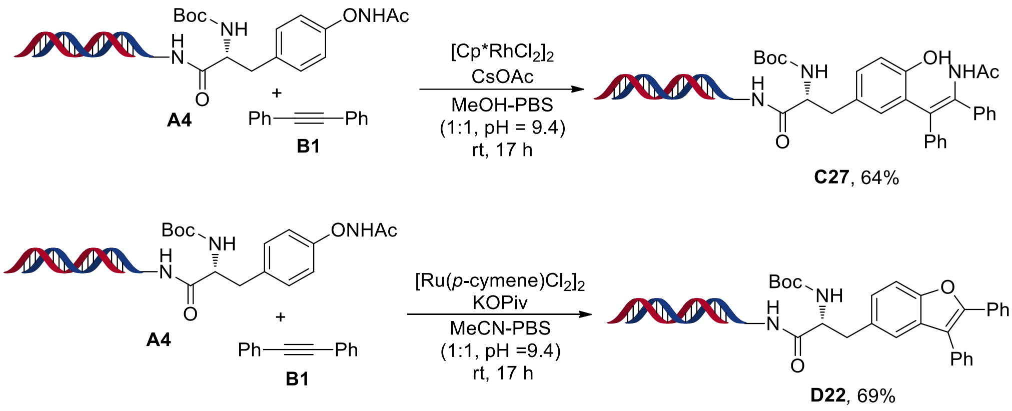

Conditions for the synthesis of compound **C27**: **A4** (1 equiv, 0.5 mM in ddH_2O), [Cp*RhCl$_2$]$_2$ (20 equiv, 10 mM in DMA), CsOAc (200 equiv, 200 mM in ddH_2O), **B1** (1000 equiv, 500 mM in DMA) in MeOH-PBS (1:1, pH = 9.4, 20 μL) at rt for 17 h without exclusion of air or moisture. The yield of **C27** was determined by LC-MS.

Conditions for the synthesis of compound **D22**: **A4** (1 equiv, 0.5 mM in ddH_2O), [Ru(*p*-cymene)Cl$_2$]$_2$ (20 equiv, 10 mM in DMA), KOPiV (200 equiv, 200 mM in ddH_2O), **B1** (1000 equiv, 500 mM in DMA) in MeCN-PBS (1:1, pH = 9.4, 20 μL) at rt for 17 h without exclusion of air or moisture. The yield of **D22** was determined by LC-MS.

Scheme S4. Synthesis of compound C28 and D23

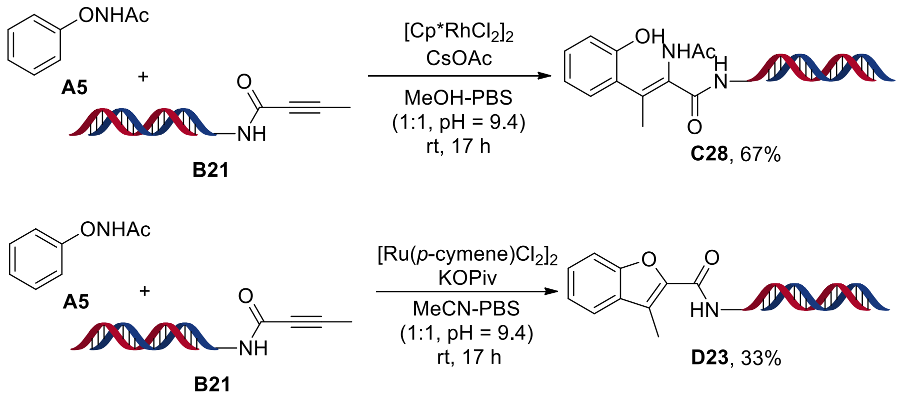

Conditions for the synthesis of compound **C28**: **B21** (1 equiv, 0.5 mM in ddH_2O), [Cp*RhCl$_2$]$_2$ (20 equiv, 10 mM in DMA), CsOAc (200 equiv, 200 mM in ddH_2O), **A5** (1000 equiv, 500 mM in DMA) in MeOH-PBS (1:1, pH = 9.4, 20 μL) at rt for 17 h without exclusion of air or moisture. The yield of **C28** was determined by LC-MS.

Conditions for the synthesis of compound **D23**: **B21** (1 equiv, 0.5 mM in ddH_2O), [Ru(*p*-cymene)Cl_2]$_2$ (20 equiv, 10 mM in DMA), KOPiv (200 equiv, 200 mM in ddH_2O), **A5** (1000 equiv, 500 mM in DMA) in MeCN-PBS (1:1, pH = 9.4, 20 μL) at rt for 17 h without exclusion of air or moisture. The yield of **D23** was determined by LC-MS.

Scheme S5. Synthesis of compound I1 and I2

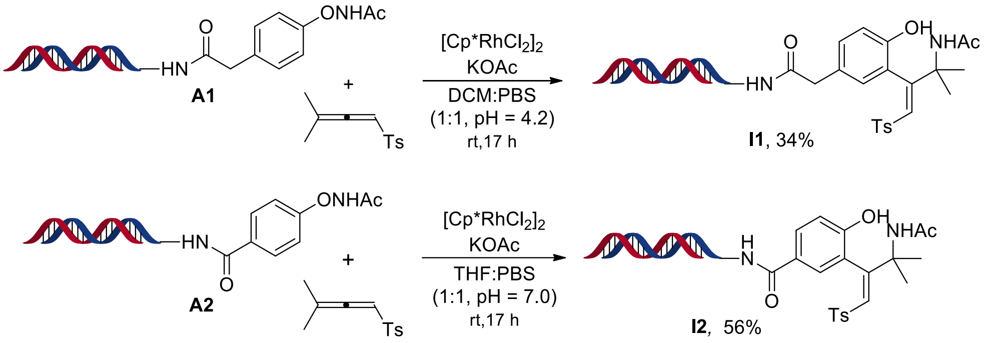

Conditions for the synthesis of compound **I1**: **A1** (1 equiv, 0.5 mM in ddH_2O), [Cp*RhCl_2]$_2$ (20 equiv, 10 mM in DMA), KOAc (200 equiv, 200 mM in ddH_2O) and allene (1000 equiv, 500 mM in DMA) in DCM-PBS (1:1, pH = 4.2, 20 μL) at rt for 17 h without exclusion of air or moisture. The yield of **I1** was determined by LC-MS.

Conditions for the synthesis of compound **I2**: **A2** (1 equiv, 0.5 mM in ddH_2O), [Cp*RhCl_2]$_2$ (20 equiv, 10 mM in DMA), KOAc (200 equiv, 200 mM in ddH_2O) and allene (1000 equiv, 500 mM in DMA) in THF-PBS (1:1, pH = 7.2, 20 μL). The yield of **I2** was determined by LC-MS.

Scheme S6. Synthesis of compound J1 and J2

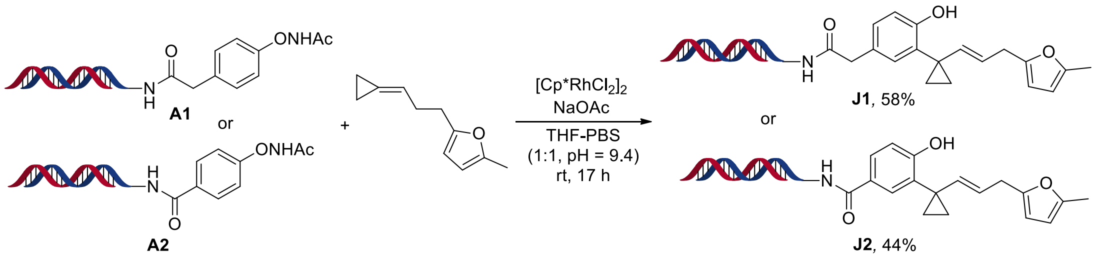

Reaction Conditions: **A1** or **A2** (1 equiv, 0.5 mM in ddH_2O), [Cp*RhCl_2]$_2$ (20 equiv, 10 mM in DMA), NaOAc (200 equiv, 200 mM in ddH_2O), methylenecyclopropane

substrate (1000 equiv, 500 mM in DMA) in THF-PBS (1:1, pH = 9.4, 20 μL) at rt for 17 h without exclusion of air or moisture. The yield of **J1** or **J2** was determined by LC-MS.

Scheme S7. Synthesis of compound K1

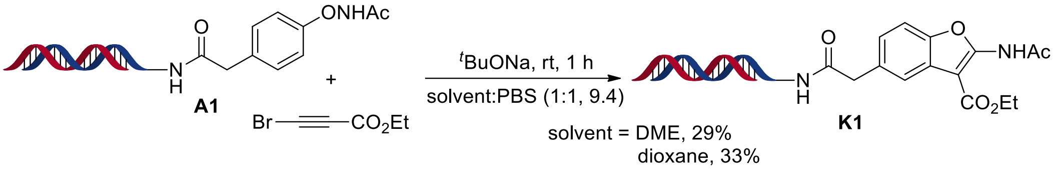

Reaction conditions: **A1** (1 equiv, 0.5 mM in ddH_2O), ^{t}BuONa (200 equiv, 200 mM in ddH_2O), and ethyl 3-bromopropiolate (1000 equiv, 500 mM in DMA) in solvent-PBS (1:1, pH = 9.4, 20 μL) at rt for 1 h without exclusion of air or moisture. The yield of **K1** was determined by LC-MS.

Scheme S8. Synthesis of compound L1

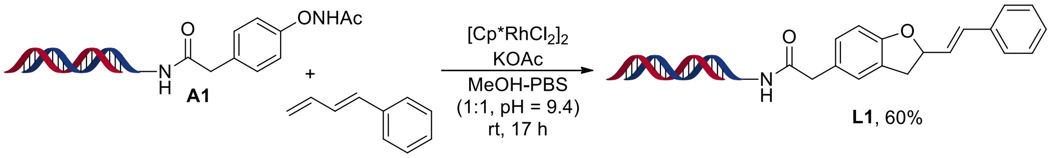

Reaction conditions: **A1** (1 equiv, 0.5 mM in ddH_2O), $[Cp^*RhCl_2]_2$ (20 equiv, 10 mM in DMA), KOAc (200 equiv, 200 mM in ddH_2O), and 1,3-diene (1000 equiv, 500 mM in DMA) in MeOH-PBS (1:1, pH = 9.4, 20 μL) at rt for 17 h without exclusion of air or moisture. The yield of **L1** was determined by LC-MS.

Scheme S9. Synthesis of compound M and N

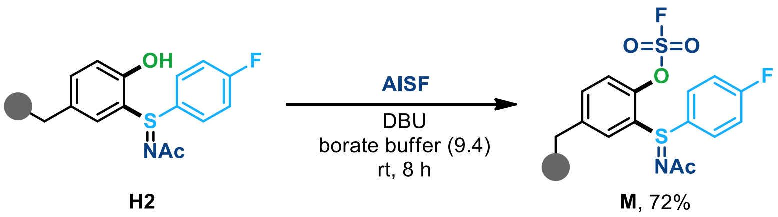

Synthesis of M: To the DNA conjugated phenol **H2** (5 μL, 1 mM), were sequentially added of **AISF** (5 μL, 100 mM), borate buffer (5 μL, 250 mM, pH = 9.4), and 5 DBU

(5 μL, 200 mM). The resultant mixture was vortexed and stood at 25 °C for 8 hours. NaCl (120 μL, 5 M in ddH_2O) and cold ethanol (2.5 times by volume, stored at –20 °C) were sequentially added, and the resultant mixture was incubated at −80 °C for at least 30 min. The mixture was centrifuged at 4 °C for 30 min at 12,000 rpm to remove the supernatant. The resulting pellet (precipitate) was re-dissolved in *dd*H_2O (300 μL) for LC-MS detection.

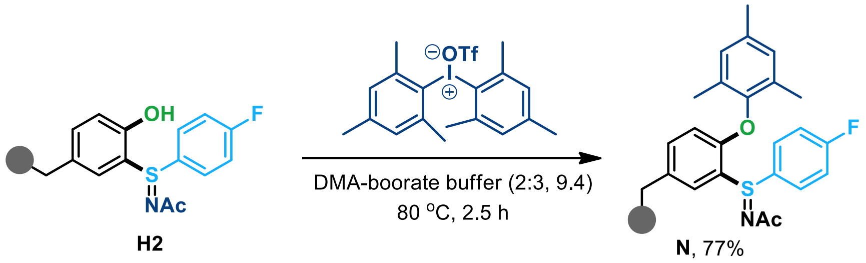

Synthesis of N: To the DNA conjugated phenols (5 μL, 1 mM in *dd*H_2O), were added 25 μL borate buffer (250 mM, pH = 9.4), and diaryliodonium salt (20 μL, 50mM in DMA). The resultant mixture was vortexed and stood at 80 °C for 2.5 hours. 5.0M NaCl (120 μL, 5 M in *dd*H_2O) and cold ethanol (2.5 times by volume, stored at –20 °C) were sequentially added, and the resultant mixture was incubated at −80 °C for at least 30 min. The mixture was centrifuged at 4 °C for 30 min at 12,000 rpm to remove the supernatant. The resulting pellet (precipitate) was re-dissolved in *dd*H_2O (300 μL) for LC-MS detection.

9. Synthesis of off-DNA Products

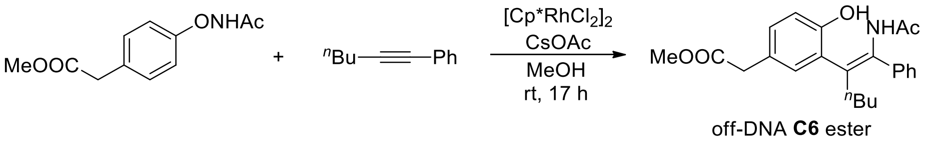

Without any particular precautions to extrude oxygen or moisture, the 4-ethyl ester substituted *N*-phenoxyacetamide (1 equiv), 1-phenyl-1-hexyne (1.2 equiv), $[Cp^*RhCl_2]_2$ (2.5 mol %) and CsOAc (0.25 equiv) were weighted in a 5 mL vial equipped with a stir bar. MeOH (0.4 M) was then added. The reaction was stirred at room temperature for 17 hours. Afterwards, it was diluted with EtOAc and transferred

to a round bottom flask. Silica gel was added to the flask and volatiles were evaporated under reduced pressure. The purification was performed by flash column chromatography on silica gel.

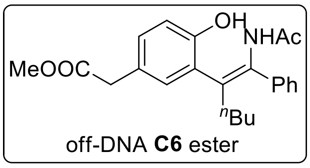

^{1}H NMR (400 MHz, CD$_3$OD): δ 7.44 (d, *J* = 7.2 Hz, 2H), 7.34 (t, *J* = 7.1 Hz, 2H), 7.26 (t, *J* = 6.8 Hz, 1H), 7.09-7.00 (m, 2H), 6.83 (d, *J* = 8.5 Hz, 1H), 3.66 (s, 3H), 3.55 (s, 2H), 2.47-2.43 (m, 2H), 1.74 (s, 3H), 1.29-1.25 (m, 2H), 1.21-1.17 (m, 2H), 0.78-0.69 (m, 3H).

^{13}C NMR (100 MHz, CD$_3$OD): δ 174.5, 172.3, 154.4, 139.8, 136.2, 132.9, 132.0, 130.3, 130.1, 128.9, 128.4, 128.2, 126.4, 116.9, 52.4, 40.8, 33.2, 31.8, 23.6, 22.5, 14.2.

HRMS (ESI) calculated for $C_{23}H_{28}NO_4$ ([M+H$^+$]): 382.2013; found: 382.2019.

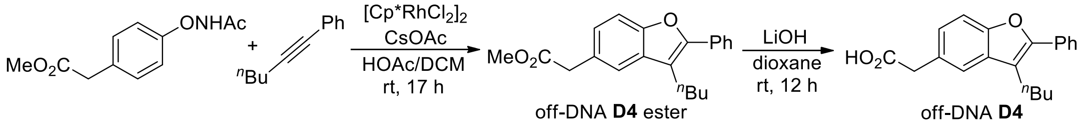

Without any particular precautions to extrude oxygen or moisture, the 4-ethyl ester substituted *N*-phenoxyacetamide (1 equiv), 1-phenyl-1-hexyne (1.2 equiv), [Cp*RhCl$_2$]$_2$ (2.5 mol %), CsOAc (0.25 equiv) and HOAc (1.2 equiv) were weighted into a 5 mL vial equipped with a stir bar, DCM (0.4 M) was then added. The reaction was stirred at room temperature for 17 hours. Afterwards, it was diluted with EtOAc and transferred to a round bottom flask. Silica gel was added to the flask and volatiles were evaporated under reduced pressure. The purification was performed by flash column chromatography on silica gel to give the benzofuran product.

The benzofuran obtained above was dissolved in dioxane (0.2 M), followed by the addition of LiOH (1.2 equiv, 1 M in H_2O). The reaction was allowed to stir at room temperature for 12 h. Afterwards, HCl aqueous solution (1 M) was added and then the

reaction solution was diluted with EtOAc, dried over Na_2SO_4, filtered and washed with EtOAc. The filtrate was concentrated under reduce pressure to afford the off-DNA acid as a white solid.

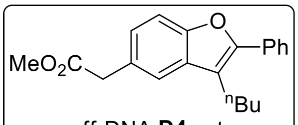

off-DNA **D4** ester

^{1}H NMR (400 MHz, $CDCl_3$): δ 7.80-7.75 (m, 2H), 7.49-7.41 (m, 4H), 7.36 (t, *J* = 7.4 Hz, 1H), 7.20 (dd, *J* = 8.4, 1.5 Hz, 1H), 3.74 (s, 2H), 3.71 (s, 3H), 2.93-2.86 (m, 2H), 1.78-1.68 (m, 2H), 1.53-1.42 (m, 2H), 0.97 (t, *J* = 7.3 Hz, 3H).

^{13}C NMR (100 MHz, $CDCl_3$): δ 172.6, 153.3, 151.3, 131.5, 131.1, 128.8, 128.2, 128.1, 126.9, 125.7, 120.3, 116.6, 111.2, 52.2, 41.4, 32.0, 24.1, 23.1, 14.1.

HRMS (ESI) calculated for $C_{21}H_{23}O_3$ ([M+H$^+$]): 323.1642; found: 323.1641.

^{1}H-^{1}H NOESY (400 MHz, $CDCl_3$):

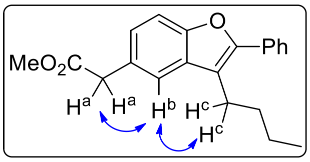

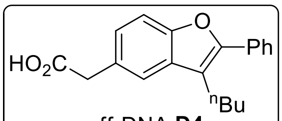

off-DNA **D4**

^{1}H NMR (400 MHz, $CDCl_3$): δ 7.82-7.75 (m, 2H), 7.49-7.45 (m, 4H), 7.38 (t, *J* = 6.9 Hz, 1H), 7.21 (d, *J* = 8.3 Hz, 1H), 3.77 (s, 2H), 2.90 (t, *J* = 7.8 Hz, 2H), 1.80-1.67 (m, 2H), 1.55-1.42 (m, 2H), 0.98 (t, *J* = 7.3 Hz, 3H).

^{13}C NMR (100 MHz, $CDCl_3$): δ 178.4, 153.4, 151.3, 131.4, 131.1, 128.8, 128.2, 127.5, 126.9, 125.7, 120.5, 116.6, 111.2, 41.3, 32.0, 24.1, 23.1, 14.1.

HRMS (ESI) calculated for $C_{20}H_{21}O_3$ ([M+H$^+$]): 309.1485; found: 309.1481.

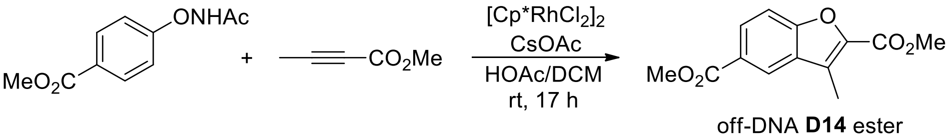

off-DNA **D14** ester

Without any particular precautions to extrude oxygen or moisture, the 4-ester

substituted *N*-phenoxyacetamide (1 equiv), the methyl but-2-ynoate (1.2 equiv), $[Cp^*RhCl_2]_2$ (2.5 mol %), CsOAc (0.25 equiv) and HOAc (1.2 equiv) were weighted into a 5 mL vial equipped with a stir bar, DCM (0.4 M) was then added. The reaction was stirred at room temperature for 17 hours. Afterwards, it was diluted with EtOAc and transferred to a round bottom flask. Silica gel was added to the flask and volatiles were evaporated under reduced pressure. The purification was performed by flash column chromatography on silica gel to give the benzofuran product.

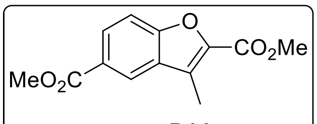

off-DNA **D14** ester

^{1}H NMR (400 MHz, $CDCl_3$): δ 8.33 (d, *J* = 2.4 Hz, 1H), 8.15-8.09 (m, 1H), 7.52 (dd, *J* = 8.7, 2.4 Hz, 1H), 3.98 (s, 3H), 3.94 (d, *J* = 0.8 Hz, 3H), 2.61-2.57 (m, 3H).

^{13}C NMR (100 MHz, $CDCl_3$): δ 166.8, 160.5, 156.8, 142.0, 129.2, 129.1, 126.3, 125.7, 123.9, 112.1, 52.4, 52.3, 9.4.

HRMS (ESI) calculated for $C_{13}H_{13}O_5$ ($[M+H^+]$): 249.0757; found: 249.0753.

^{1}H-^{1}H NOESY (400 MHz, $CDCl_3$):

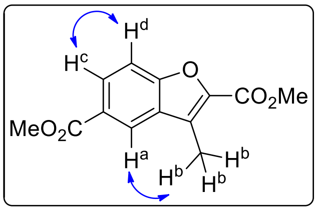

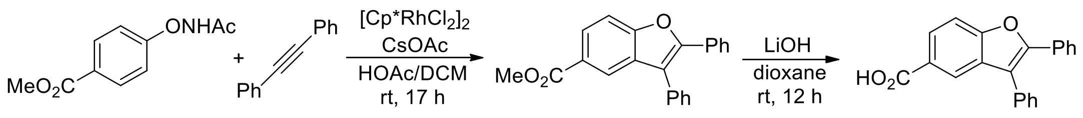

off-DNA **D13**

Without any particular precautions to extrude oxygen or moisture, the 4-ester substituted *N*-phenoxyacetamide (1 equiv), the 1,2-diphenylethyne (1.2 equiv), $[Cp^*RhCl_2]_2$ (2.5 mol%), CsOAc (0.25 equiv) and HOAc (1.2 equiv) were weighted in a 5 mL vial equipped with a stir bar. DCM (0.4 M) was then added. The reaction was stirred at room temperature for 17 hours. Afterwards, it was diluted with EtOAc and transferred to a round bottom flask. Silica gel was added to the flask and volatiles

were evaporated under reduced pressure. The purification was performed by flash column chromatography on silica gel to give the benzofuran product. This is a known compound and the characterization data was in line with literature precedent.[S2]

The benzofuran obtained above was dissolved in dioxane (0.2 M), followed by the addition of LiOH (1.2 equiv, 1 M in H_2O). The reaction was allowed to stir at rt for 12 h. Afterwards, HCl (1 M) was added and then the reaction solution was diluted with EA, dried over Na_2SO_4, filtered off and washed with EA. The filtrate was concentrated under reduce pressure to afford the off-DNA **D13** acid as a white soild.

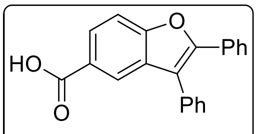

off-DNA **D13**

^{1}H NMR (400 MHz, DMSO-*d*6): δ 8.00 (dd, *J* = 6.9, 1.6 Hz, 2H), 7.81-7.71 (m, 1H), 7.63-7.45 (m, 7H), 7.43-7.34 (m, 3H).

^{13}C NMR (100 MHz, DMSO-*d*6): δ 167.3, 155.7, 151.3, 131.3, 129.7, 129.4, 129.3, 128.9, 128.3, 126.7, 126.4, 121.6, 117.4, 111.4.

HRMS (ESI) calculated for $C_{21}H_{15}O_3$ ([M+H$^+$]): 315.1016; found: 315.1018.

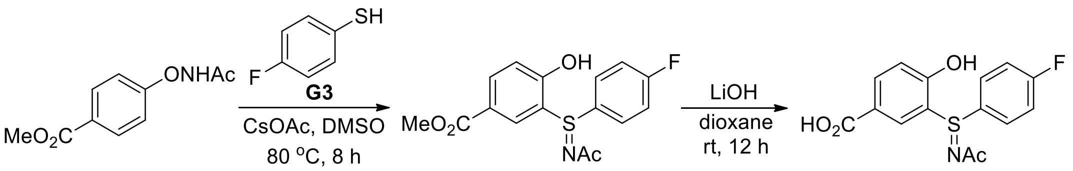

off-DNA **H5**

Without any particular precautions to extrude oxygen or moisture, the 4-ester substituted *N*-phenoxyacetamide (15.1 mg, 0.1 mmol), **G3** (25.6 mg, 0.2 mmol), CsOAc (19.2 mg, 0.1 mmol) were weighed in a 5 mL vial equipped with a stir bar, to which was added DMSO (1 mL). The reaction vessel was stirred at 80 °C for 8 h. Then the mixture was concentrated under vacuum and the residue was purified by column chromatography on silica gel to obtain the *ortho*-sulfiliminyl phenol product as a white solid.

The *ortho*-sulfiliminyl phenol obtained above was dissolved in dioxane (0.2 M), followed by the addition of LiOH (1.2 equiv, 1 M in H_2O). The reaction was allowed

to stir at rt for 12 h. Afterwards, HCl (1 M) was added and then the reaction solution diluted with EA, dried over Na_2SO_4, filtered off and washed with EA. The filtrate was concentrated under reduce pressure to afford the off-DNA **H5** as a white soild.

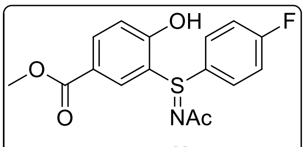

off-DNA **H5** ester

^{1}H NMR (400 MHz, DMSO-*d*6): δ 8.37 (d, *J* = 2.2 Hz, 1H), 7.97 (dd, *J* = 8.5, 2.1 Hz, 1H), 7.81-7.75 (m, 2H), 7.41-7.34 (m, 2H), 7.05 (d, *J* = 8.4 Hz, 1H), 3.83 (s, 3H), 2.01 (s, 3H).

^{13}C NMR (100 MHz, DMSO-*d*6): δ 180.0, 165.2 (d, *J* = 22.8 Hz), 162.6, 160.2, 134.5, 131.8, 130.9 (d, *J* = 37.2 Hz), 128.3, 121.1, 120.8, 117.1, 116.8 (d, *J* = 31.2 Hz), 52.2, 24.4.

^{19}F NMR (376 MHz, DMSO-*d*6): -107.37.

HRMS (ESI) calculated for $C_{16}H_{15}FNO_4S$ ([M+H$^+$]): 336.0700; found: 336.0699.

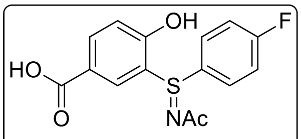

off-DNA **H5**

^{1}H NMR (400 MHz, DMSO-*d*6): δ 8.35 (d, *J* = 2.2 Hz, 1H), 7.96 (dd, *J* = 8.5, 2.1 Hz, 1H), 7.82-7.75 (m, 2H), 7.42-7.35 (m, 2H), 6.99 (d, *J* = 8.6 Hz, 1H), 2.01 (s, 3H).

^{13}C NMR (100 MHz, DMSO-*d*6): δ 180.0, 166.3, 165.1, 162.6, 159.5, 134.7, 131.7, 130.9 (d, *J* = 36.8 Hz), 128.5, 122.65, 120.7, 117.0 (d, *J* = 90.8 Hz), 117.1, 116.9, 24.4.

^{19}F NMR (376 MHz, DMSO-*d*6): -107.38.

HRMS (ESI) calculated for $C_{15}H_{13}FNO_4S$ ([M+H$^+$]): 322.0544; found: 322.0544.

Scheme S9. Resynthesis of H5-1 from off-DNA substrates

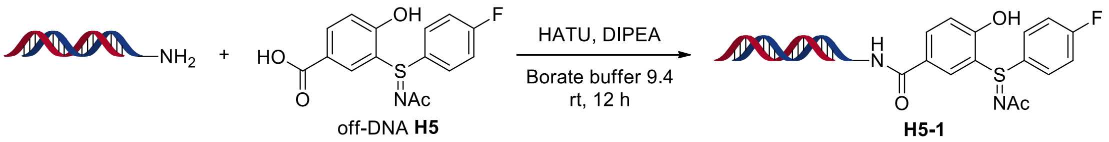

To a solution of DNA headpiece ($HP-NH_2$, 2 nmol) in 4 μL boric buffer (pH = 9.4, 5 mM) was added a mixture of HATU (20 μL, 200 mM in DMA), the off-DNA substrate **H5** (20 μL, 200 mM in DMA) and DIPEA (20 μL, 200 mM in DMA). The resultant mixture was vortexed and stood at 25 °C for 12 h. Aqueous NaCl (26 μL, 5 M) and cold EtOH (780 μL) were sequentially added and the resultant mixture was stored at -80 °C for 30 min. The mixture was centrifuged at 4 °C for 30 min at 12000 rpm before the resultant supernatant was removed. The pellet (precipitate) was dissolved in deionized water for LC-MS detection.

Scheme S10. Synthesis of H5

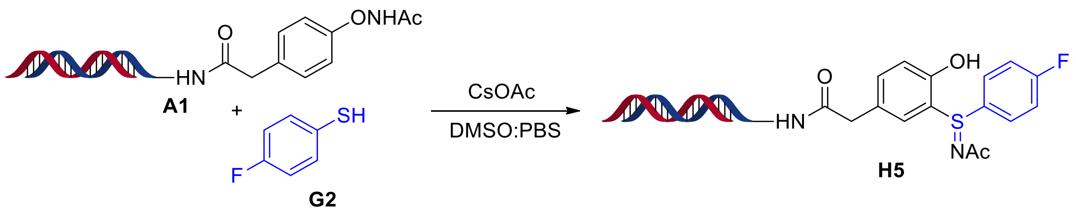

To the **A1** (4 μL, 0.5 mM in ddH_2O) was added 1000 equiv of **G2** (4 μL, 500 mM in DMA) and 200 equiv of CsOAc (2 μL, 200 mM in ddH_2O) in DMSO-PBS (10 μL, 1:1, pH = 9.4). The mixture was vortexed and stood at 80 °C for 8 h. After the reaction was completed, added scavenger sodium diethyldithiocarbamic acid (3.2 μL, 500 mM in ddH_2O) to the mixture, and heated the reaction mixture at 60 °C for at least 30 min. Then the mixture was centrifuged at 4 °C for 30 min at 4800 rpm, and the resultant supernatant was collected. Add aqueous 5 N NaCl solution (10% by volume) and cold ethanol (2.5 times by volume, ethanol stored at -20°C) to the resultant supernatant. The mixture was vortexed and stored at a -80 °C freezer for more than 1 h. The sample was centrifuged for around 30 min at 4 °C in a microcentrifuge at 12000 rpm. The above supernatant was removed and the pellet (precipitate) was dissolved in deionized water for LC-MS detection.

10. Parallel Injection Experiment and Analysis

Samples were dissolved in an appropriate amount of distilled and deionized water

(ddH_2O) and injected or co-injected into a reverse-phase chromatography column (Xbridge Oligonucleotide BEH C18 column, 1.7 μm, 2.1×50 mm). The elution was carried out as followings: 5-30% solvent B over 30 min, 0.4 mL/min, λ = 260 nm; solvent A: 0.75% v/v hexafluoroisopropanol/ 0.038% v/v triethylamine in methanol/water = 5/95; solvent B: 0.75% v/v hexafluoroisopropanol/ 0.038% v/v triethylamine in methanol/water = 90/10. The effluents were analyzed by a Xevo G2-XS Q-TOF with electrospray ionization source.

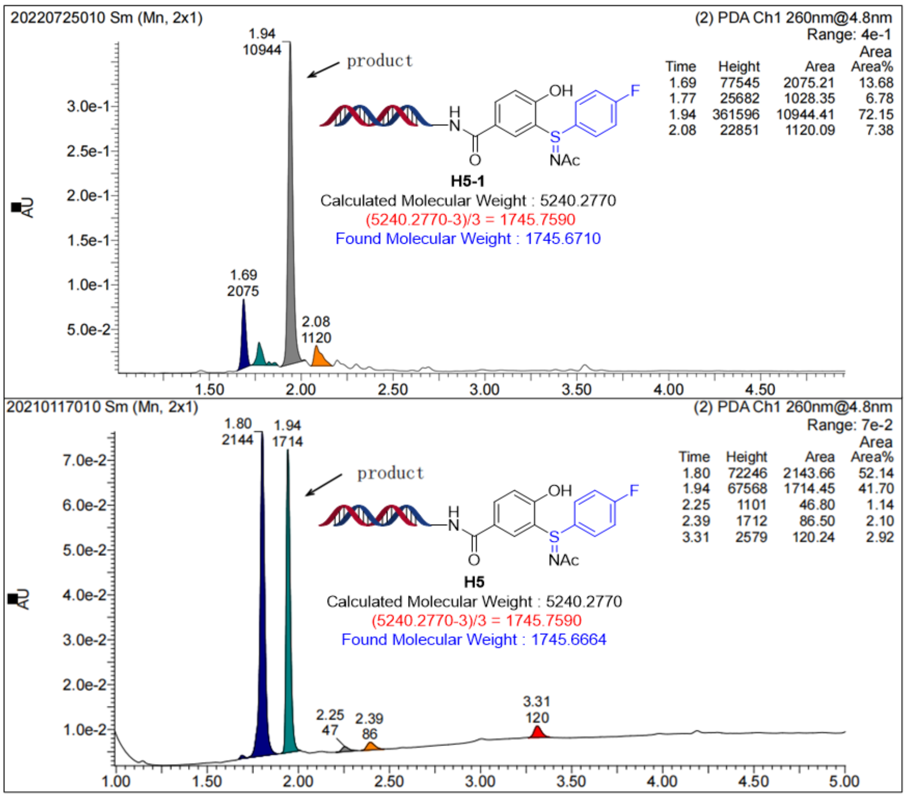

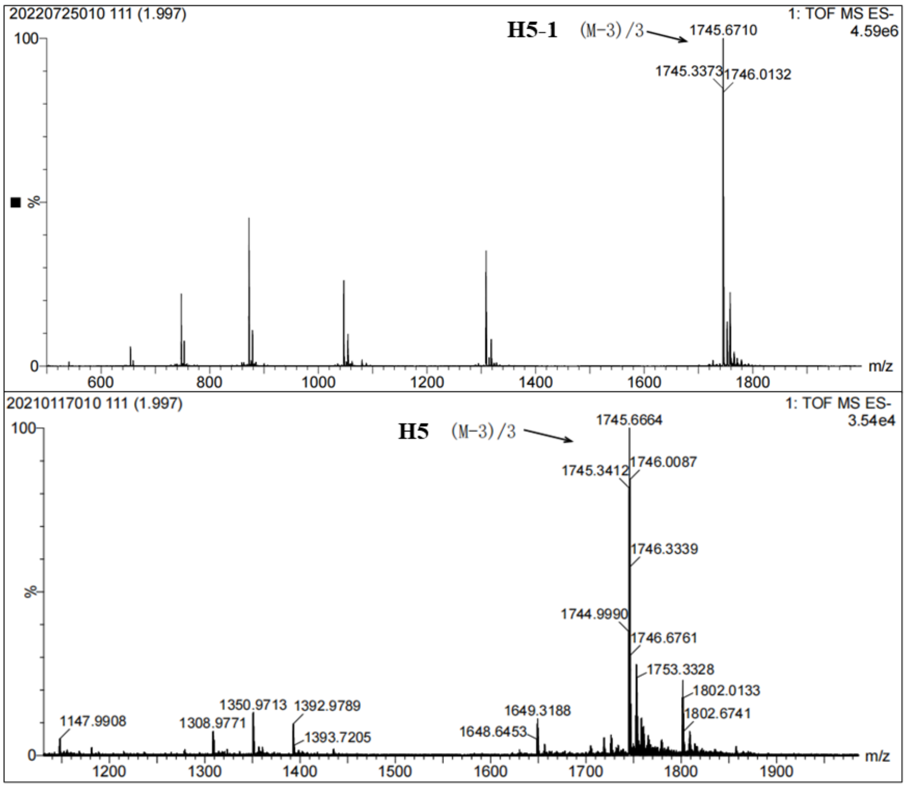

Figure S2. Parallel injection experiment of H5 with resynthesized H5-1

Scheme S11. Synthesis of D4 from A1 or HP-NH2 and off-DNA D4.

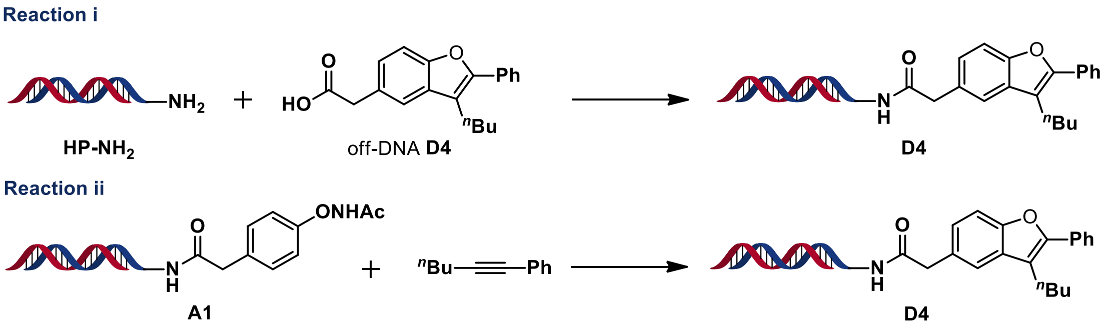

Reaction i: To a solution of DMSO (50 μL) was added the acid off-DNA **D4** (12 μL, 200 mM in DMA), EDCI (12 μL, 200 mM in DMSO), NHS (12 μL, 150 mM in DMSO:*dd*H_2O = 2:1) and 50 μL DMSO. The resulting mixture was vortexed and incubated at 40 °C for 30 min, after that a solution of DNA headpiece (10 nmol, 1 mM in *dd*H_2O) and MOPS buffer (40 μL, 200 mM, pH = 8) was added to the mixture. The resulting mixture was vortexed and stood at 25°C for 4 hours. Then the supernatant was collected. 5 N NaCl solution (10% by volume) and cold ethanol (2.5 times by volume, stored at -20°C) were added to the resultant supernatant. The mixture was vortexed and stored at -80 °C for more than 30 min. The sample was

centrifuged for around 30 min at 4 °C in a microcentrifuge at 14000 rpm. The above supernatant was removed and the pellet (precipitate) was dissolved in *dd*H_2O for LC-MS detection.

Yield: 88%
Exact mass: 5227.3605
Triple charged mass (M-3)/3, calculated 1741.4535; observed 1741.1808.

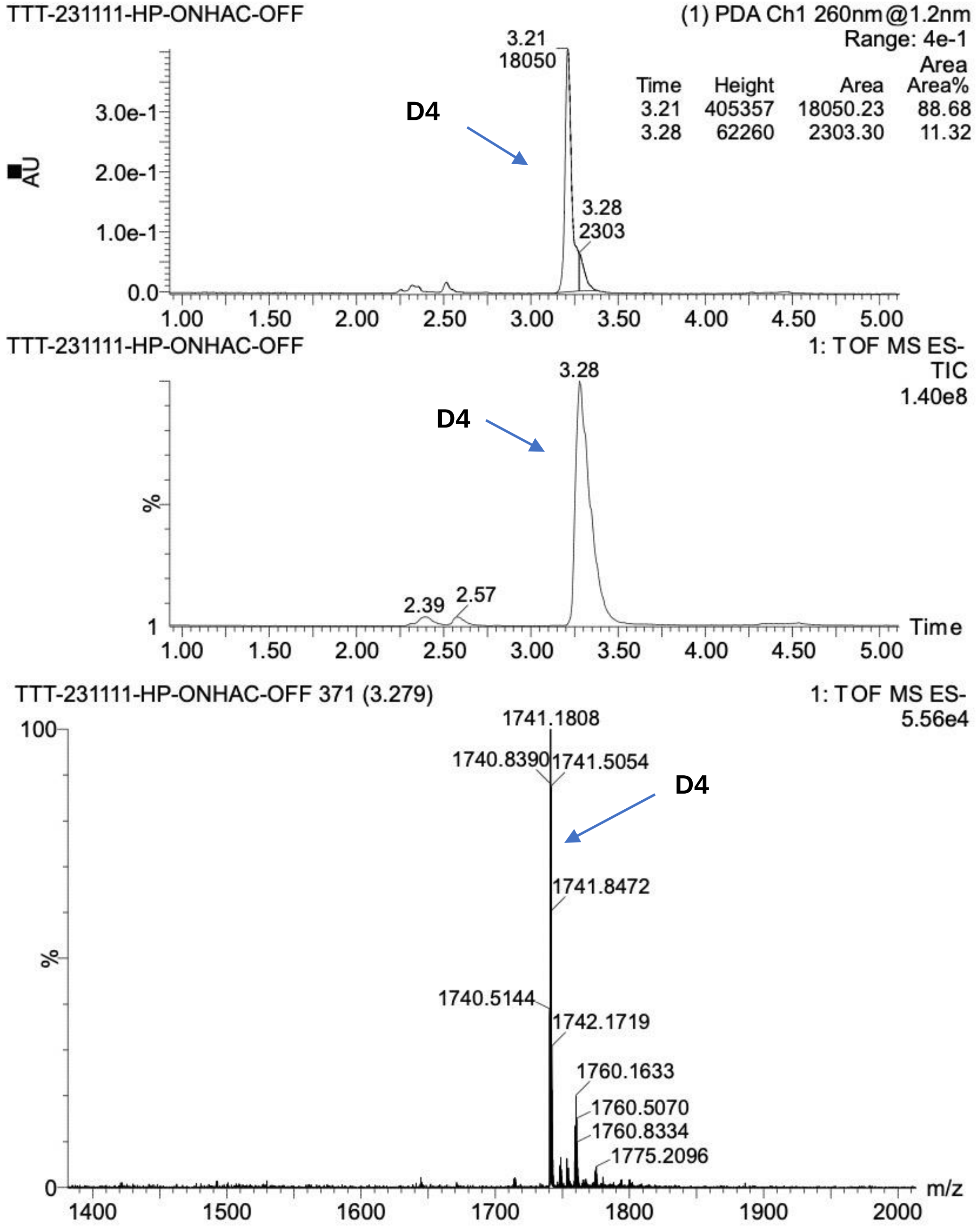

Reaction ii was performed according to the General Procedure for the Synthesis of DNA-Conjugated Benzofurans.

Yield: 81%
Exact mass: 5227.3605

Triple charged mass (M-3)/3, calculated 1741.4535; observed 1741.1808.

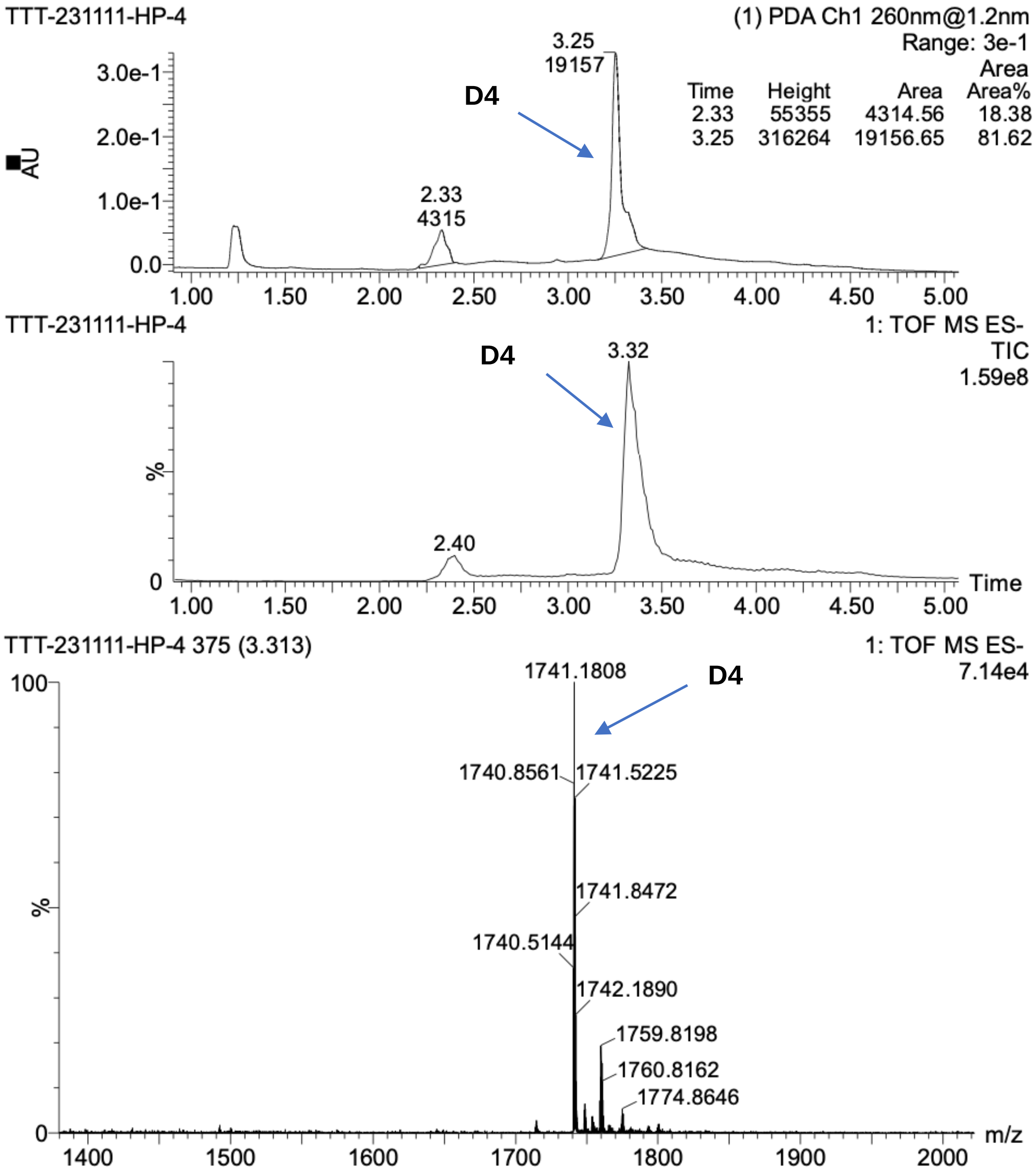
TTT-231111-HP-4
(1) PDA Ch1 260nm@1.2nm
Range: 3e-1
Time Height Area Area%
2.33 55355 4314.56 18.38
3.25 316264 19156.65 81.62
3.25
19157
2.33
4315
D4
AU
TTT-231111-HP-4
1: TOF MS ES-
TIC
1.59e8
D4
3.32
2.40
%
Time
TTT-231111-HP-4 375 (3.313)
1: TOF MS ES-
7.14e4
1741.1808
D4
1740.8561
1741.5225
1741.8472
1740.5144
1742.1890
1759.8198
1760.8162
1774.8646
%
m/z

Co-injection

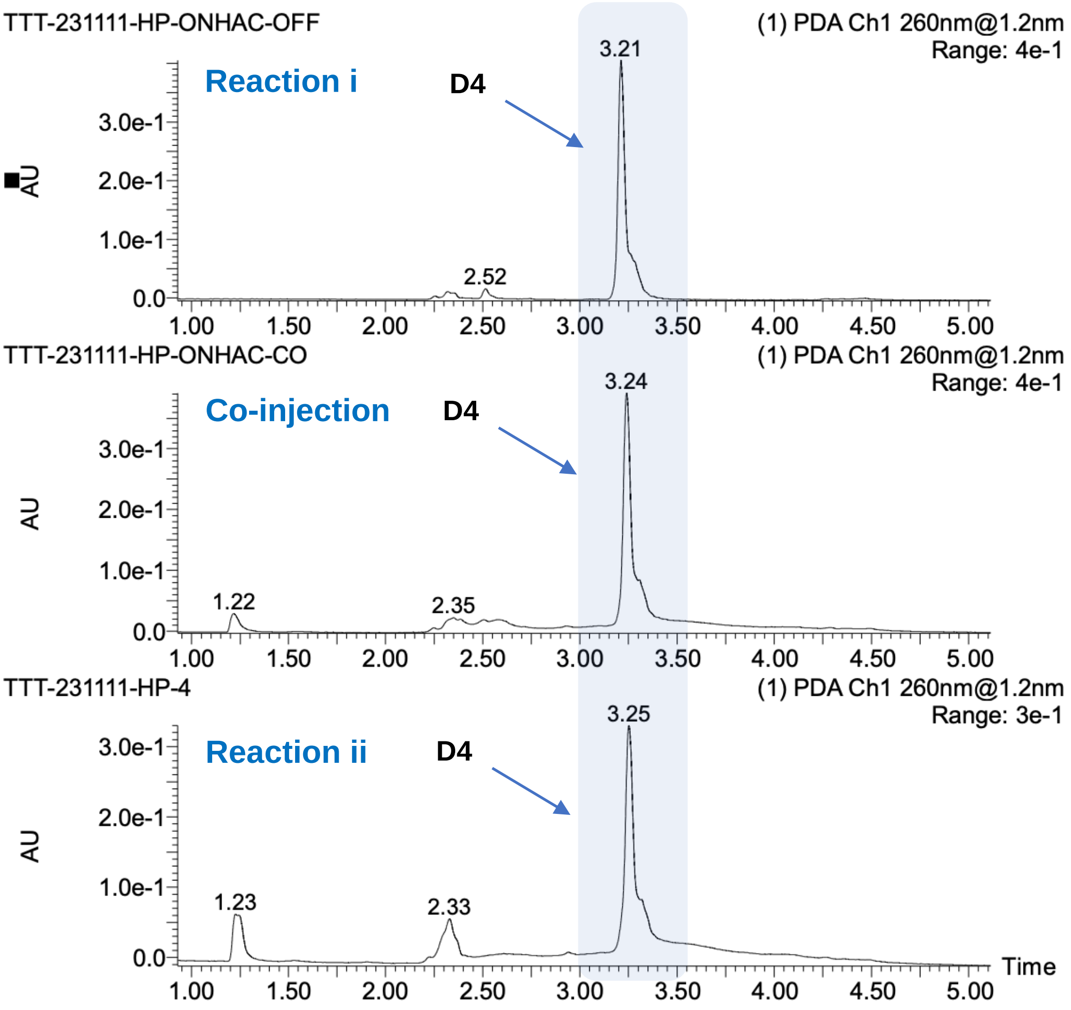

11. Evaluation of the DNA Tags Degradation

DNA ligation and sequencing: The ligation sample was prepared by mixing 5 μL on-DNA reaction product **C1** or **D1** (1 mM in ddH_2O) with 5 μL 50 bp oligo DNA (1 mM in ddH_2O, prepared by annealing of 2 mM 50 bp-F and 2mM 50 bp-R). To 0.5 nmol sample in pellet form, 1 μL 10 × T4 DNA reaction buffer (NEB, B0202S), 1 μL T4 DNA ligase (NEB, M0202L), 7 μL ddH_2O were added. The reaction mixtures were kept at room temperature for two hours. Reaction samples were then heated at 65 °C for 10 minutes to denature the T4 DNA ligase. Gel electrophoresis is executed by using 15% TBE acrylamide gel (5.25 mL 40% acryl (29 : 1), 7.2 mL ddH_2O, 1.4 mL 10 × TBE, 140 μL APS, 6 μL TEMED). The gel box (Bio-Rad, Mini-PROTEAN®

Tetra electrophoresis) was filled with 0.5 × TAE buffer until the gel was covered. The first lane of the gel was loaded with a DNA ladder, followed by loading 50 ng of DNA sample into each lane. Gel was run at 110 V for 90 min and was stained with 3000 × GelRed from Tanon™ (170-3001) in 15 mL 0.5 × TBE buffer for 15 min. DNA fragments were visualized under a UV light device.

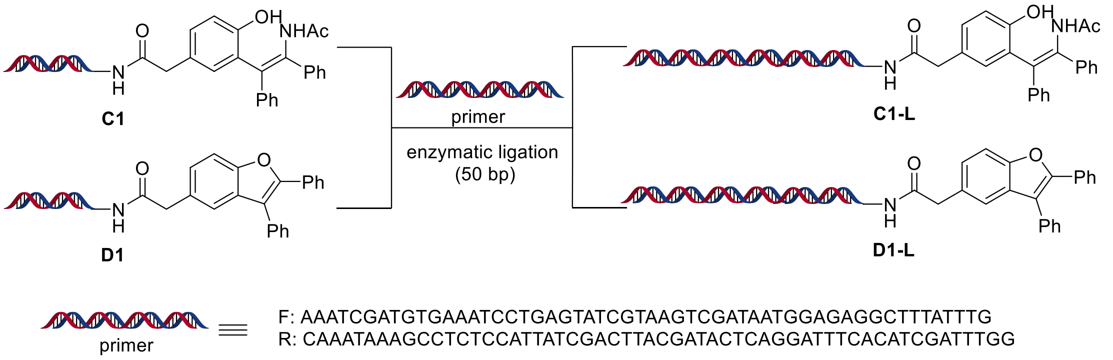

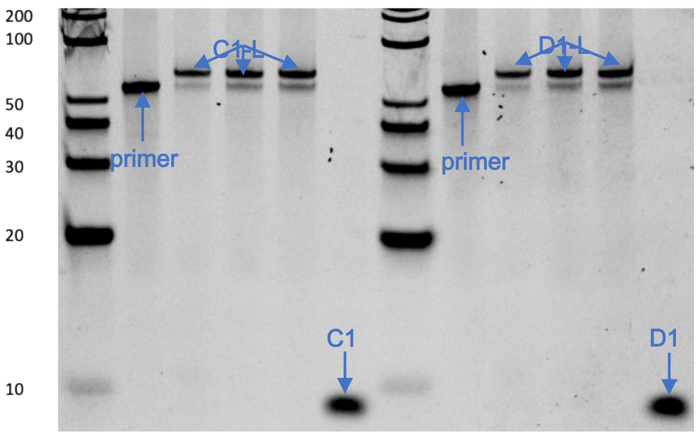

Figure S3. DNA ligation reaction analysis for synthesis of **C1-L** and **D1-L**.

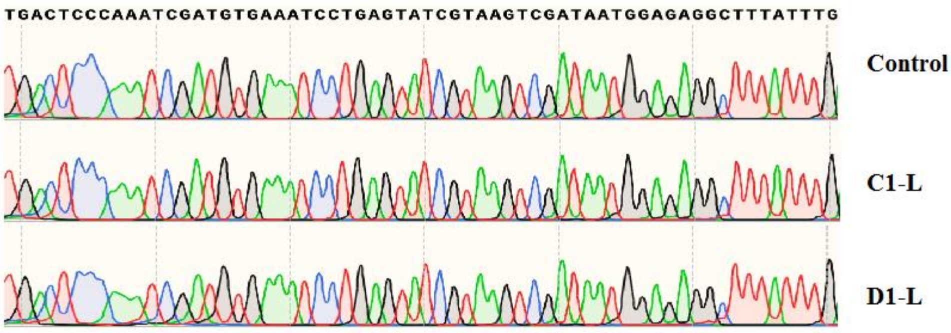

Figure S4. All DNA sample were amplificated by two rounds' PCR steps to reach 184 bp length before sequencing. Following manufacturer's guidelines, 1 ng of former DNA ligation sample was mix with 1 μL of 10 μM primer PCR1-F and PCR1-R, 25 μL 2 × Primer STAR Max DNA Polymerase (Takara, R045A) to reach a 50 μL system finally. PCR cycles as follows: 98 °C heat activation for 1 min followed by 34 cycles of 98 °C denaturation for 10 seconds, 60 °C annealing for 5 seconds and extension at 72 °C for 10 seconds. DNA sample was evaluation by 2% DNA gel and extracted by NucleoSpin Gel Clean-up Mini kit (MACHEREY-NAGEL, 740609.50). After PCR1, PCR2 was executed as the same step by using PCR1 purified sample. DNA sequencing was sent to Sanger sequencing by using primer Sequencing-F.

PCR1-F: CTCTTTCCCTACACGACGCTCTTCCGATCTTGACTCCCAAATC
PCR1-R: GTGACTGGAGTTCAGACGTGTGCTCTTCCGATCAAATAAAGCCTC
PCR2-F: AATGATACGGCGACCACCGAGATCTACACTCTTTCCCTACACGACGCTC
PCR2-R: CAAGCAGAAGACGGCATACGAGATGTCGTGATGTGACTGGAGTTCAGACG TGTGC
Sequencing-F: AATGATACGGCGACC

qPCR Analysis: qPCR was performed on the Applied Biosystems QuantStudio 7 Real-Time PCR System using 96-well plates. The sample mixtures of 20 μL total volume contained the following: 7 μL ddH_2O, 1 μL primer mix (5 μM each, reverse and forward primer), 2 μL diluted DNA sample and 10 μL Bimake™ 2 × SYBR Green qPCR master mix (Low ROX; B21702). The PCR reaction was carried out with the following thermocycling program: 95 °C, 30 s; 40 cycles of (95 °C, 15 s; 55 °C, 30 s; 72 °C, 30 s). Every assay was repeated at least three times.

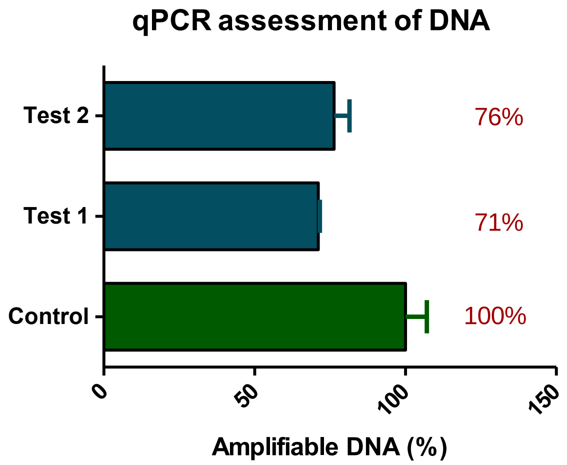

Figure S5. qPCR analysis of residual amplifiable material after the reaction condition of synthesis of **C1**. In a 100 μL polypropylene plate, a solution of a full-length DNA-encoded library (ca. 0.5 mM in water, 4 μL, ca. 2 nmol) was combined with 30

μL ddH_2O (control) or combined with $[Cp^*RhCl_2]_2$ (20 equiv, 10 mM in DMA), CsOAc (200 equiv, 200 mM in ddH_2O), alkyne **2a** (1000 equiv, 500 mM in DMA) in MeOH-PBS (pH=9.4, 20 μL) (Test 1 and Test 2). The plate was sealed (adhesive aluminum foil), briefly vortexed, and incubated at 60°C for 8 hours. All conditions were tested in triplicate (3 × 3 wells in total). Each sample was then diluted (three times 1:100, 2 μL sample in 198 μL ddH_2O), and desalted by gel filtration (GE healthcare Illustra, G-25 microspin columns) as per the manufacturer's instruction. Amount of amplifiable material was determined by qPCR analysis in technical sextuplets.

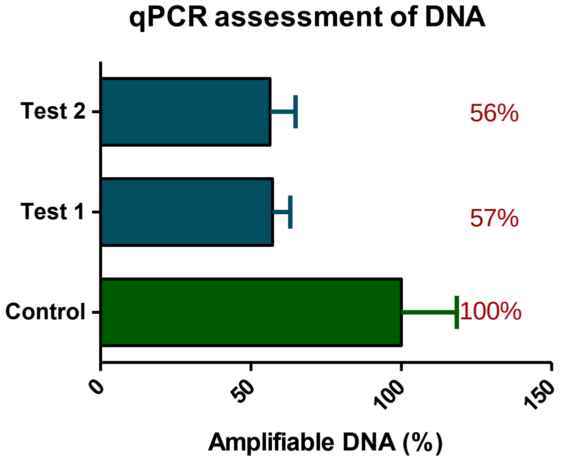

Figure S6. qPCR analysis of residual amplifiable material after the reaction condition of **D1**. In a 100 μL polypropylene plate, a solution of a full-length DNA-encoded library (ca. 0.5 mM in water, 4 μL, ca. 2 nmol) was combined with 30 μL ddH_2O (control) or combined with $[Ru(p\text{-cymene})Cl_2]_2$ (20 equiv, 10 mM in DMA), KOPiv (200 equiv, 200 mM in ddH_2O), alkyne **2b** (1000 equiv, 500 mM in DMA) in MeCN-PBS (1:1, pH = 9.4, 20 μL) (Test 1 and Test 2). The plate was sealed (adhesive aluminum foil), briefly vortexed, and incubated at 60 °C for 17 hours. All conditions were tested in triplicate (3 × 3 wells in total). Each sample was then diluted (three times 1:100, 2 μL sample in 198 μL ddH_2O), and desalted by gel filtration (GE healthcare Illustra, G-25 microspin columns) as per the manufacturer's instruction. Amount of amplifiable material was determined by qPCR analysis in technical sextuplets.

Copies of NMR Spectra

^{1}H-NMR of off-DNA C6 ester (400 MHz, CD_3OD)

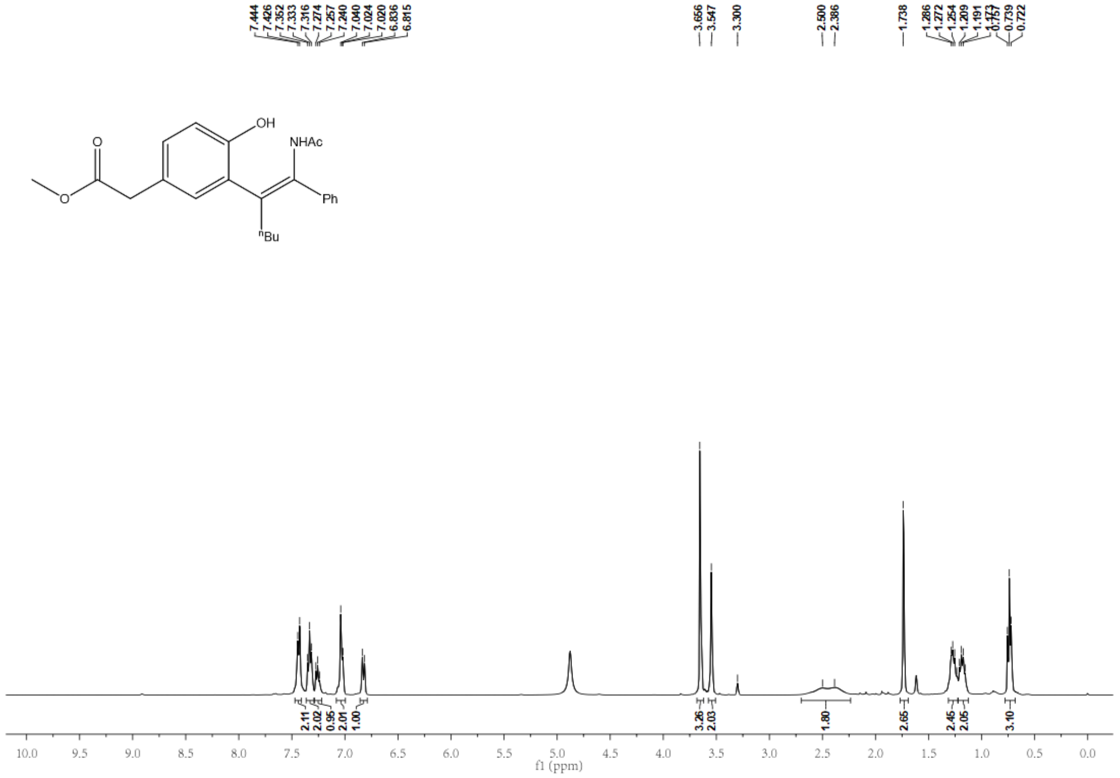

^{13}C-NMR of off-DNA C6 ester (100 MHz, CD_3OD)

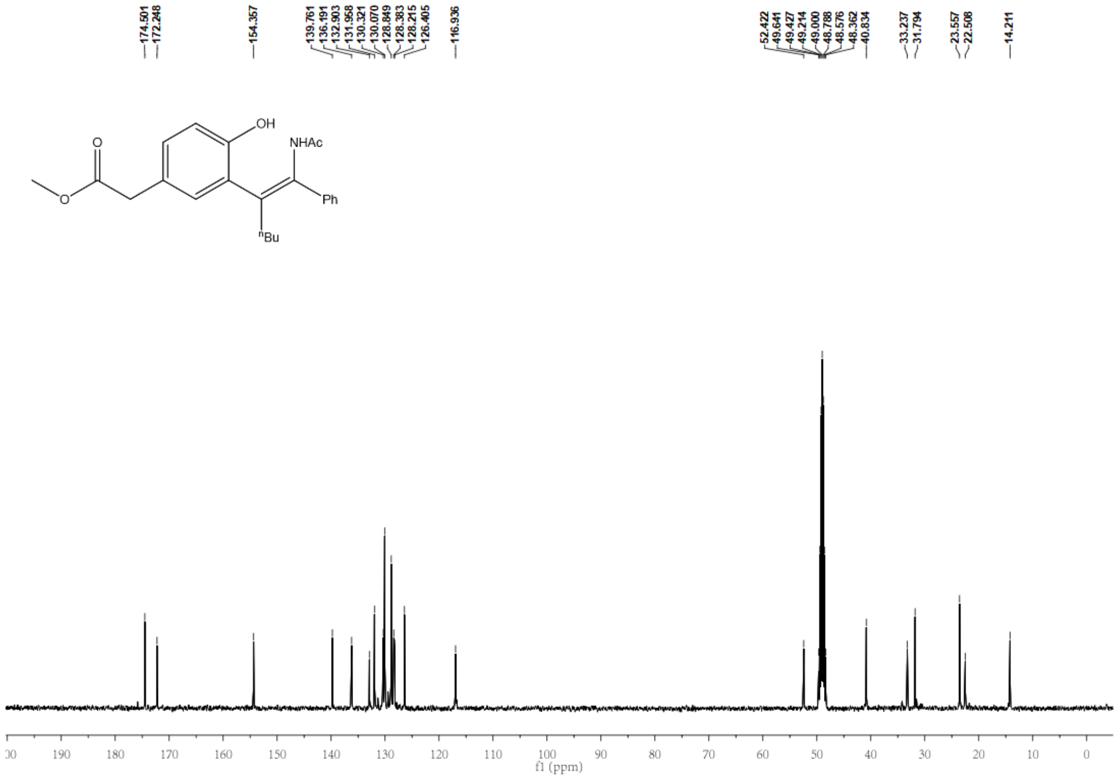

^{1}H-NMR of off-DNA D4 ester (400 MHz, $CDCl_3$)

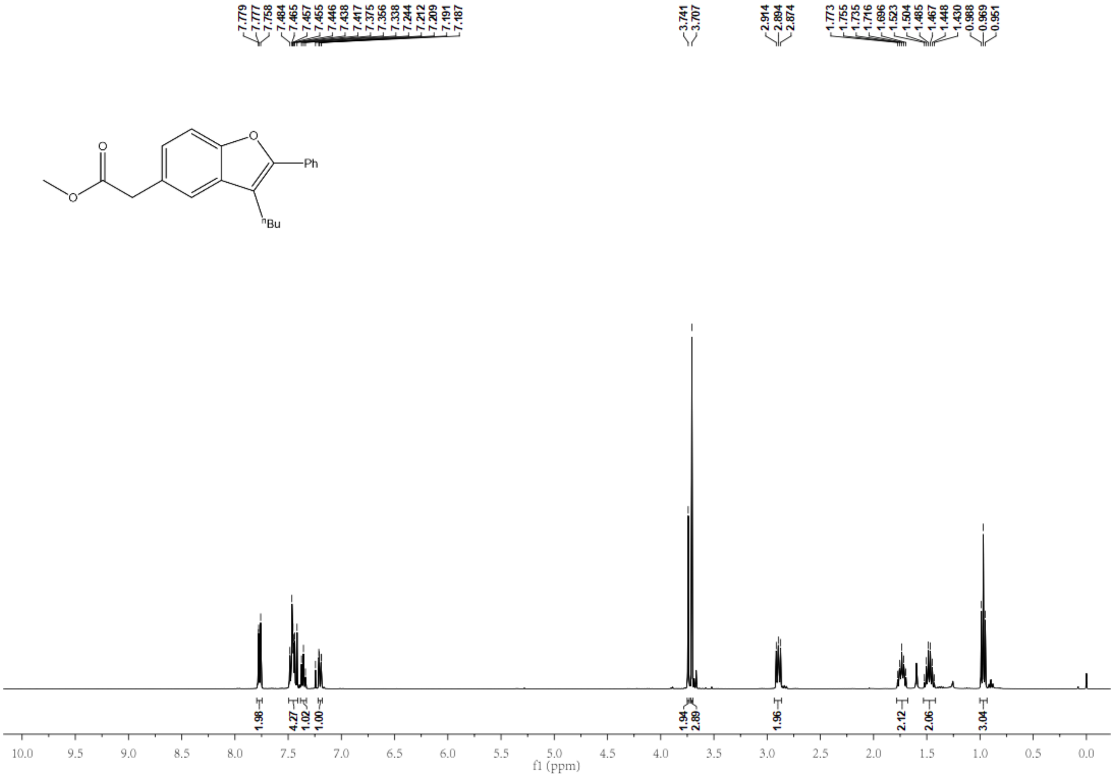

^{13}C-NMR of off-DNA D4 ester (100 MHz, $CDCl_3$)

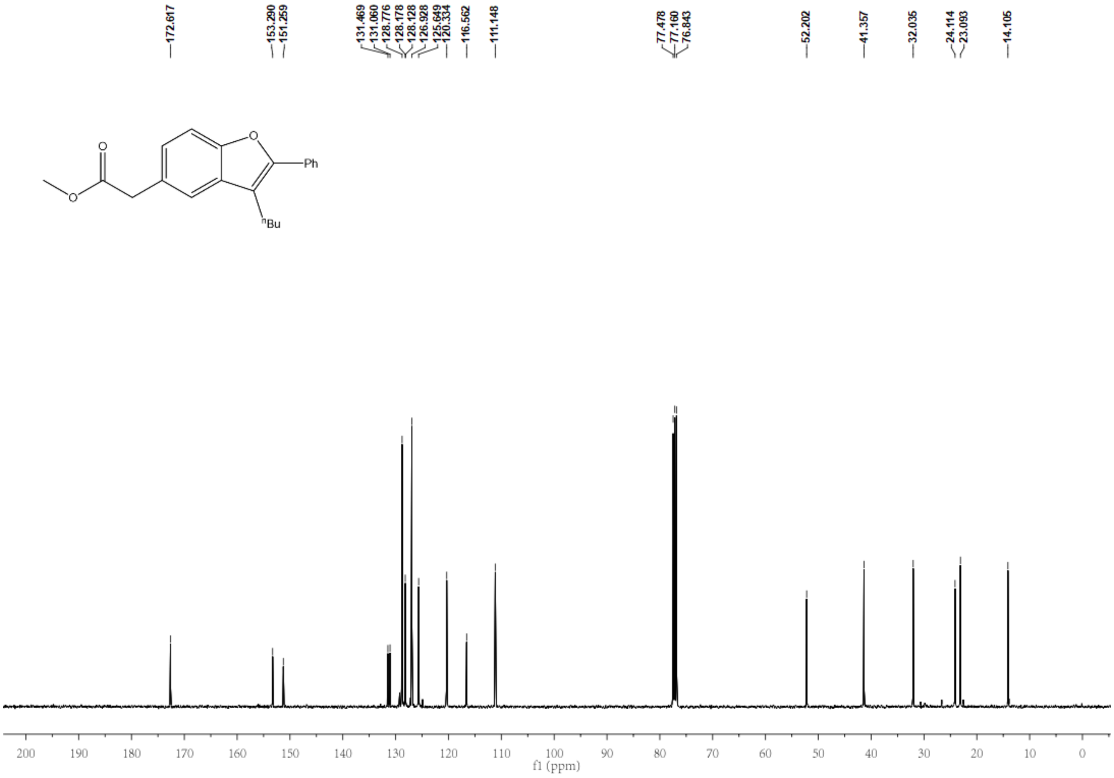

^{1}H-^{1}H NOESY of off-DNA D4 ester (400 MHz, CD_3OD):

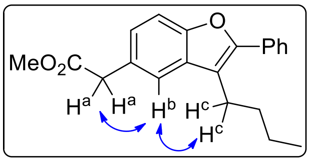

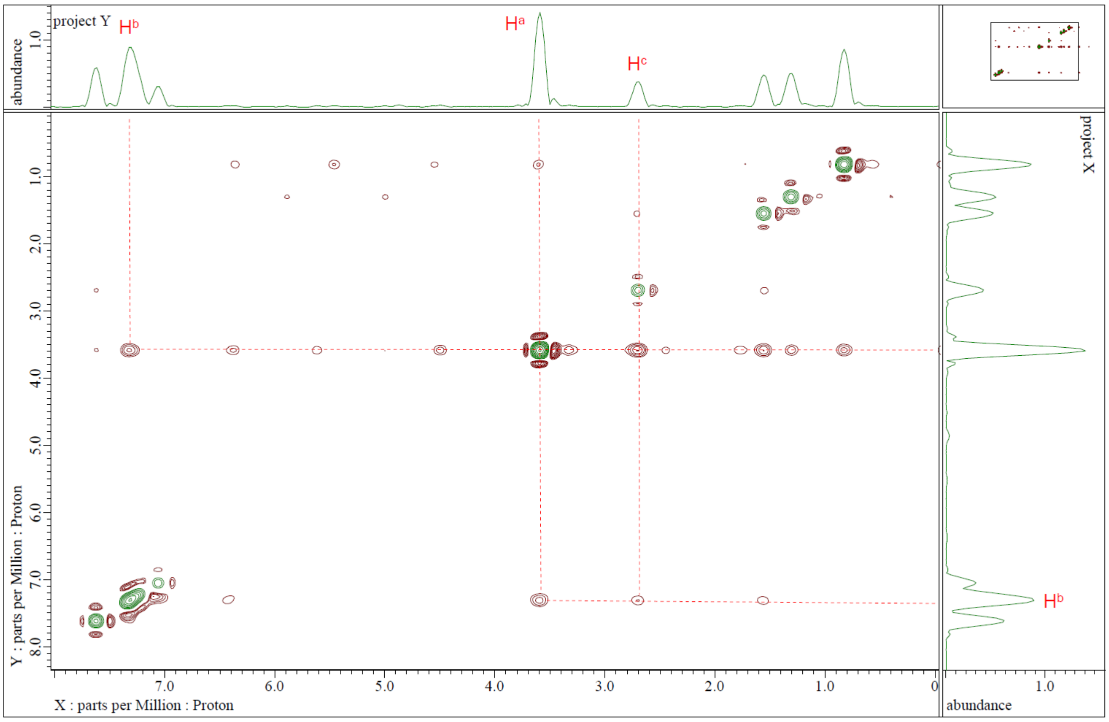

^{1}H-NMR of off-DNA D4 (400 MHz, CDCl$_3$)

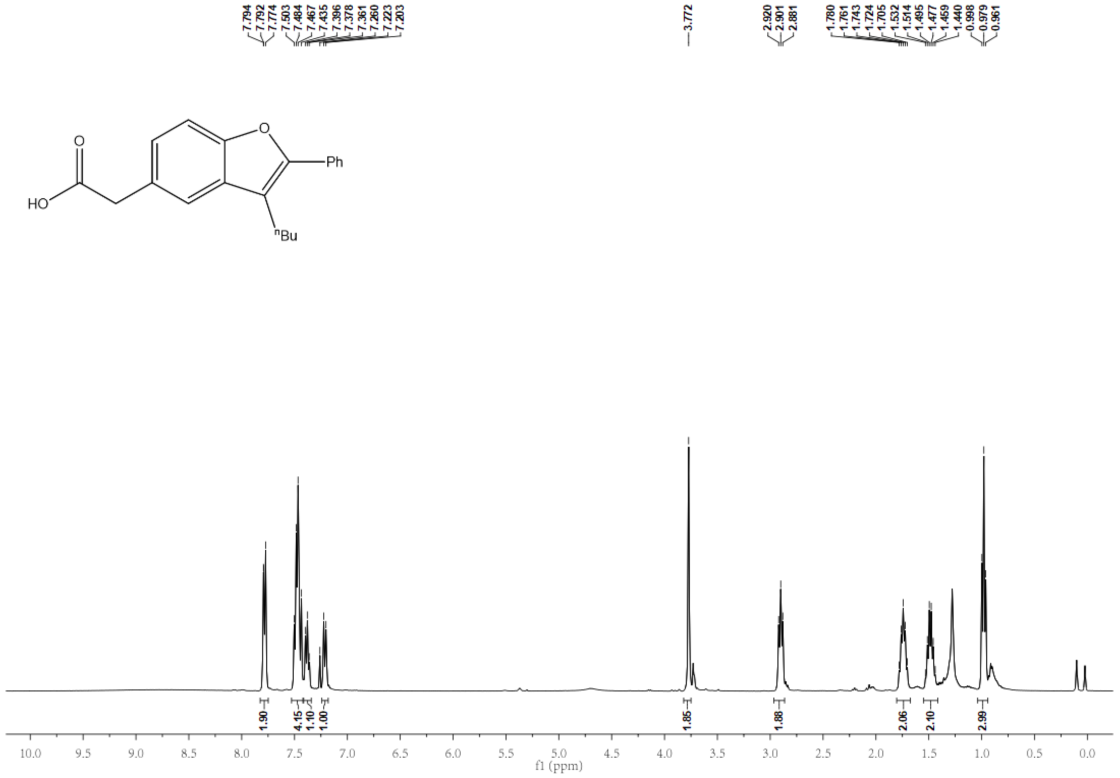

^{13}C-NMR of off-DNA D4 (100 MHz, CDCl$_3$)

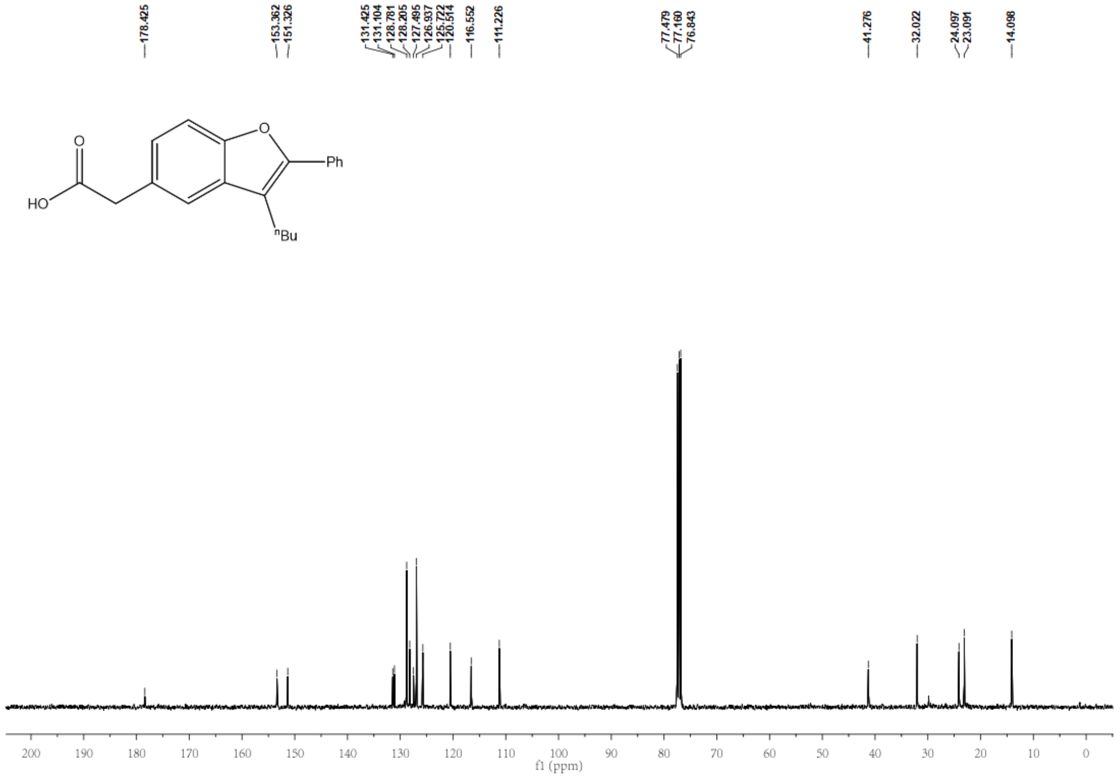

^{1}H-NMR of off-DNA D14 ester (400 MHz, $CDCl_3$)

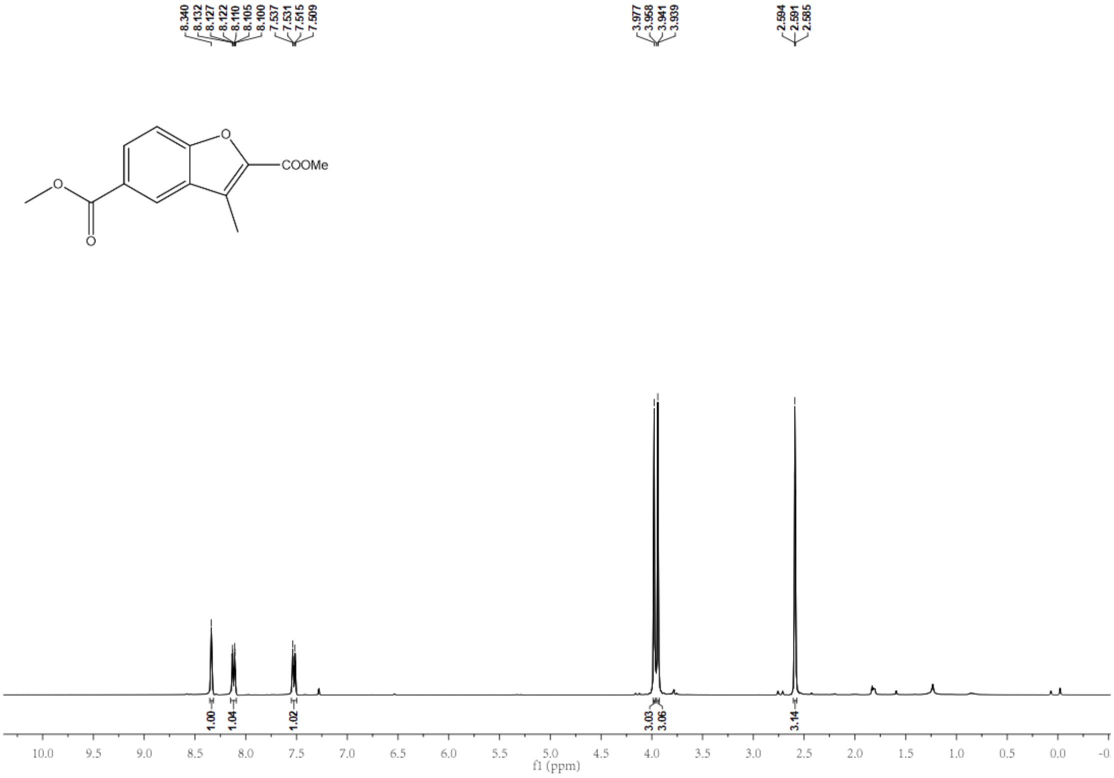

^{13}C-NMR of off-DNA D14 ester (100 MHz, $CDCl_3$)

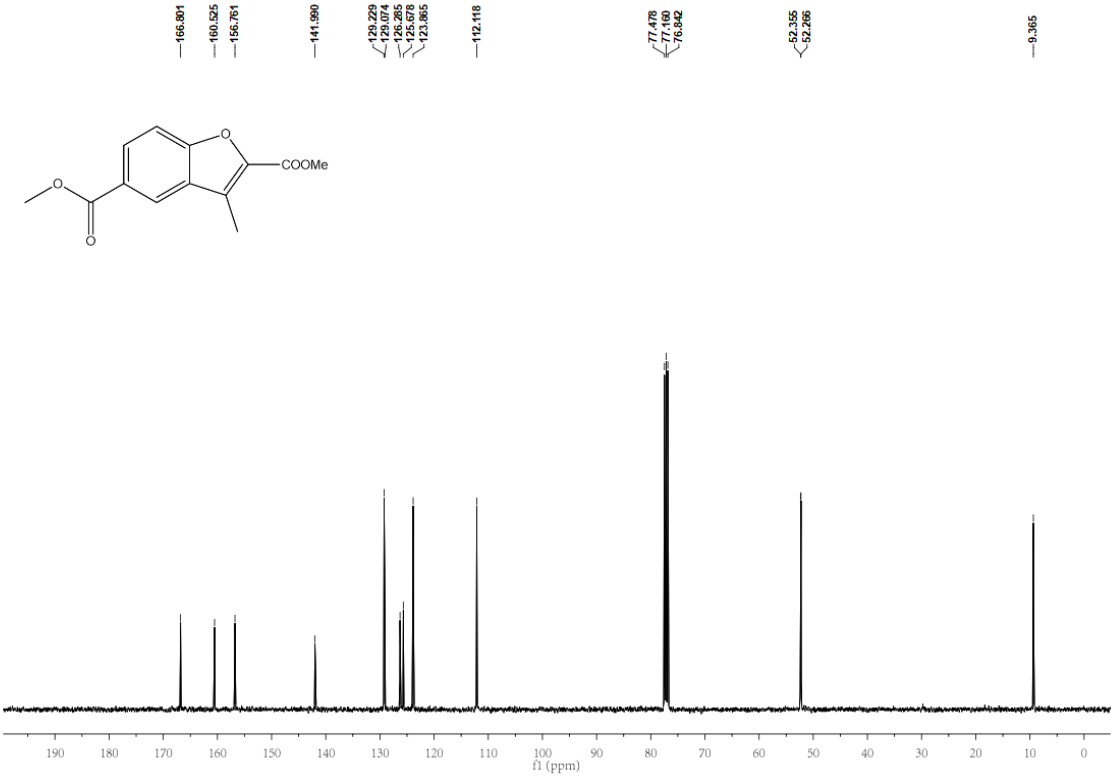

^{1}H-^{1}H NOESY of off-DNA D14 ester (400 MHz, CDCl$_3$):

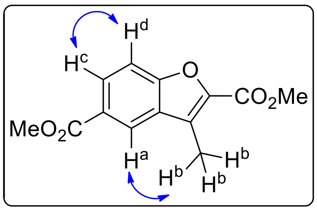

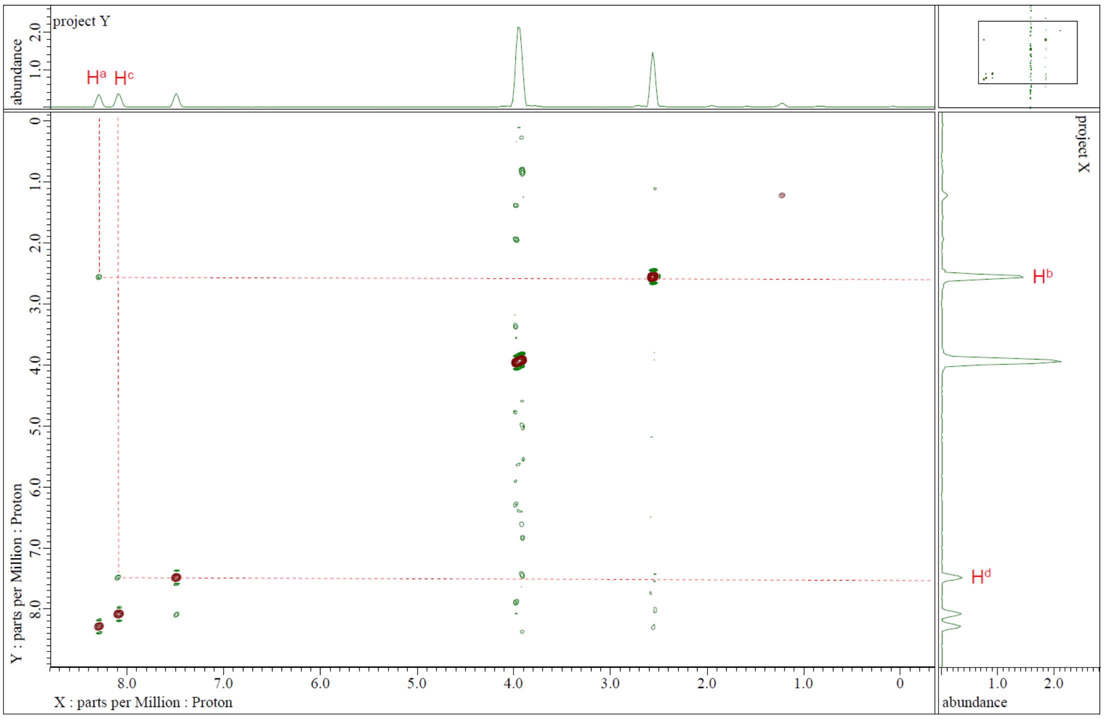

^{1}H-NMR of off-DNA H5 ester (400 MHz, DMSO-d_6)

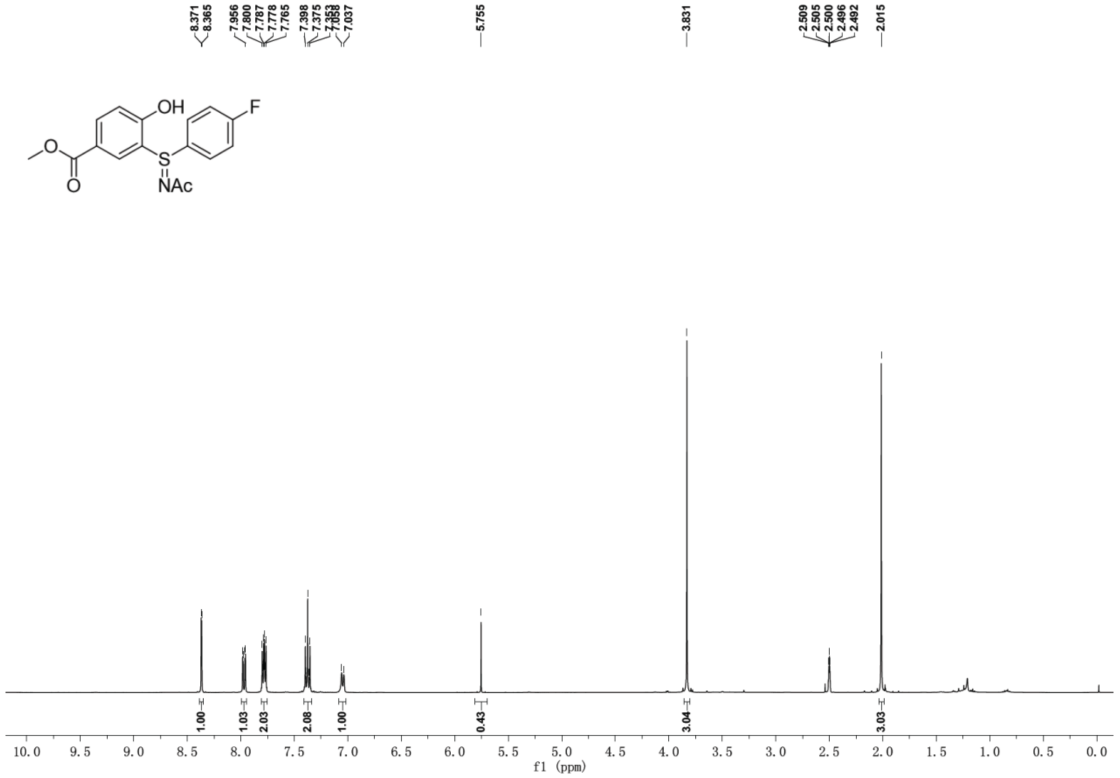

^{13}C-NMR of off-DNA H5 ester (100 MHz, DMSO-d_6)

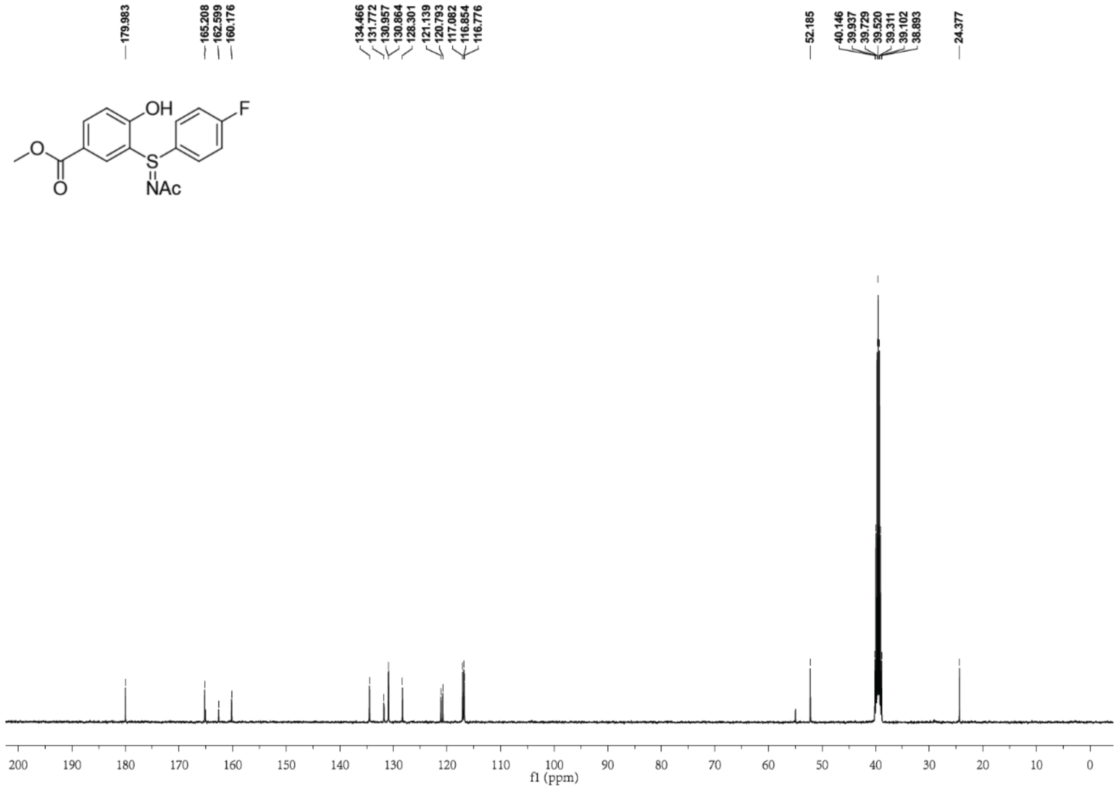

^{19}F-NMR of off-DNA H5 ester (376 MHz, DMSO-d_6)

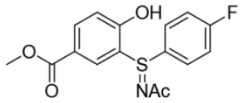

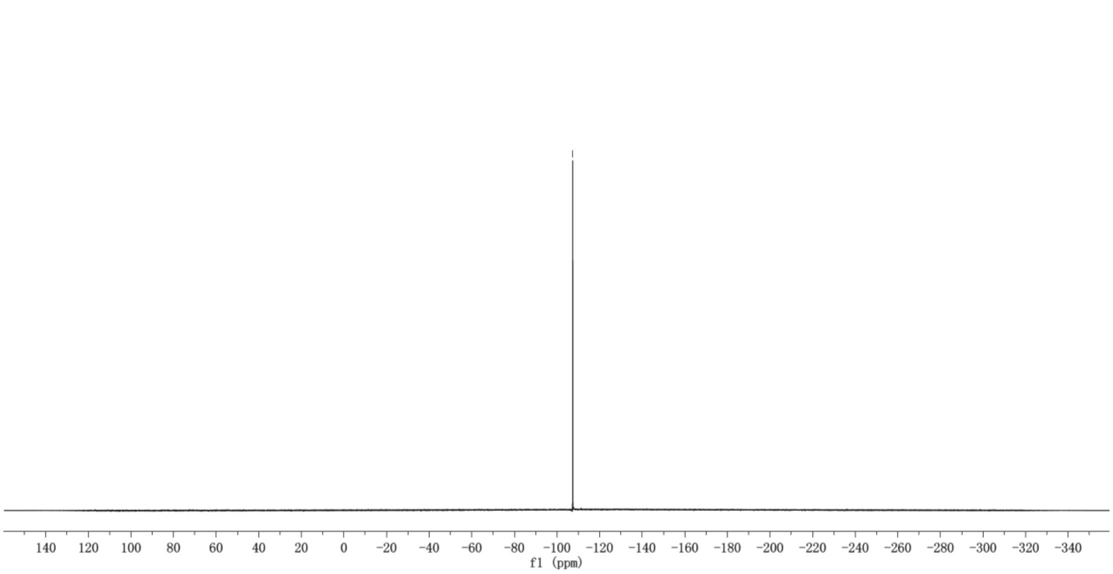

^{1}H-NMR of off-DNA H5 (400 MHz, DMSO-d_6)

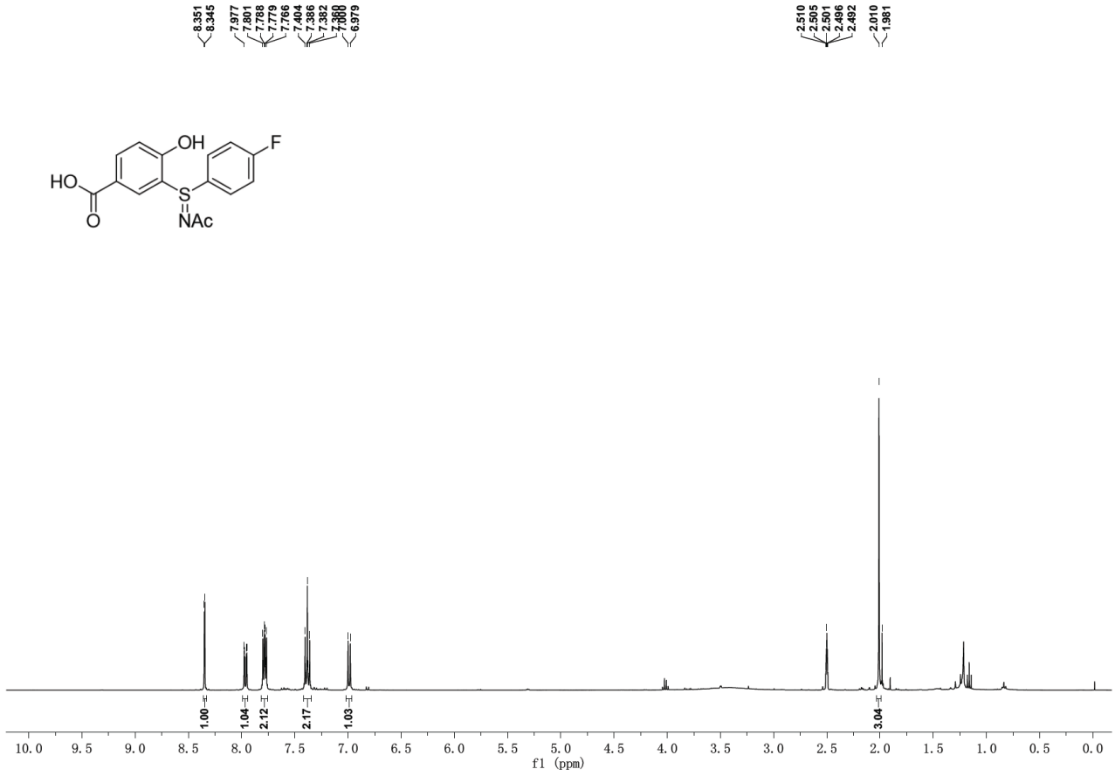

^{13}C-NMR of off-DNA H5 (100 MHz, DMSO-d_6)

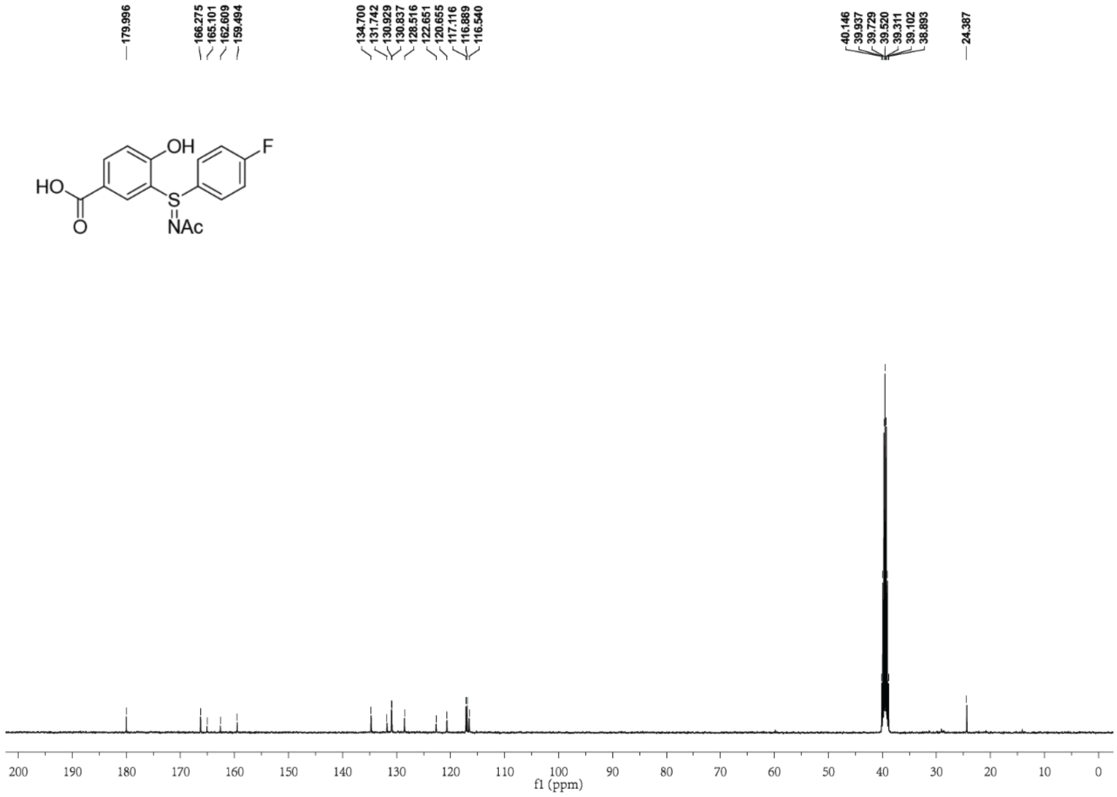

^{19}F-NMR of off-DNA H5 (376 MHz, DMSO-d_6)

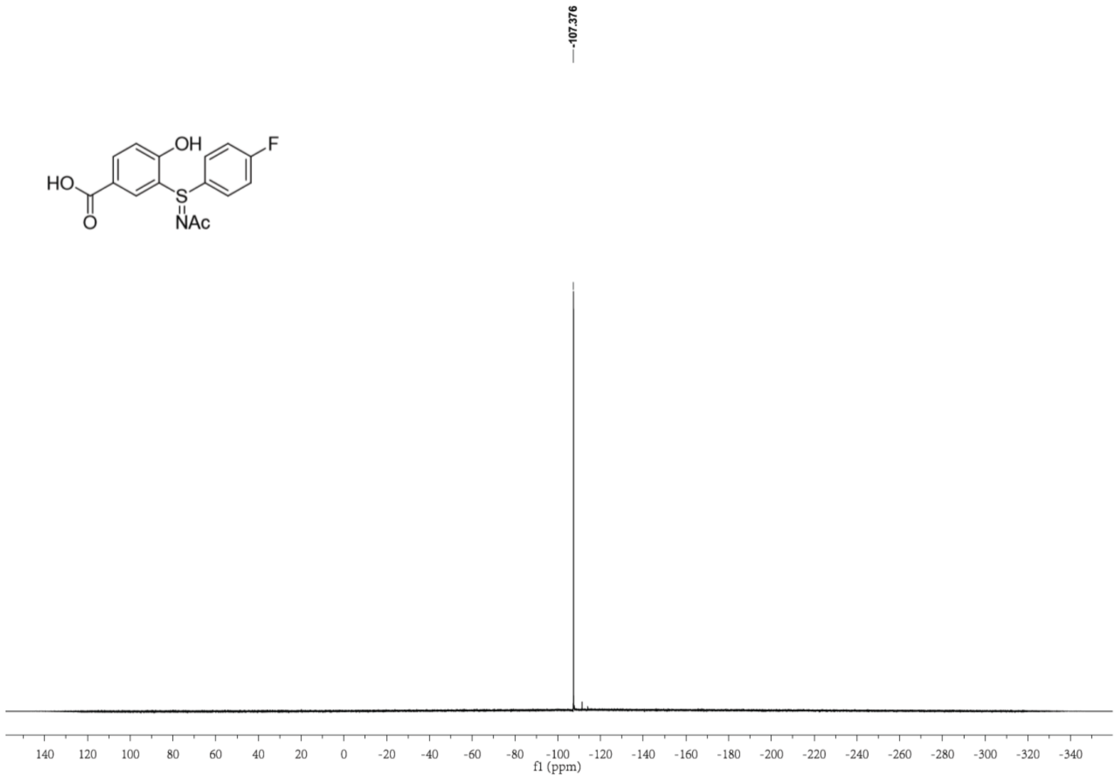

LC trace of products

LC Trace and Mass of **A1**

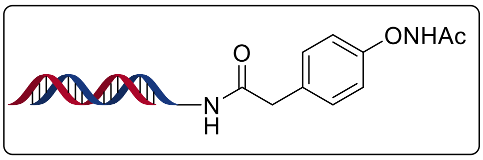

Following General Procedure **A1**
Yield: 99%
Exact mass: 5128.4310
Triply charged mass (M-3)/3, calculated 1708.4770; observed 1708.3331.

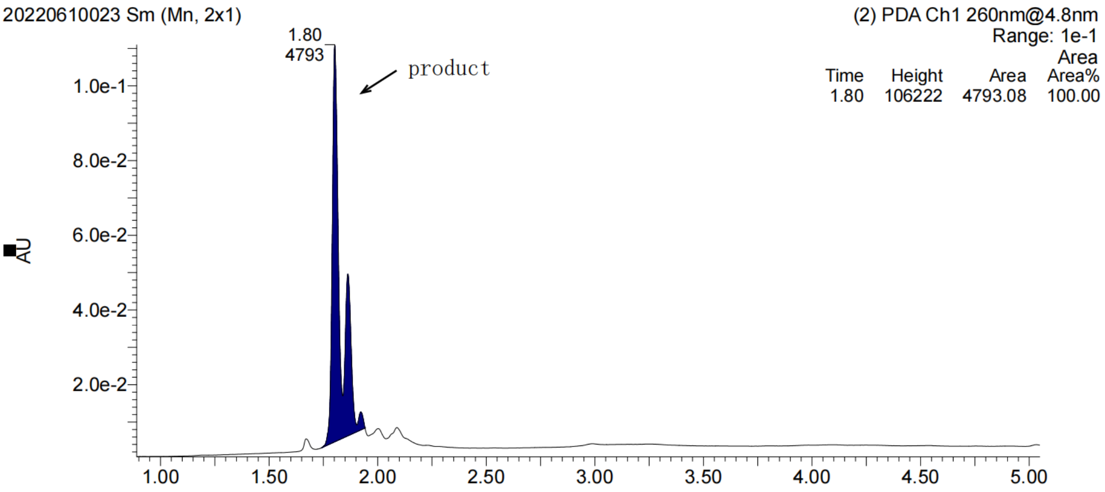

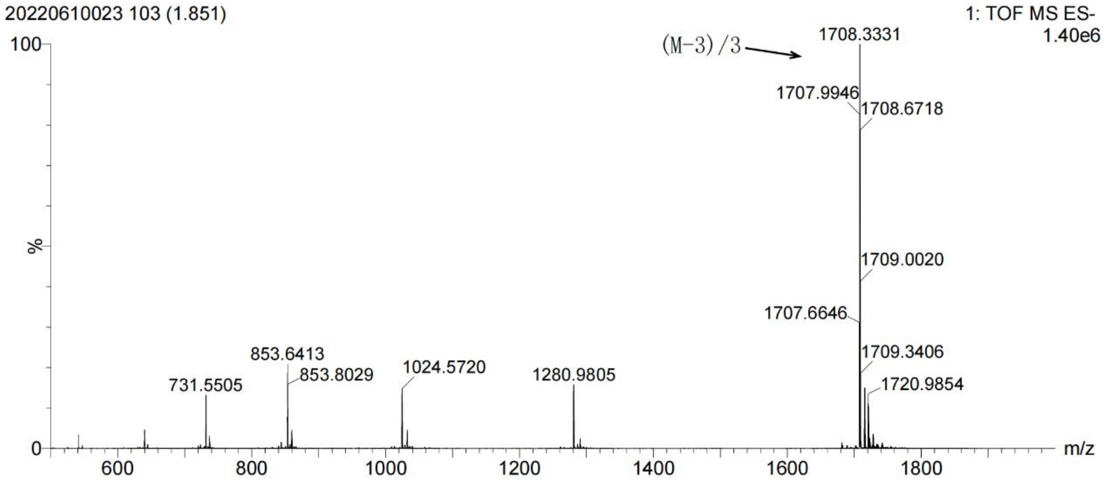

LC Trace and Mass of **A2**

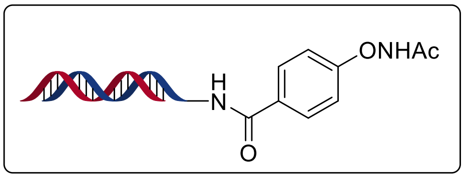

Following General Procedure **A2**
Yield: 99%
Exact mass: 5114.2833
Triply charged mass (M-3)/3, calculated 1703.7611; observed 1703.5984.

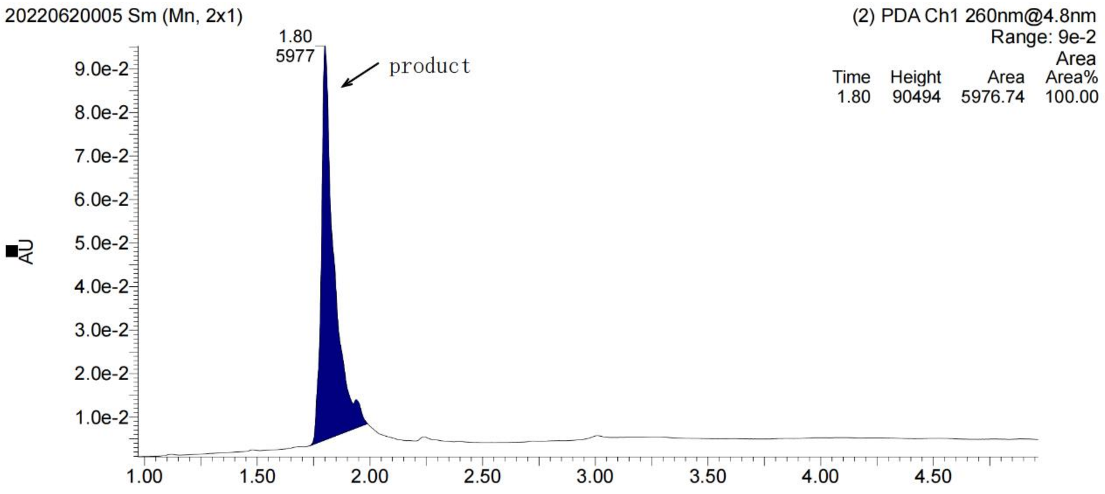

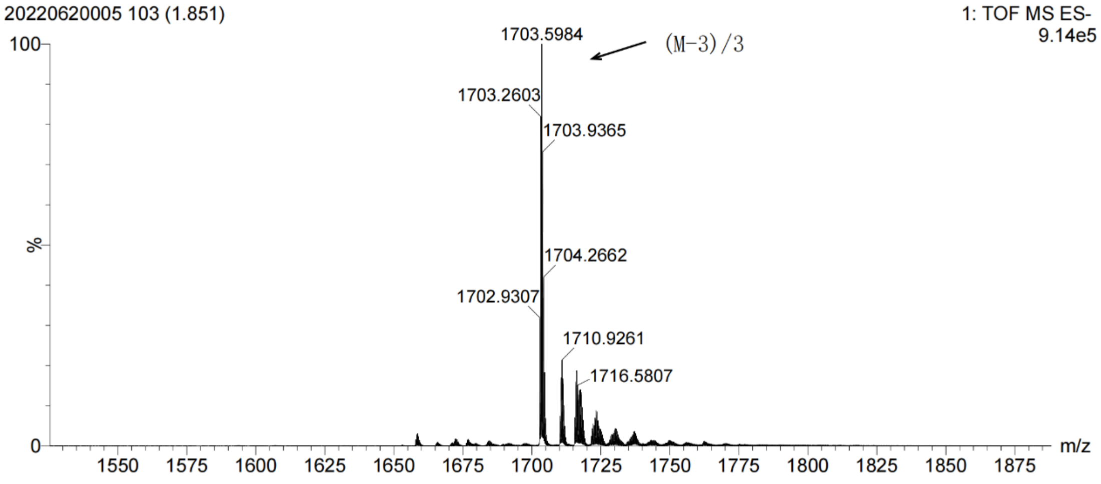

LC Trace and Mass of **A3**

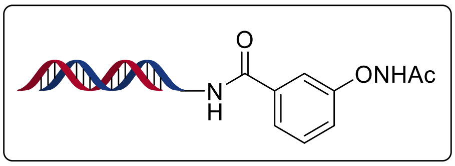

Following General Procedure **A3**

Yield: 99%

Exact mass: 5114.2833

Triply charged mass (M-3)/3, calculated 1703.7611; observed 1703.5984.

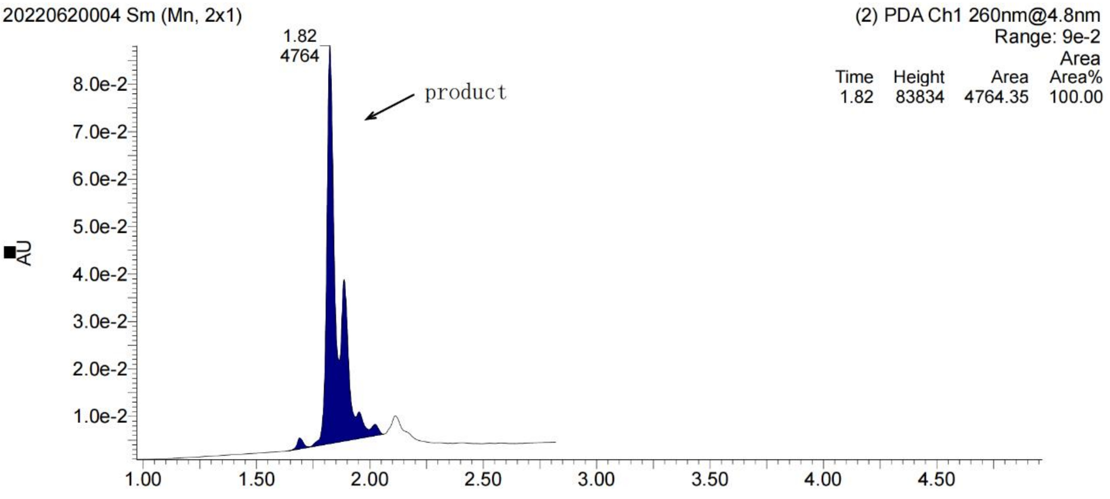

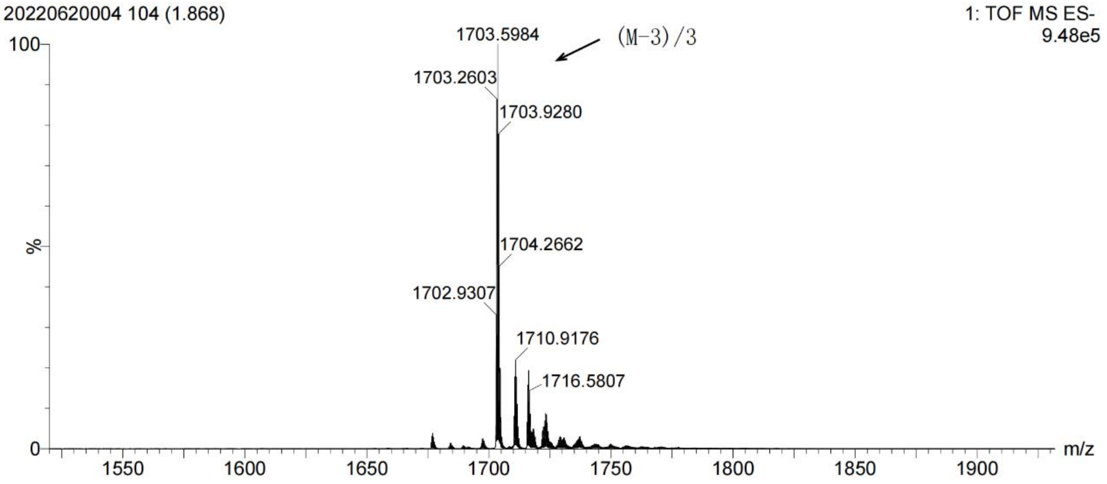

LC Trace and Mass of **A4**

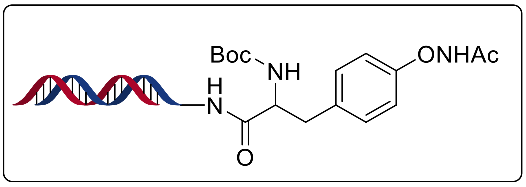

Following General Procedure **A4**

Yield: 81%

Exact mass: 5257.3779

Triply charged mass (M-3)/3, calculated 1751.4593; observed 1751.3536.

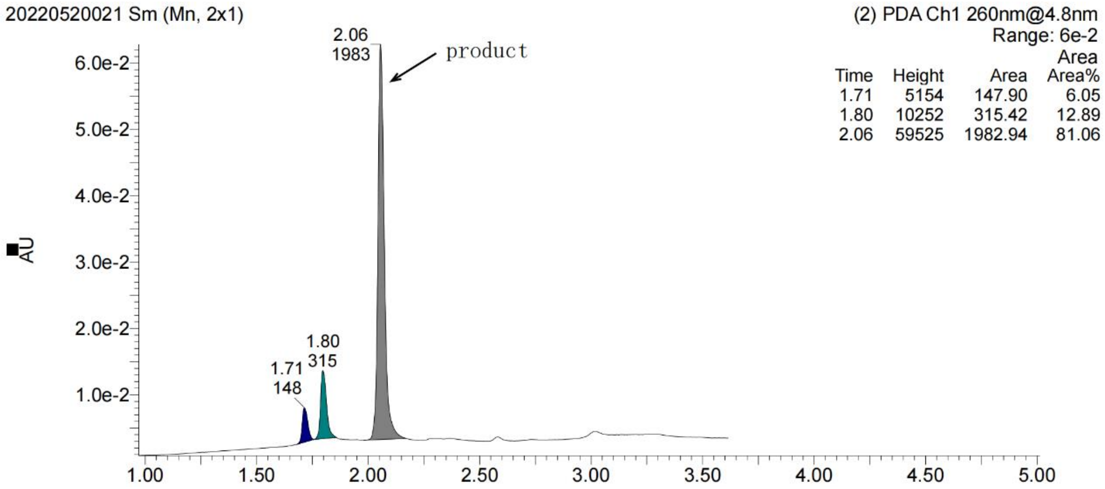

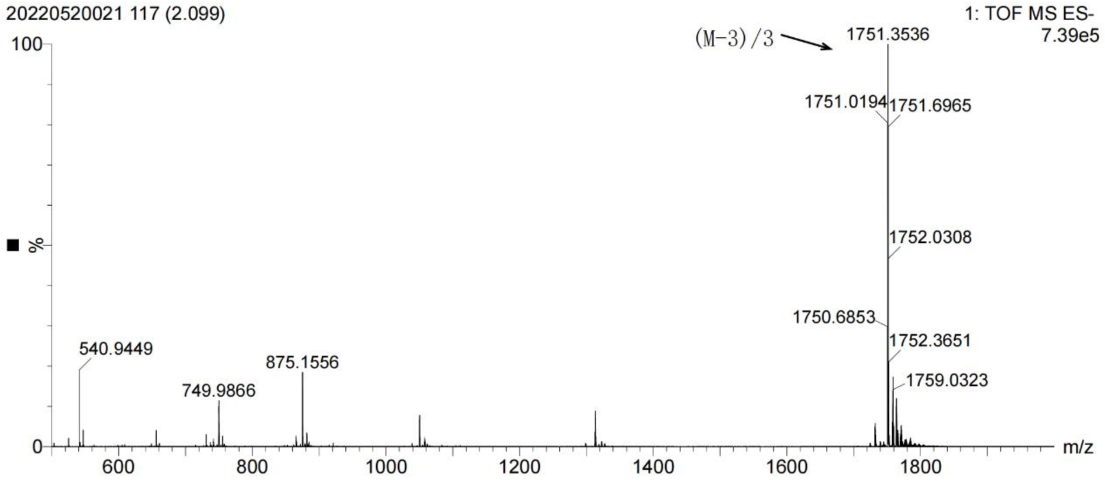

LC Trace and Mass of **B21**

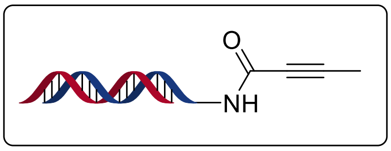

Following General Procedure **B21**
Yield: 94%
Exact mass: 5003.2512
Triply charged mass (M-3)/3, calculated 1666.7504; observed 1666.6552.

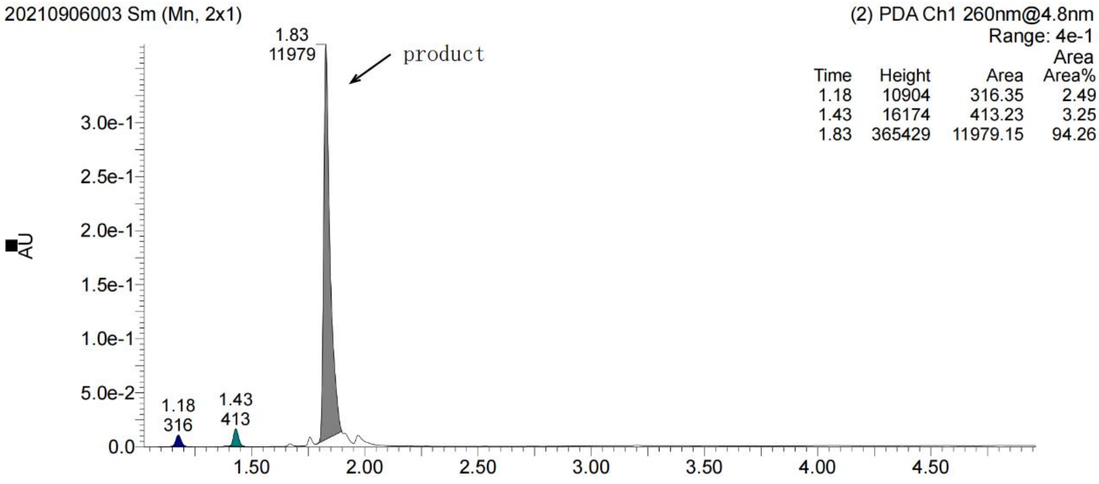

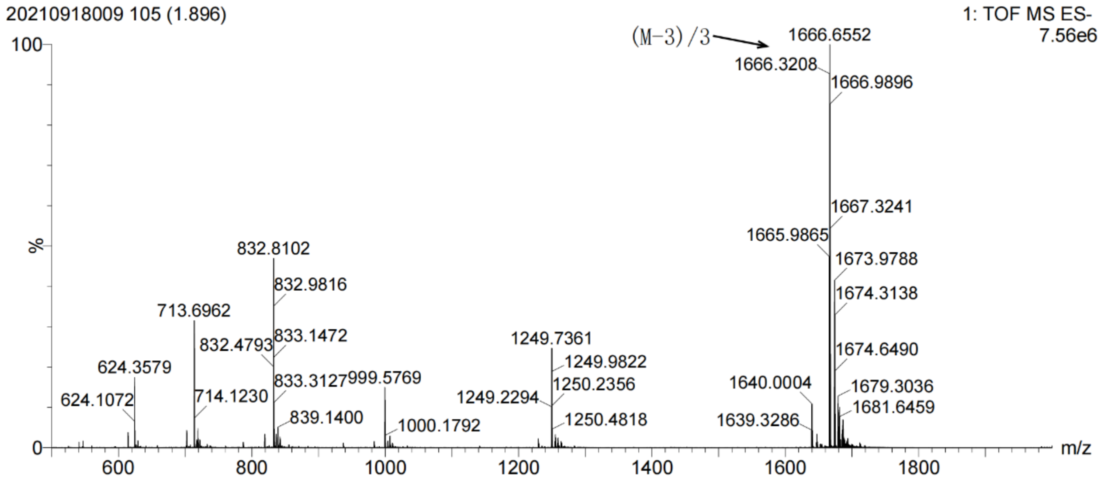

LC Trace and Mass of **D13-1**

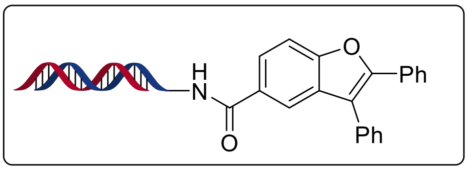

Following General Procedure **D13-1**
Yield: 92%
Exact mass: 5233.3242
Triply charged mass (M-3)/3, calculated 1743.4414; observed 1743.3530.

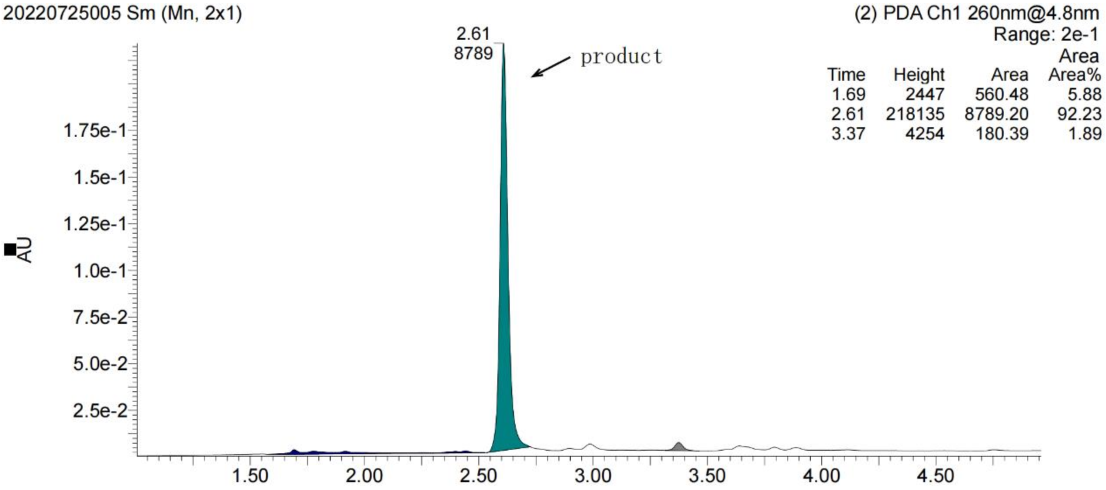

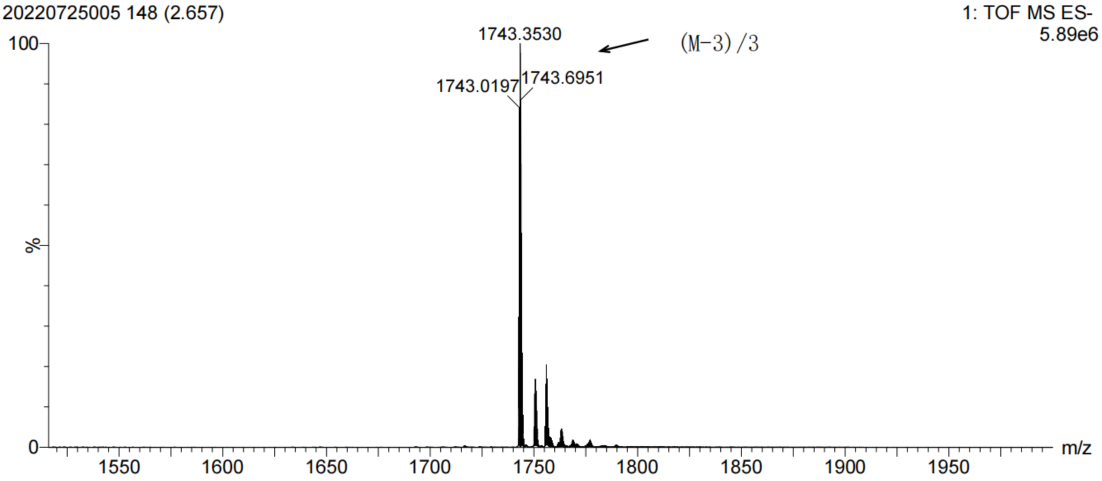

LC Trace and Mass of **H5-1**

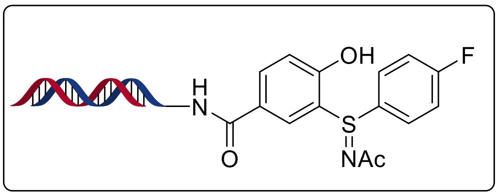

Following General Procedure **H5-1**

Yield: 72%

Exact mass: 5240.2770

Triply charged mass (M-3)/3, calculated 1745.7590; observed 1745.6710.

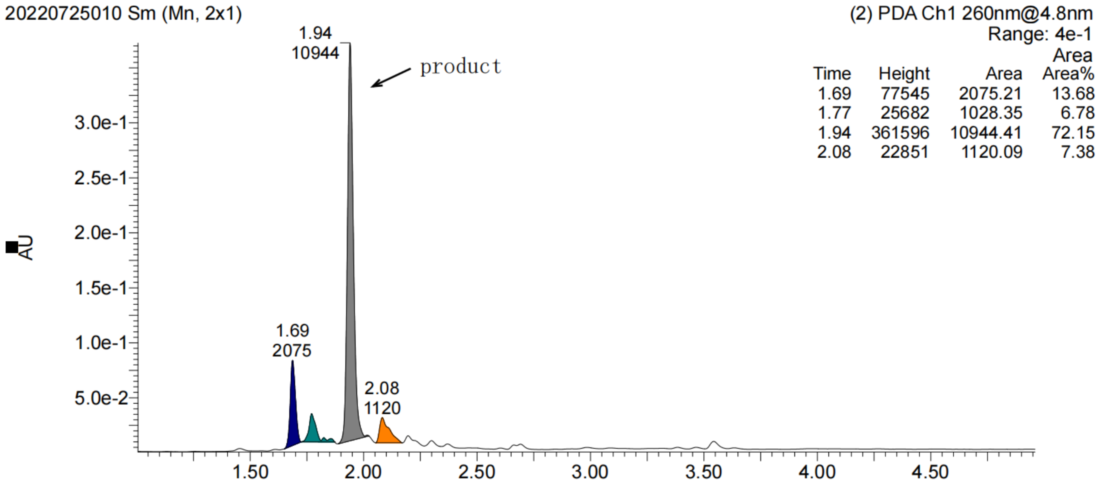

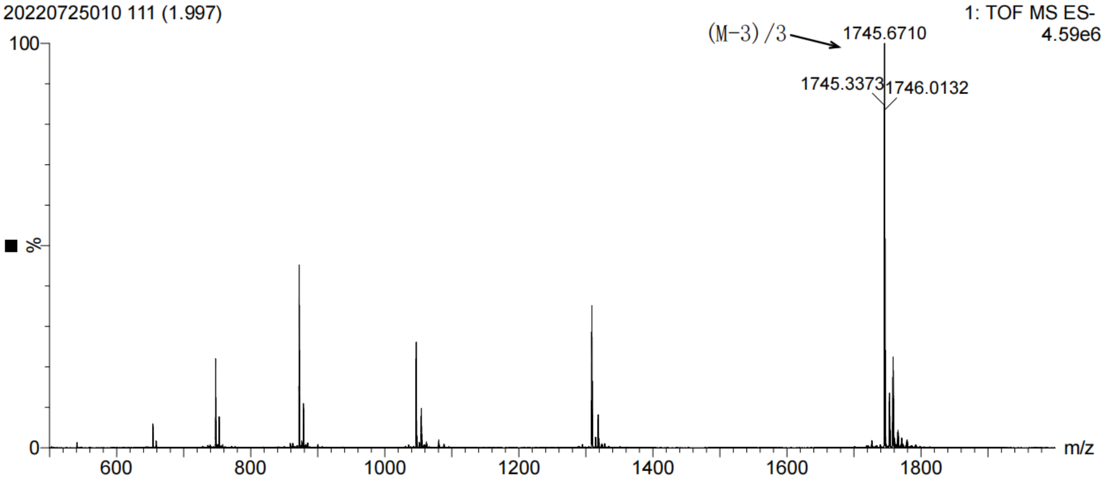

LC Trace and Mass of **C1**

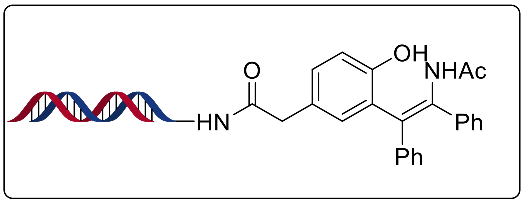

Following General Procedure **C1**

Yield: 81%

Exact mass: 5306.3664

Triply charged mass (M-3)/3, calculated 1767.7888; observed 1767.6722.

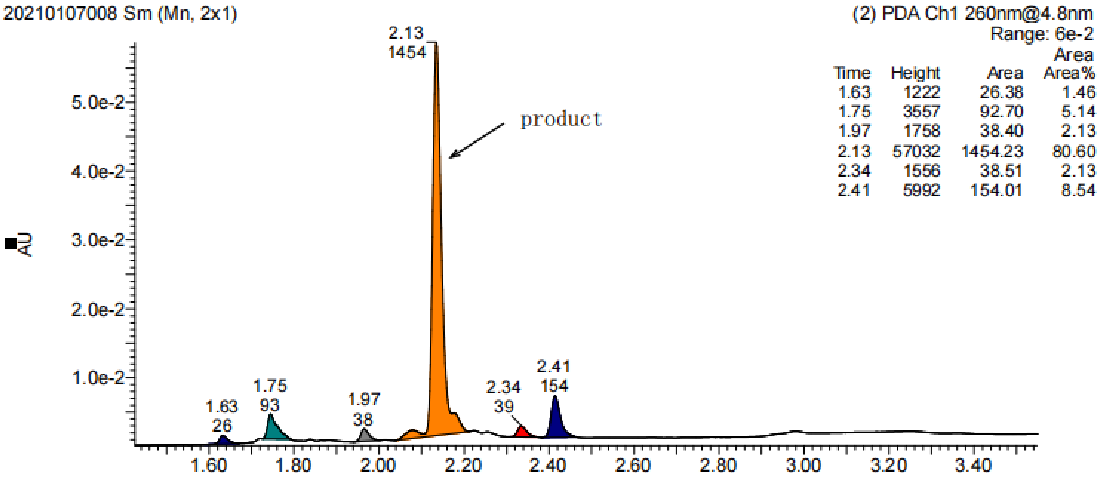

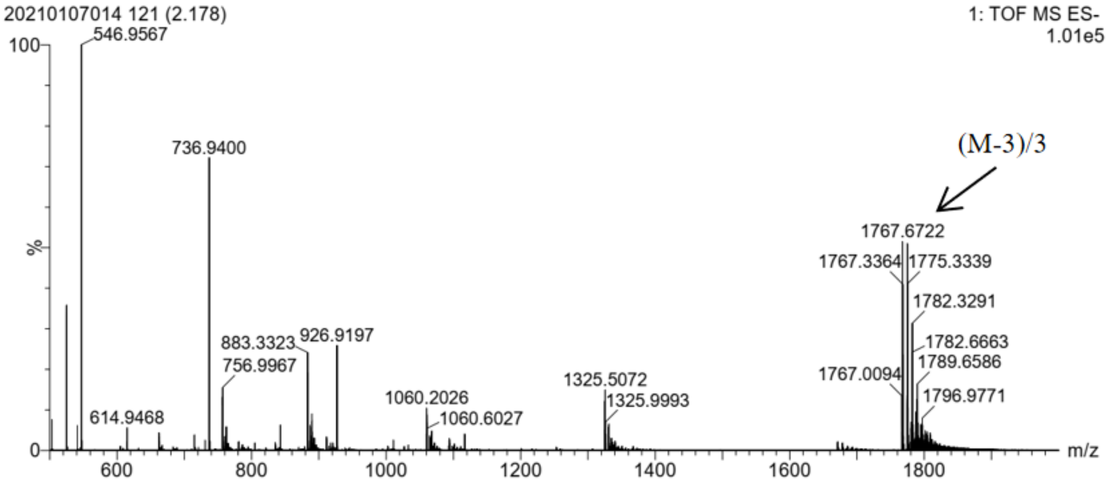

LC Trace and Mass of **C2**

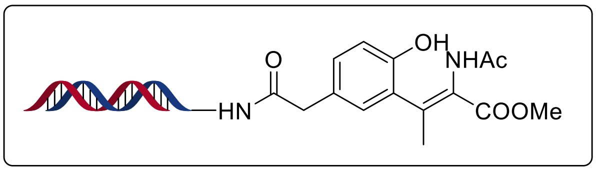

Following General Procedure **C2**

Yield: 60%

Exact mass: 5226.3249

Triply charged mass (M-3)/3, calculated 1741.1083; observed 1741.0096.

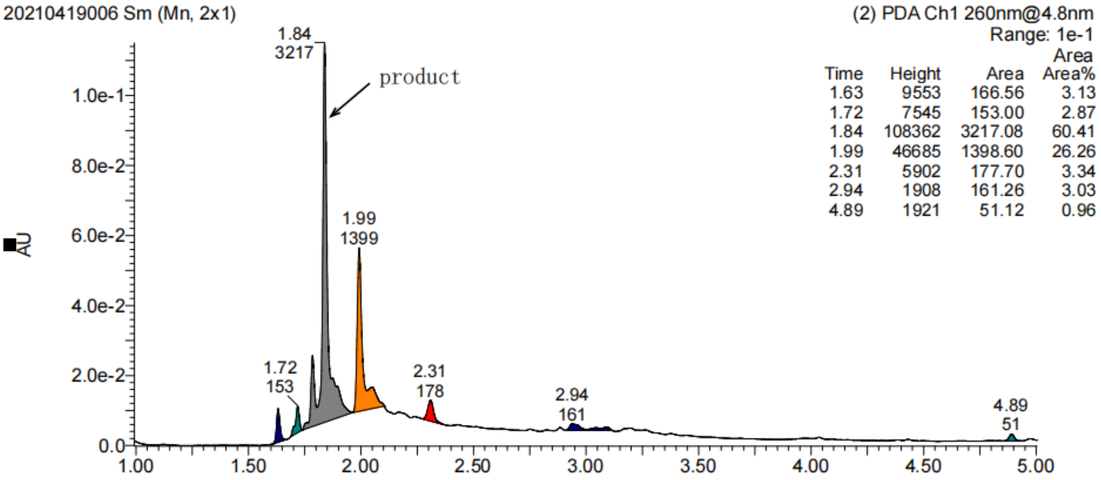

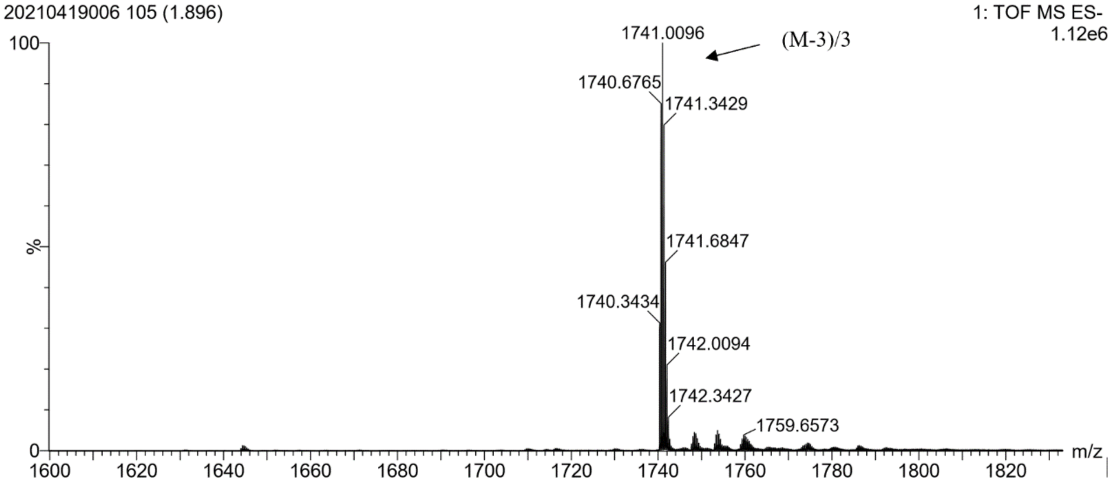

LC Trace and Mass of **C3**

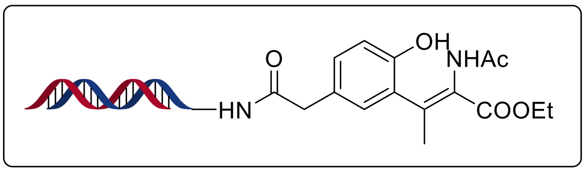

Following General Procedure **C3**
Yield: 60%
Exact mass: 5240.3406
Triply charged mass (M-3)/3, calculated 1745.7802; observed 1745.6858.

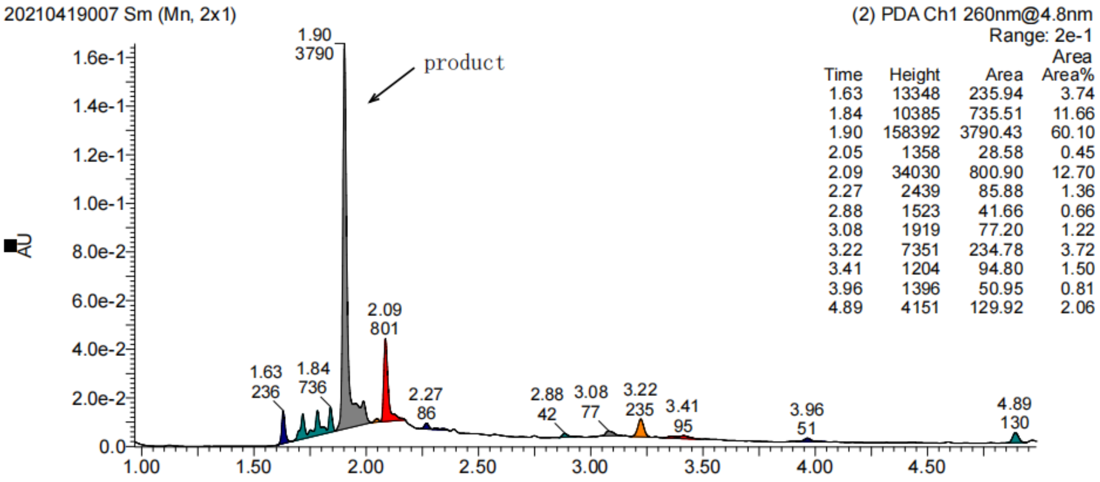

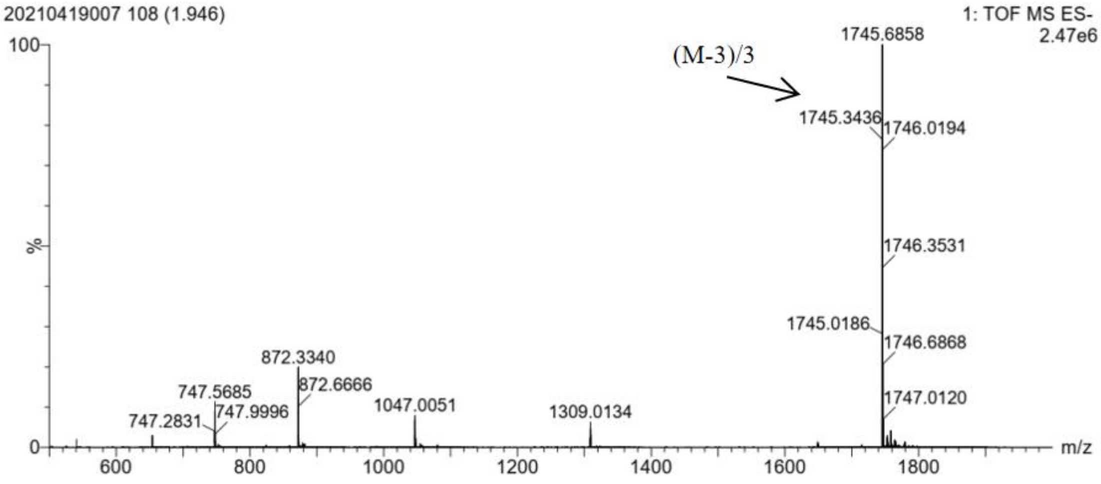

LC Trace and Mass of **C4**

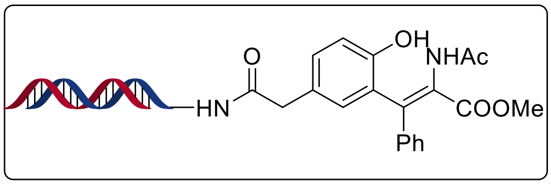

Following General Procedure **C4**
Yield: 50%
Exact mass: 5288.3406
Triply charged mass (M-3)/3, calculated 1761.7802; observed 1761.6848.

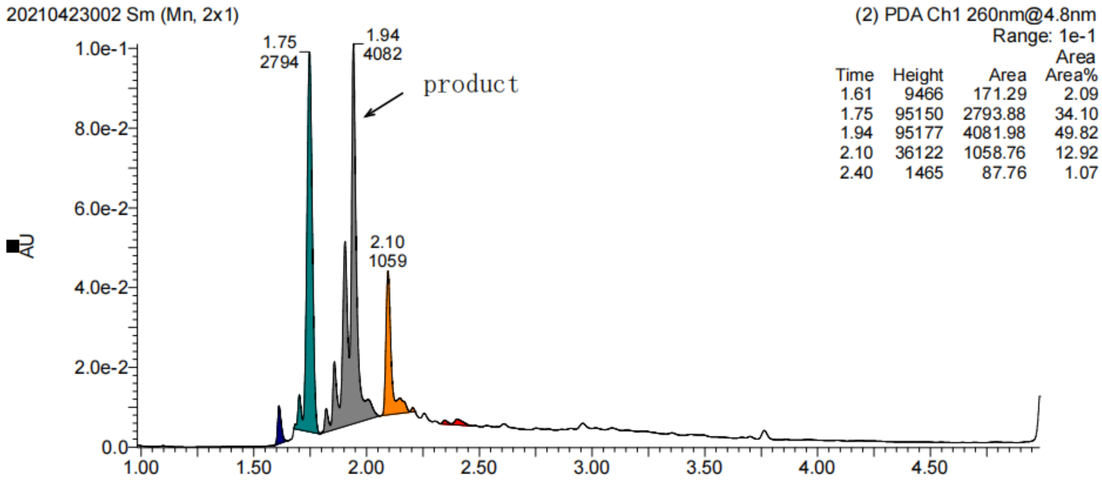

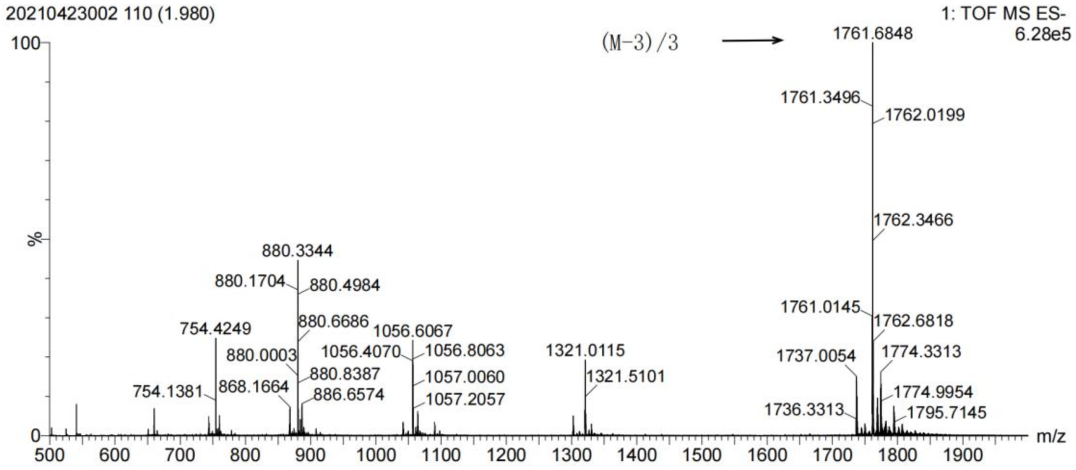

LC Trace and Mass of **C5**

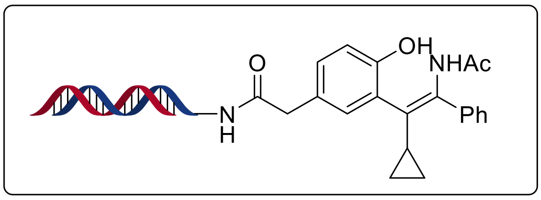

Following General Procedure **C5**
Yield: 71%
Exact mass: 5270.3799
Triply charged mass (M-3)/3, calculated 1755.7933; observed 1755.6934.

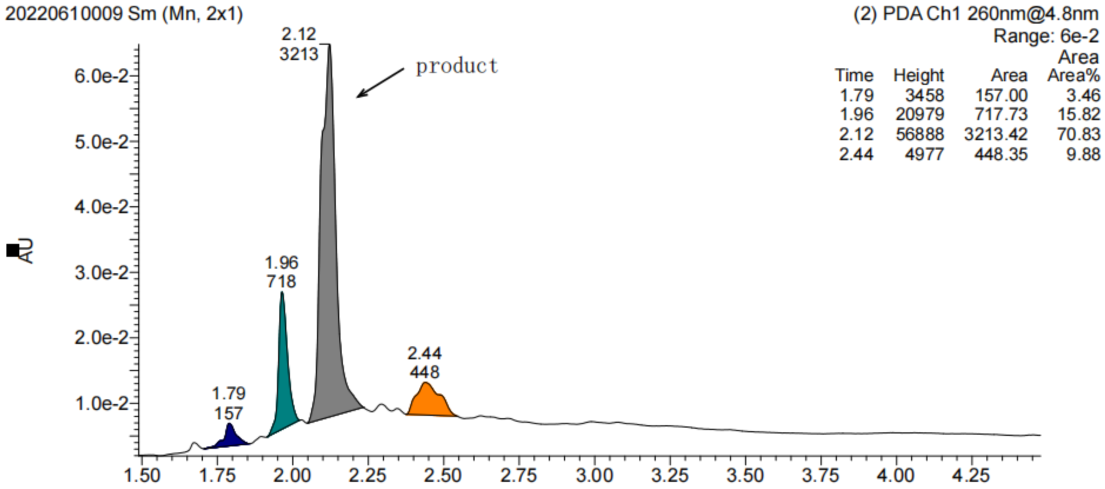

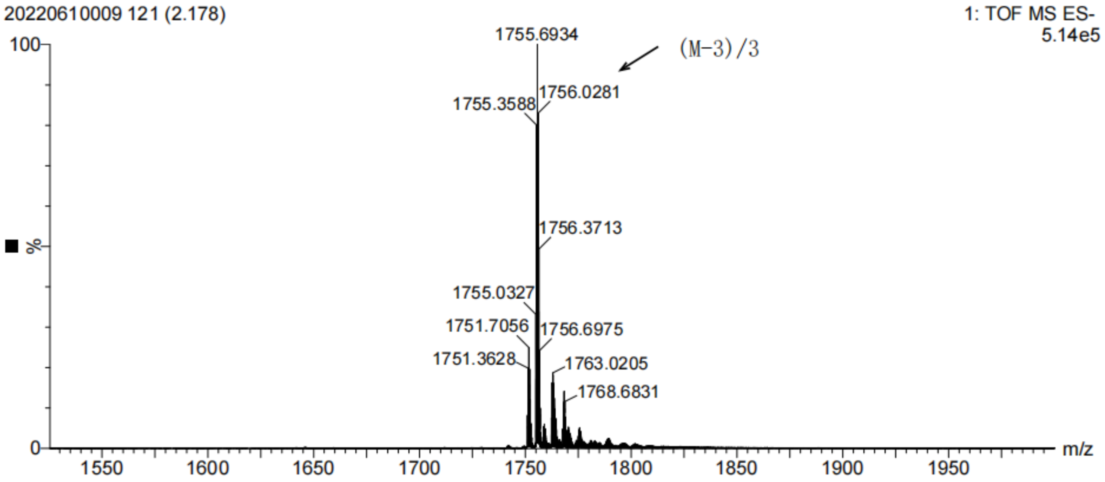

LC Trace and Mass of **C6**

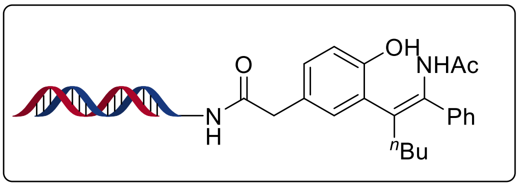

Following General Procedure **C6**
Yield: 45%
Exact mass: 5286.4098
Triply charged mass (M-3)/3, calculated 1761.1366; observed 1761.0504.

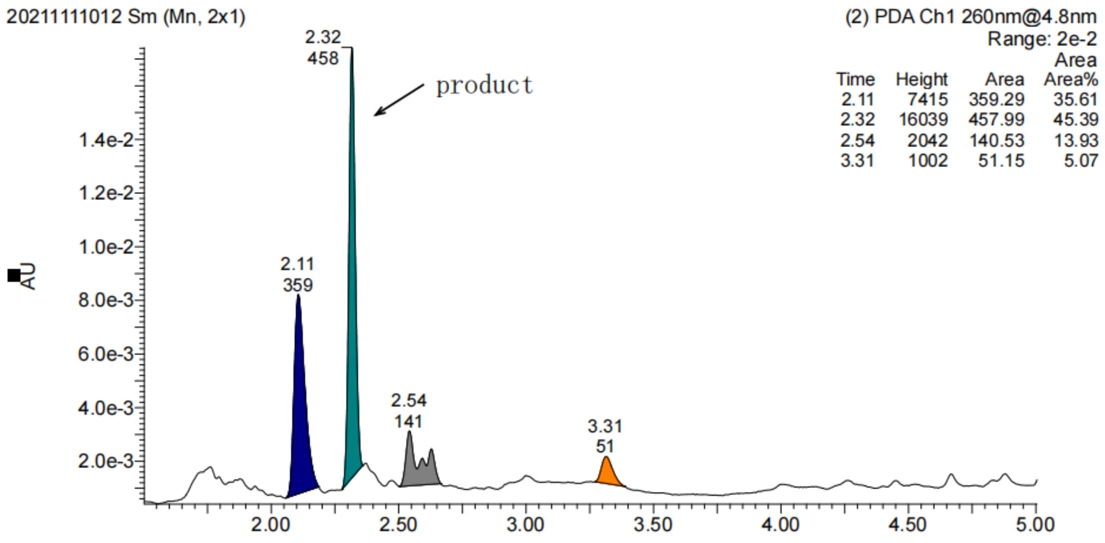

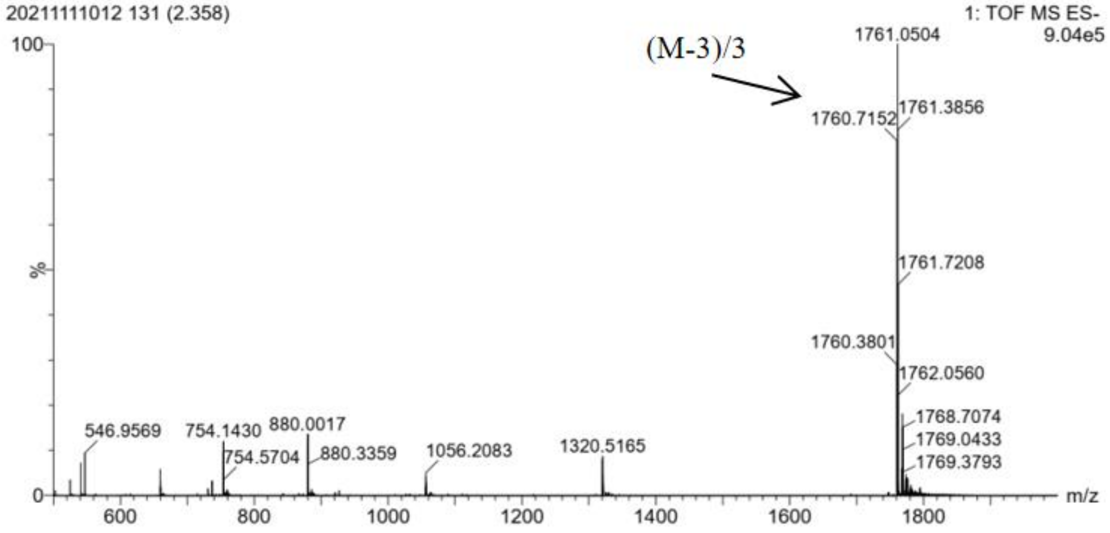

LC Trace and Mass of **C7**

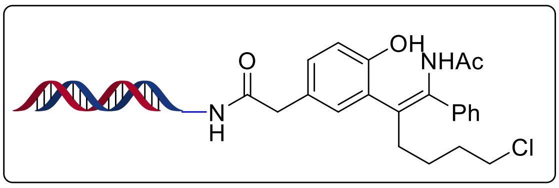

Following General Procedure **C7**
Yield: 57%
Exact mass: 5321.1171
Triply charged mass (M-3)/3, calculated 1772.7057; observed 1772.7233.

Time	Height	Area	Area%
1.80	27016	2087.45	19.20
2.17	7549	409.61	3.77
2.32	156560	6160.51	56.66
2.46	5135	135.93	1.25
2.60	17234	1086.79	10.00
2.69	21316	866.60	7.97
3.32	2561	126.24	1.16

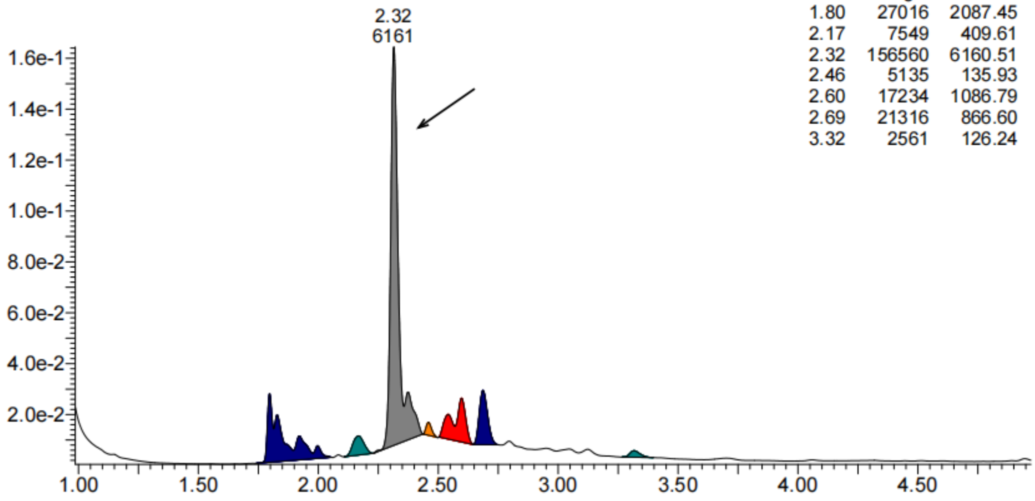

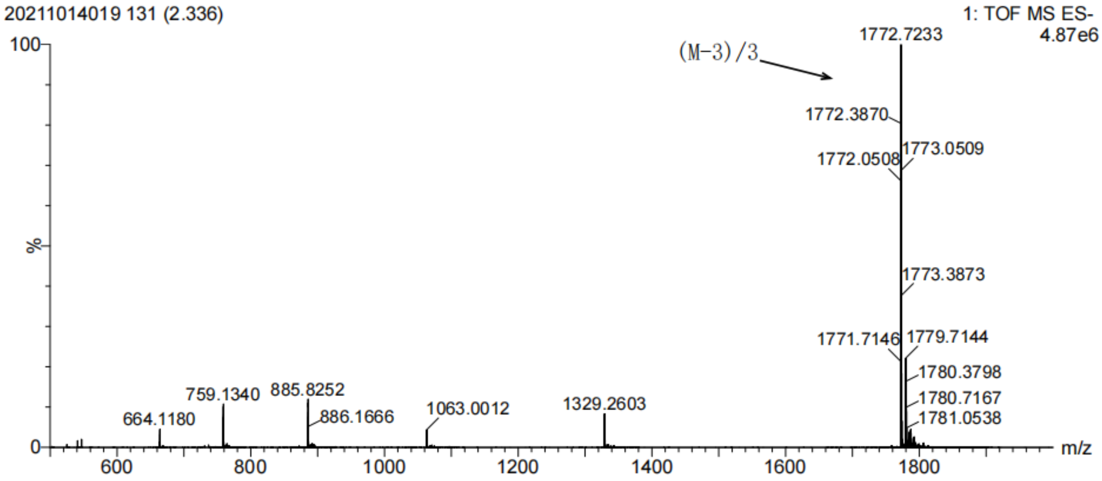

LC Trace and Mass of **C8**

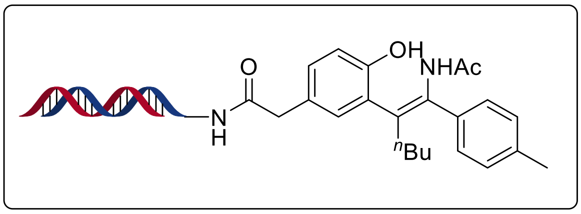

Following General Procedure **C8**
Yield: 80%
Exact mass: 5300.4135
Triply charged mass (M-3)/3, calculated 1765.8045; observed 1765.7089.

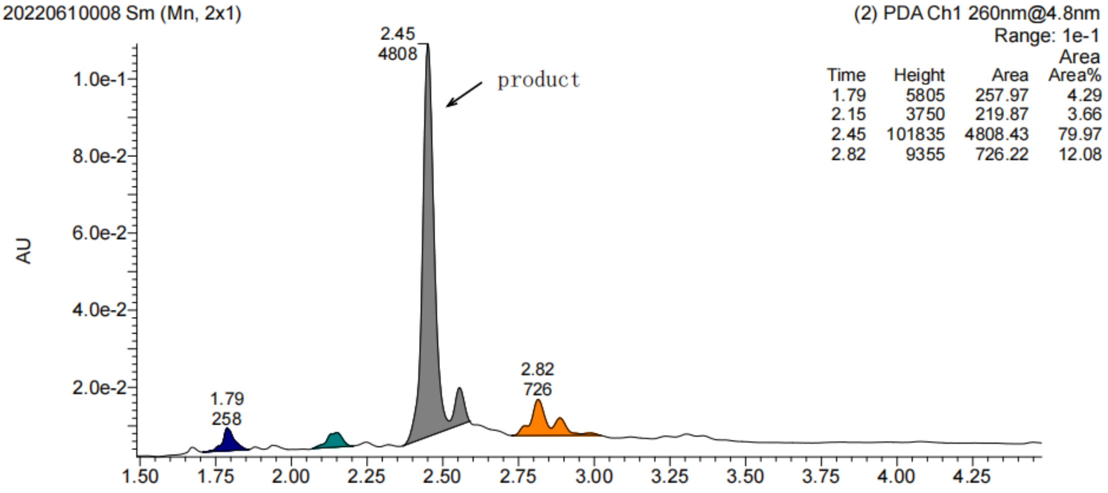

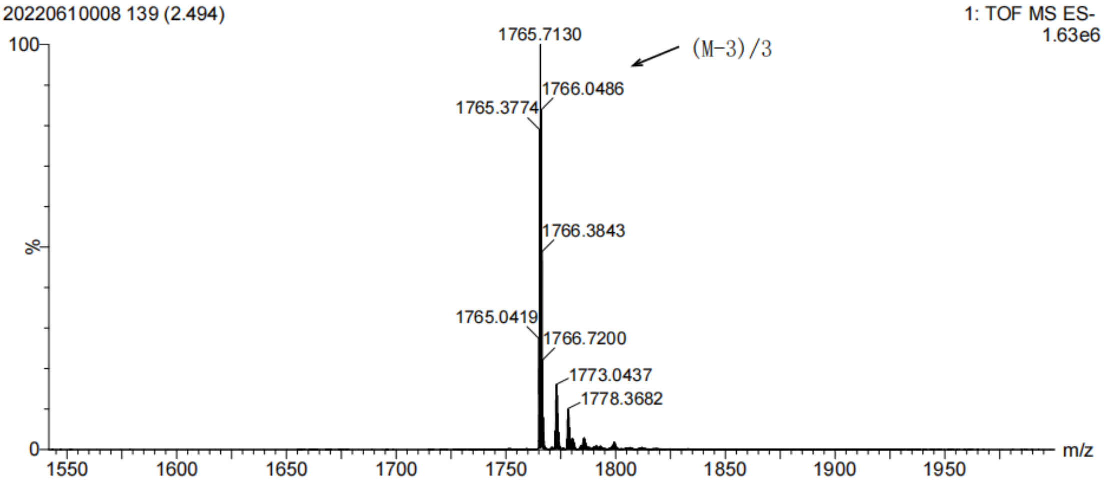

LC Trace and Mass of **C9**

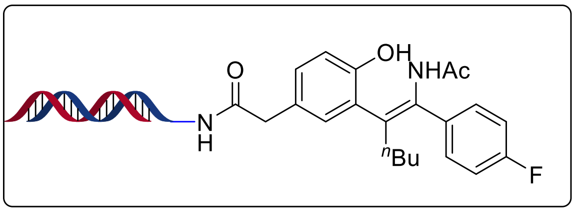

Following General Procedure **C9**
Yield: 75%
Exact mass: 5304.3999
Triply charged mass (M-3)/3, calculated 1767.1333; observed 1767.0385.

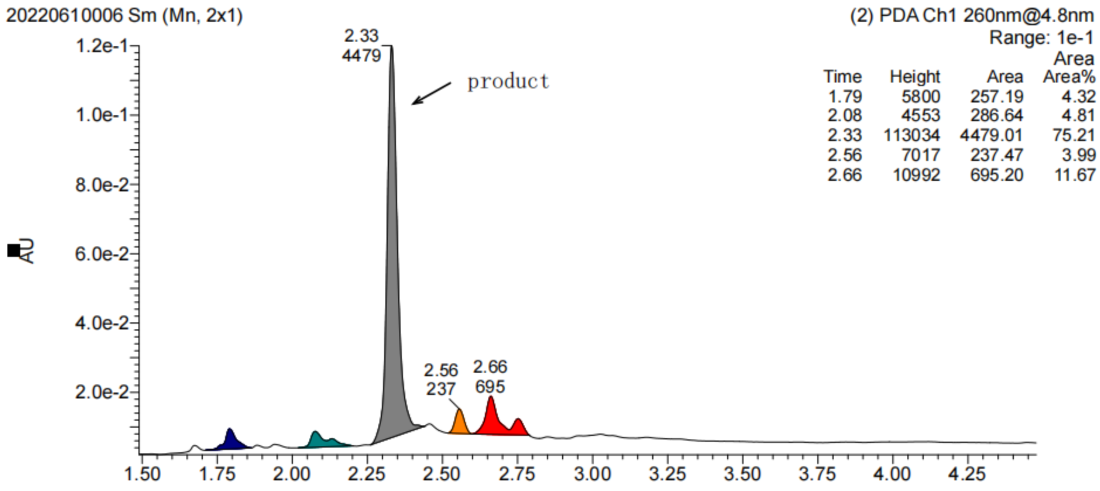

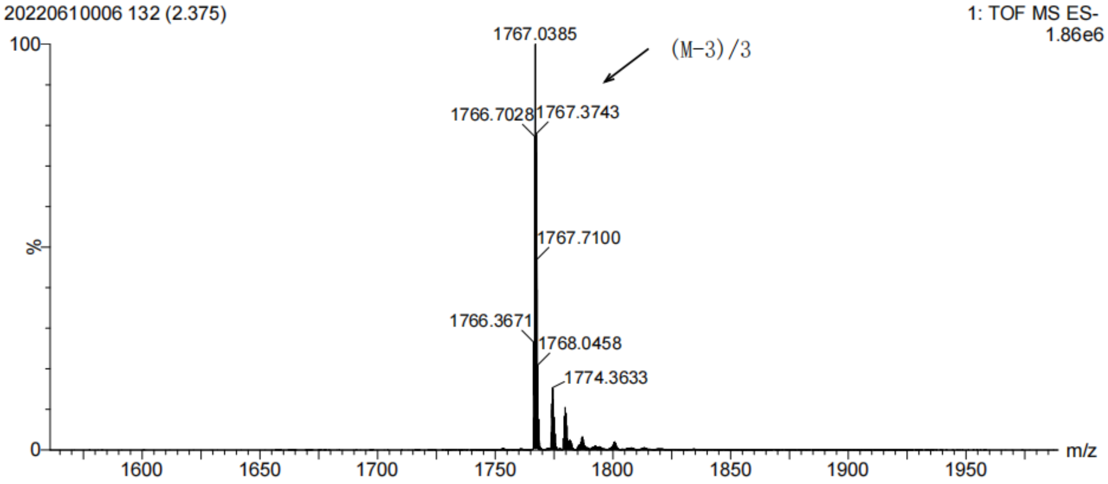

LC Trace and Mass of **C10**

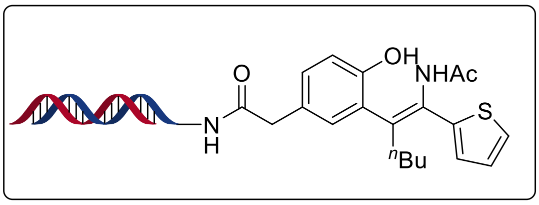

Following General Procedure **C10**

Yield: 81%

Exact mass: 5292.3543

Triply charged mass (M-3)/3, calculated 1763.1181; observed 1763.0205.

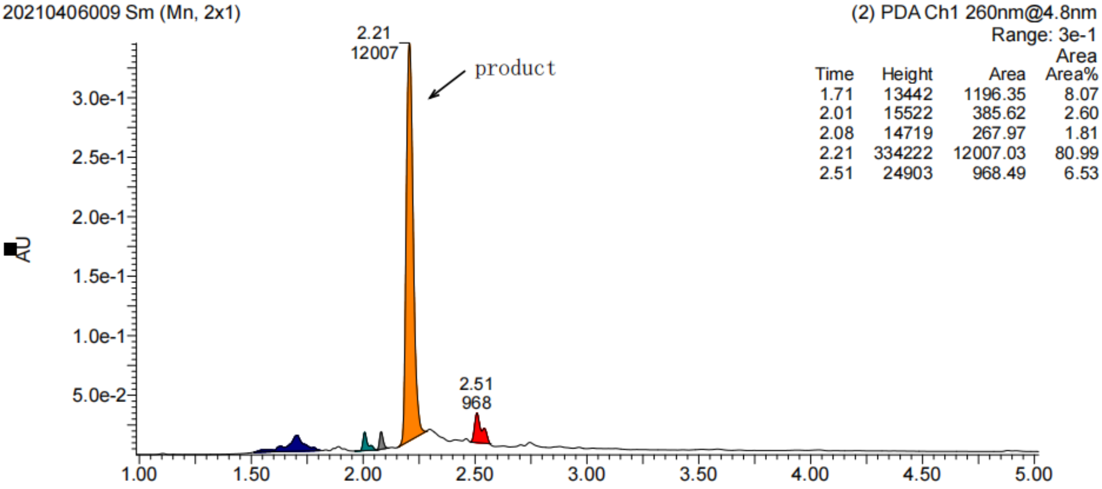

Time	Height	Area	Area Area%
1.71	13442	1196.35	8.07
2.01	15522	385.62	2.60
2.08	14719	267.97	1.81
2.21	334222	12007.03	80.99
2.51	24903	968.49	6.53

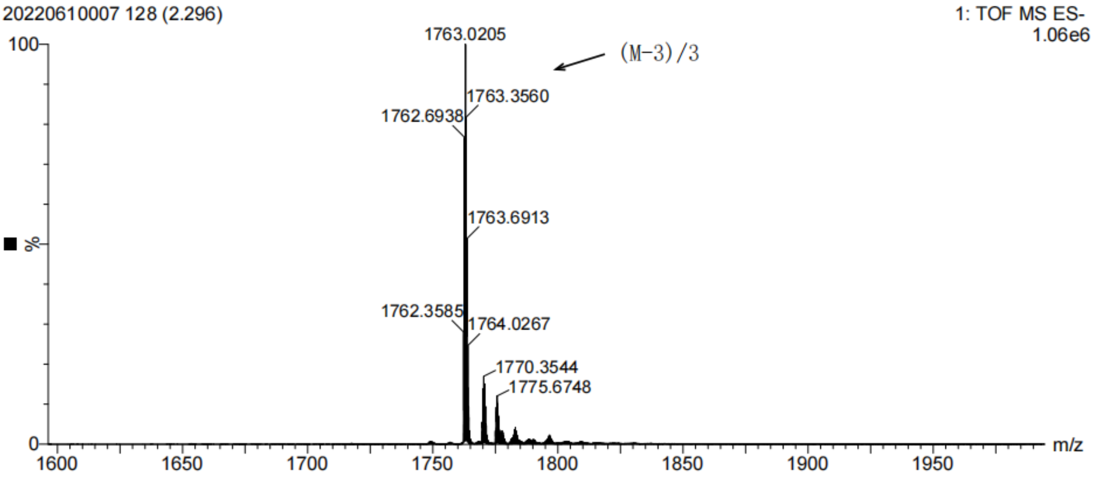

LC Trace and Mass of **C11**

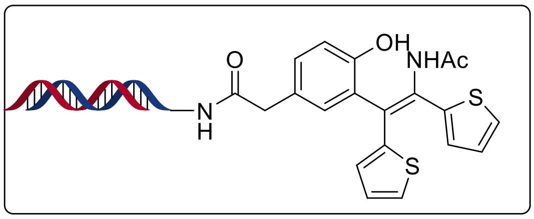

Following General Procedure **C11**
Yield: 63%
Exact mass: 5318.2794
Triply charged mass (M-3)/3, calculated 1771.7598; observed 1771.6587.

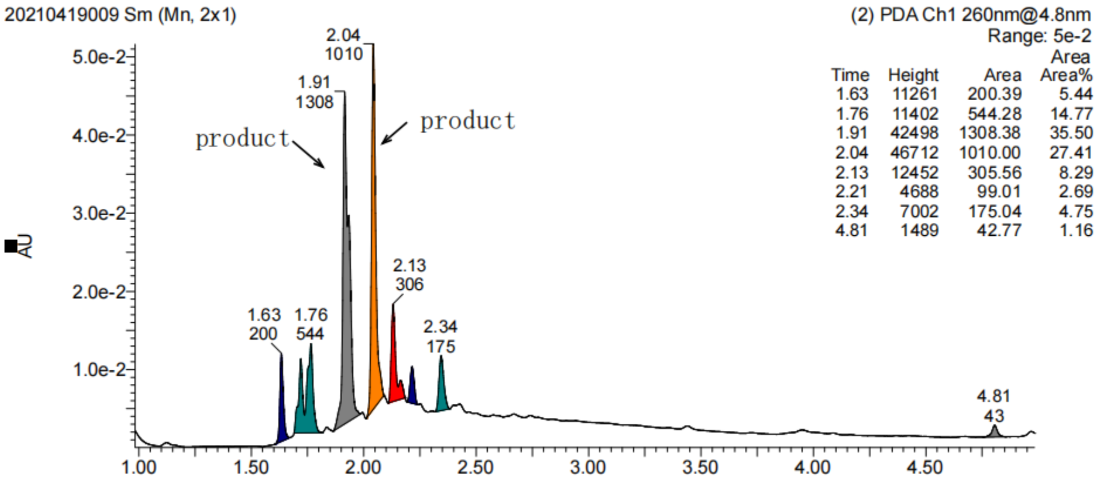

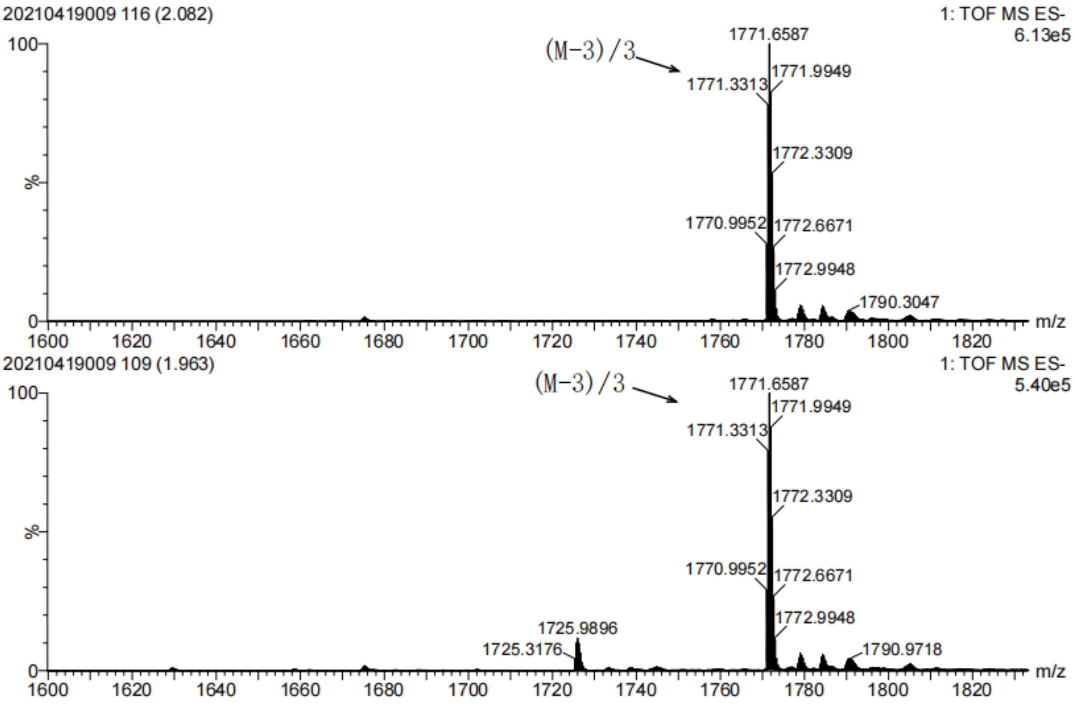

LC Trace and Mass of **C12**

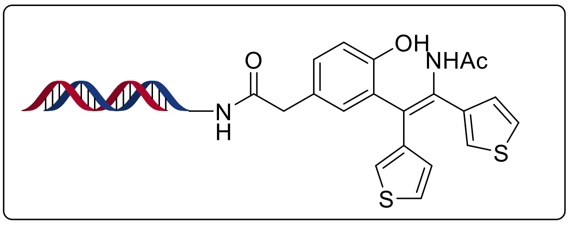

Following General Procedure **C12**
Yield: 36%
Exact mass: 5318.2794
Triply charged mass (M-3)/3, calculated 1771.7598; observed 1771.6887.

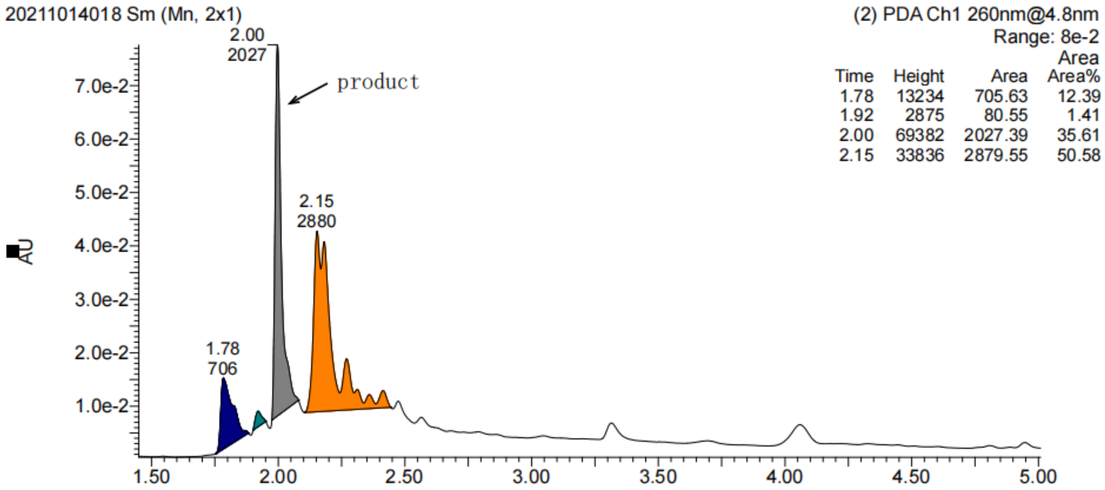

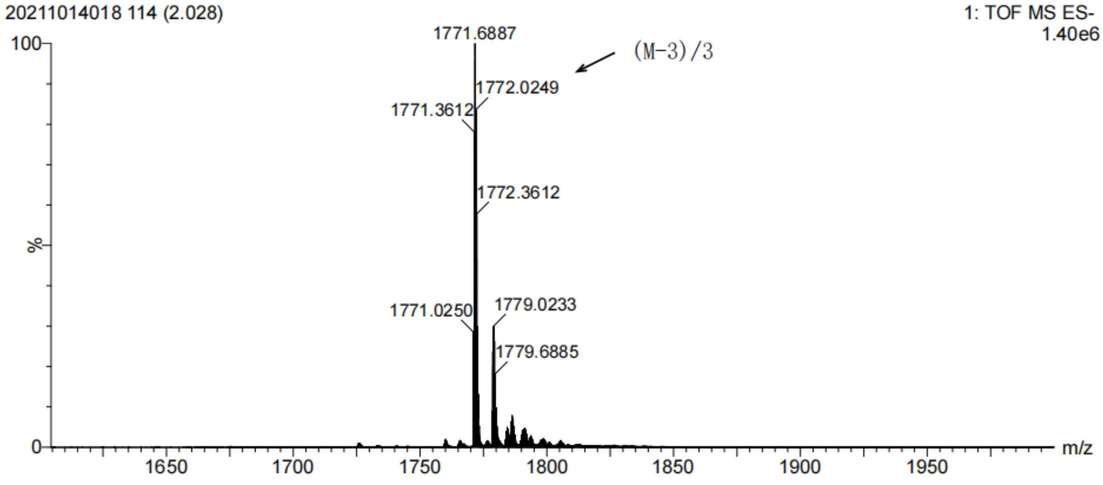

LC Trace and Mass of **C13**

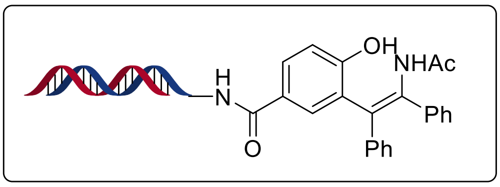

Following General Procedure **C13**
Yield: 82%
Exact mass: 5292.3507
Triply charged mass (M-3)/3, calculated 1763.1169; observed 1763.0178.

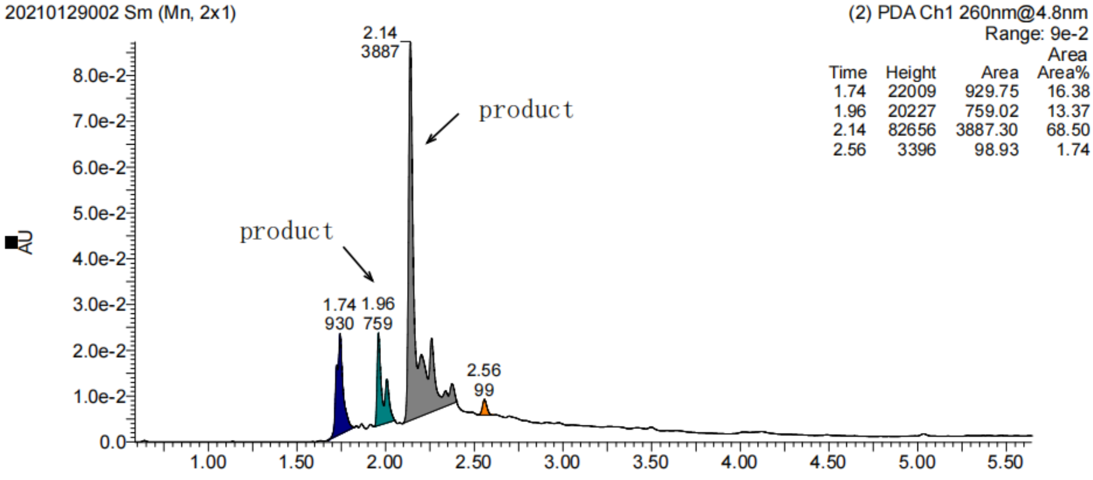

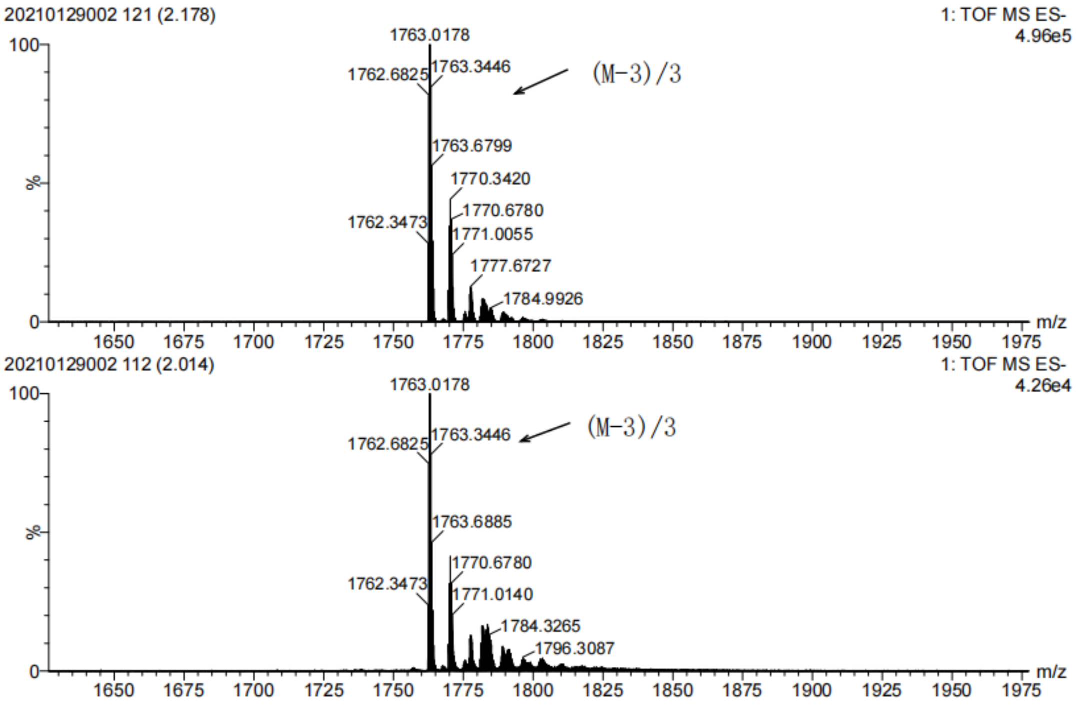

LC Trace and Mass of **C14**

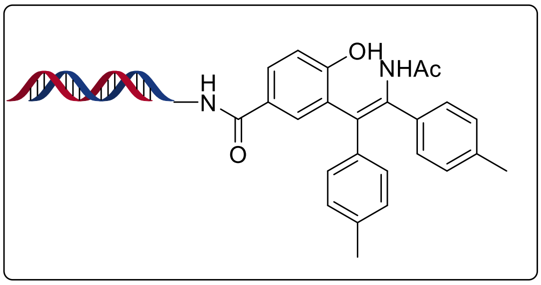

Following General Procedure **C14**
Yield: 33%
Exact mass: 5320.3821
Triply charged mass (M-3)/3, calculated 1772.4607; observed 1772.3784.

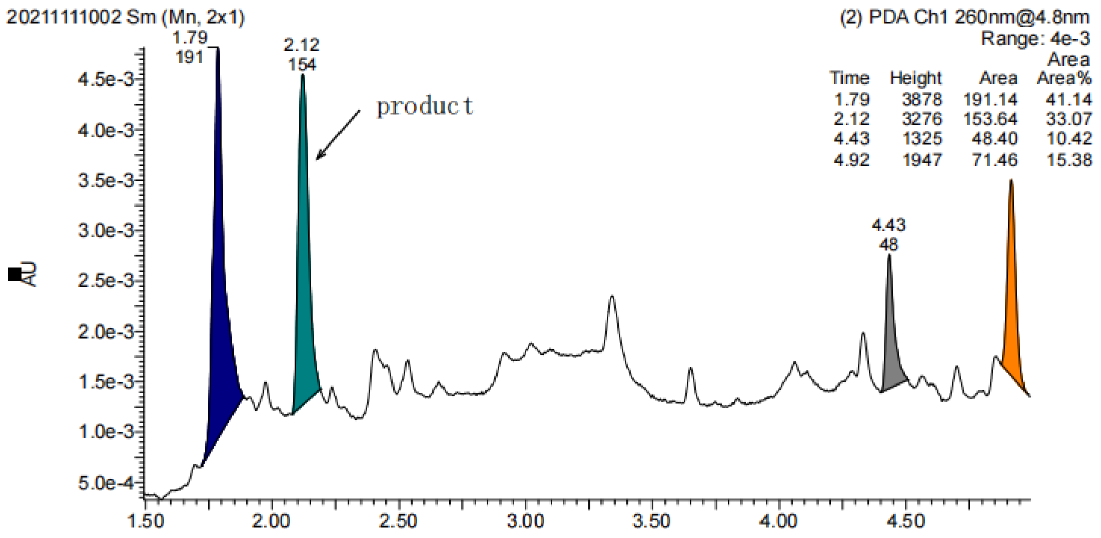

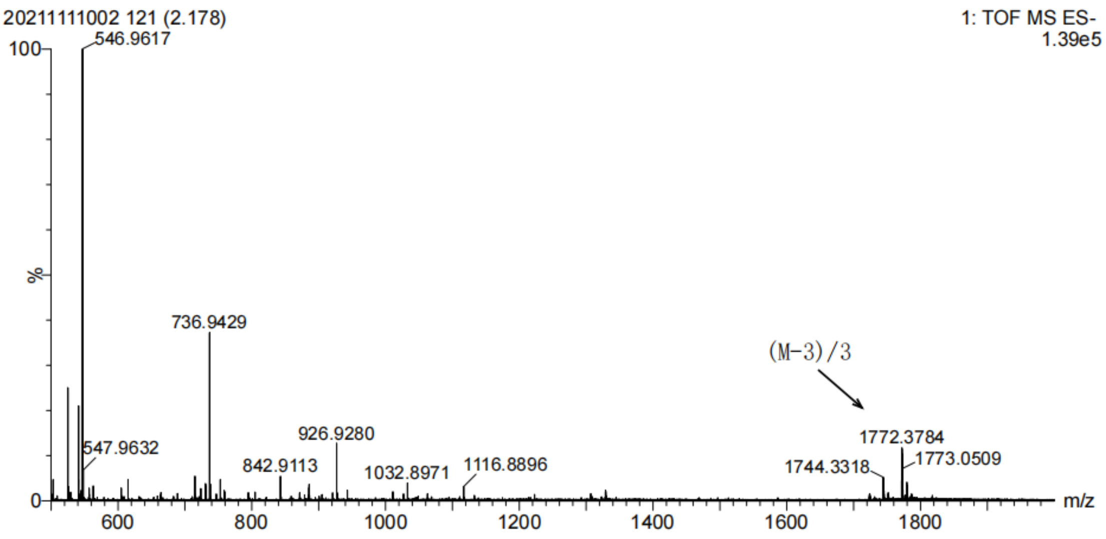

LC Trace and Mass of **C15**

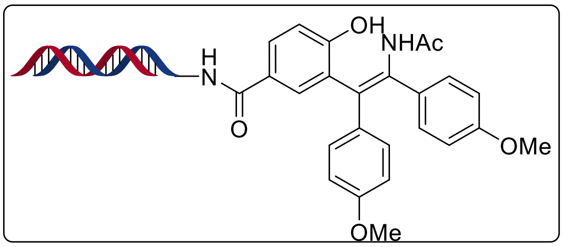

Following General Procedure **C15**

Yield: 44%

Exact mass: 5352.3720

Triply charged mass (M-3)/3, calculated 1783.1240; observed 1783.0422.

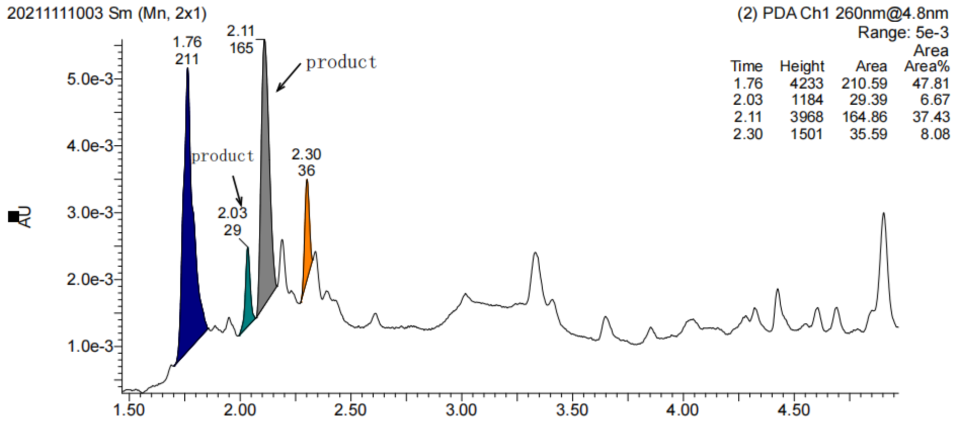

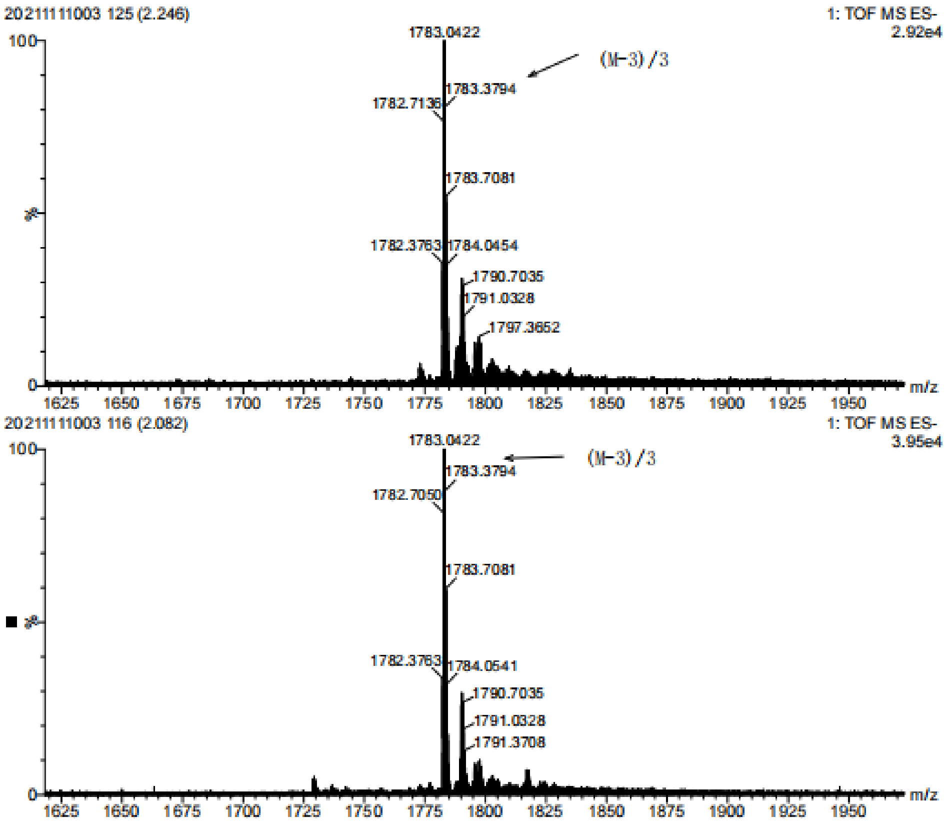

LC Trace and Mass of **C16**

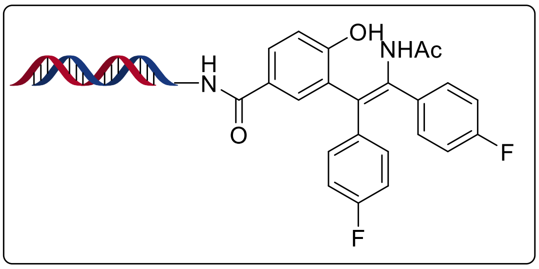

Following General Procedure **C16**
Yield: 56%
Exact mass: 5328.3321
Triply charged mass (M-3)/3, calculated 1775.1107; observed 1775.3146.

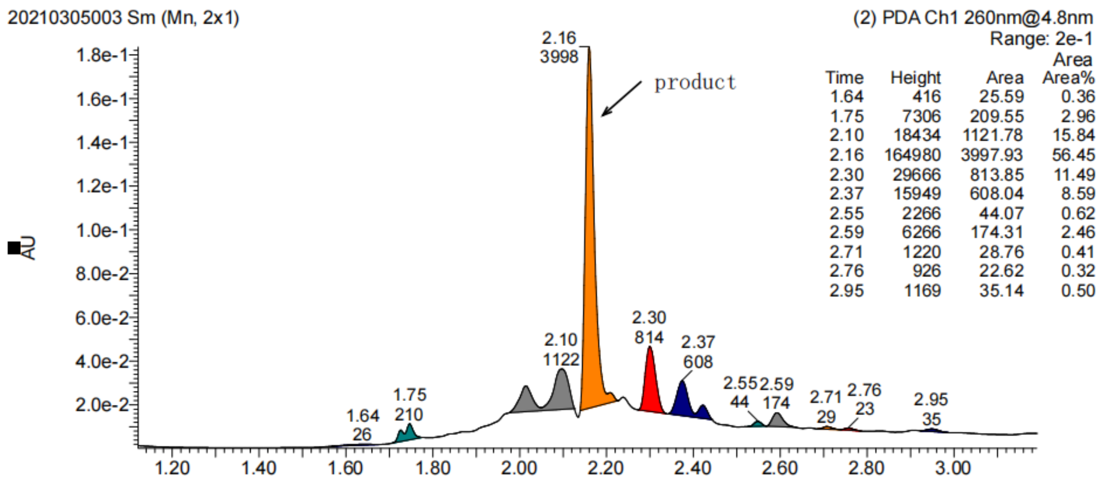

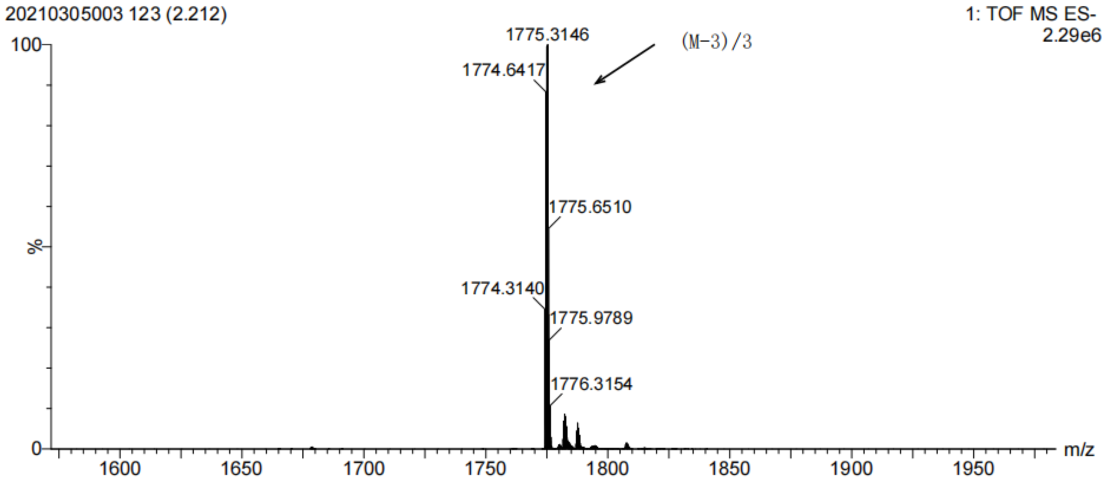

LC Trace and Mass of **C17**

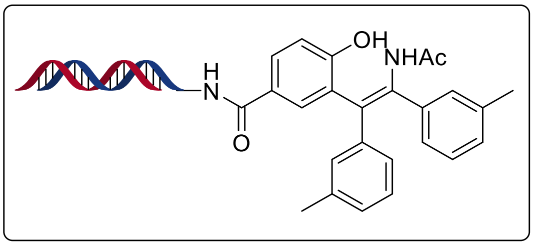

Following General Procedure **C17**

Yield: 66%

Exact mass: 5320.3821

Triply charged mass (M-3)/3, calculated 1772.4607; observed 1772.3715.

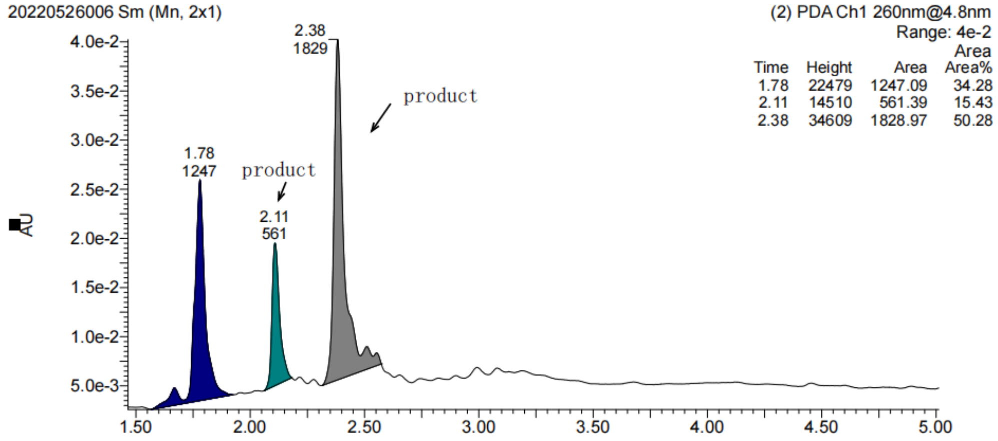

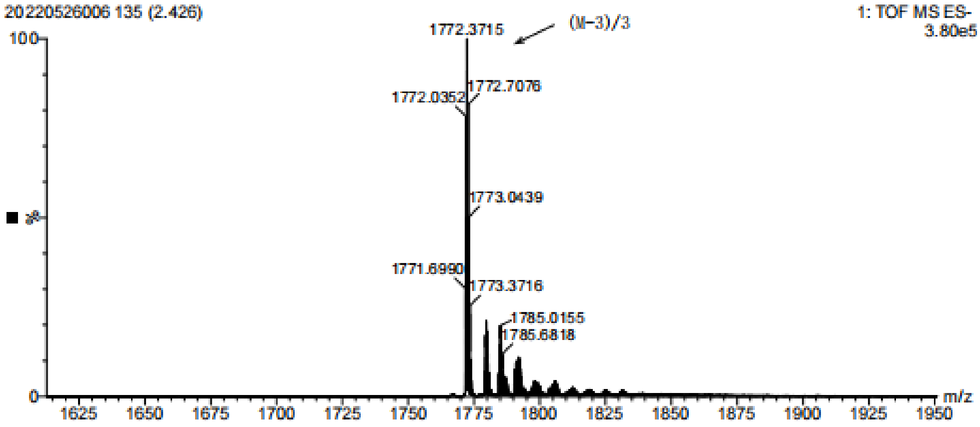

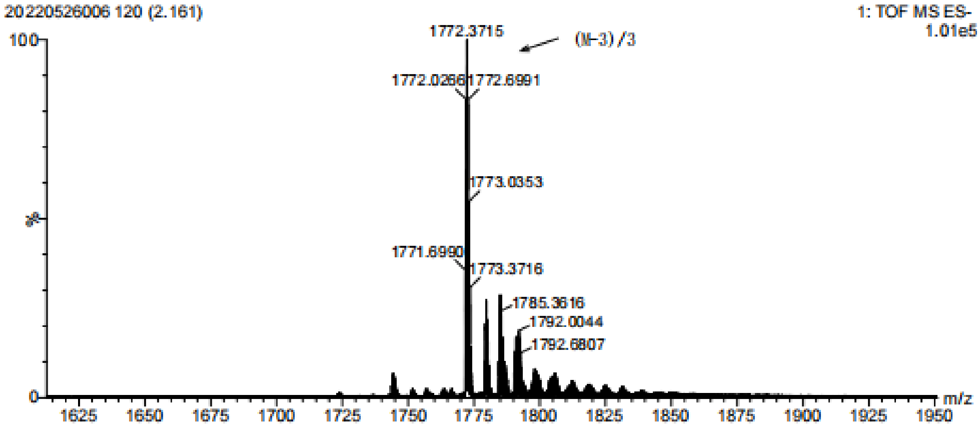

LC Trace and Mass of **C18**

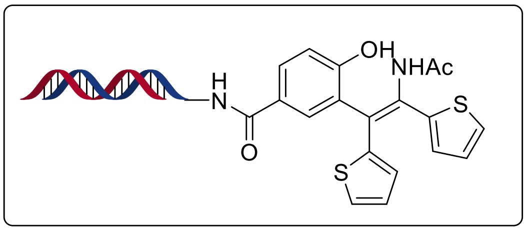

Following General Procedure **C18**

Yield: 67%

Exact mass: 5304.2637

Triply charged mass (M-3)/3, calculated 1767.0879; observed 1766.9954.

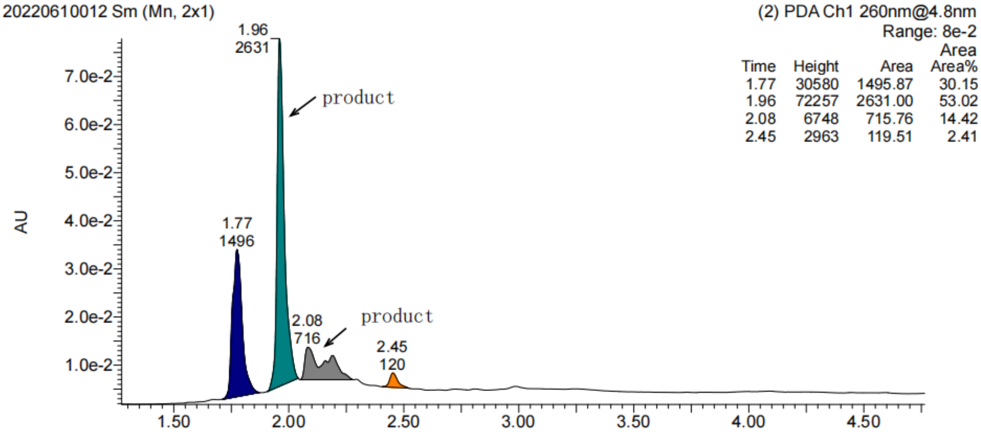

Time	Height	Area	Area Area%
1.77	30580	1495.87	30.15
1.96	72257	2631.00	53.02
2.08	6748	715.76	14.42
2.45	2963	119.51	2.41

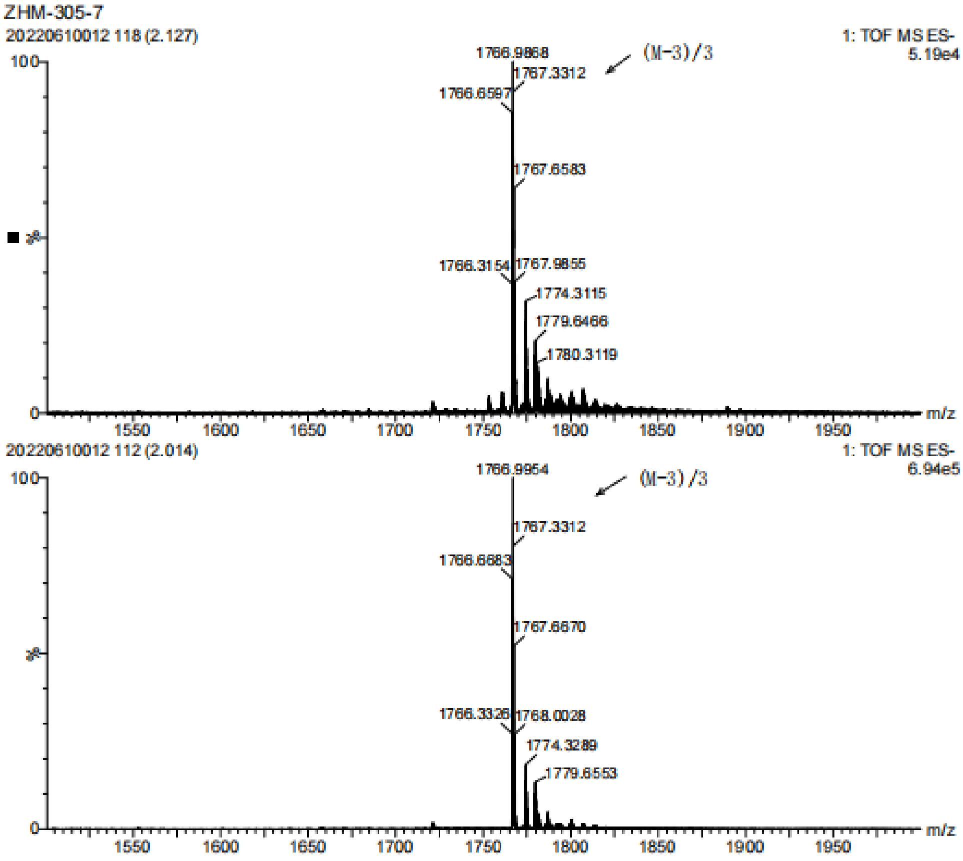

LC Trace and Mass of **C19**

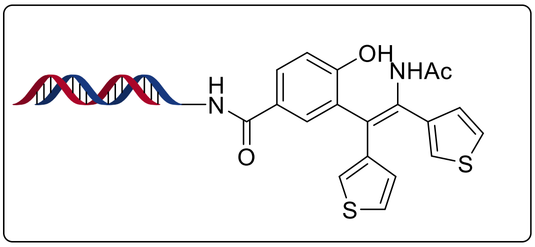

Following General Procedure **C19**

Yield: 78%

Exact mass: 5304.2637

Triply charged mass (M-3)/3, calculated 1767.0879; observed 1767.0198.

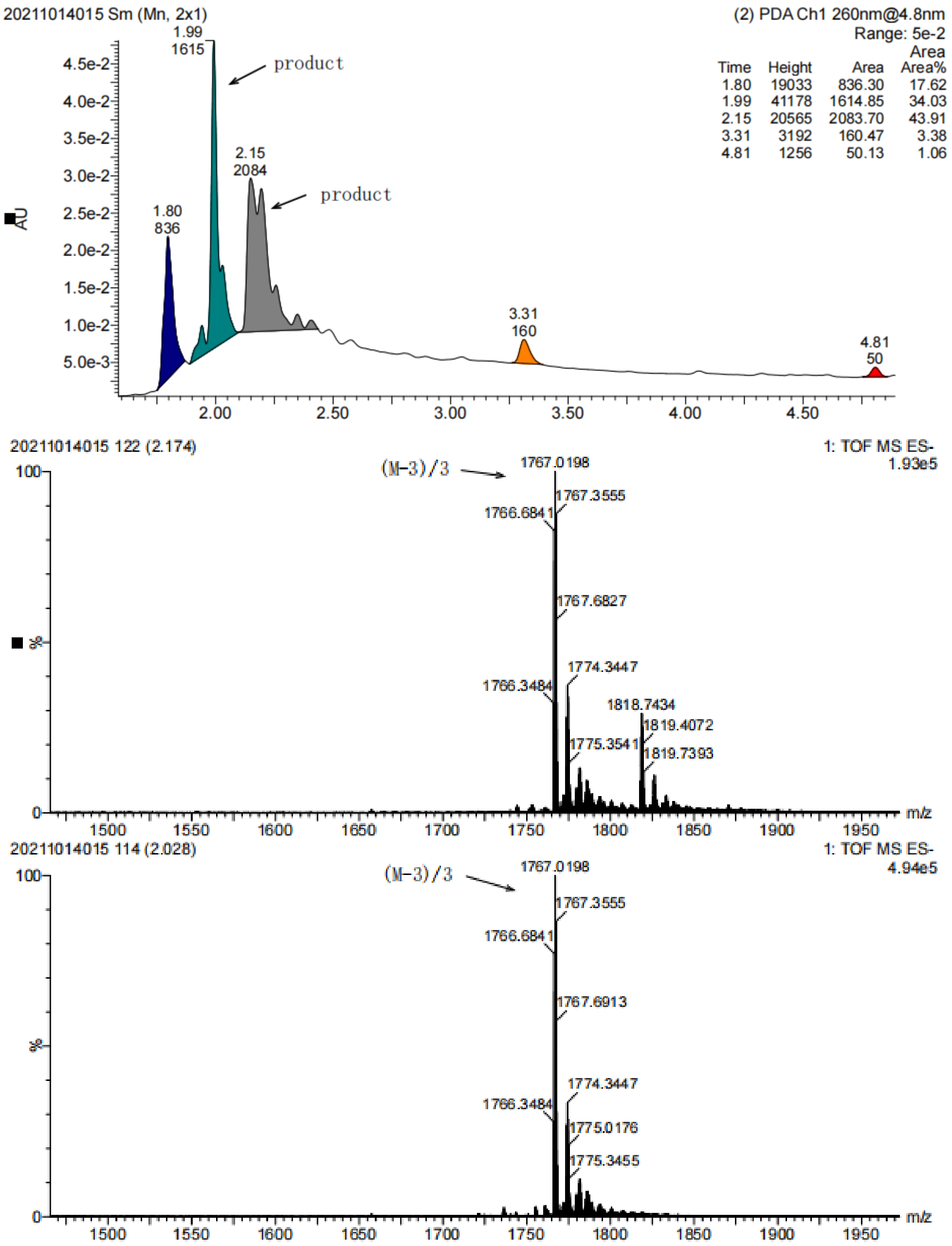

LC Trace and Mass of **C20**

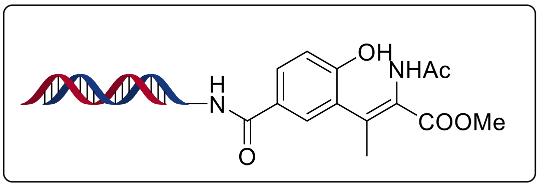

Following General Procedure **C20**
Yield: 48%
Exact mass: 5212.3197
Triply charged mass (M-3)/3, calculated 1736.4399; observed 1736.3369.

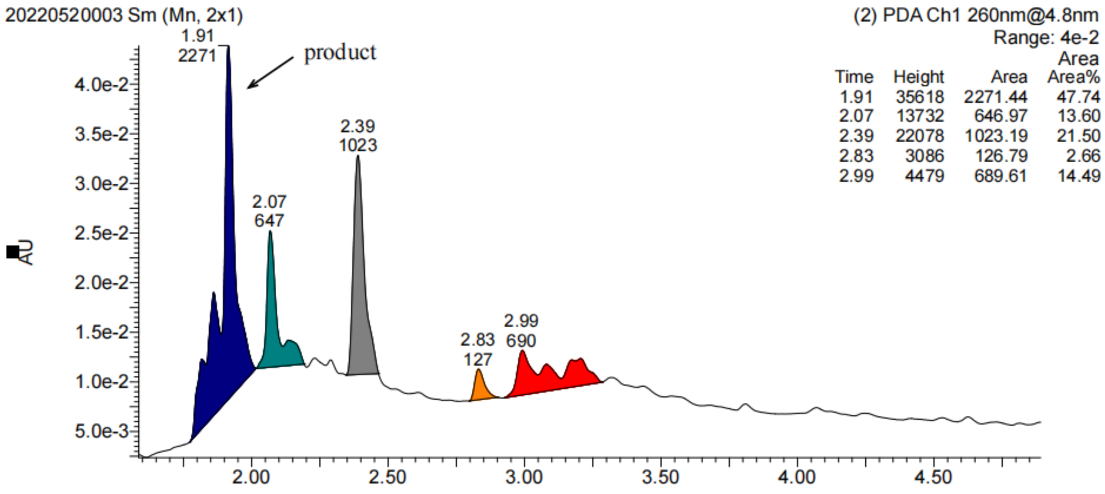

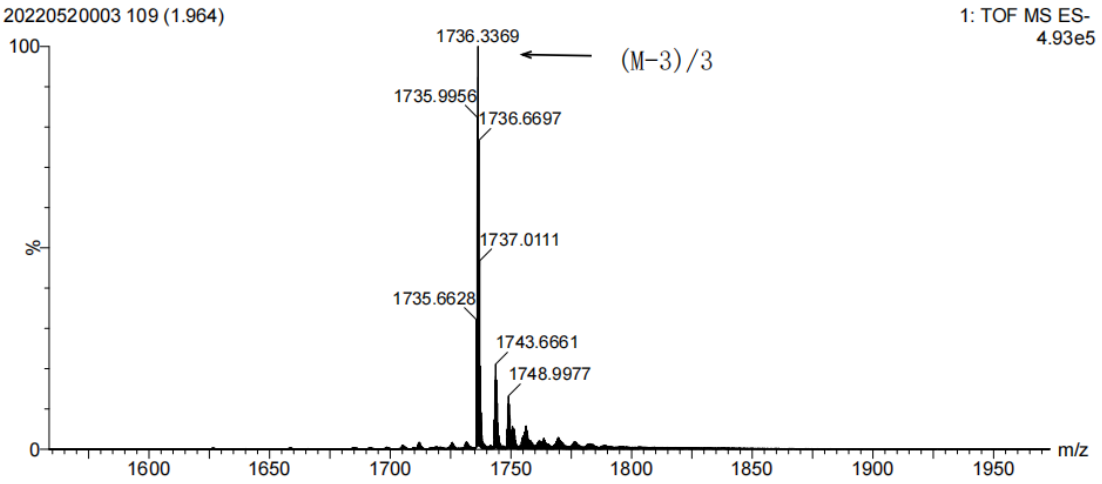

LC Trace and Mass of **C21**

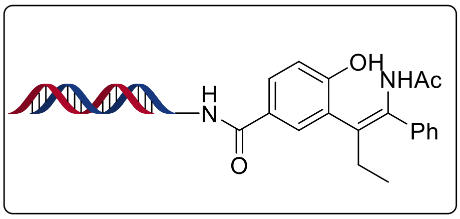

Following General Procedure **C21**
Yield: 82%
Exact mass: 5244.3507
Triply charged mass (M-3)/3, calculated 1747.1169; observed 1746.9863.

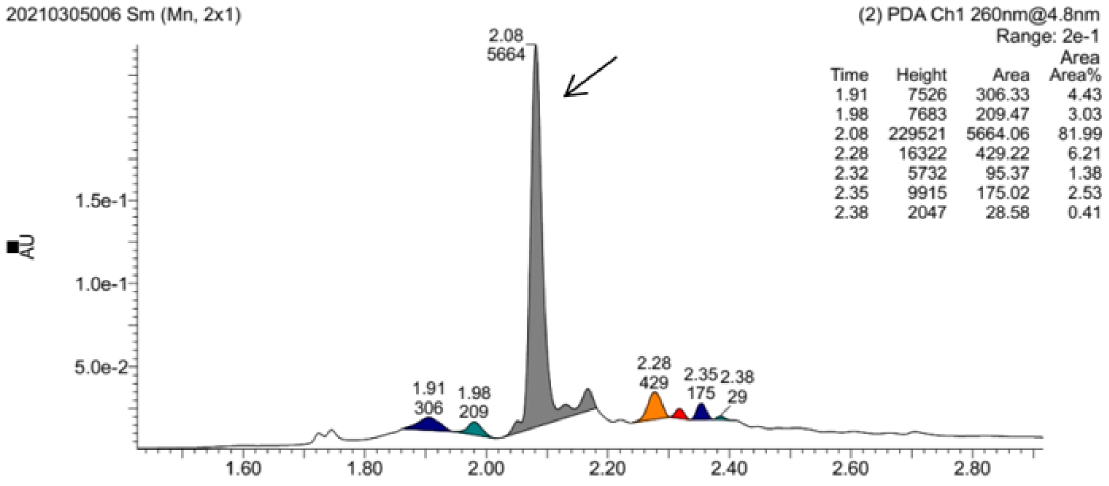

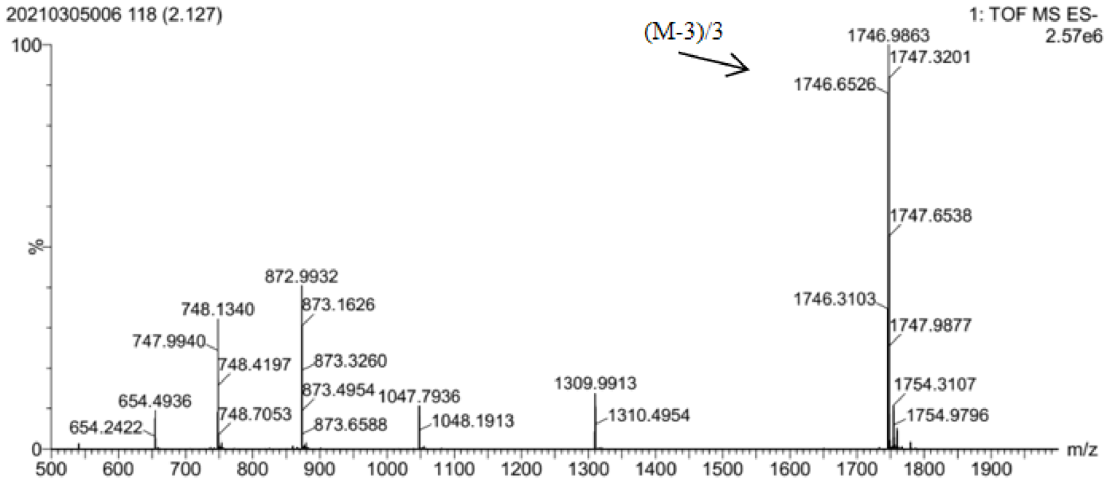

LC Trace and Mass of **C22**

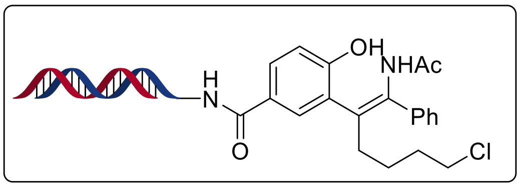

Following General Procedure **C22**
Yield: 50%
Exact mass: 5307.2691
Triply charged mass (M-3)/3, calculated 1768.0897; observed 1768.7085.

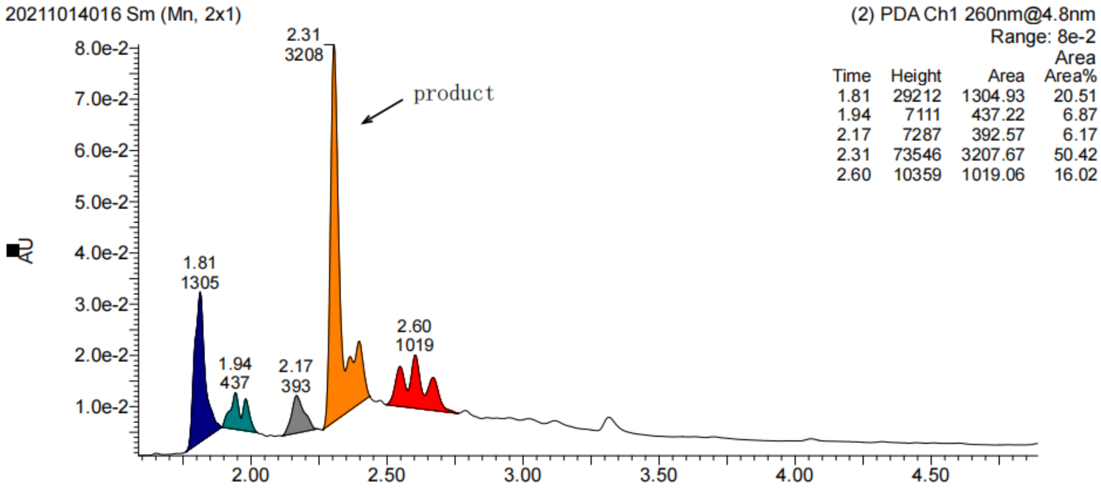

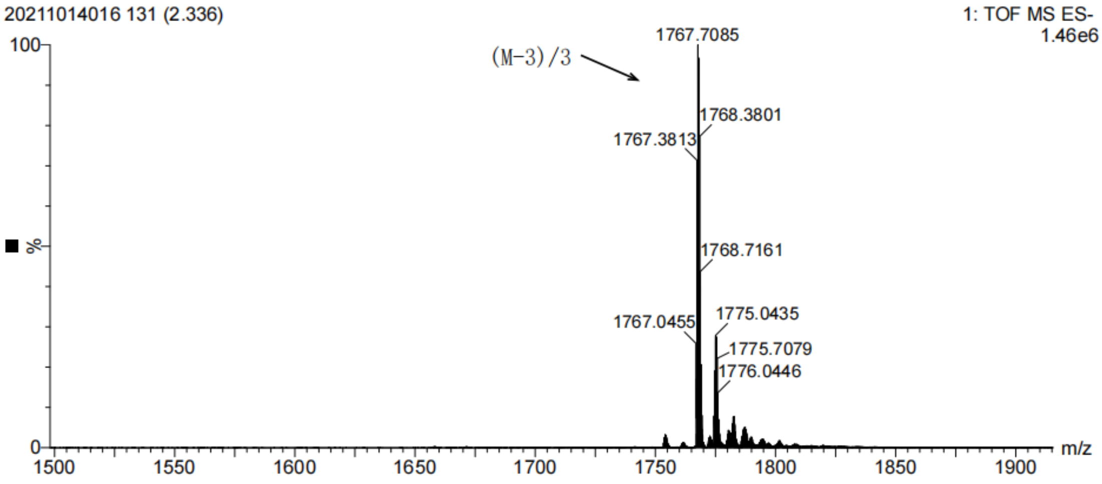

LC Trace and Mass of **C23**

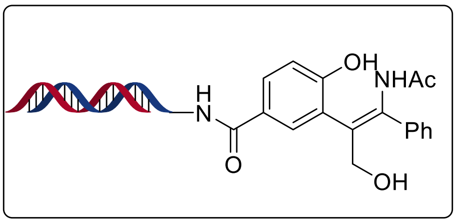

Following General Procedure **C23**

Yield: 62%

Exact mass: 5246.3406

Triply charged mass (M-3)/3, calculated 1747.7802; observed 1747.5853.

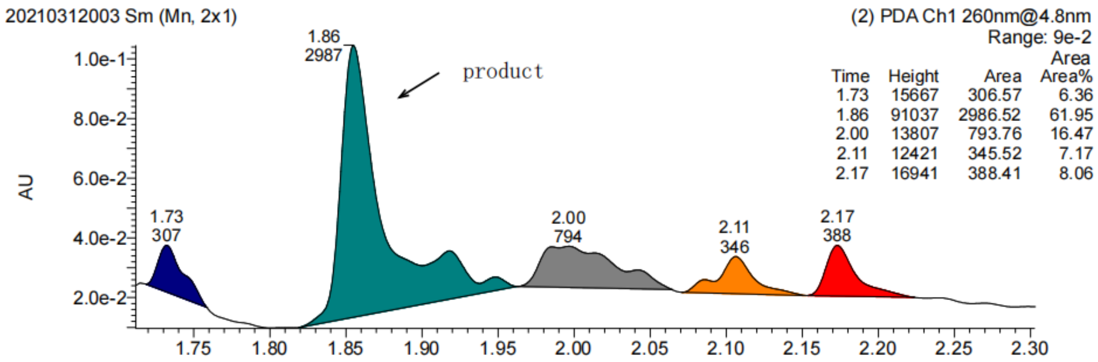

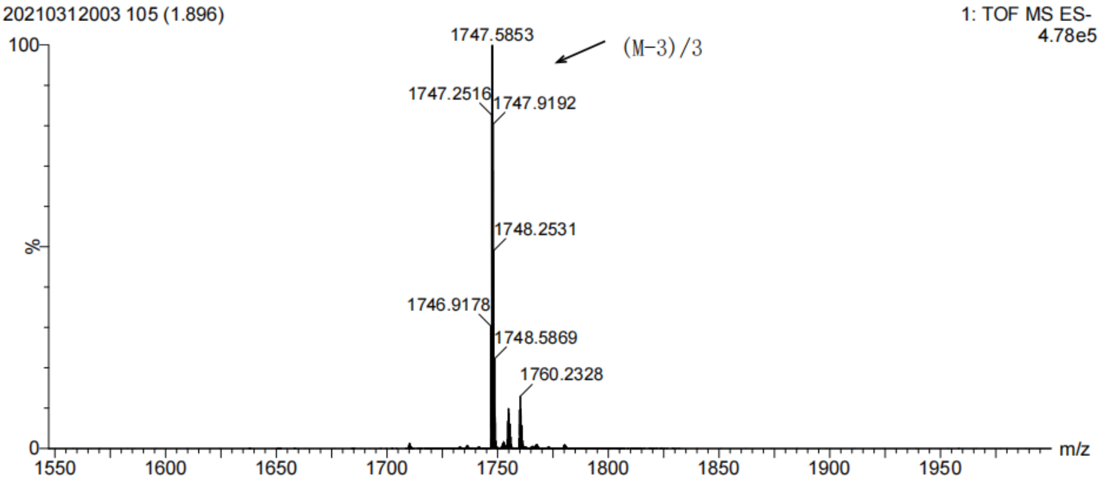

LC Trace and Mass of **C24**

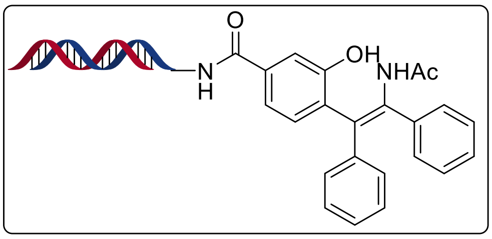

Following General Procedure **C24**
Yield: 64%
Exact mass: 5292.3507
Triply charged mass (M-3)/3, calculated 1763.1169; observed 1763.0292.

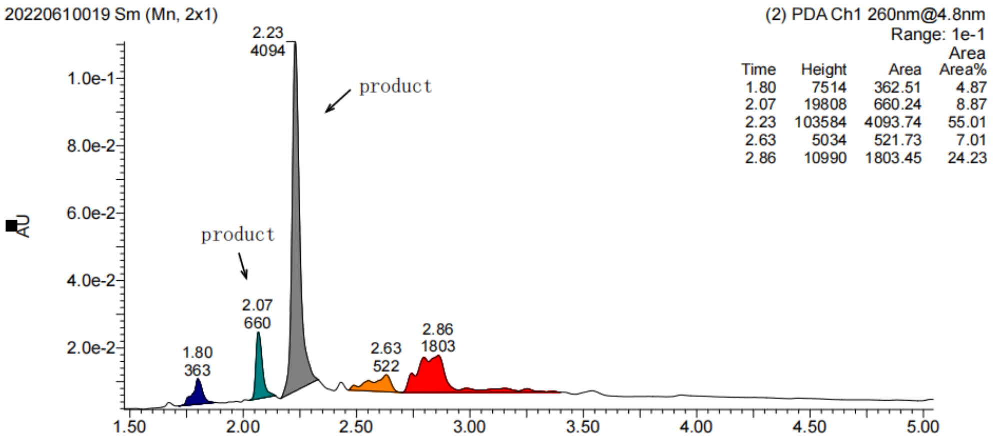

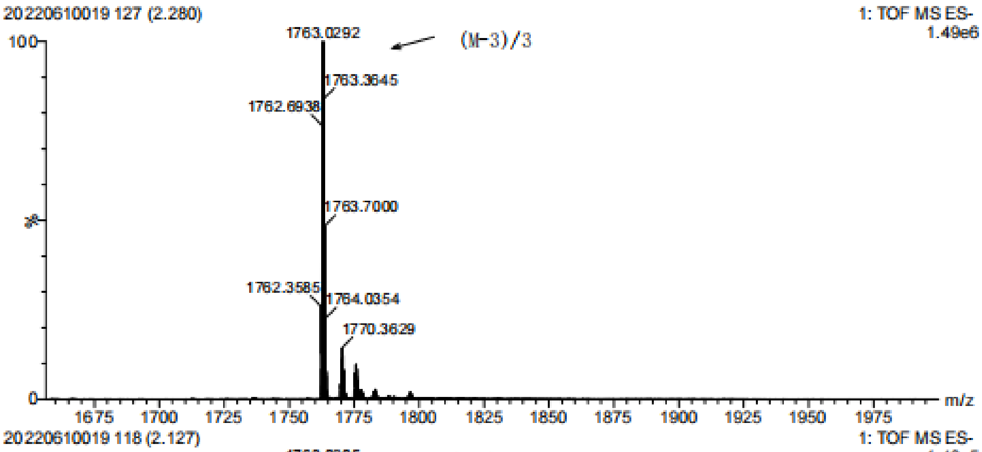

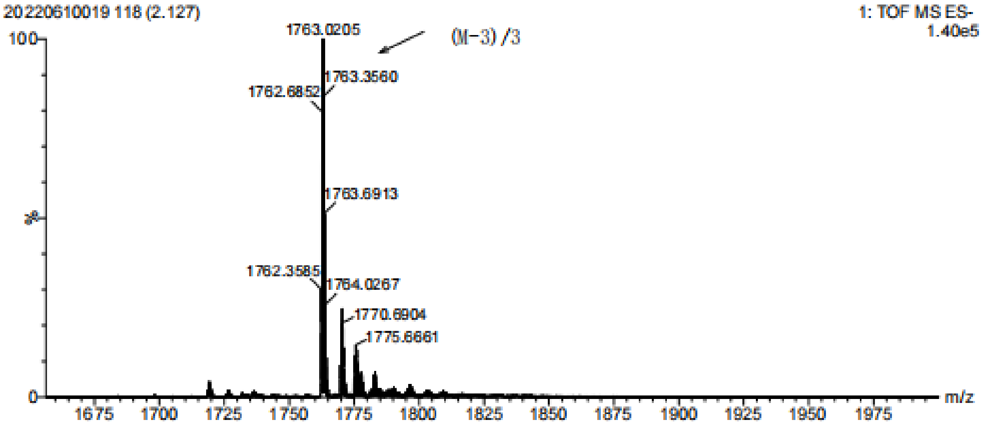

LC Trace and Mass of **C25**

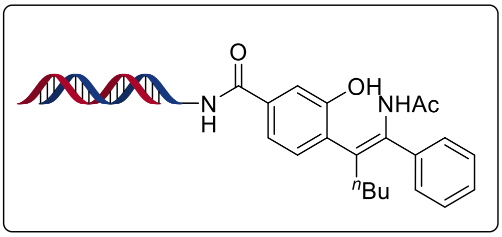

Following General Procedure **C25**

Yield: 63%

Exact mass: 5272.3926

Triply charged mass (M-3)/3, calculated 1756.4642; observed 1756.3721.

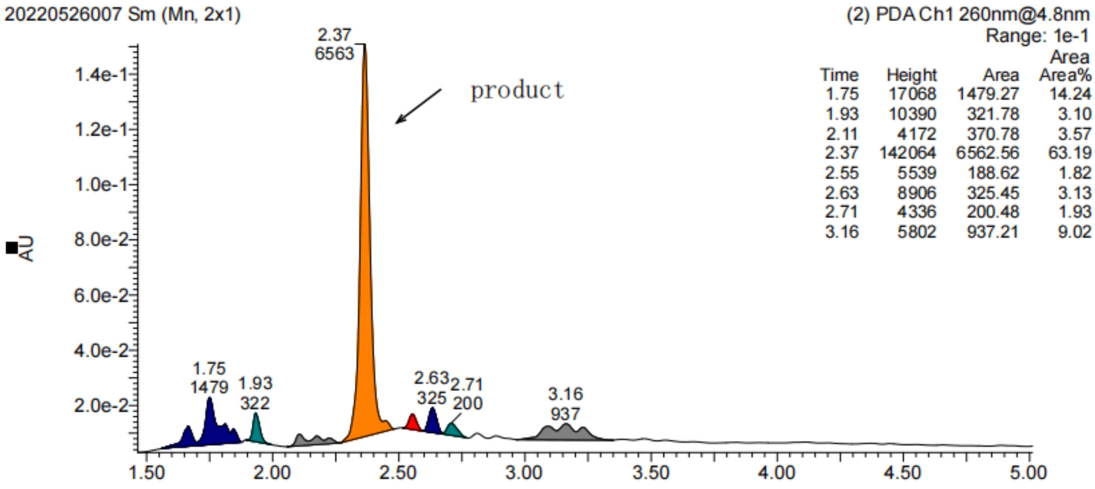

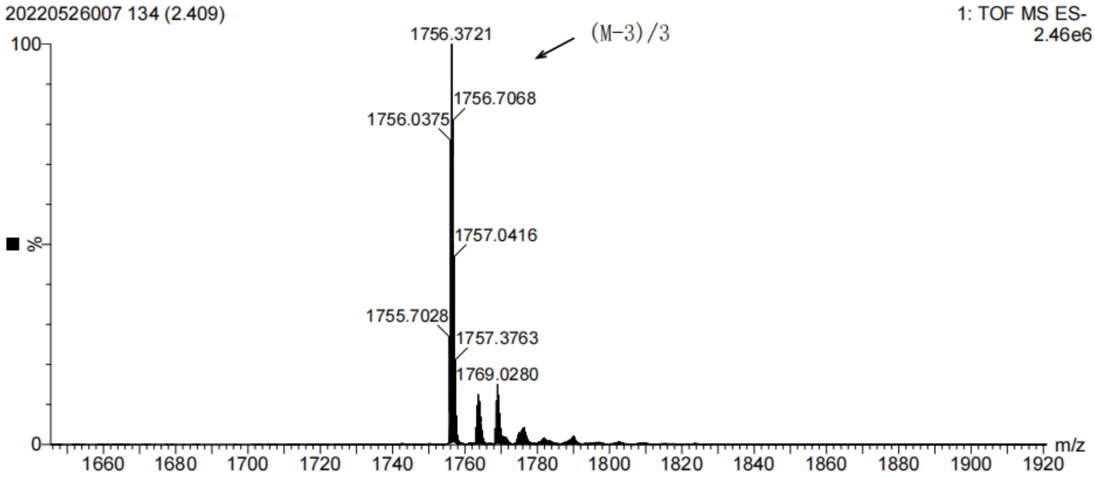

LC Trace and Mass of **C26**

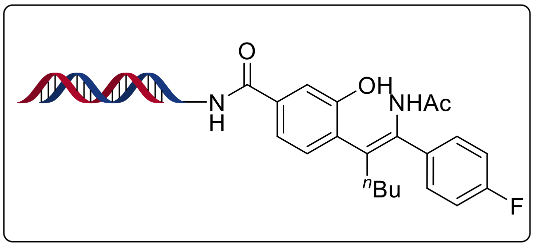

Following General Procedure **C26**

Yield: 65%

Exact mass: 5290.3833

Triply charged mass (M-3)/3, calculated 1762.4611; observed 1762.3762.

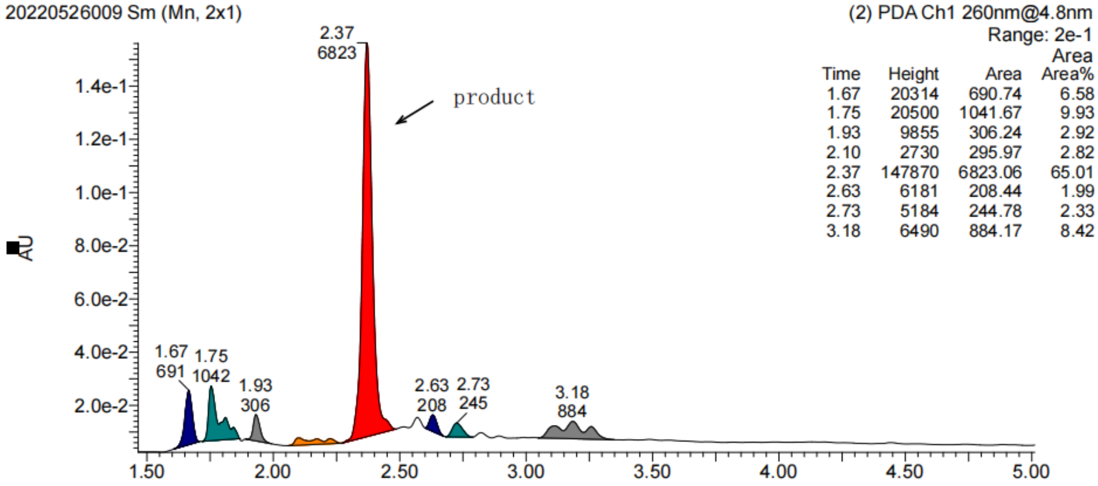

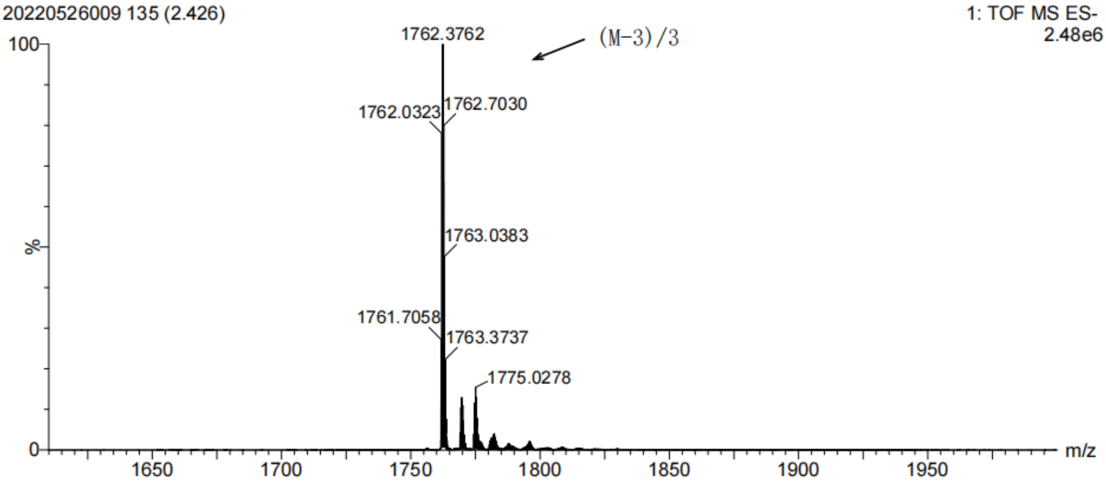

LC Trace and Mass of **D1**

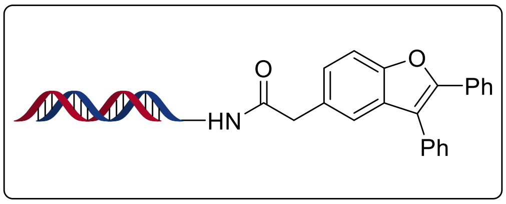

Following General Procedure **D1**

Yield: 74%

Exact mass: 5247.3399

Triply charged mass (M-3)/3, calculated 1748.1133; observed 1748.0350.

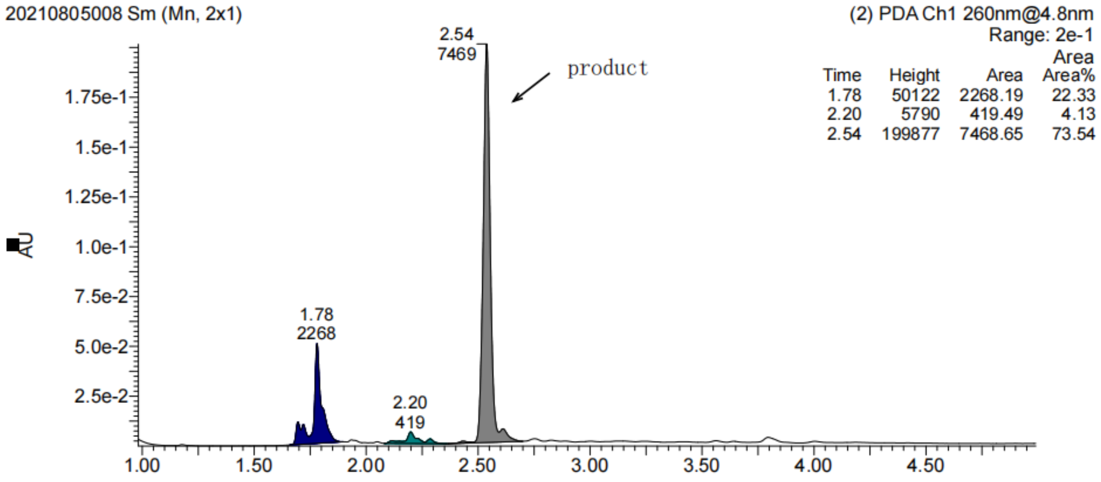

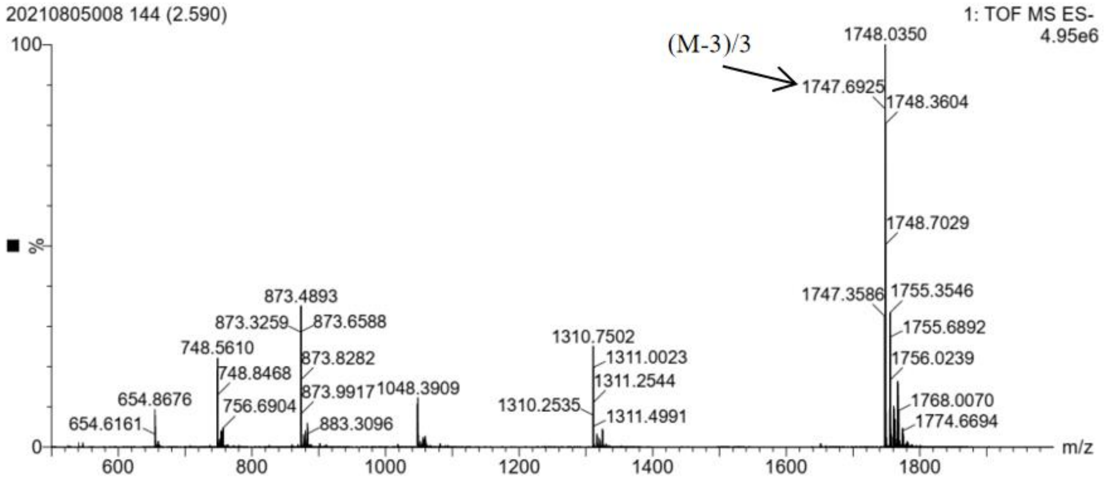

LC Trace and Mass of **D2**

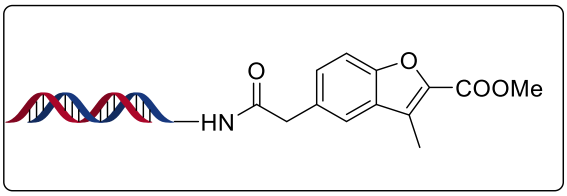

Following General Procedure **D2**

Yield: 78%

Exact mass: 5167.4154

Triply charged mass (M-3)/3, calculated 1721.4718; observed 1721.3485.

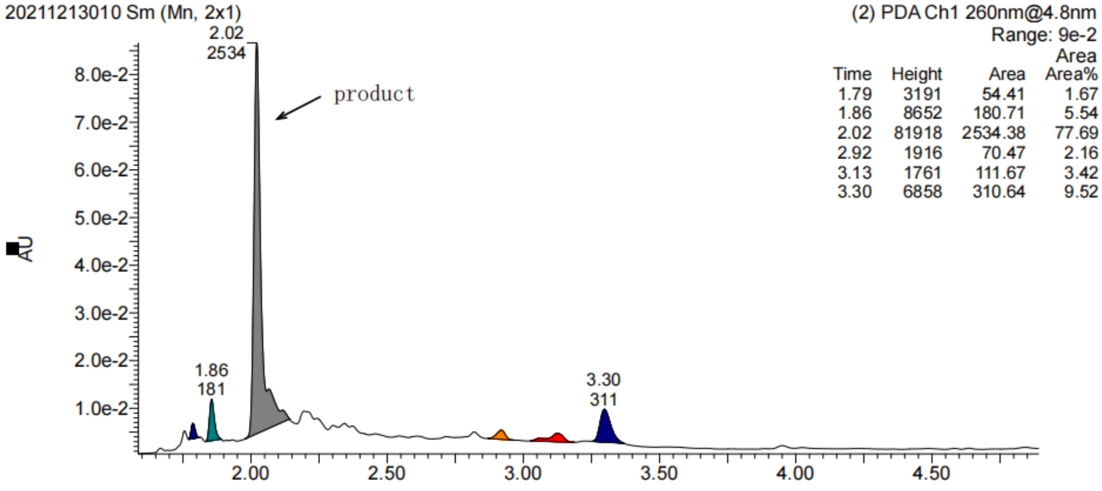

Time	Height	Area	Area Area%
1.79	3191	54.41	1.67
1.86	8652	180.71	5.54
2.02	81918	2534.38	77.69
2.92	1916	70.47	2.16
3.13	1761	111.67	3.42
3.30	6858	310.64	9.52

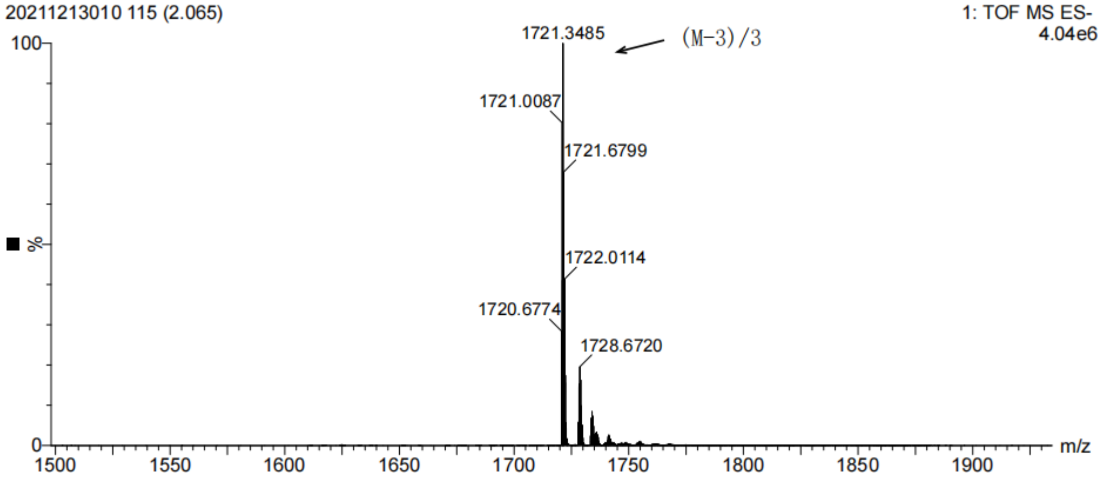

LC Trace and Mass of **D3**

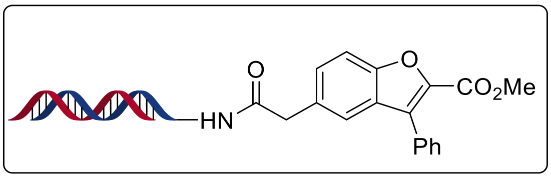

Following General Procedure **D3**
Yield: 53%
Exact mass: 5229.3036
Triply charged mass (M-3)/3, calculated 1742.1012; observed 1742.0210.

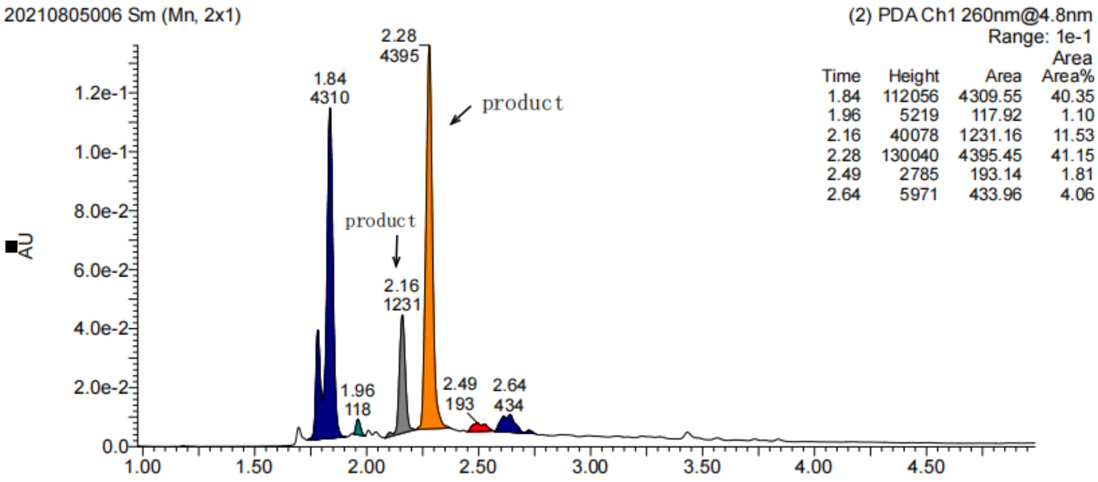

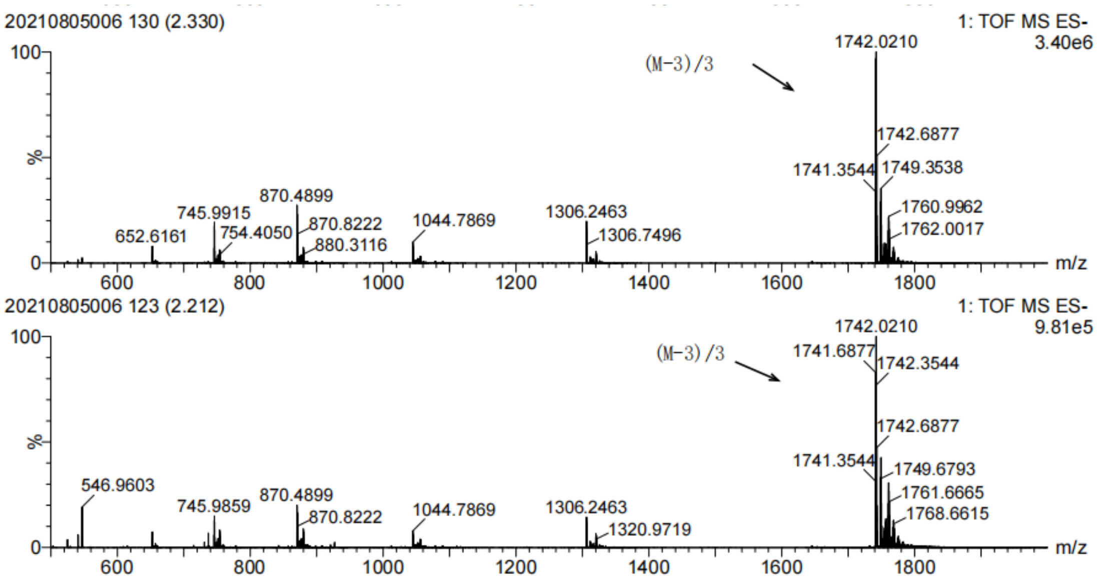

LC Trace and Mass of **D4**

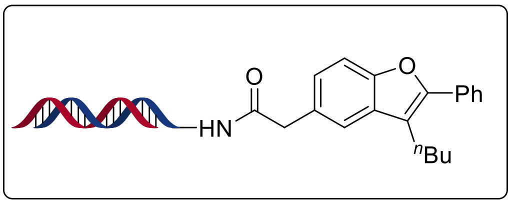

Following General Procedure **D4**

Yield: 82%

Exact mass: 5227.3605

Triply charged mass (M-3)/3, calculated 1741.4535; observed 1741.3715.

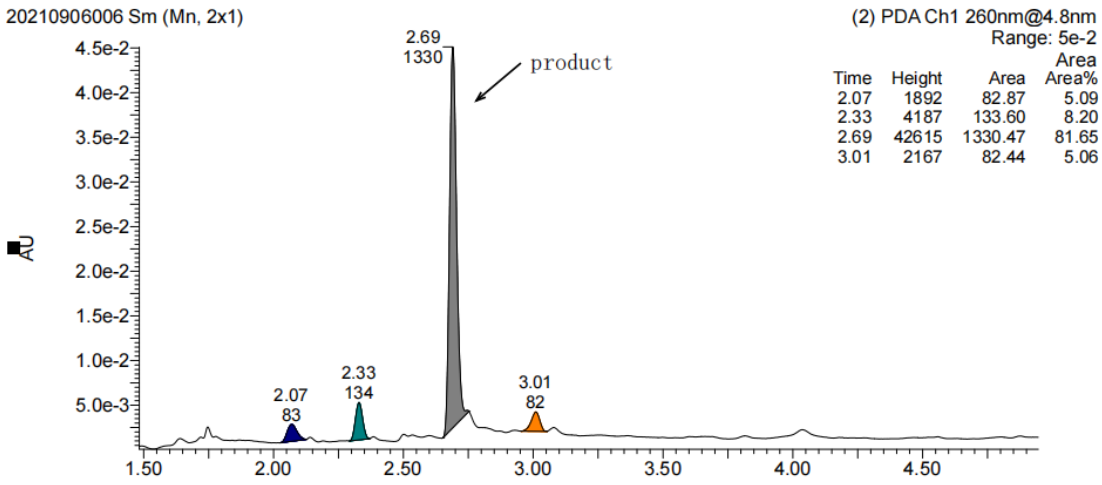

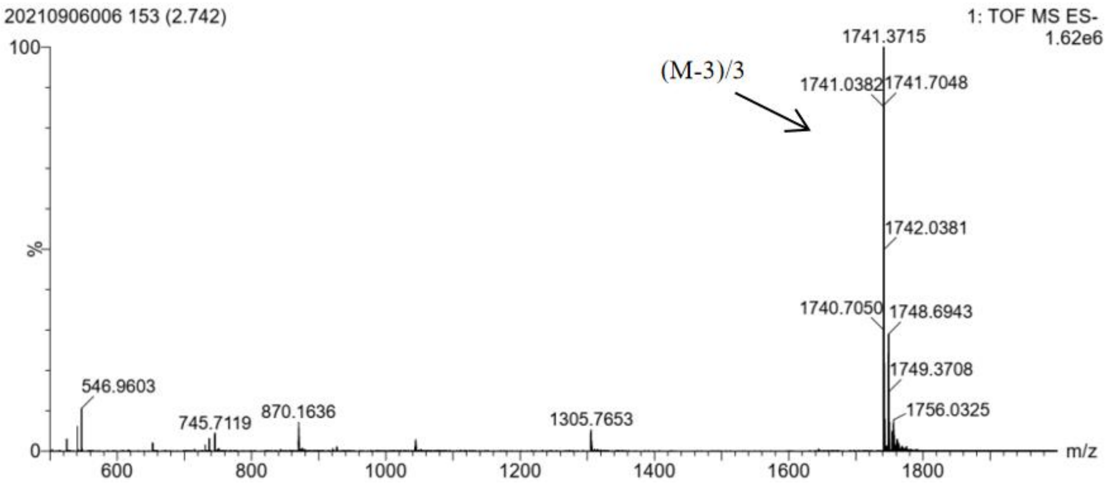

LC Trace and Mass of **D5**

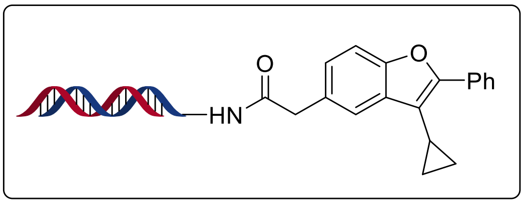

Following General Procedure **D5**

Yield: 69%

Exact mass: 5211.3294

Triply charged mass (M-3)/3, calculated 1736.1098; observed 1736.0260.

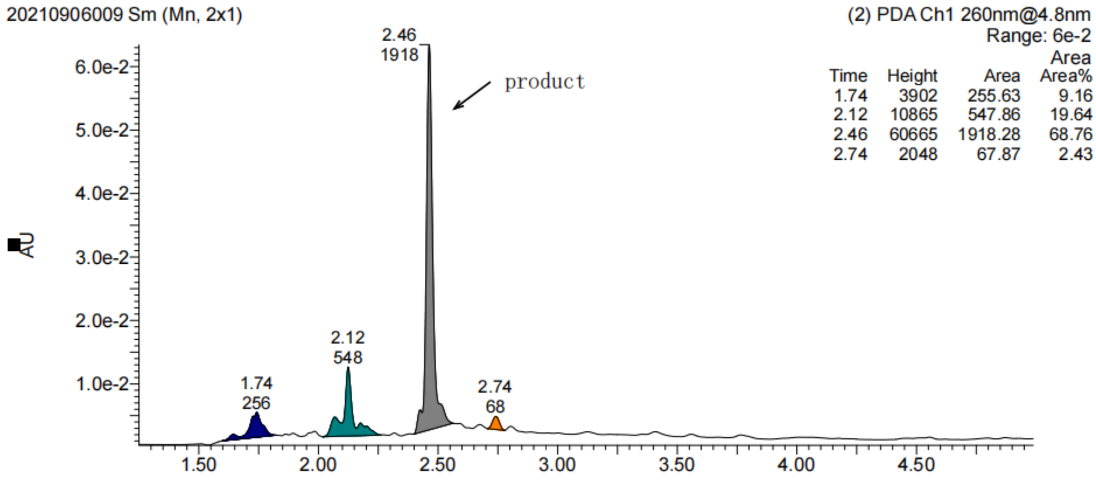

Time	Height	Area	Area%
1.74	3902	255.63	9.16
2.12	10865	547.86	19.64
2.46	60665	1918.28	68.76
2.74	2048	67.87	2.43

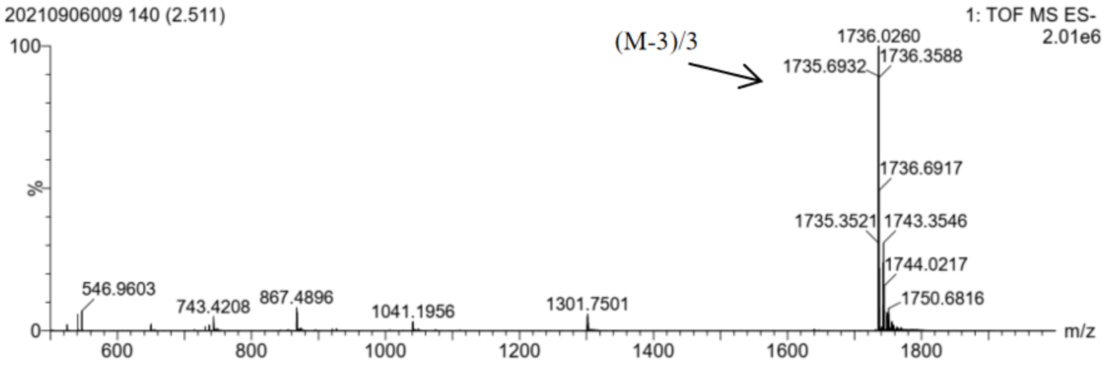

LC Trace and Mass of **D6**

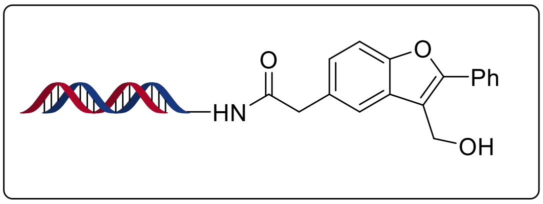

Following General Procedure **D6**
Yield: 63%
Exact mass: 5201.3085
Triply charged mass (M-3)/3, calculated 1732.7695; observed 1732.6914.

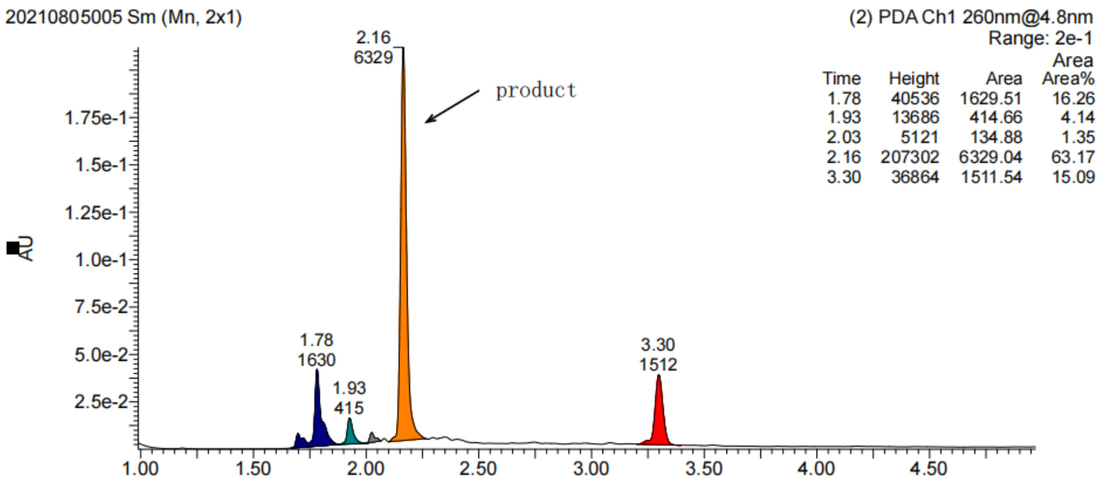

Time	Height	Area	Area Area%
1.78	40536	1629.51	16.26
1.93	13686	414.66	4.14
2.03	5121	134.88	1.35
2.16	207302	6329.04	63.17
3.30	36864	1511.54	15.09

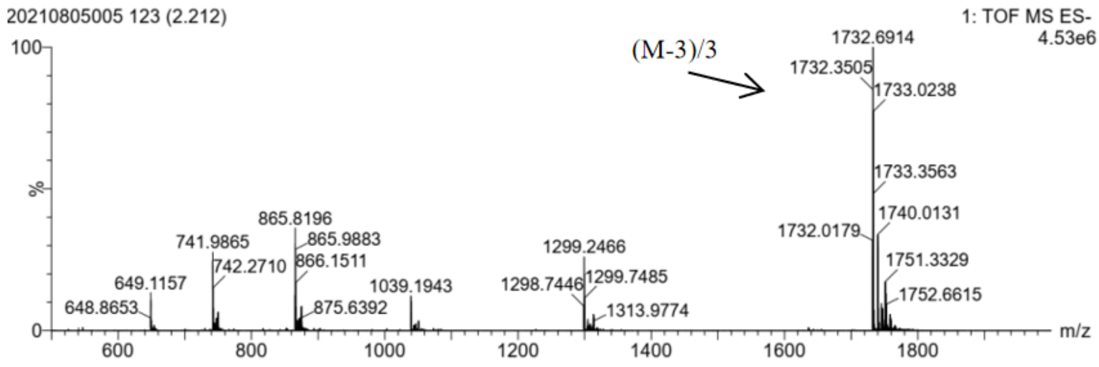

LC Trace and Mass of **D7**

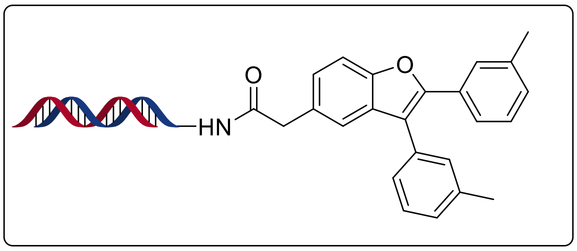

Following General Procedure **D7**
Yield: 53%
Exact mass: 5275.4883
Triply charged mass (M-3)/3, calculated 1757.4961; observed 1757.3467.

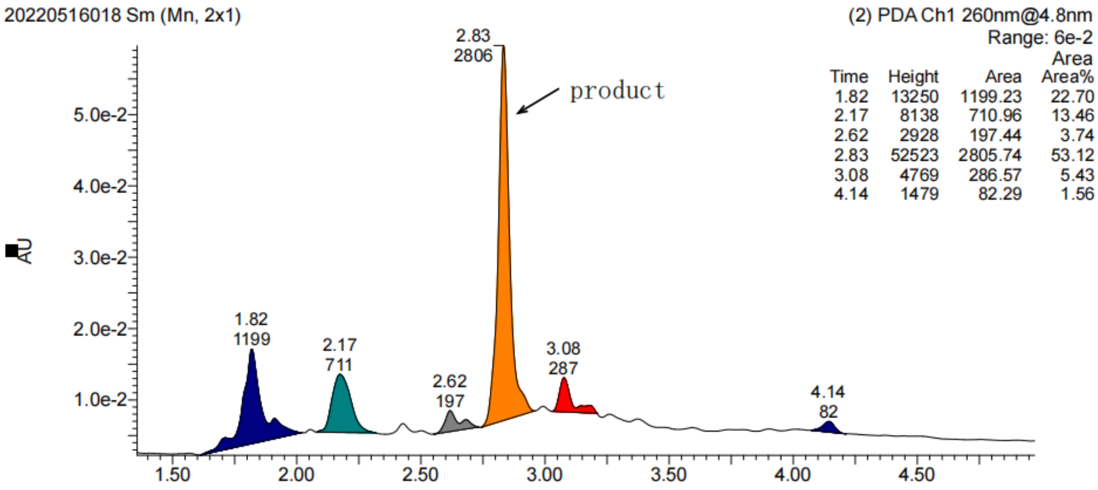

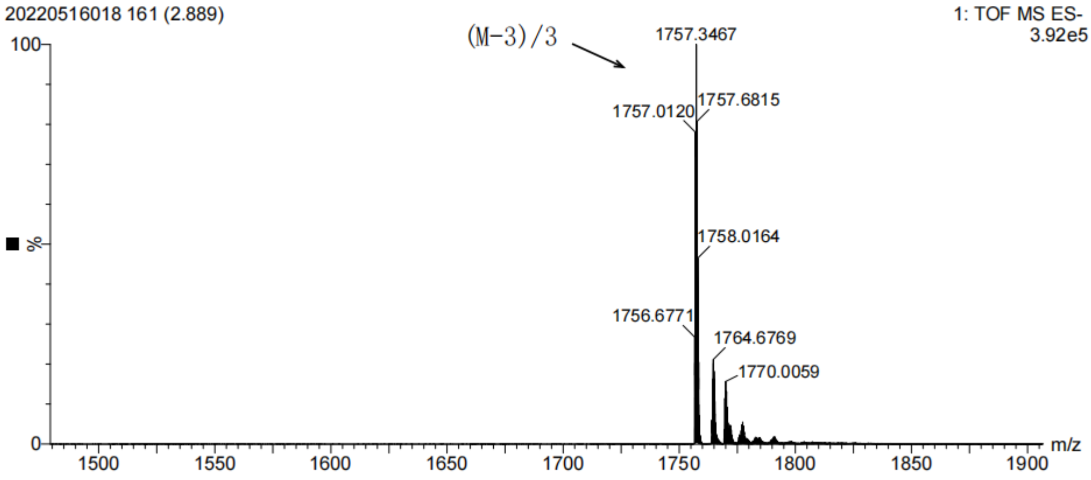

LC Trace and Mass of **D8**

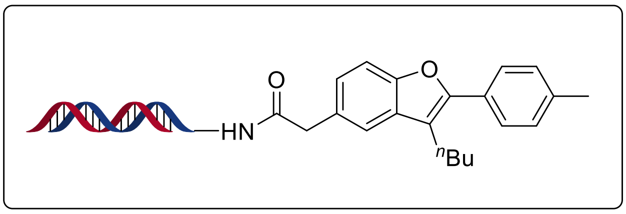

Following General Procedure **D8**
Yield: 77%
Exact mass: 5241.3762
Triply charged mass (M-3)/3, calculated 1746.1254; observed 1746.0405.

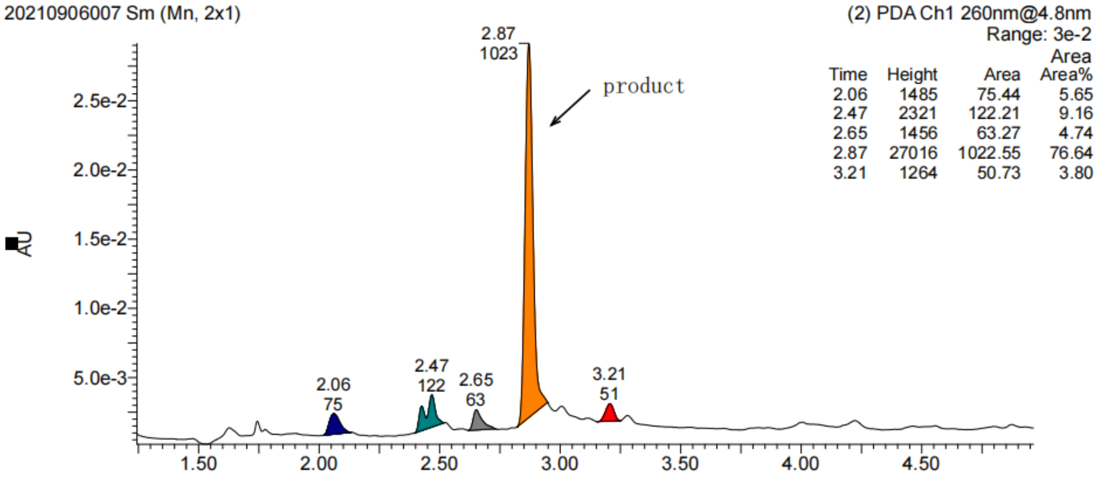

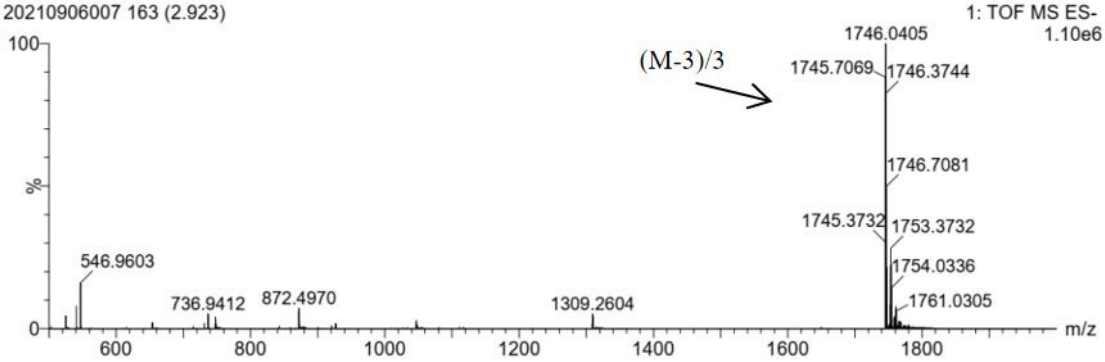

LC Trace and Mass of **D9**

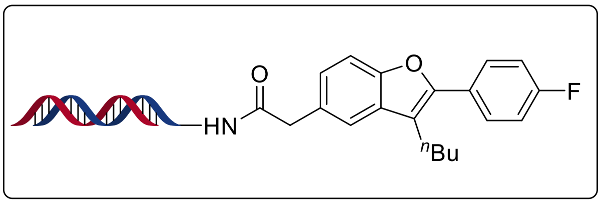

Following General Procedure **D9**

Yield: 68%

Exact mass: 5245.3512

Triply charged mass (M-3)/3, calculated 1747.4504; observed 1747.3672.

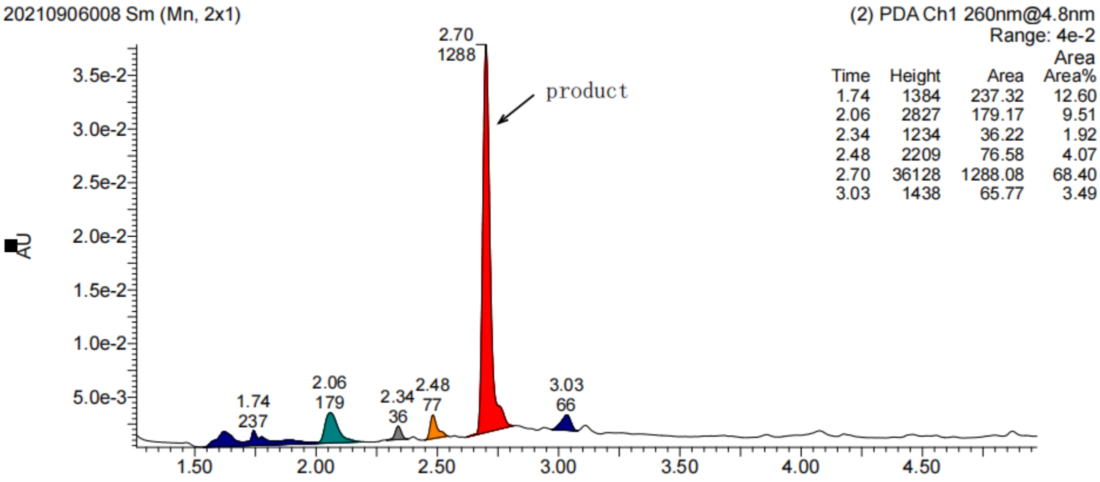

Time	Height	Area	Area Area%
1.74	1384	237.32	12.60
2.06	2827	179.17	9.51
2.34	1234	36.22	1.92
2.48	2209	76.58	4.07
2.70	36128	1288.08	68.40
3.03	1438	65.77	3.49

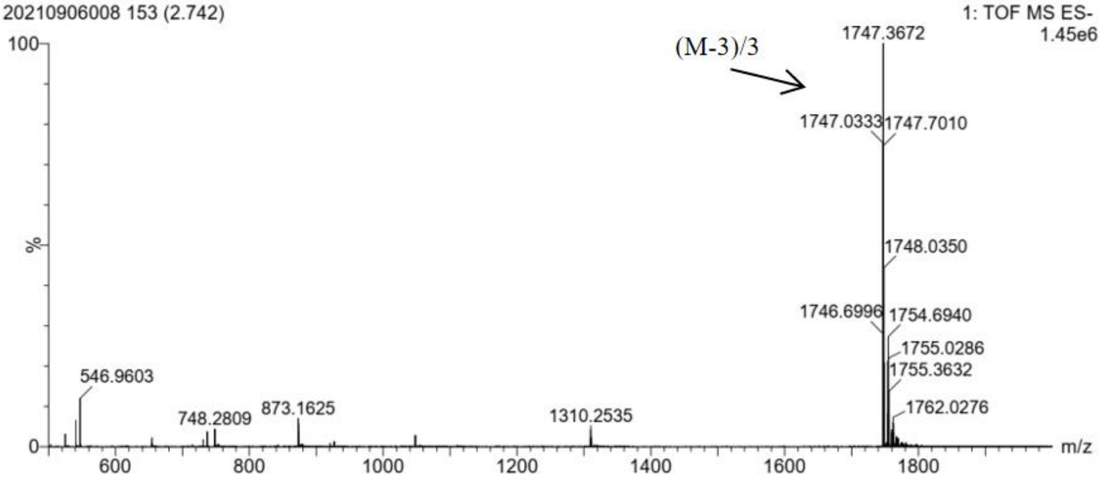

LC Trace and Mass of **D10**

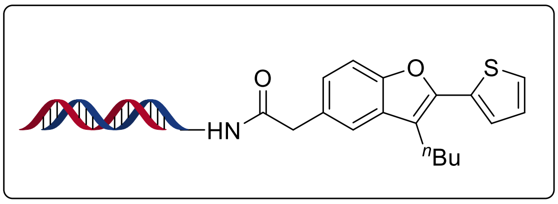

Following General Procedure **D10**

Yield: 58%

Exact mass: 5233.3170

Triply charged mass (M-3)/3, calculated 1743.4390; observed 1743.3546.

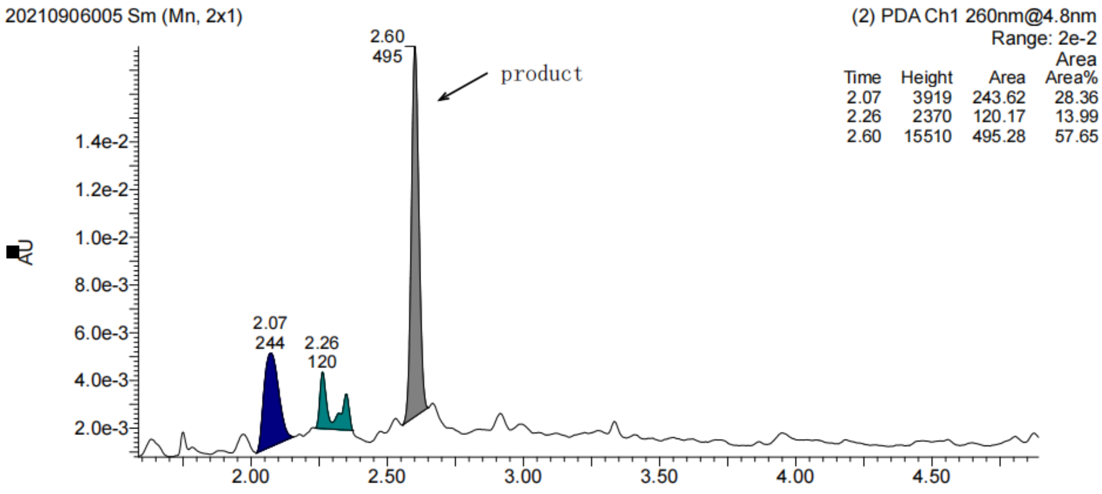

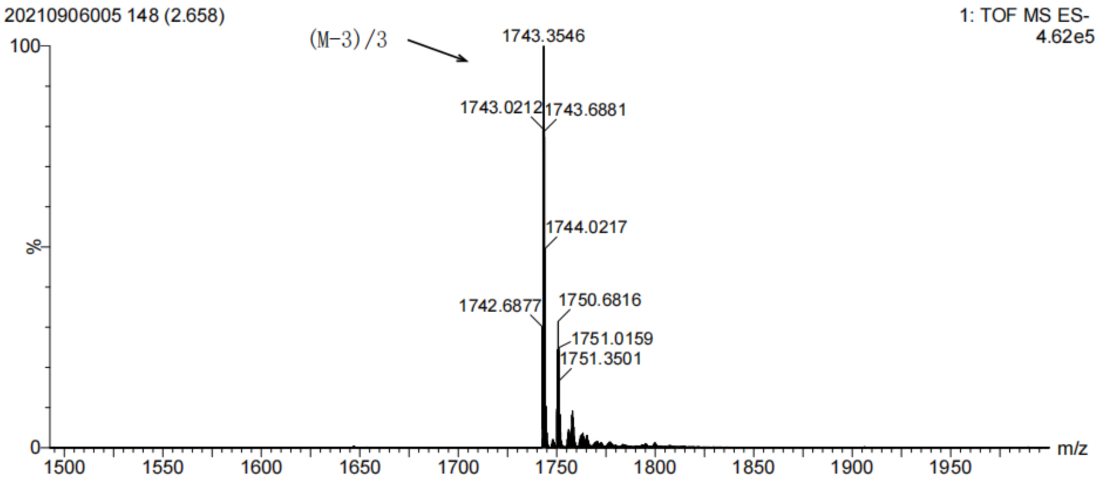

LC Trace and Mass of **D11**

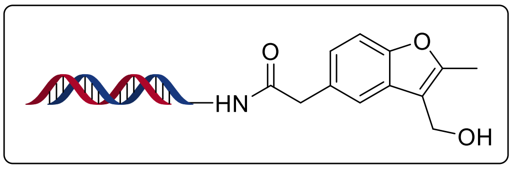

Following General Procedure **D11**
Yield: 71%
Exact mass: 5139.2931
Triply charged mass (M-3)/3, calculated 1712.0977; observed 1712.0135.

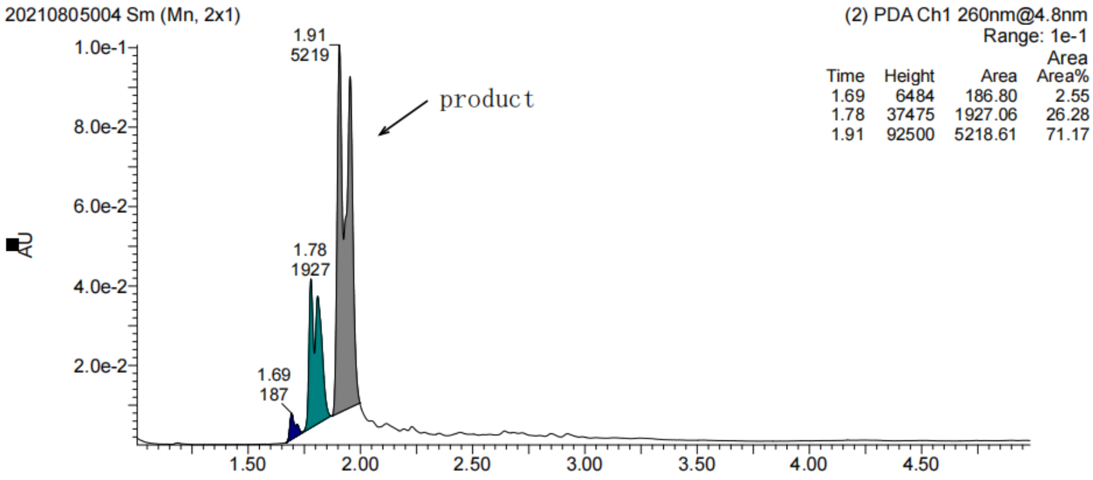

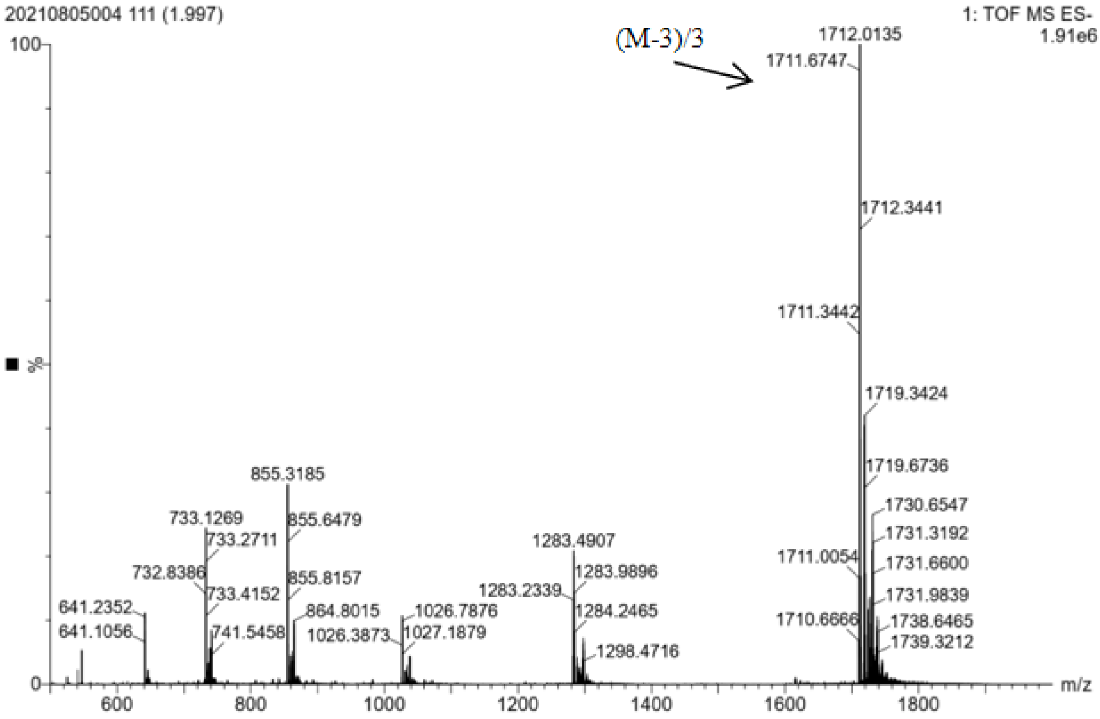

LC Trace and Mass of **D12**

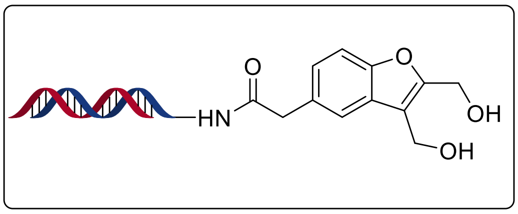

Following General Procedure **D12**
Yield: 100%
Exact mass: 5155.2879
Triply charged mass (M-3)/3, calculated 1717.4293; observed 1717.3475.

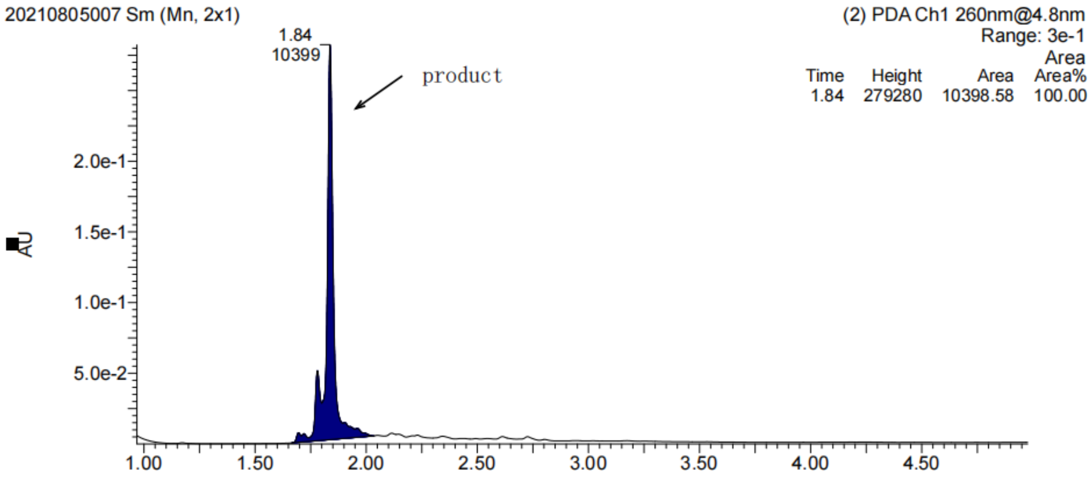

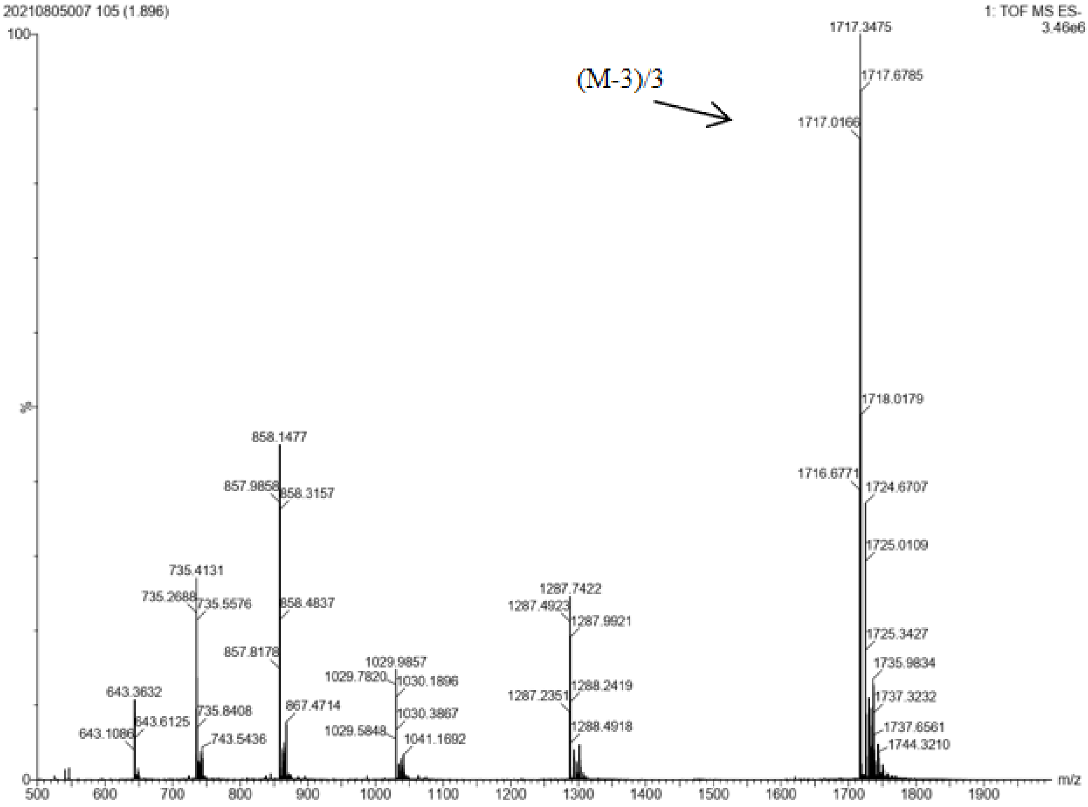

LC Trace and Mass of **D13**

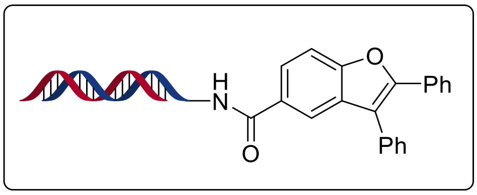

Following General Procedure **D13**

Yield: 70%

Exact mass: 5233.3242

Triply charged mass (M-3)/3, calculated 1743.4414; observed 1743.3289.

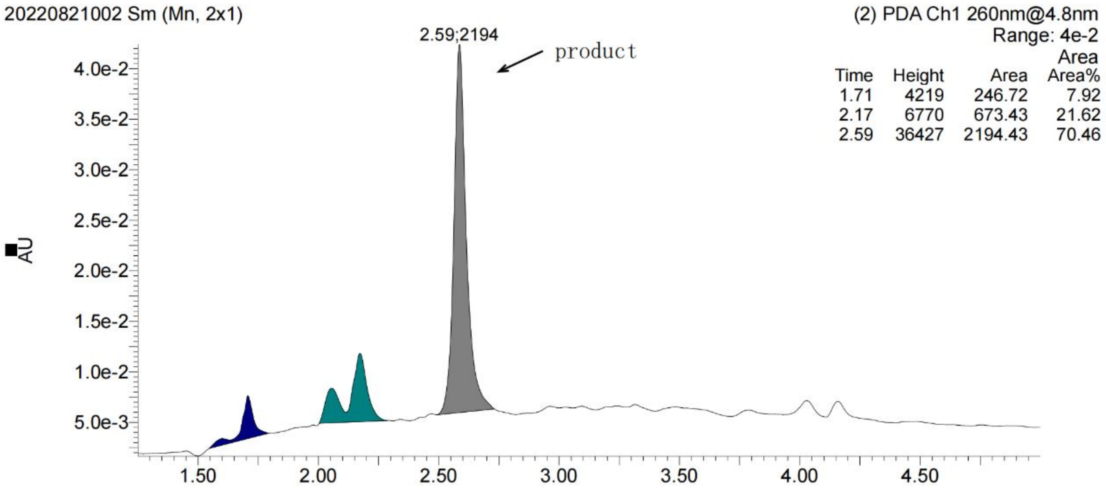

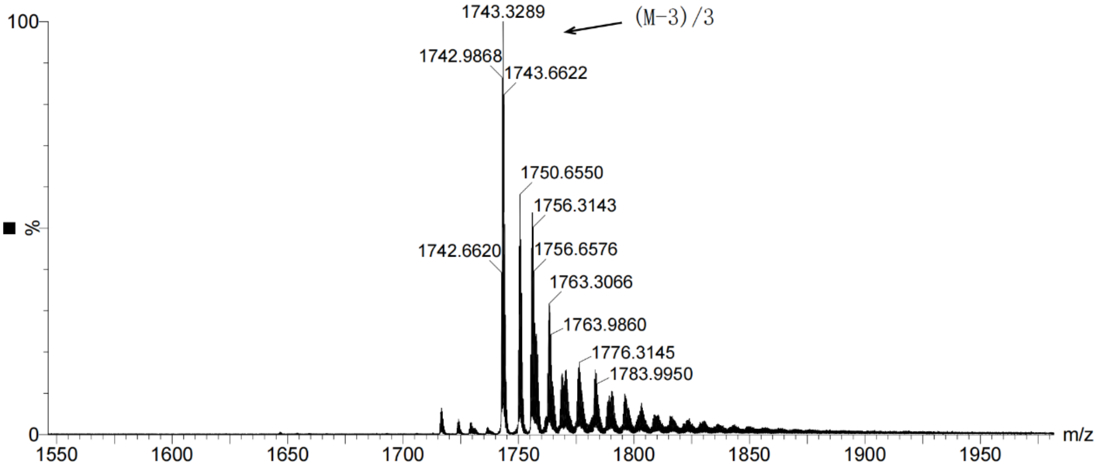

LC Trace and Mass of **D14**

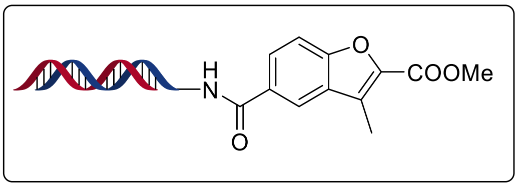

Following General Procedure **D14**

Yield: 41%

Exact mass: 5153.2827

Triply charged mass (M-3)/3, calculated 1716.7609; observed 1716.6541.

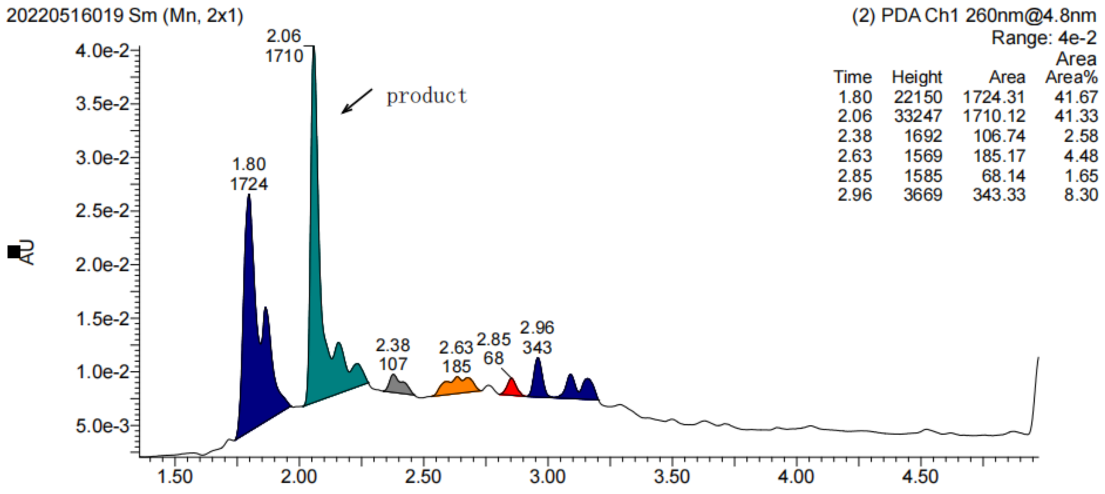

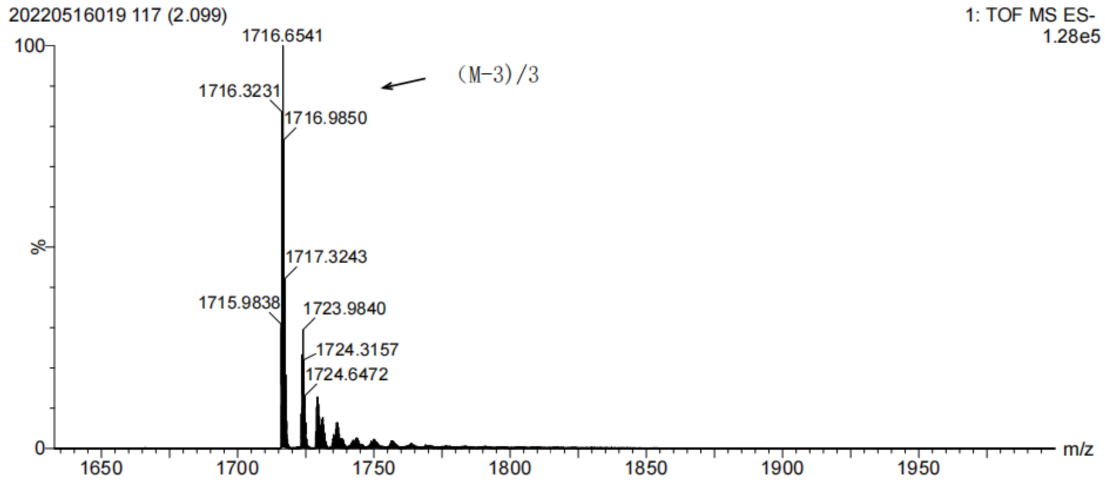

LC Trace and Mass of **D15**

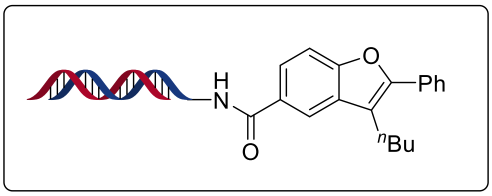

Following General Procedure **D15**

Yield: 21%

Exact mass: 5213.3556

Triply charged mass (M-3)/3, calculated 1736.7852; observed 1736.6936.

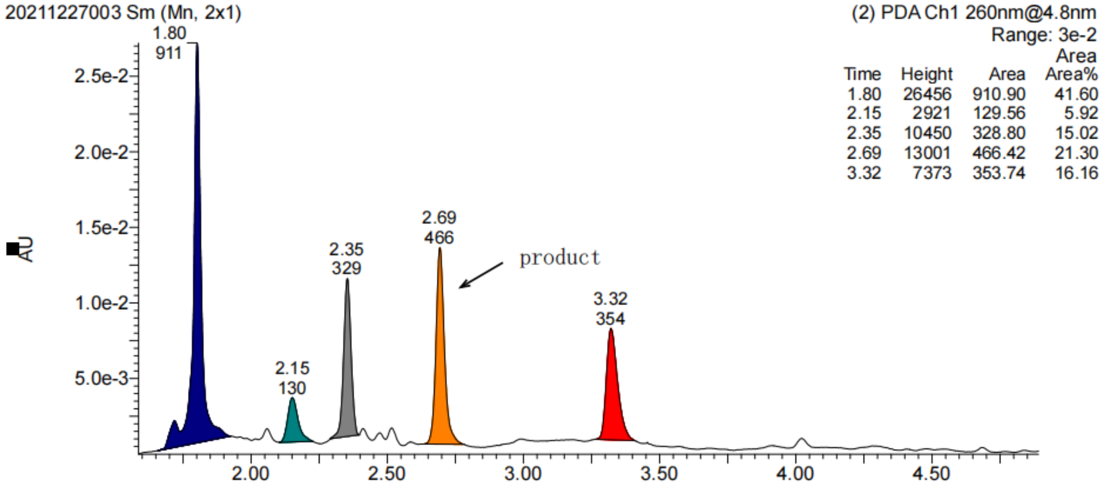

Time	Height	Area	Area Area%
1.80	26456	910.90	41.60
2.15	2921	129.56	5.92
2.35	10450	328.80	15.02
2.69	13001	466.42	21.30
3.32	7373	353.74	16.16

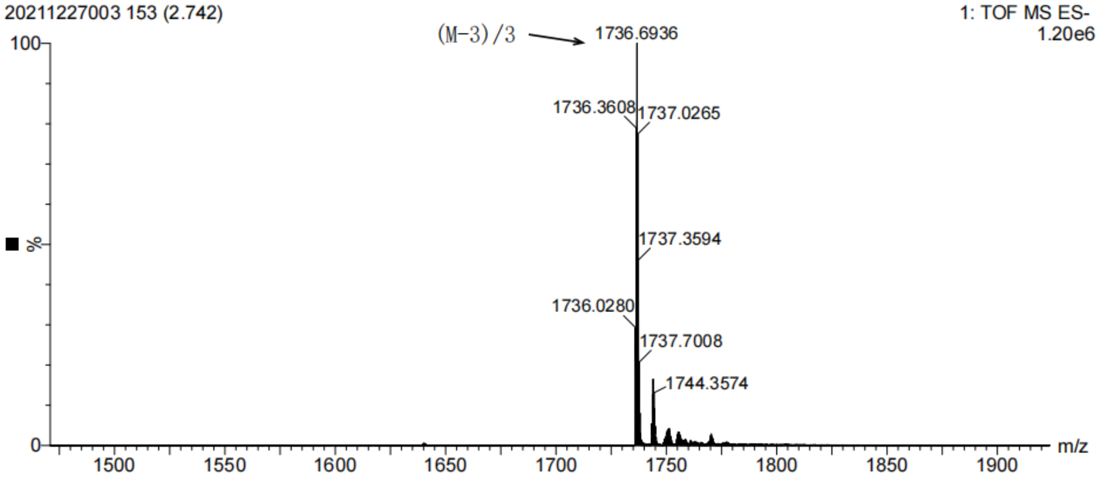

LC Trace and Mass of **D16**

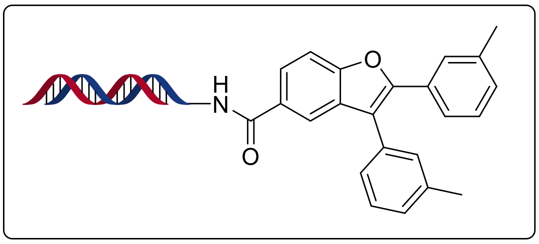

Following General Procedure **D16**

Yield: 32%

Exact mass: 5261.3556

Triply charged mass (M-3)/3, calculated 1752.7852; observed 1752.6982.

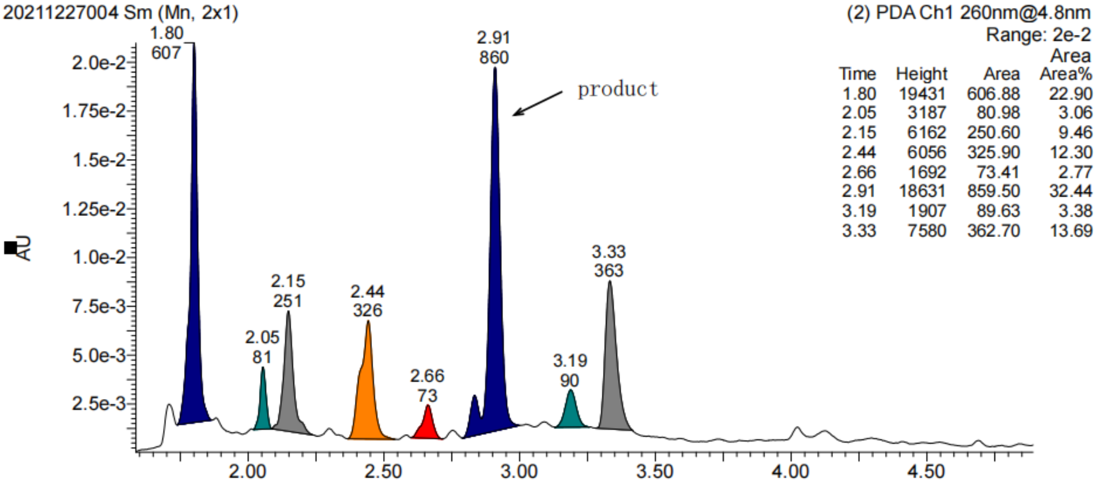

Time	Height	Area	Area%
1.80	19431	606.88	22.90
2.05	3187	80.98	3.06
2.15	6162	250.60	9.46
2.44	6056	325.90	12.30
2.66	1692	73.41	2.77
2.91	18631	859.50	32.44
3.19	1907	89.63	3.38
3.33	7580	362.70	13.69

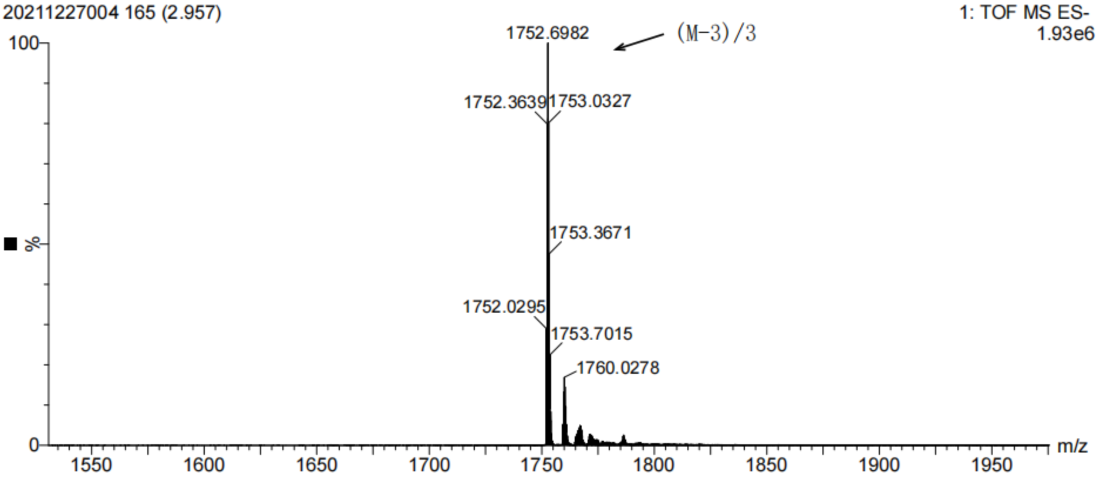

LC Trace and Mass of **D17**

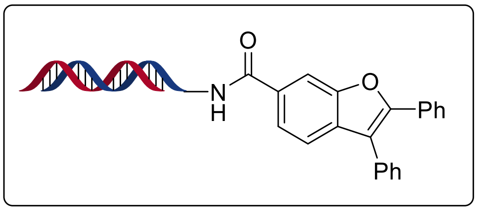

Following General Procedure **D17**
Yield: 78%
Exact mass: 5233.3242
Triply charged mass (M-3)/3, calculated 1743.4414; observed 1743.3412.

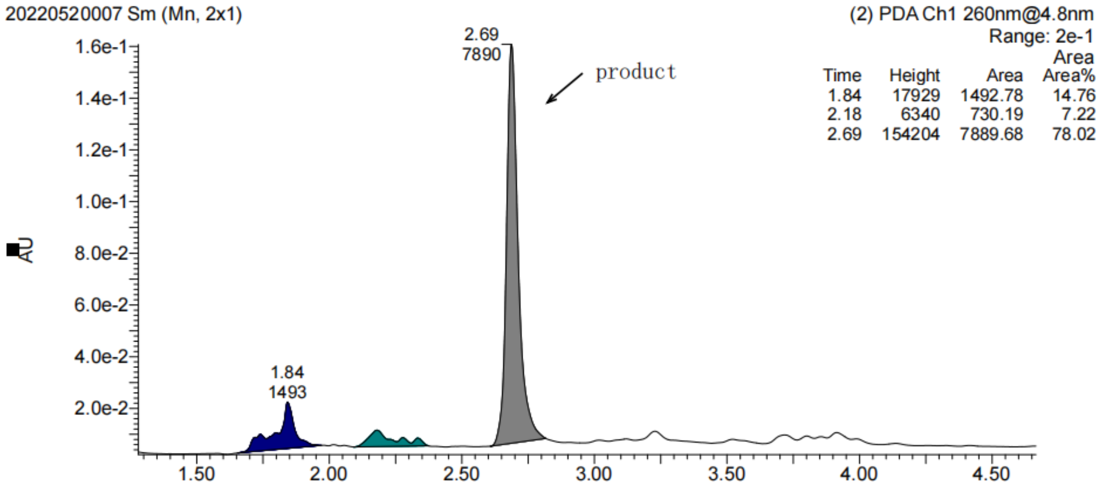

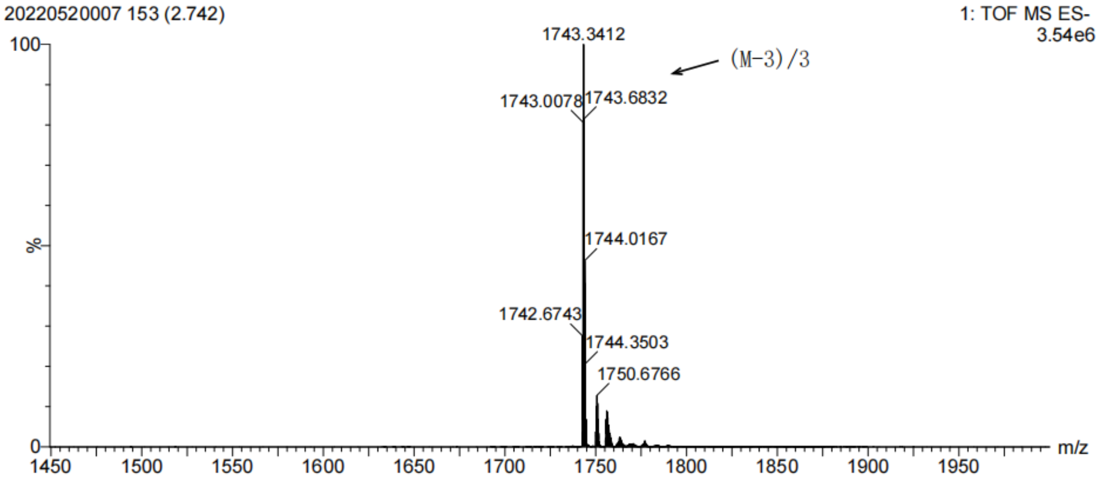

LC Trace and Mass of **D18**

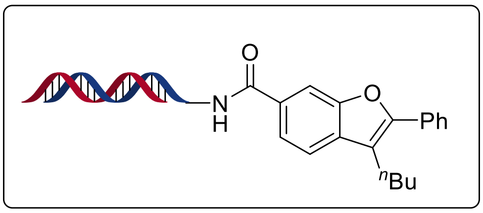

Following General Procedure **D18**
Yield: 59%
Exact mass: 5213.3556
Triply charged mass (M-3)/3, calculated 1736.7852; observed 1736.6868.

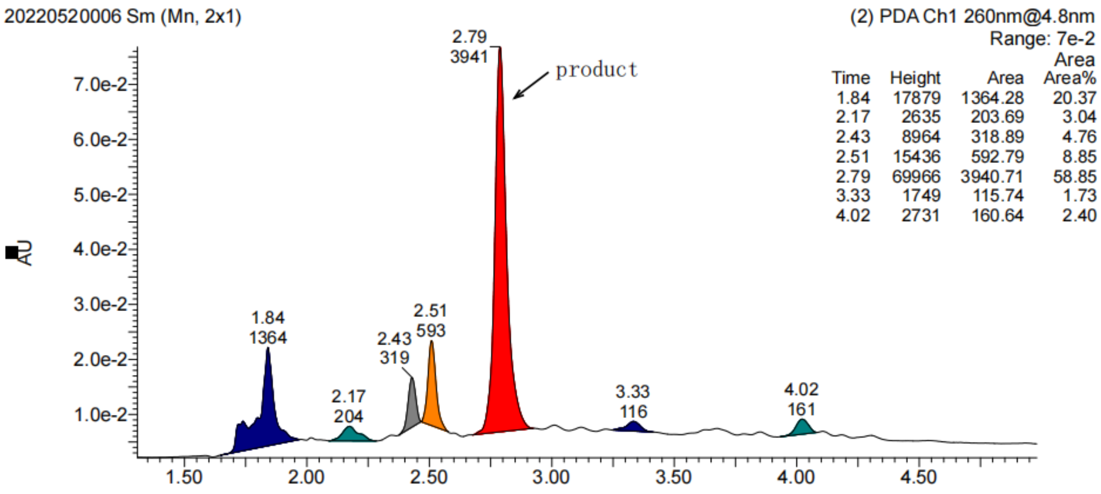

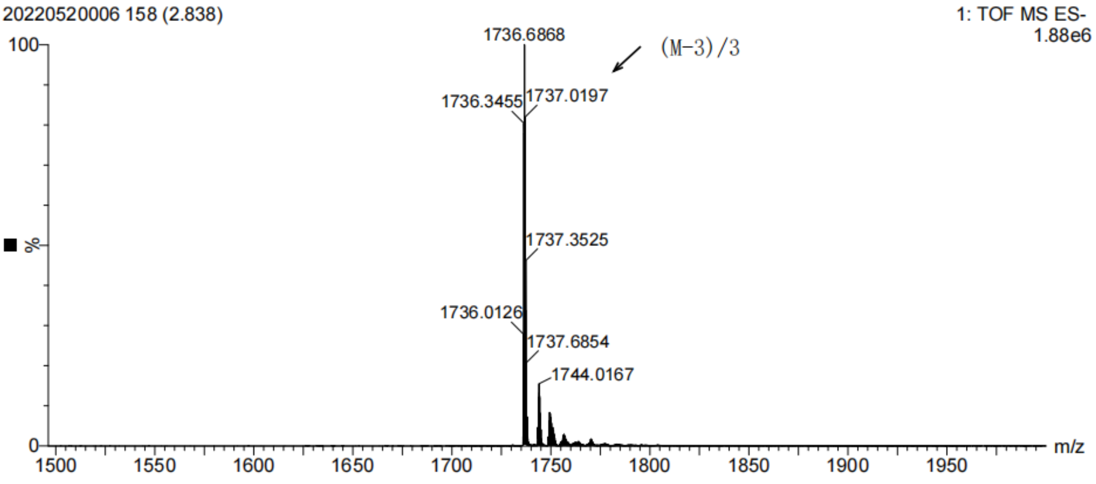

LC Trace and Mass of **D19**

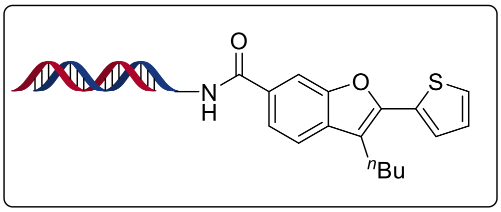

Following General Procedure **D19**
Yield: 31%
Exact mass: 5219.3121
Triply charged mass (M-3)/3, calculated 1738.7707; observed 1738.6007.

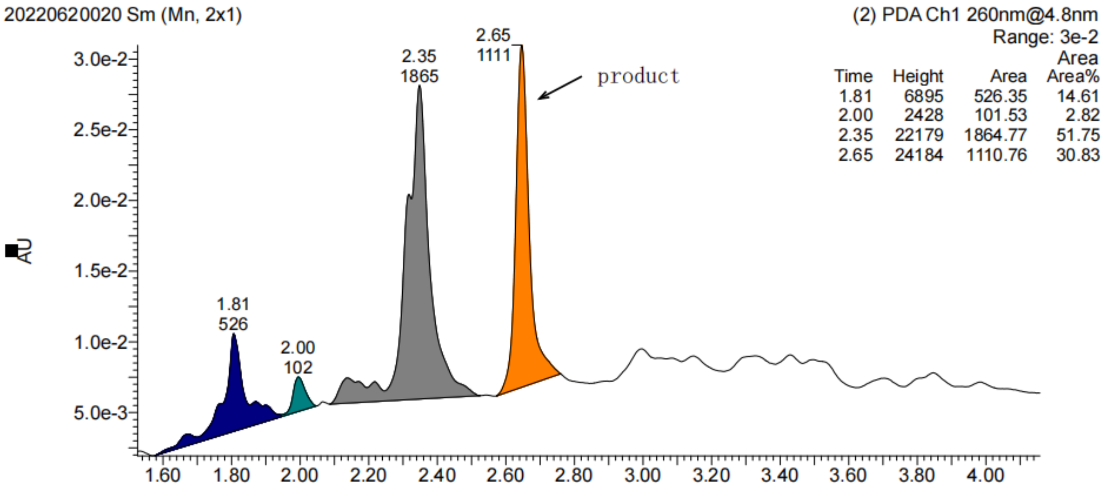

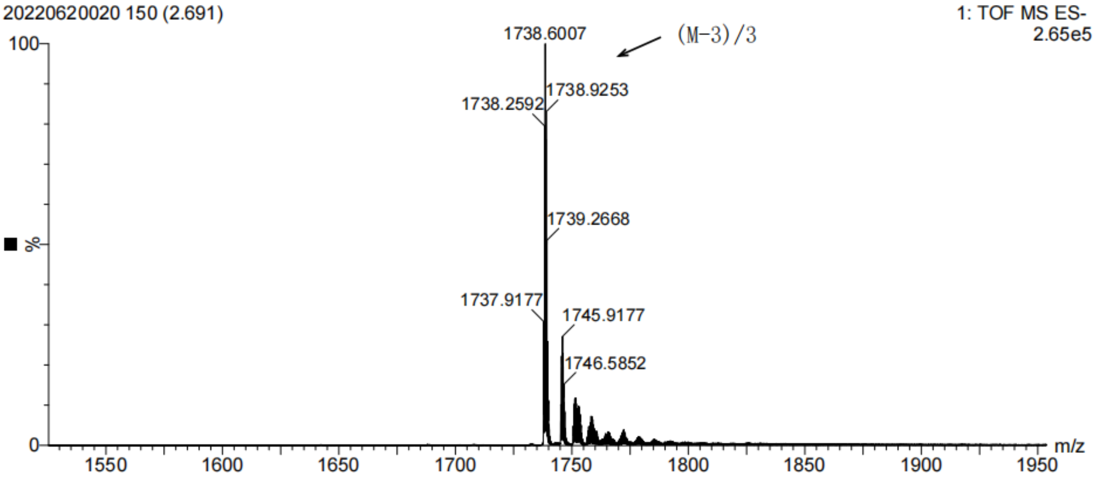

LC Trace and Mass of **D20**

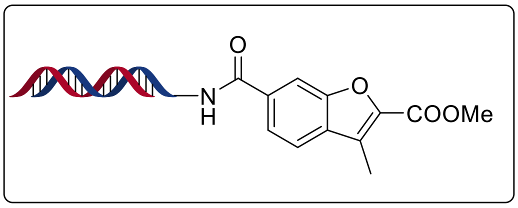

Following General Procedure **D20**
Yield: 80%
Exact mass: 5153.2827
Triply charged mass (M-3)/3, calculated 1716.7609; observed 1716.6555.

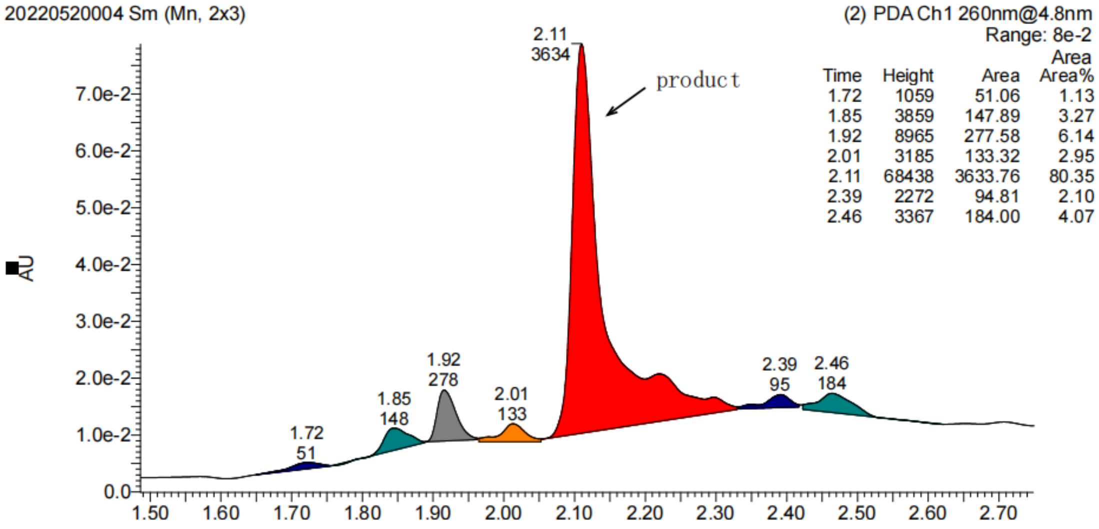

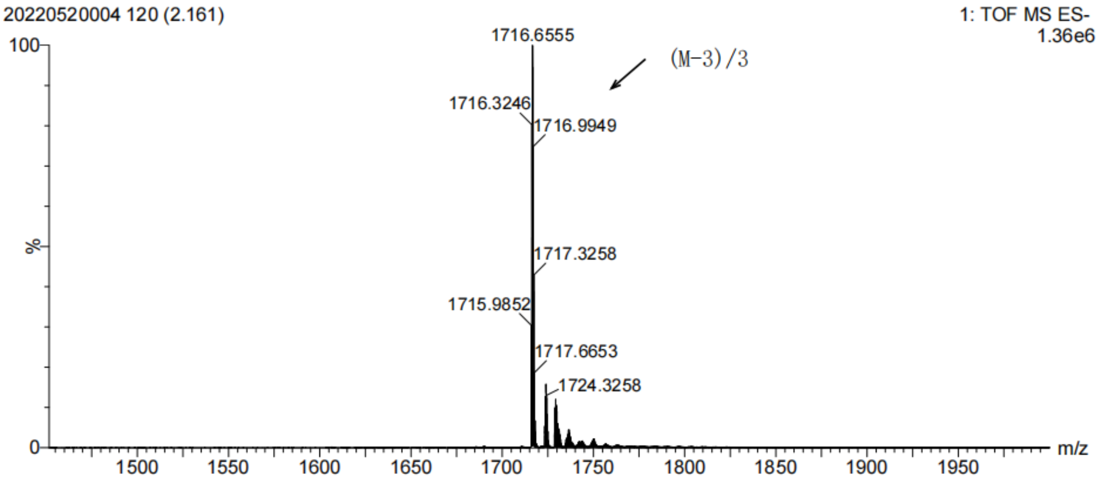

LC Trace and Mass of **D21**

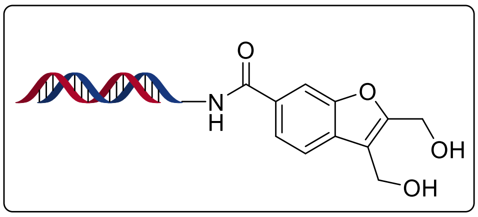

Following General Procedure **D21**
Yield: 94%
Exact mass: 5141.2827
Triply charged mass (M-3)/3, calculated 1712.7609; observed 1712.6615.

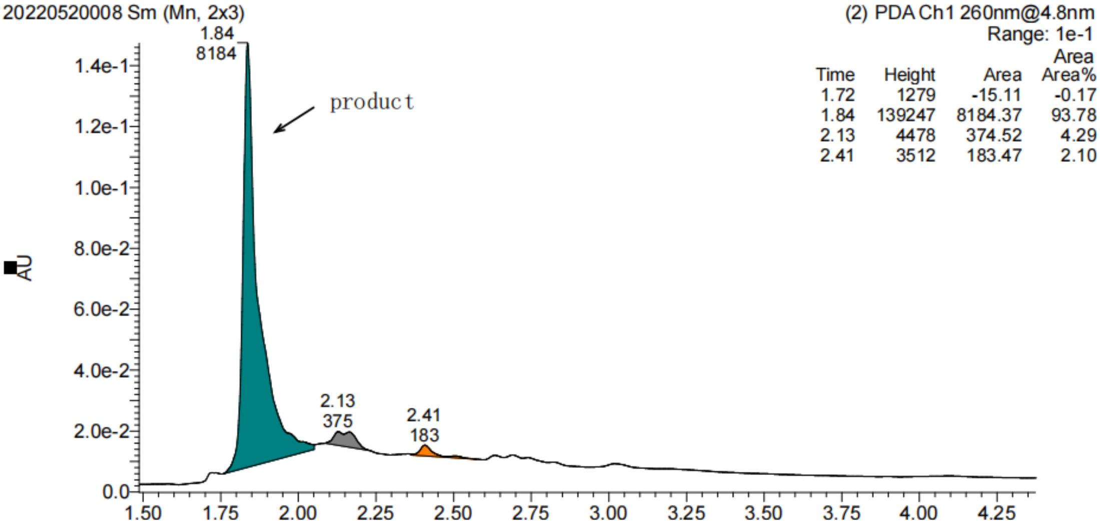

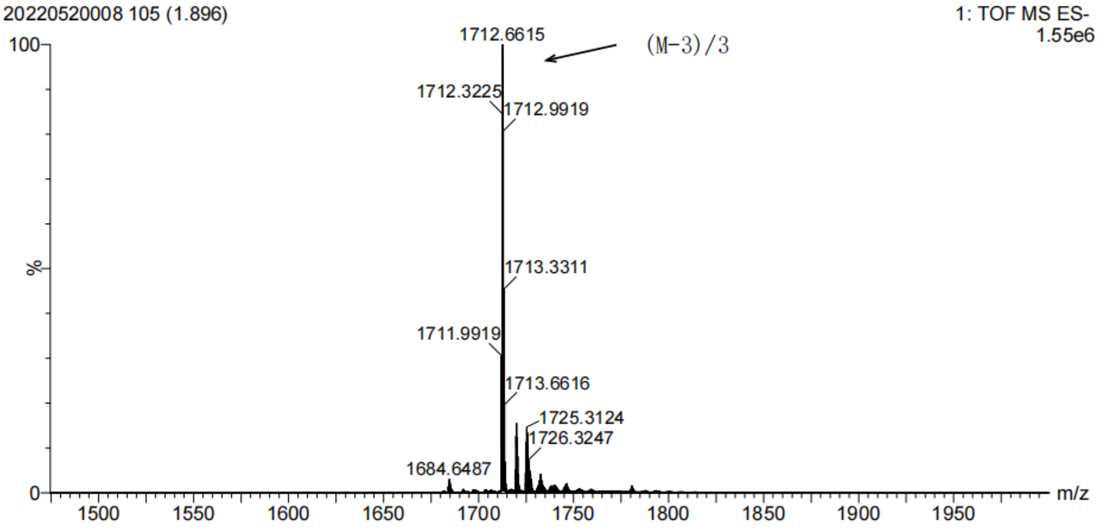

LC Trace and Mass of **H1**

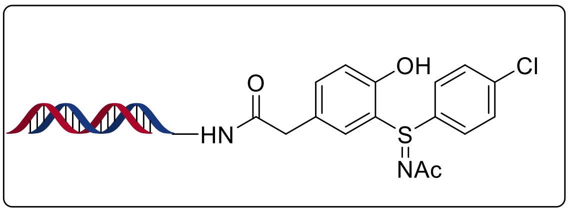

Following General Procedure **H1**
Yield: 40%
Exact mass: 5270.2632
Triply charged mass (M-3)/3, calculated 1755.7544; observed 1755.6473.

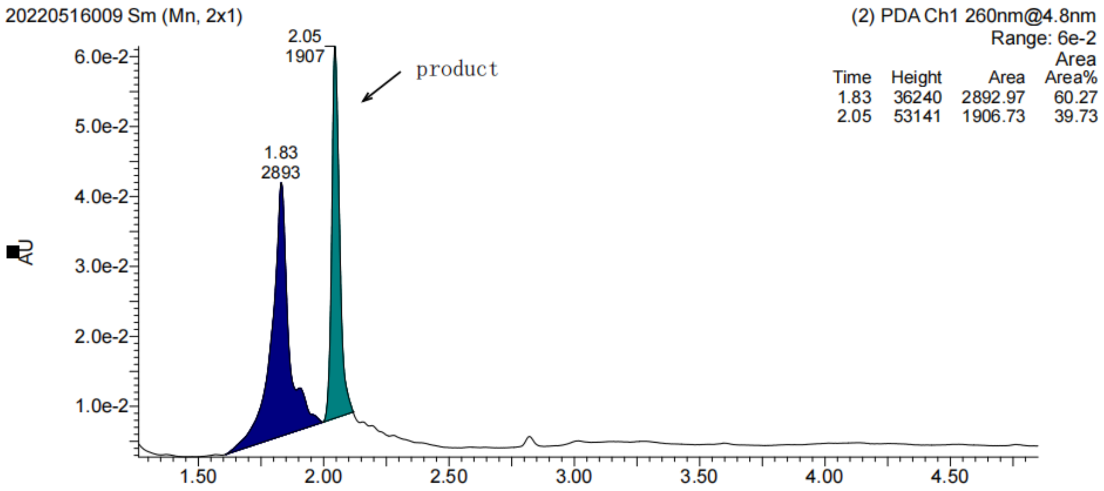

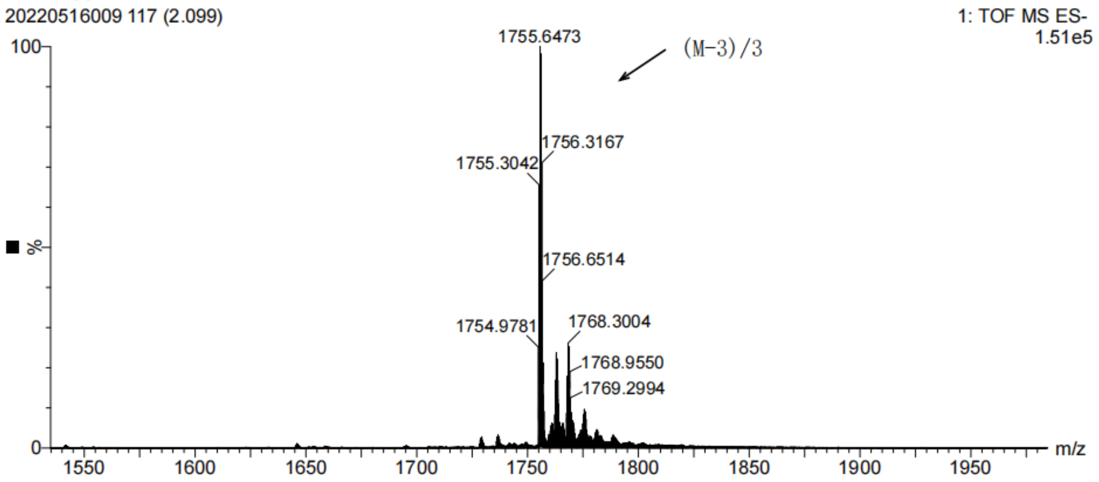

LC Trace and Mass of **H2**

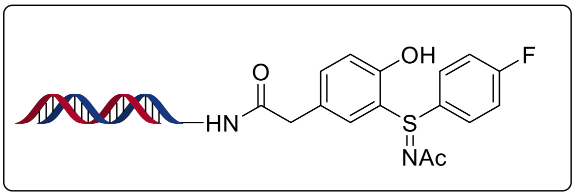

Following General Procedure **H2**

Yield: 80%

Exact mass: 5254.2918

Triply charged mass (M-3)/3, calculated 1750.4309; observed 1750.0967.

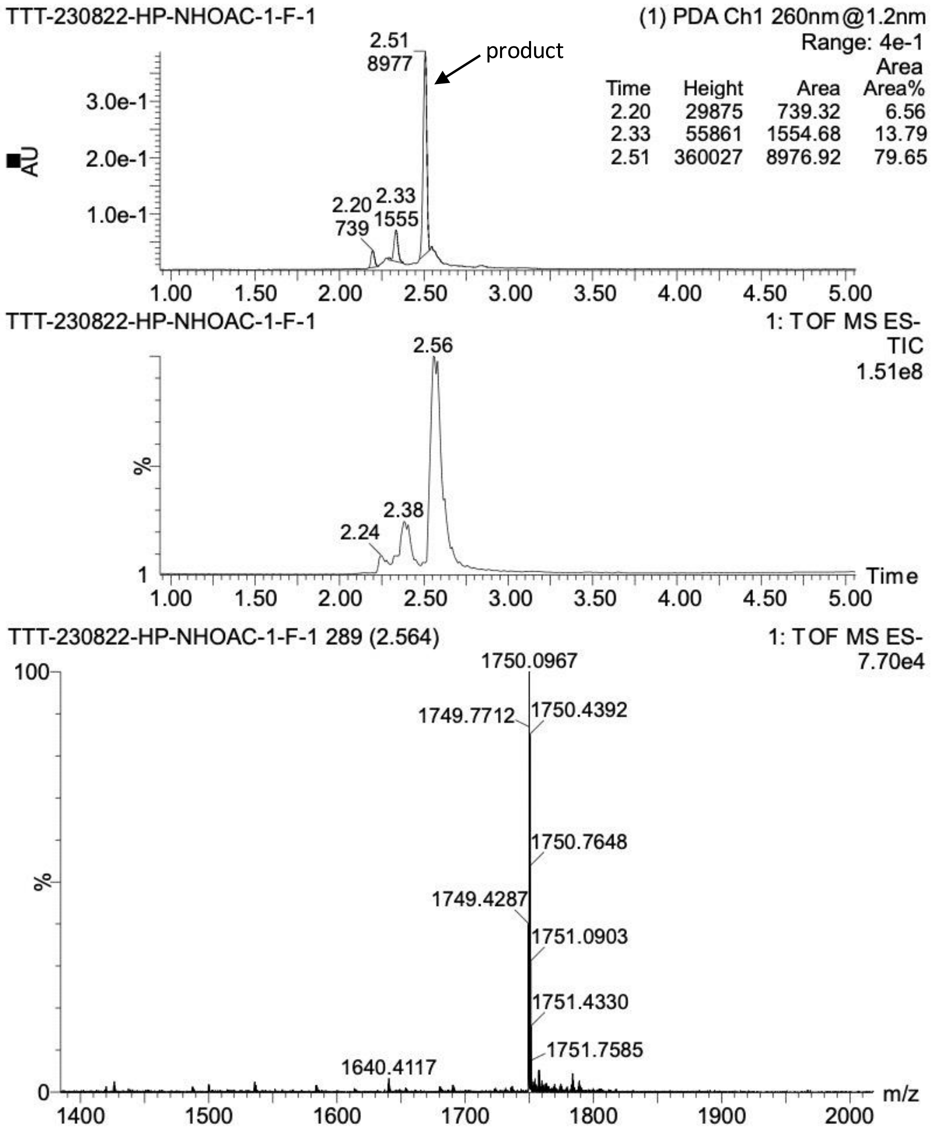

LC Trace and Mass of **H3**

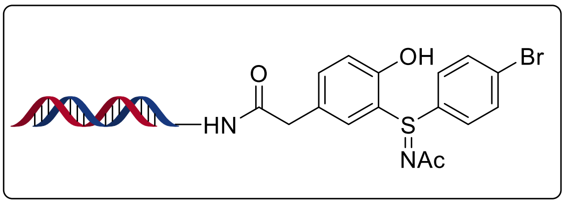

Following General Procedure **H3**

Yield: 45%

Exact mass: 5314.2126

Triply charged mass (M-3)/3, calculated 1770.4042; observed 1770.6348.

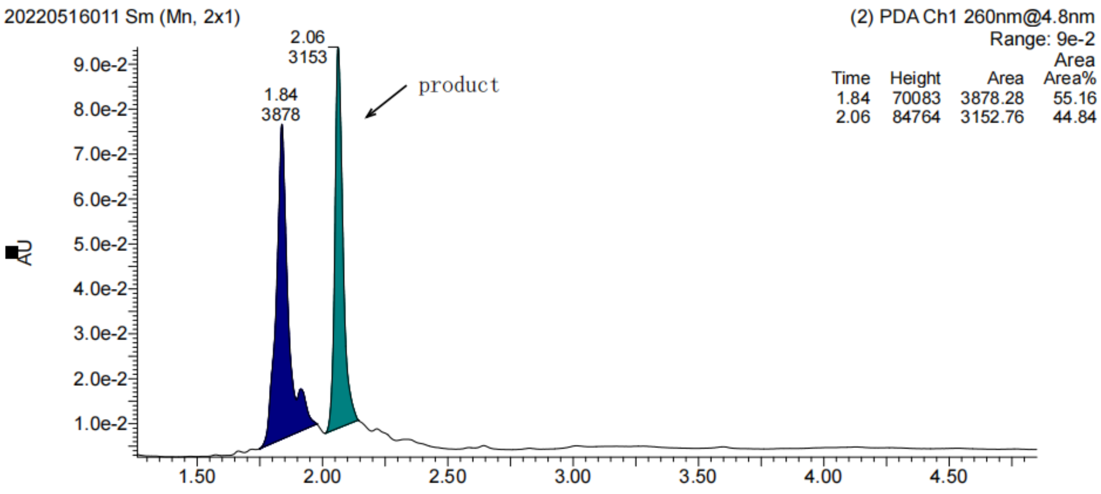

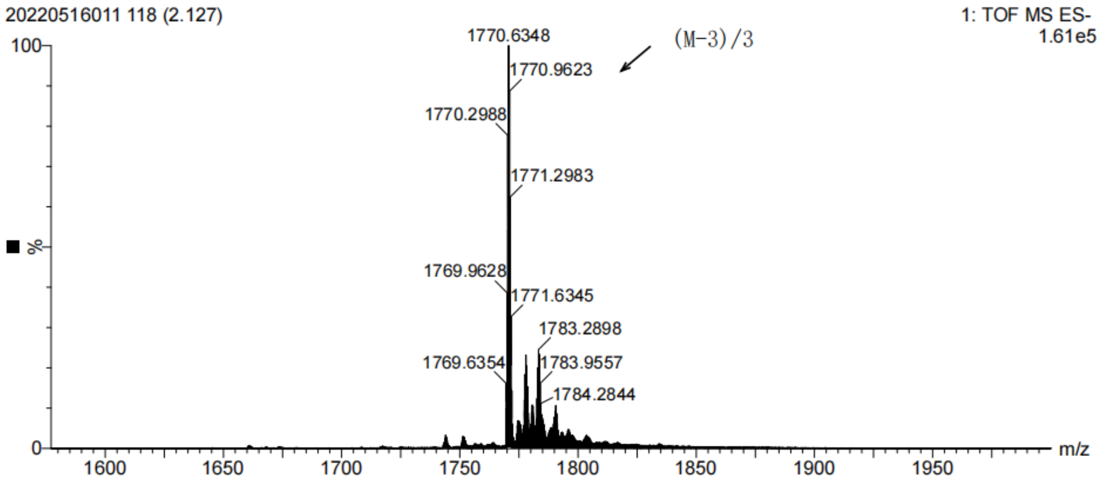

LC Trace and Mass of **H4**

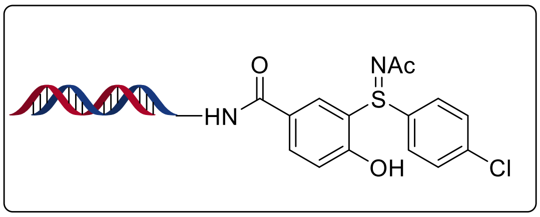

Following General Procedure **H4**

Yield: 32%

Exact mass: 5256.2475

Triply charged mass (M-3)/3, calculated 1751.0825; observed 1751.3268.

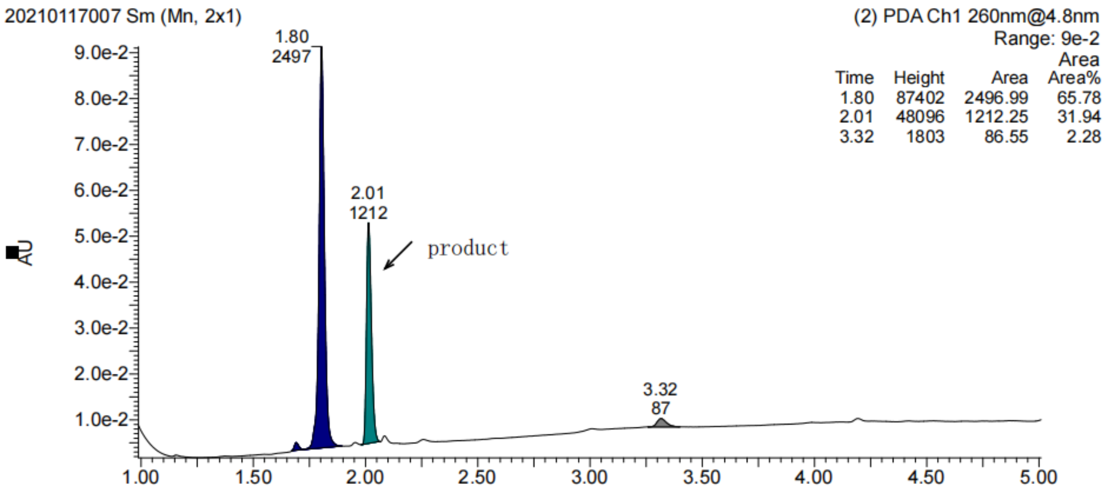

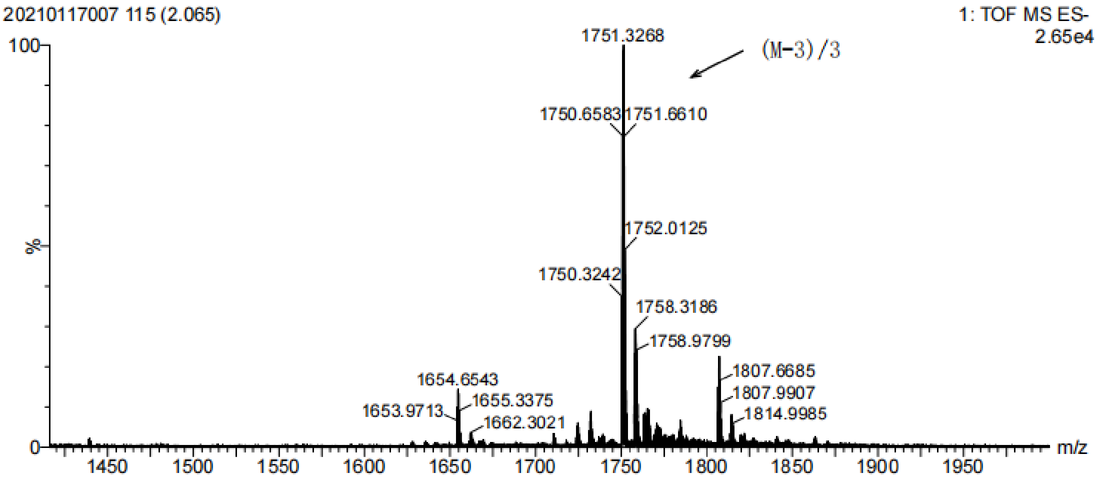

LC Trace and Mass of **H5**

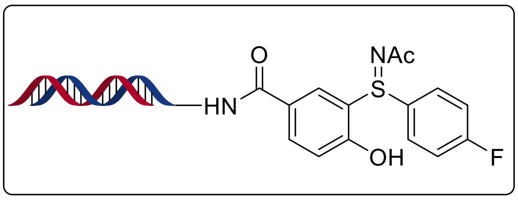

Following General Procedure **H5**

Yield: 42%

Exact mass: 5240.2770

Triply charged mass (M-3)/3, calculated 1745.7590; observed 1745.6664.

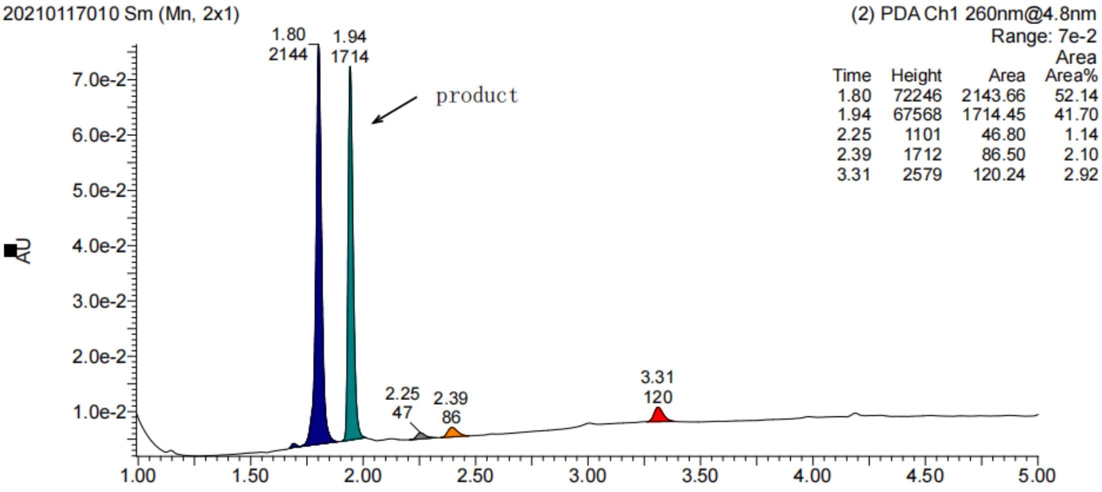

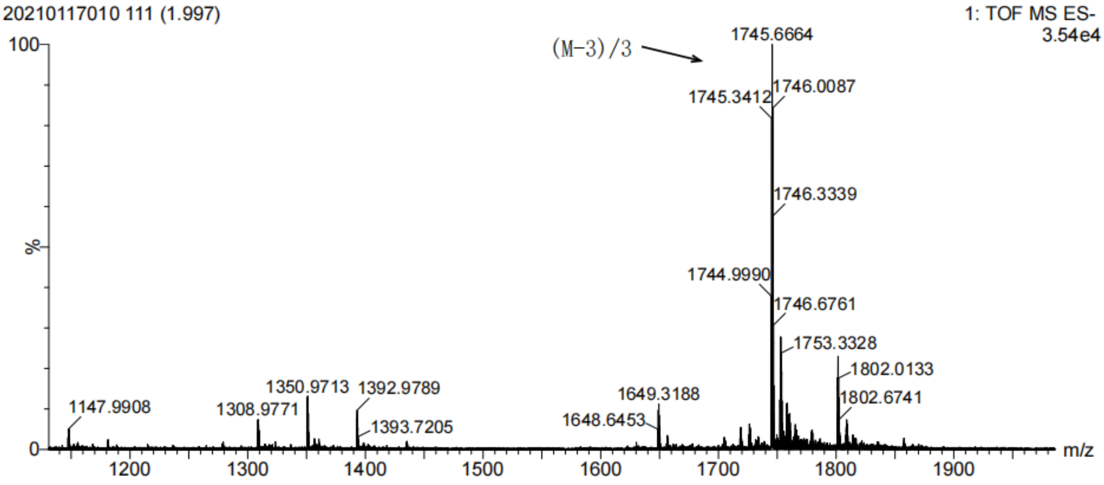

LC Trace and Mass of **H6**

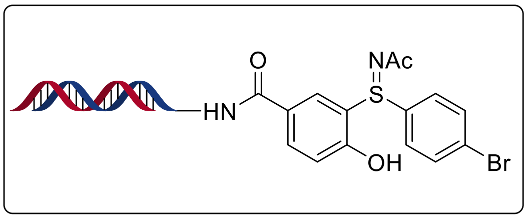

Following General Procedure **H6**
Yield: 27%
Exact mass: 5300.1969
Triply charged mass (M-3)/3, calculated 1765.7323; observed 1765.9868.

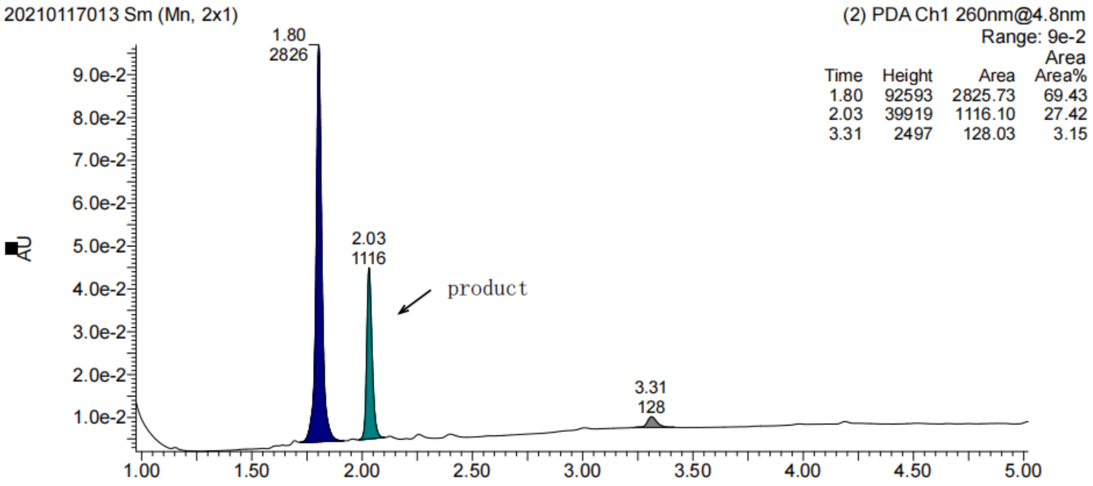

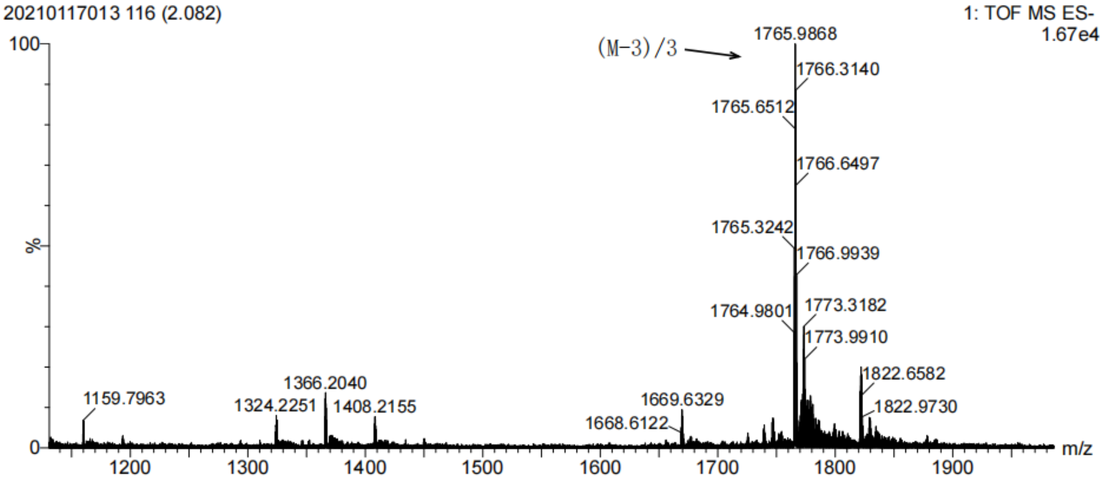

LC Trace and Mass of **H7**

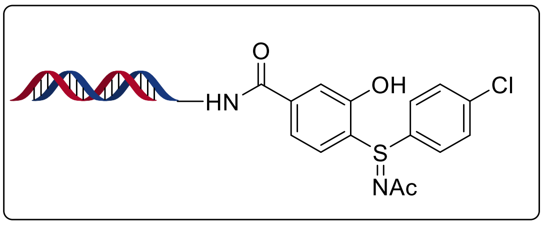

Following General Procedure **H7**
Yield: 24%
Exact mass: 5256.2475
Triply charged mass (M-3)/3, calculated 1751.0825; observed 1751.3083.

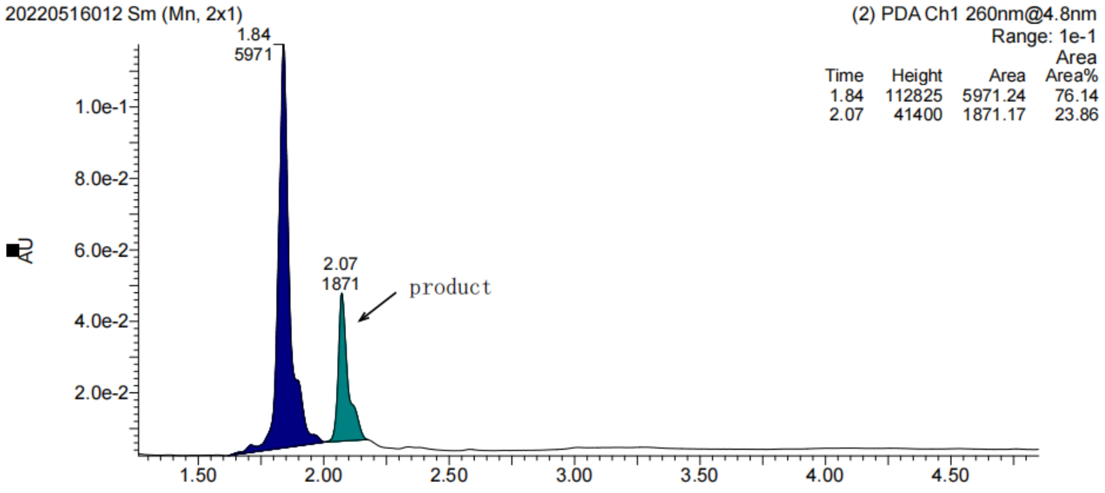

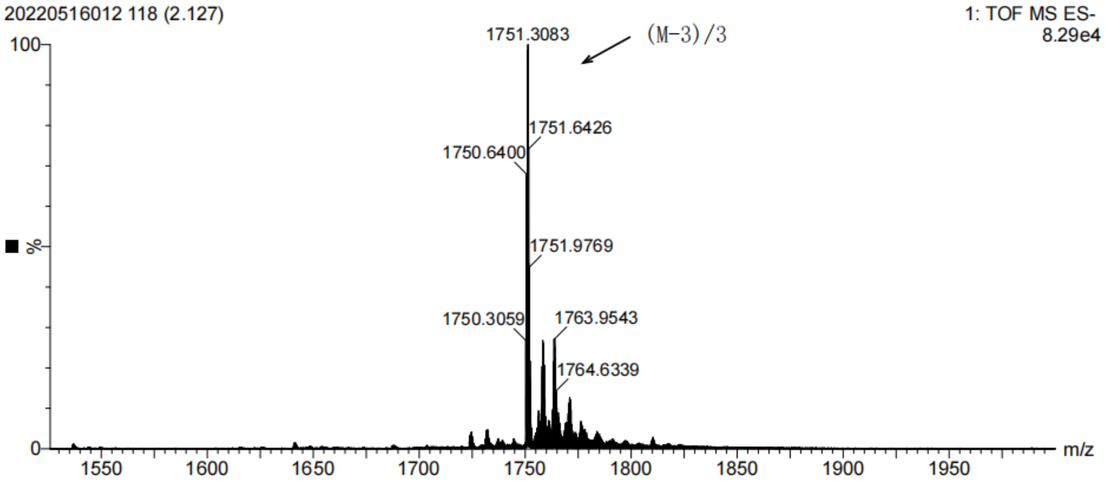

LC Trace and Mass of **H8**

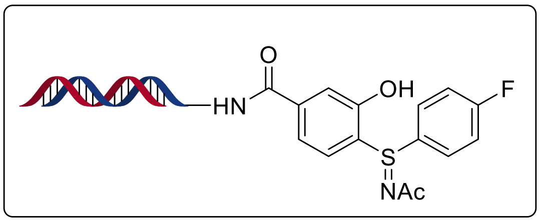

Following General Procedure **H8**
Yield: 35%
Exact mass: 5240.2770
Triply charged mass (M-3)/3, calculated 1745.7590; observed 1745.6483.

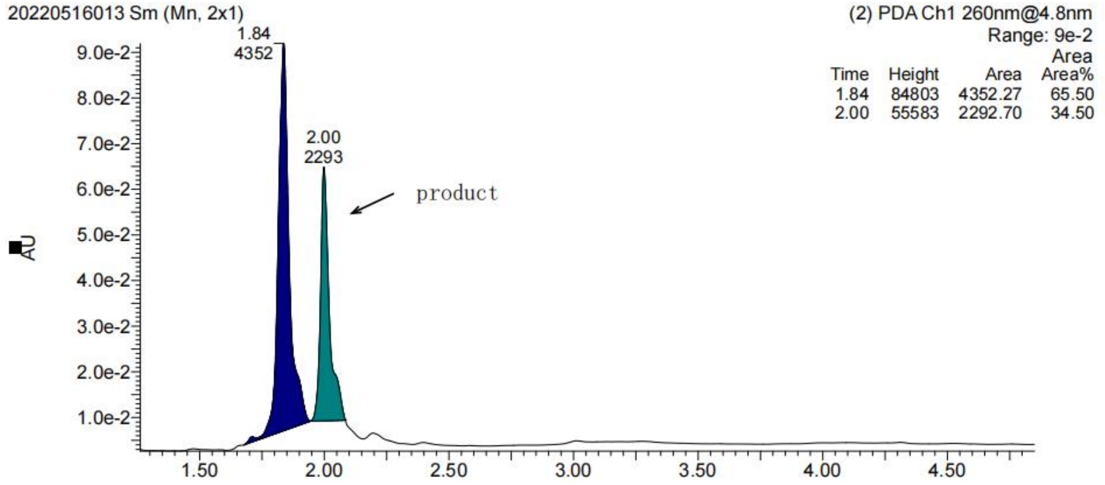

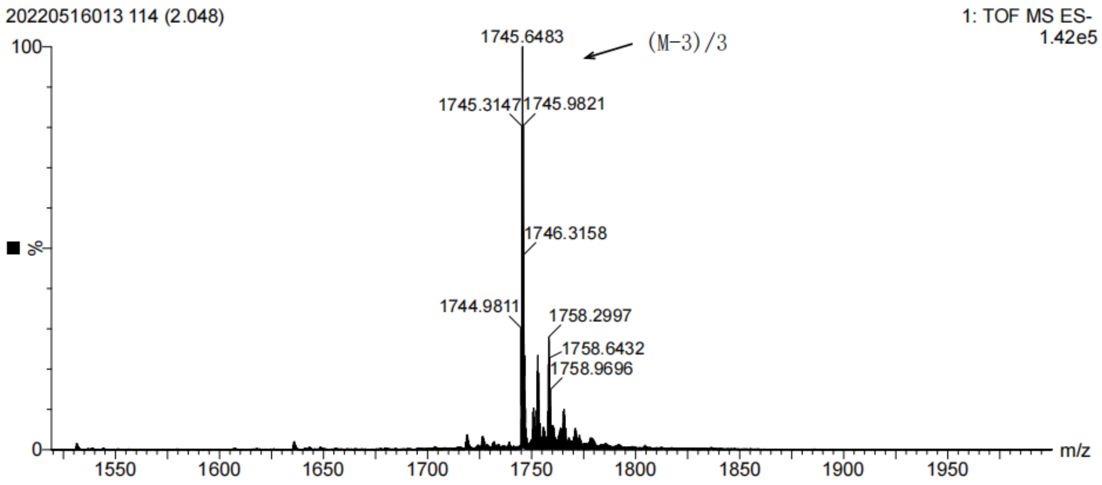

LC Trace and Mass of **H9**

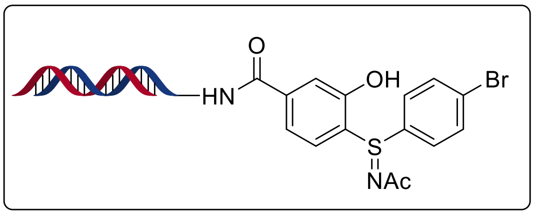

Following General Procedure **H9**
Yield: 21%
Exact mass: 5300.1969
Triply charged mass (M-3)/3, calculated 1765.7323; observed 1765.9589.

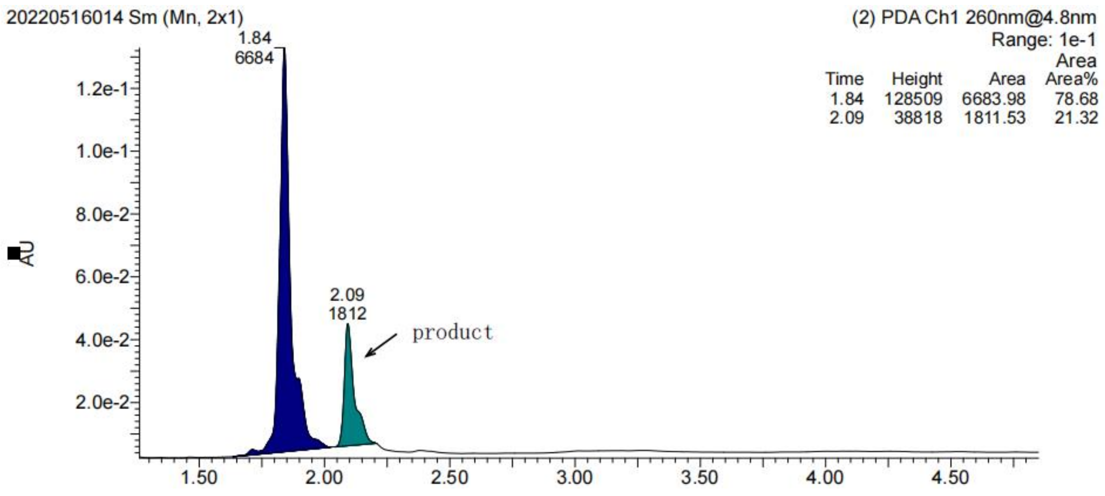

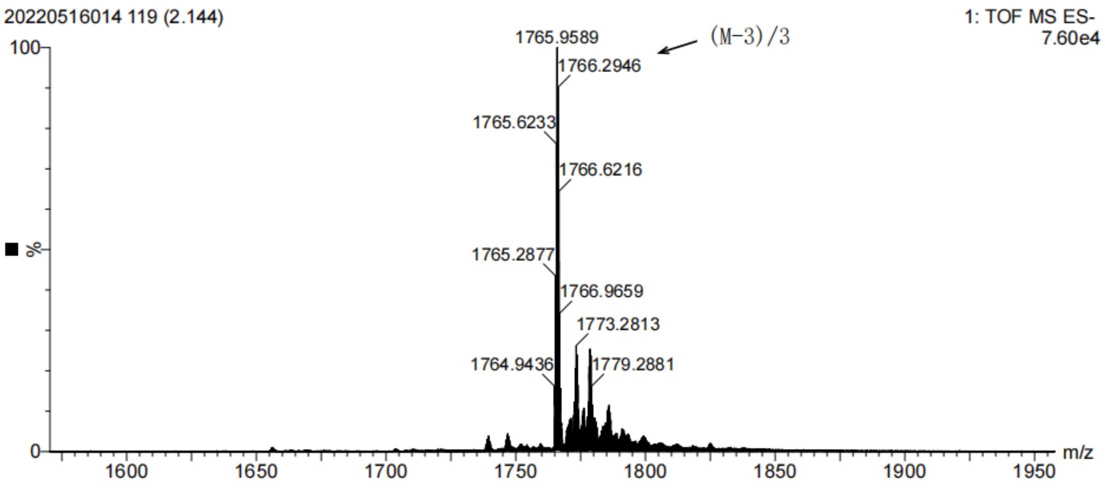

LC Trace and Mass of **C27**

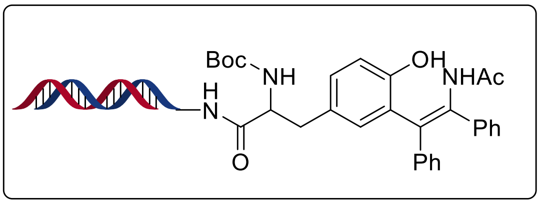

Following General Procedure **C27**

Yield: 64%

Exact mass: 5435.4561

Triply charged mass (M-3)/3, calculated 1810.8187; observed 1810.7258.

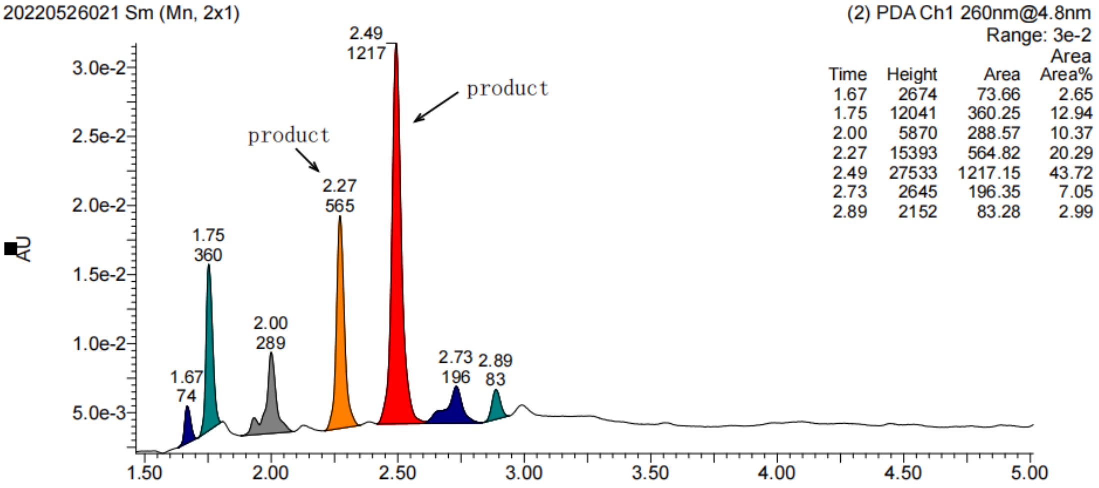

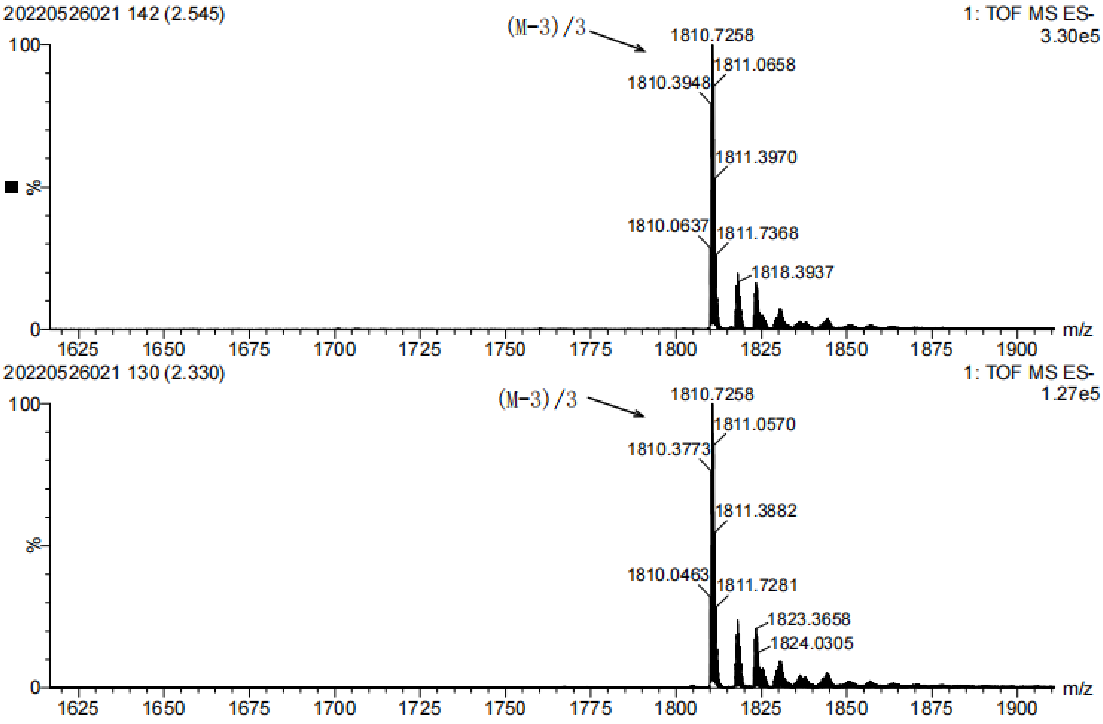

LC Trace and Mass of **D22**

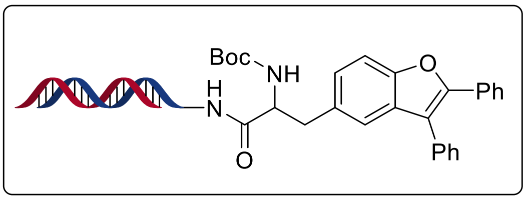

Following General Procedure **D22**

Yield: 69%

Exact mass: 5376.7560

Triply charged mass (M-3)/3, calculated 1791.2520; observed 1791.0424.

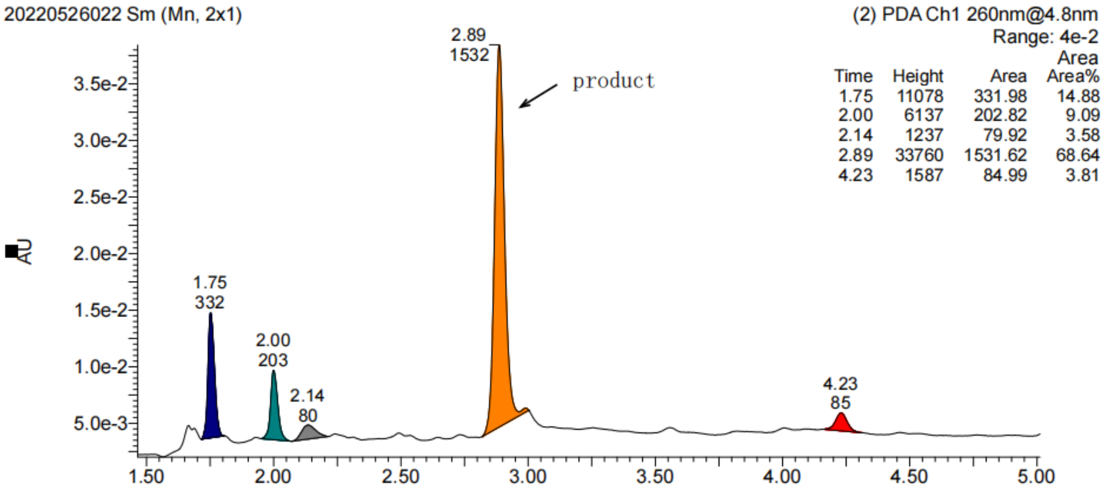

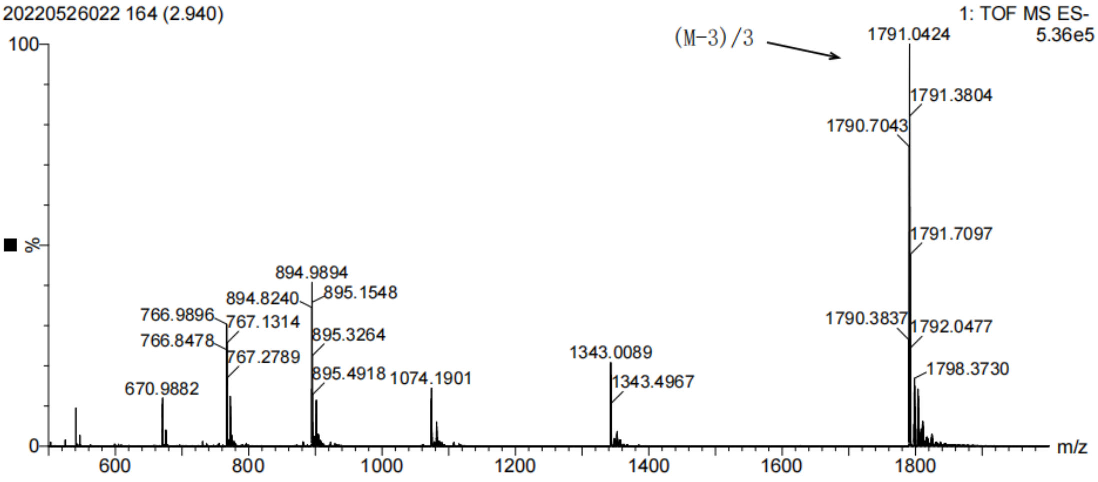

LC Trace and Mass of **C28**

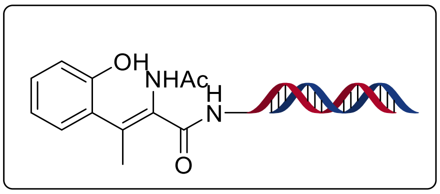

Following General Procedure **C28**

Yield: 67%

Exact mass: 5154.3144

Triply charged mass (M-3)/3, calculated 1717.1048; observed 1717.0095.

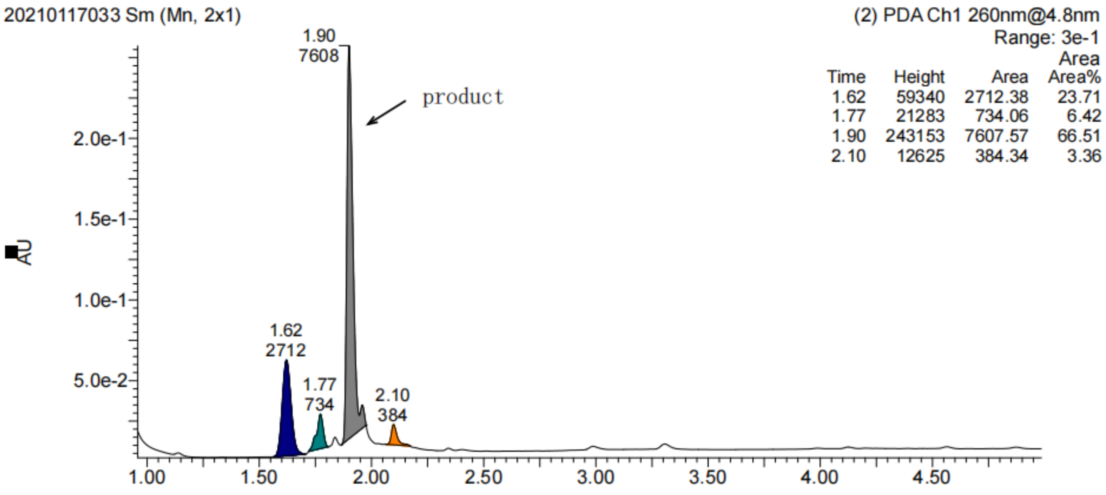

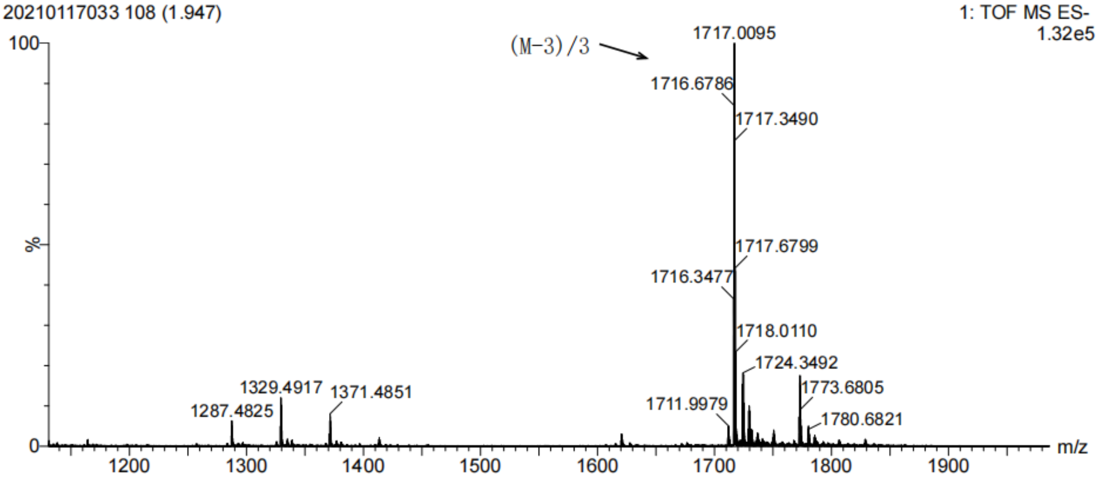

LC Trace and Mass of **D23**

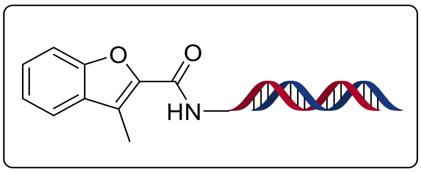

Following General Procedure **D23**
Yield: 33%
Exact mass: 5095.2774
Triply charged mass (M-3)/3, calculated 1697.4258; observed 1697.3447.

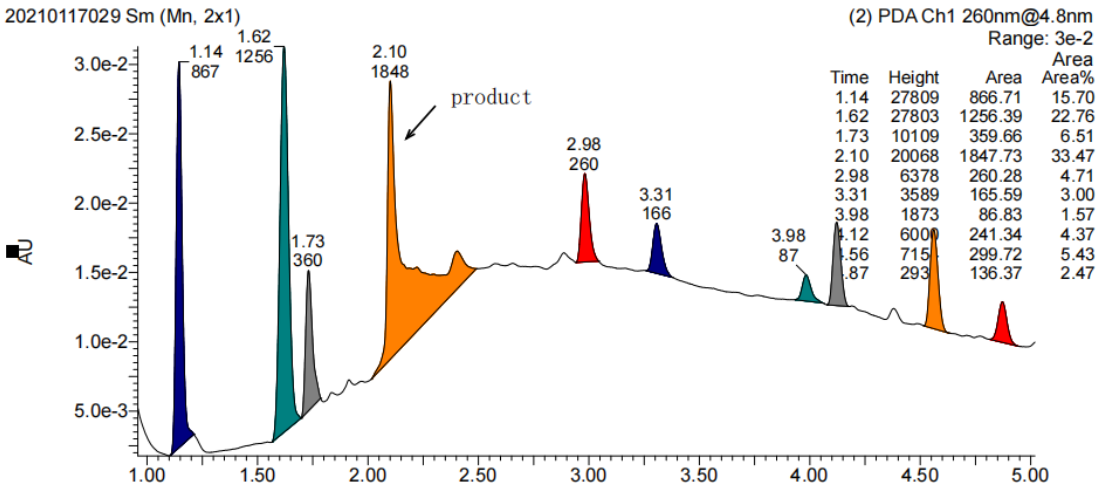

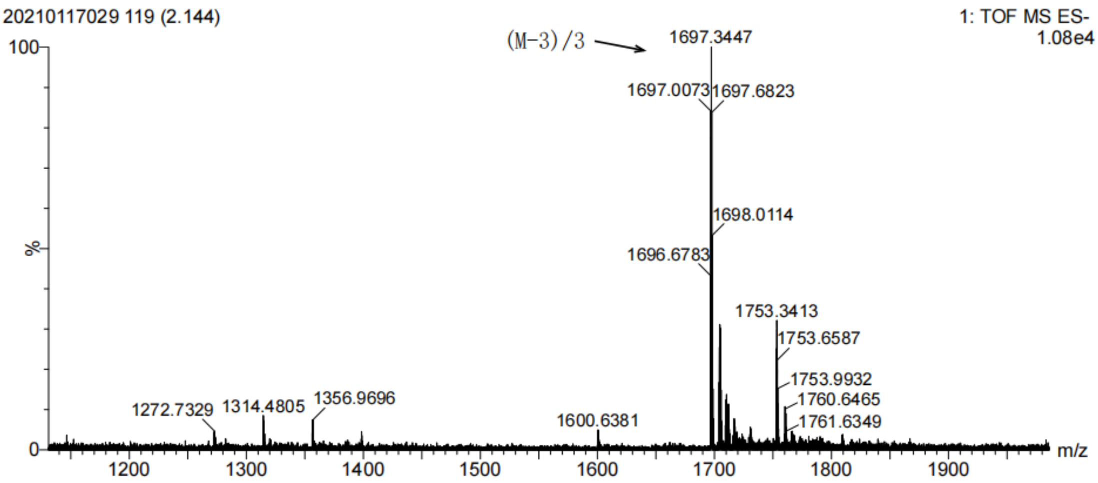

LC Trace and Mass of **I1**

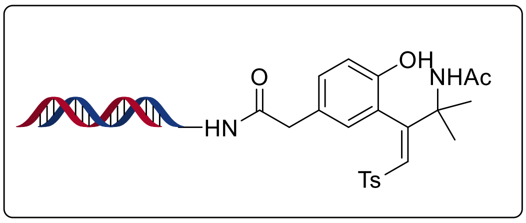

Following General Procedure **I1**
Yield: 34%
Exact mass: 5350.7331
Triply charged mass (M-3)/3, calculated 1782.5777; observed 1782.3677.

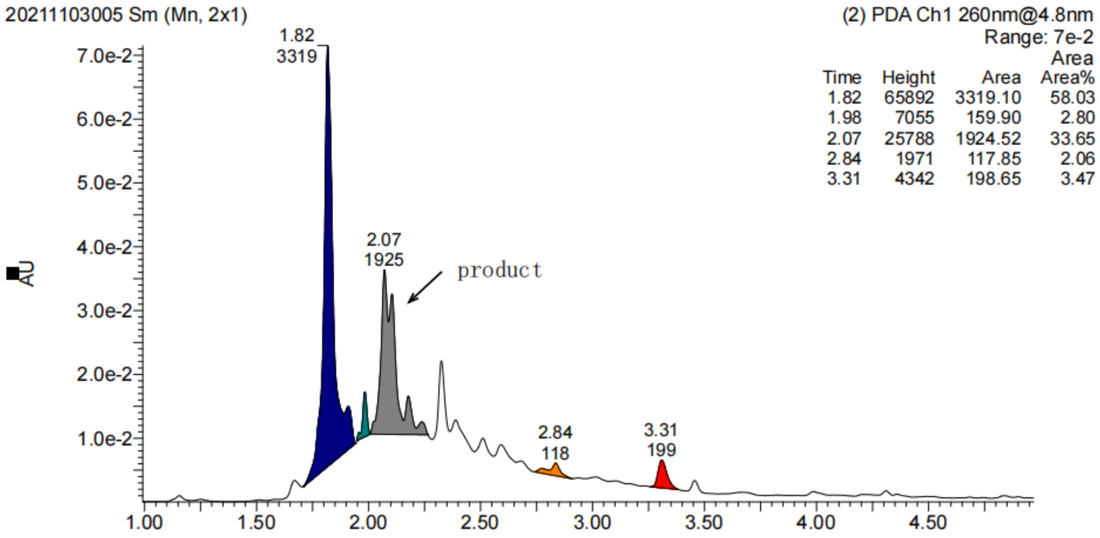

Time	Height	Area	Area Area%
1.82	65892	3319.10	58.03
1.98	7055	159.90	2.80
2.07	25788	1924.52	33.65
2.84	1971	117.85	2.06
3.31	4342	198.65	3.47

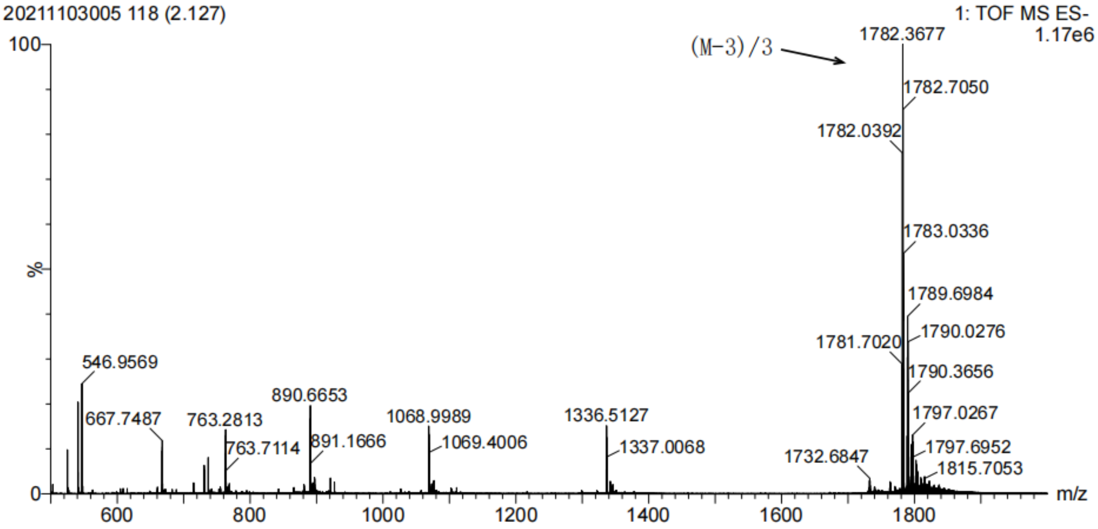

LC Trace and Mass of **I2**

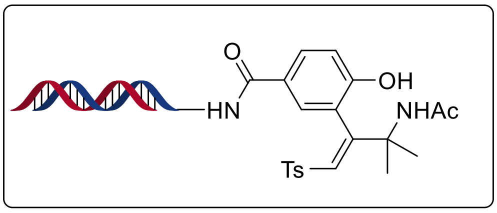

Following General Procedure **I2**
Yield: 56%
Exact mass: 5336.7060
Triply charged mass (M-3)/3, calculated 1777.9020; observed 1777.7020.

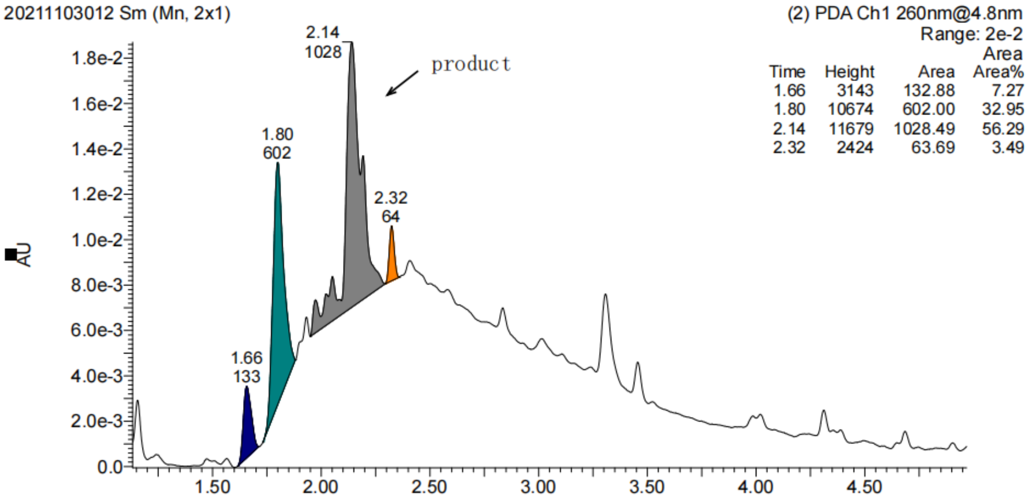

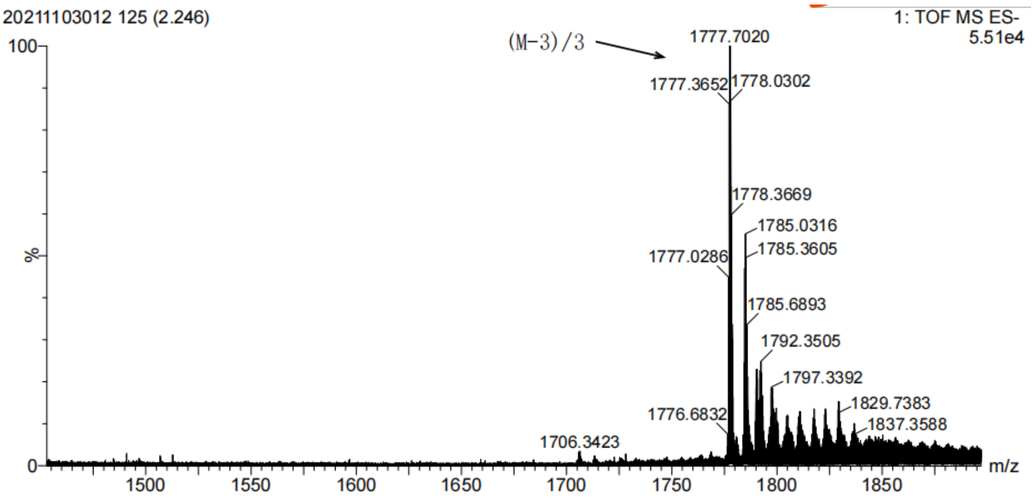

LC Trace and Mass of **J1**

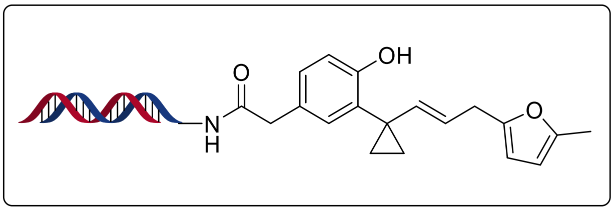

Following General Procedure **J1**
Yield: 58%
Exact mass: 5231.3661
Triply charged mass (M-3)/3, calculated 1742.7887; observed 1742.6807.

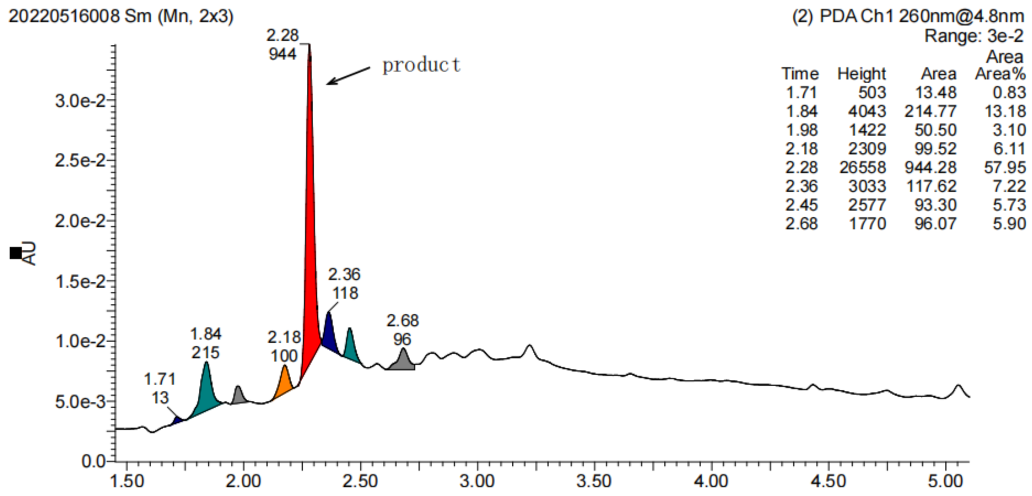

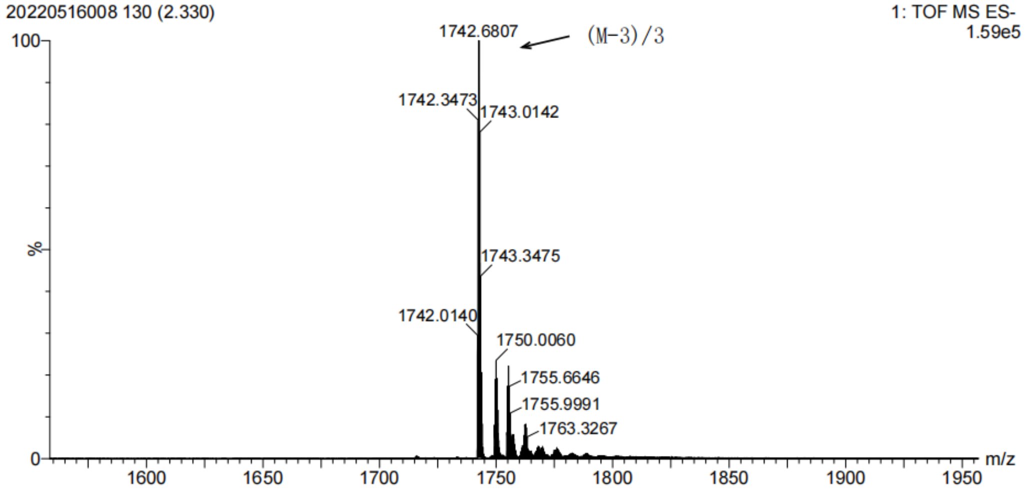

LC Trace and Mass of **J2**

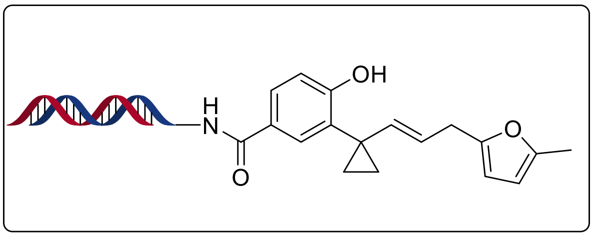

Following General Procedure **J2**
Yield: 37%
Exact mass: 5217.3504
Triply charged mass (M-3)/3, calculated 1738.1168; observed 1738.0098.

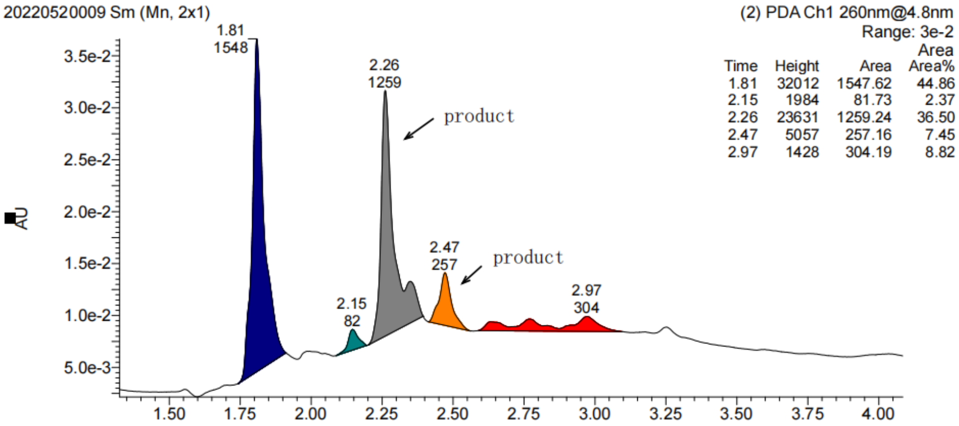

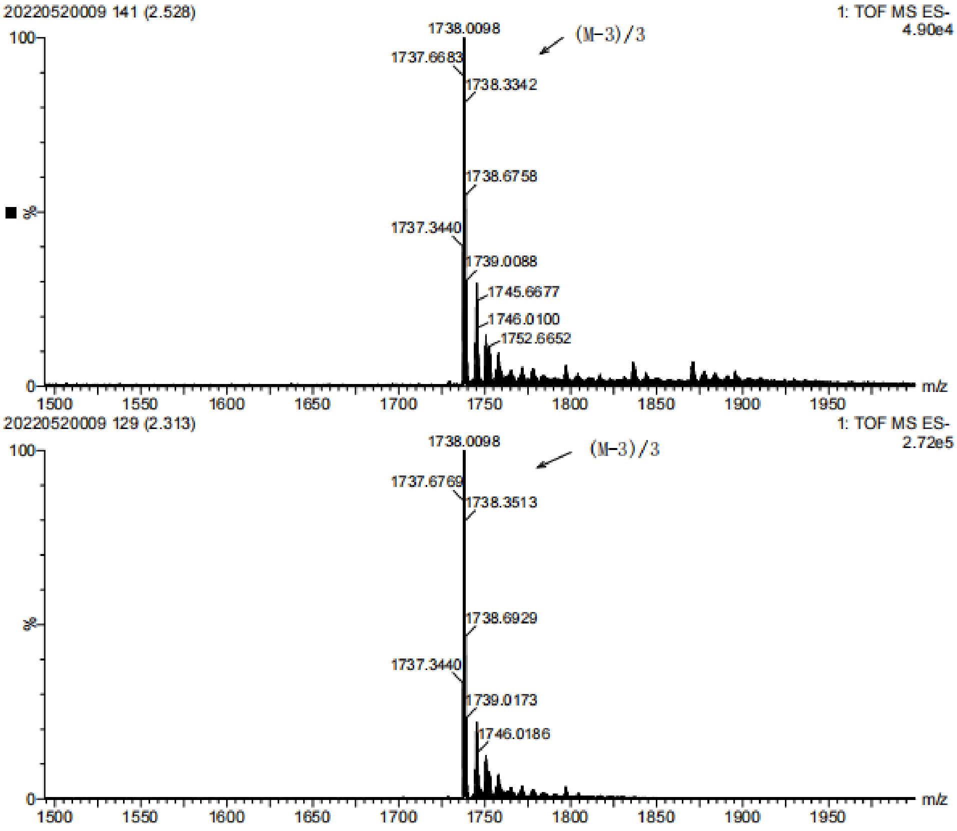

LC Trace and Mass of **K1**

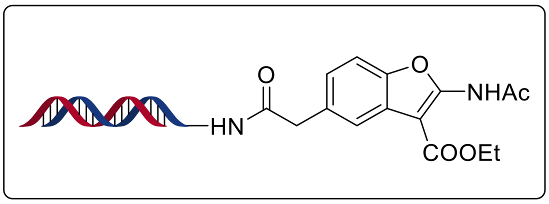

Following General Procedure **K1**

Yield: 33%

Exact mass: 5224.3197

Triply charged mass (M-3)/3, calculated 1740.4399; observed 1740.3434.

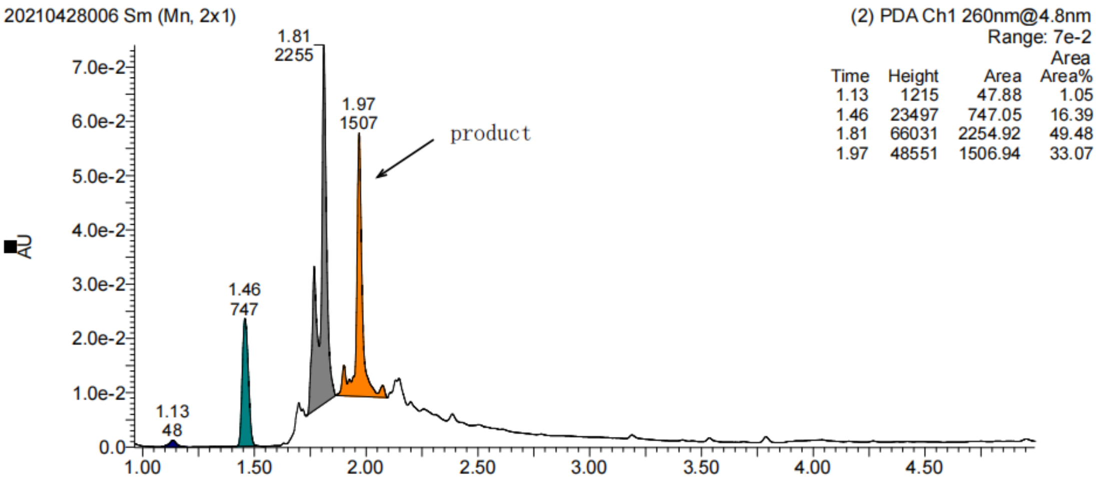

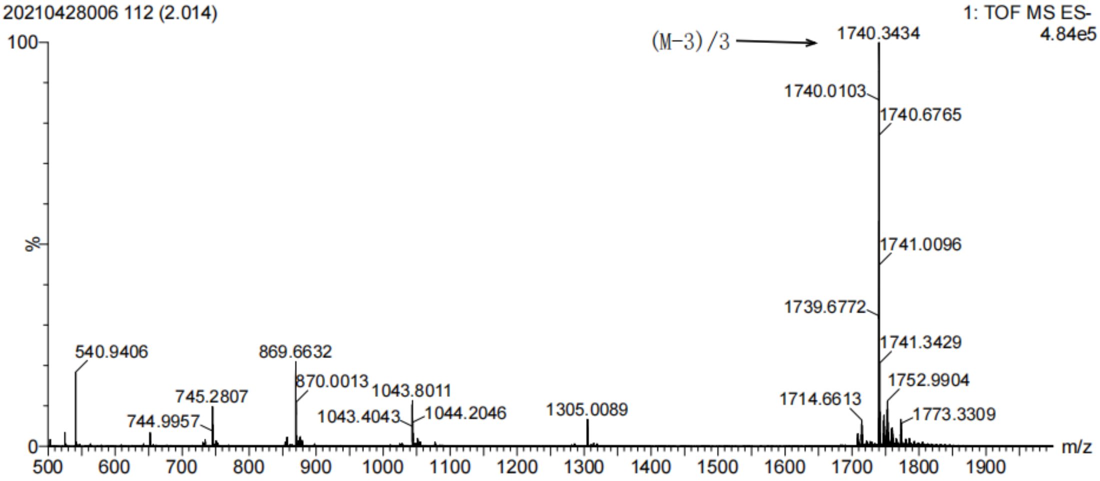

LC Trace and Mass of **L1**

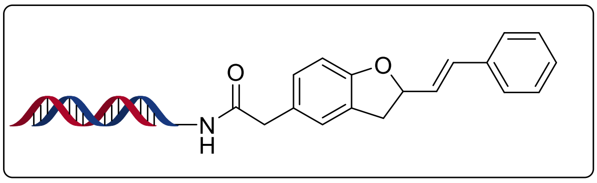

Following General Procedure **L1**
Yield: 60%
Exact mass: 5199.3399
Triply charged mass (M-3)/3, calculated 1732.1133; observed 1732.0062.

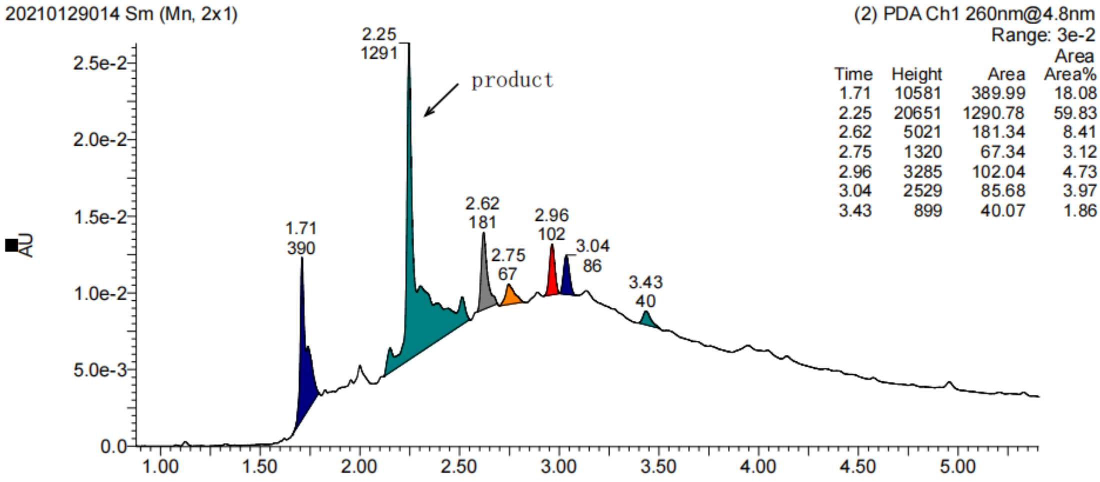

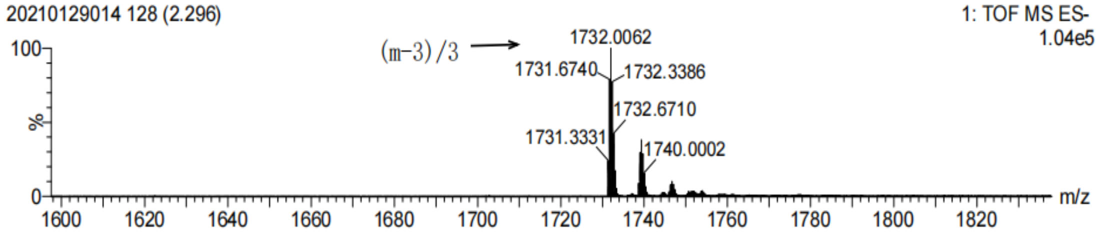

LC Trace and Mass of **E1**

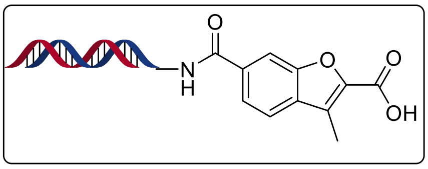

Following General Procedure **E1**

Yield: 100%

Exact mass: 5139.2673

Triply charged mass (M-3)/3, calculated 1712.0891; observed 1711.9597.

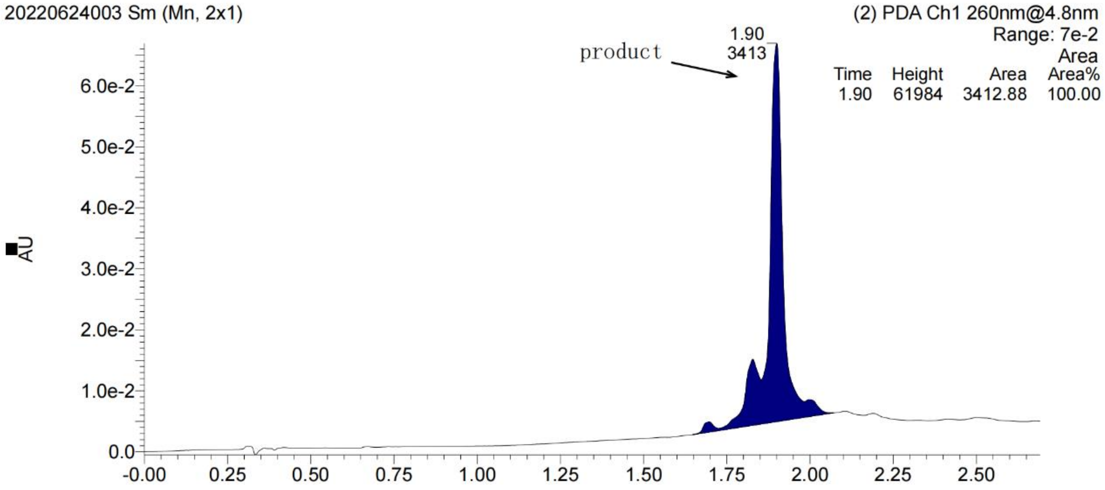

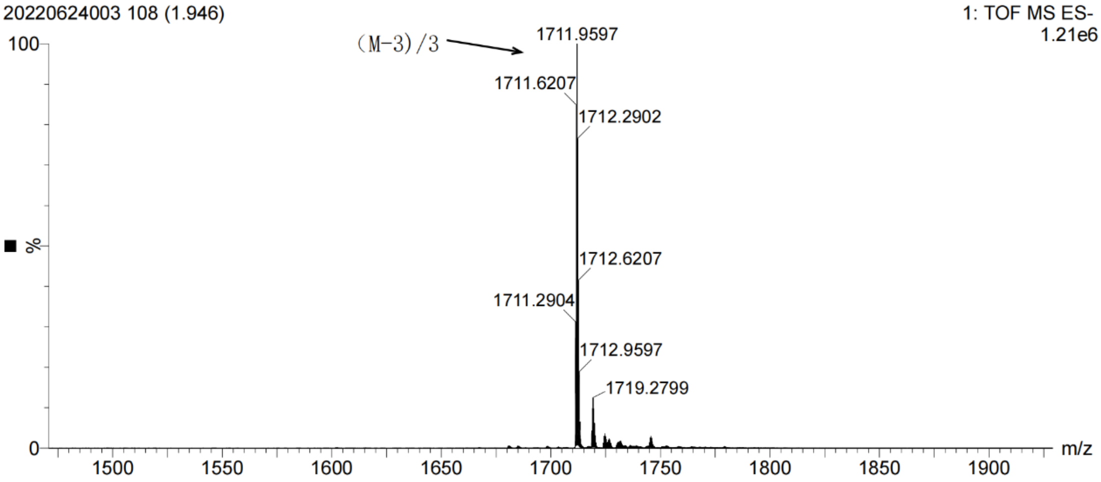

LC Trace and Mass of **F1**

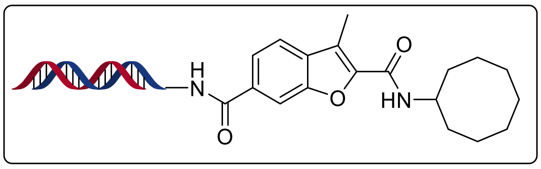

Following General Procedure **F1**
Yield: 72%
Exact mass: 5242.3468
Triply charged mass (M-3)/3, calculated 1746.4410; observed 1746.3302.

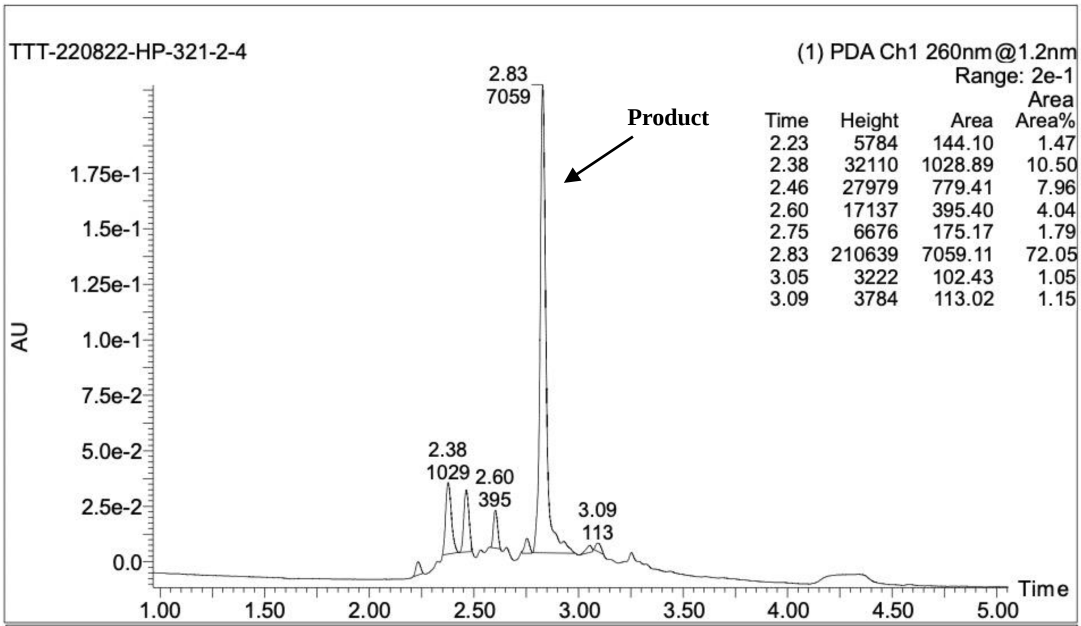

Time	Height	Area	Area Area%
2.23	5784	144.10	1.47
2.38	32110	1028.89	10.50
2.46	27979	779.41	7.96
2.60	17137	395.40	4.04
2.75	6676	175.17	1.79
2.83	210639	7059.11	72.05
3.05	3222	102.43	1.05
3.09	3784	113.02	1.15

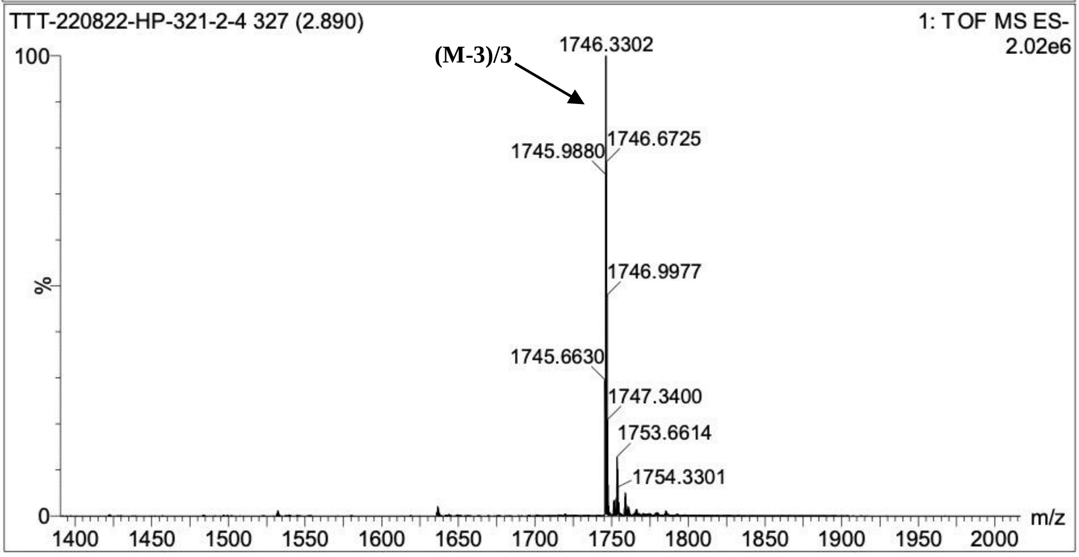

LC Trace and Mass of **F2**

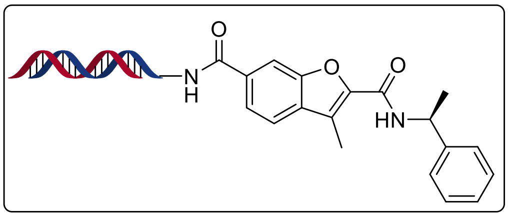

Following General Procedure **F2**

Yield: 74%

Exact mass: 5248.3937

Triply charged mass (M-3)/3, calculated 1748.4566; observed 1748.2129.

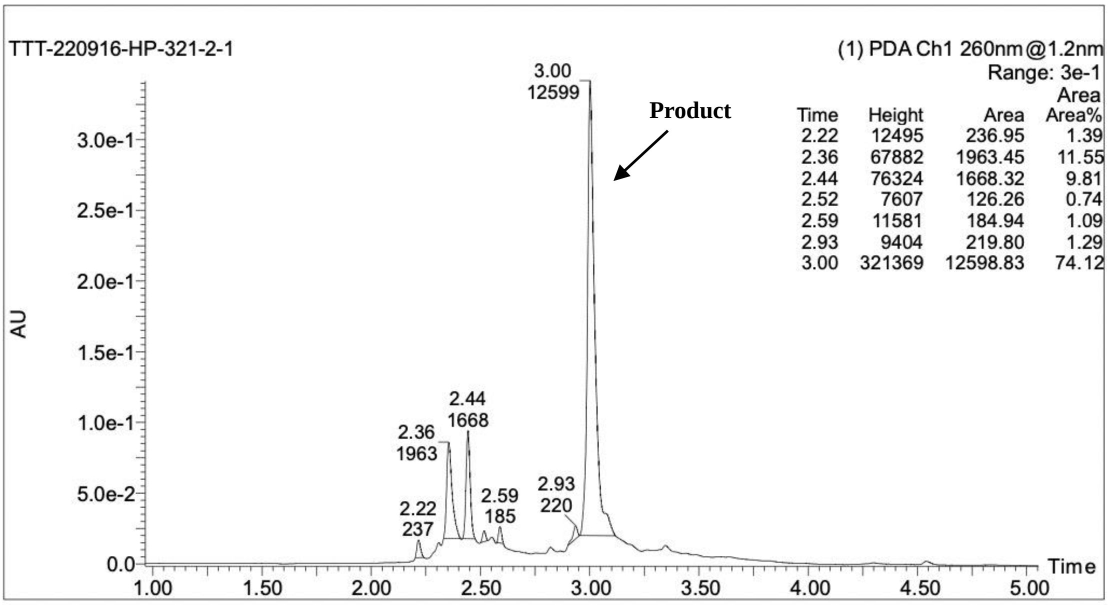

Time	Height	Area	Area Area%
2.22	12495	236.95	1.39
2.36	67882	1963.45	11.55
2.44	76324	1668.32	9.81
2.52	7607	126.26	0.74
2.59	11581	184.94	1.09
2.93	9404	219.80	1.29
3.00	321369	12598.83	74.12

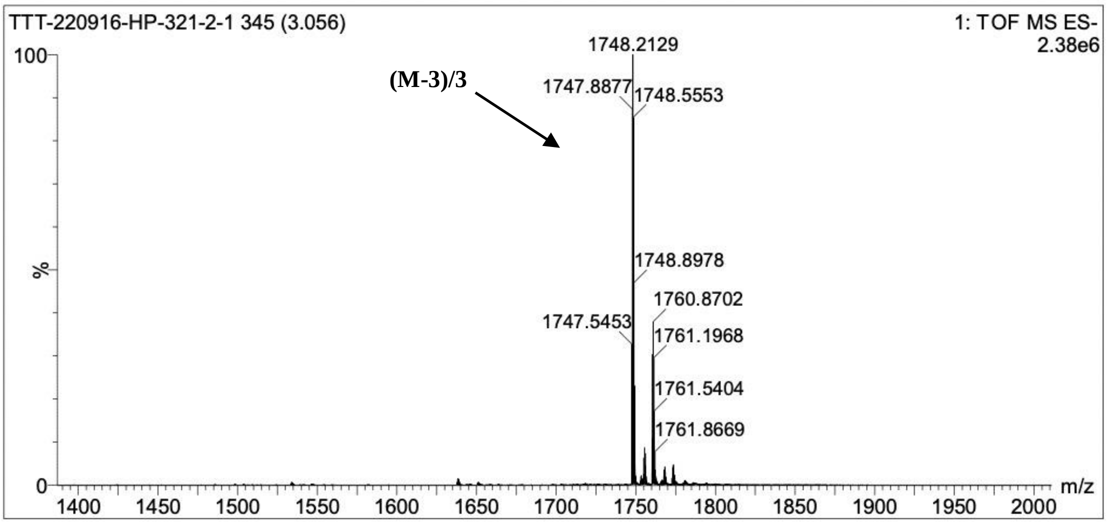

LC Trace and Mass of **F3**

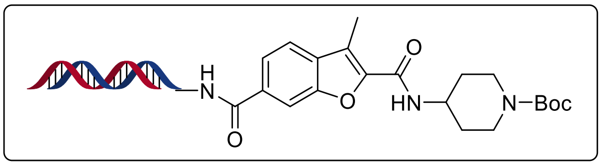

Following General Procedure **F3**

Yield: 73%

Exact mass: 5321.4101

Triply charged mass (M-3)/3, calculated 1772.7954; observed 1772.5388.

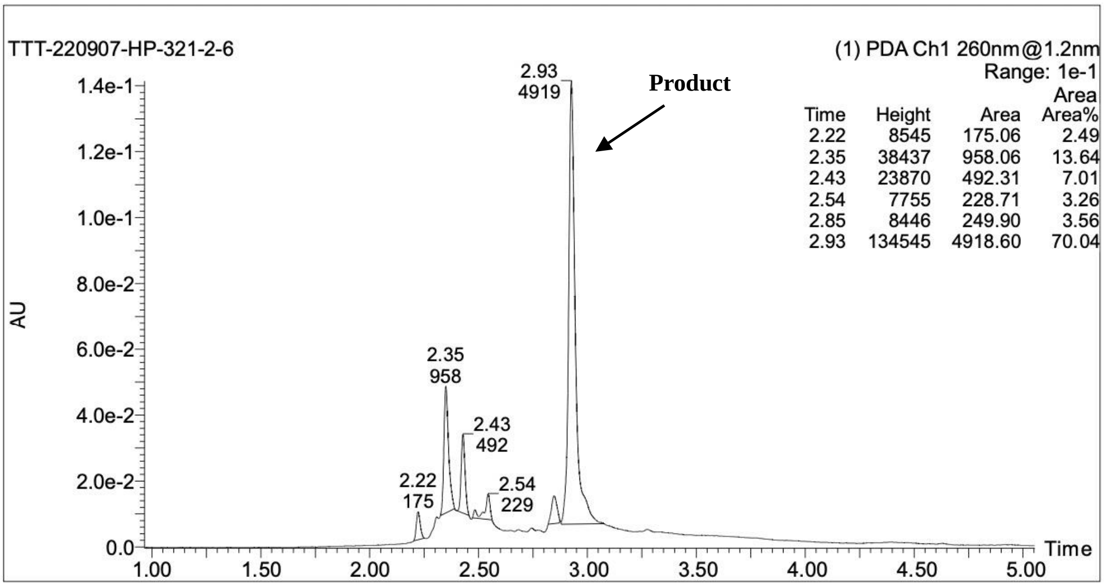

Time	Height	Area	Area%
2.22	8545	175.06	2.49
2.35	38437	958.06	13.64
2.43	23870	492.31	7.01
2.54	7755	228.71	3.26
2.85	8446	249.90	3.56
2.93	134545	4918.60	70.04

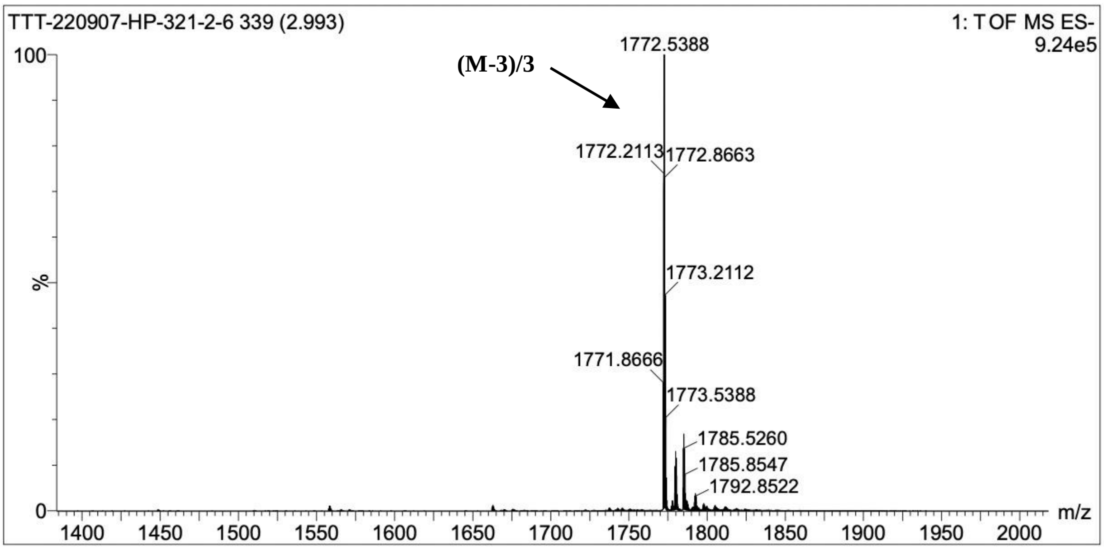

LC Trace and Mass of **F4**

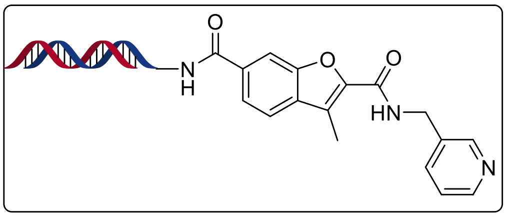

Following General Procedure **F4**

Yield: 64%

Exact mass: 5229.3264

Triply charged mass (M-3)/3, calculated 1742.1009; observed 1741.8159.

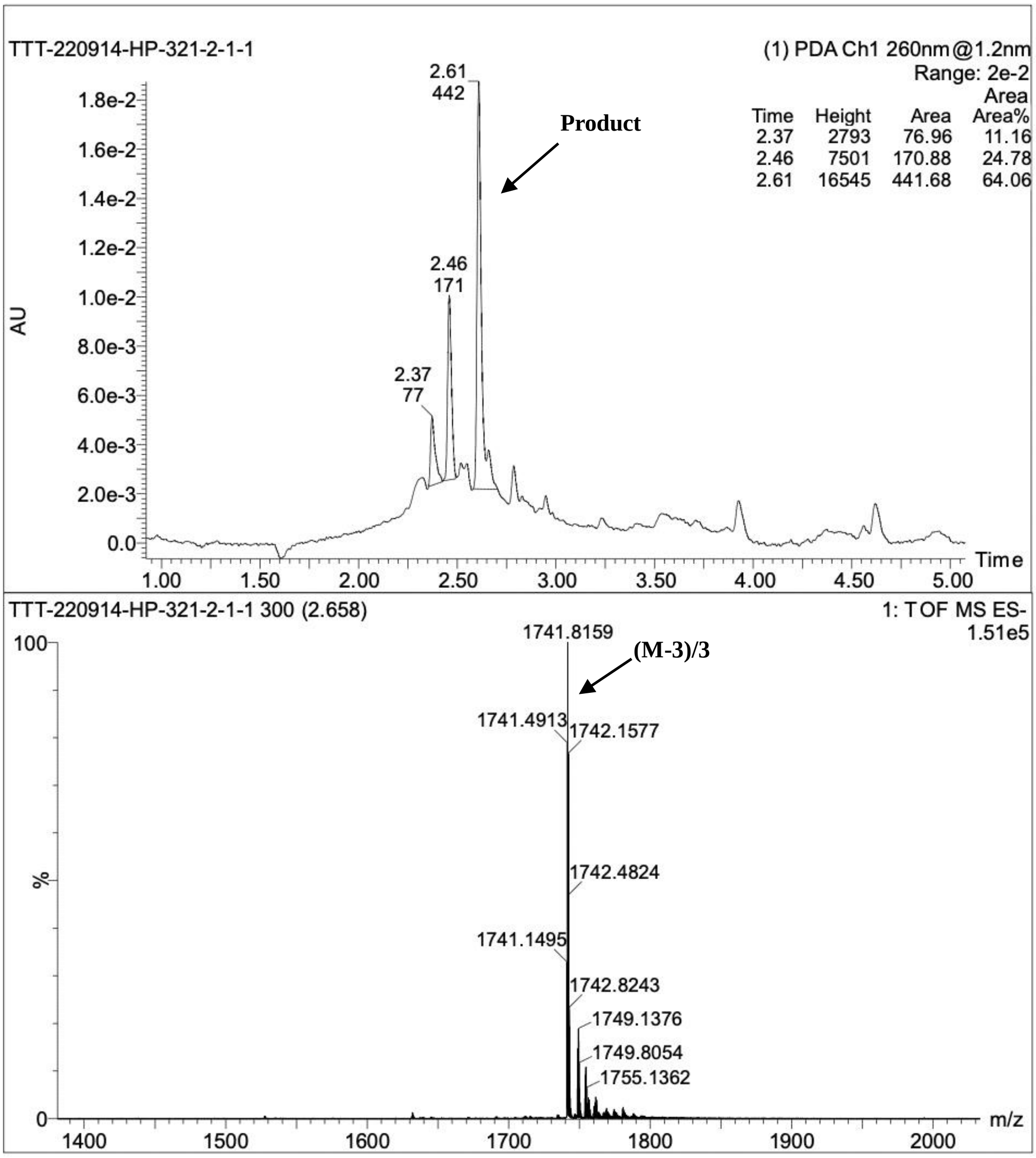

LC Trace and Mass of **M**

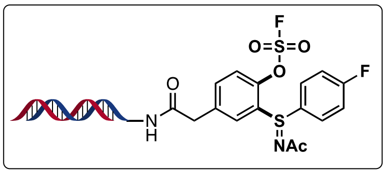

Yield: 72%

Exact mass: 5336.2443

Triply charged mass (M-3)/3, calculated 1777.7481; observed 1777.4034.

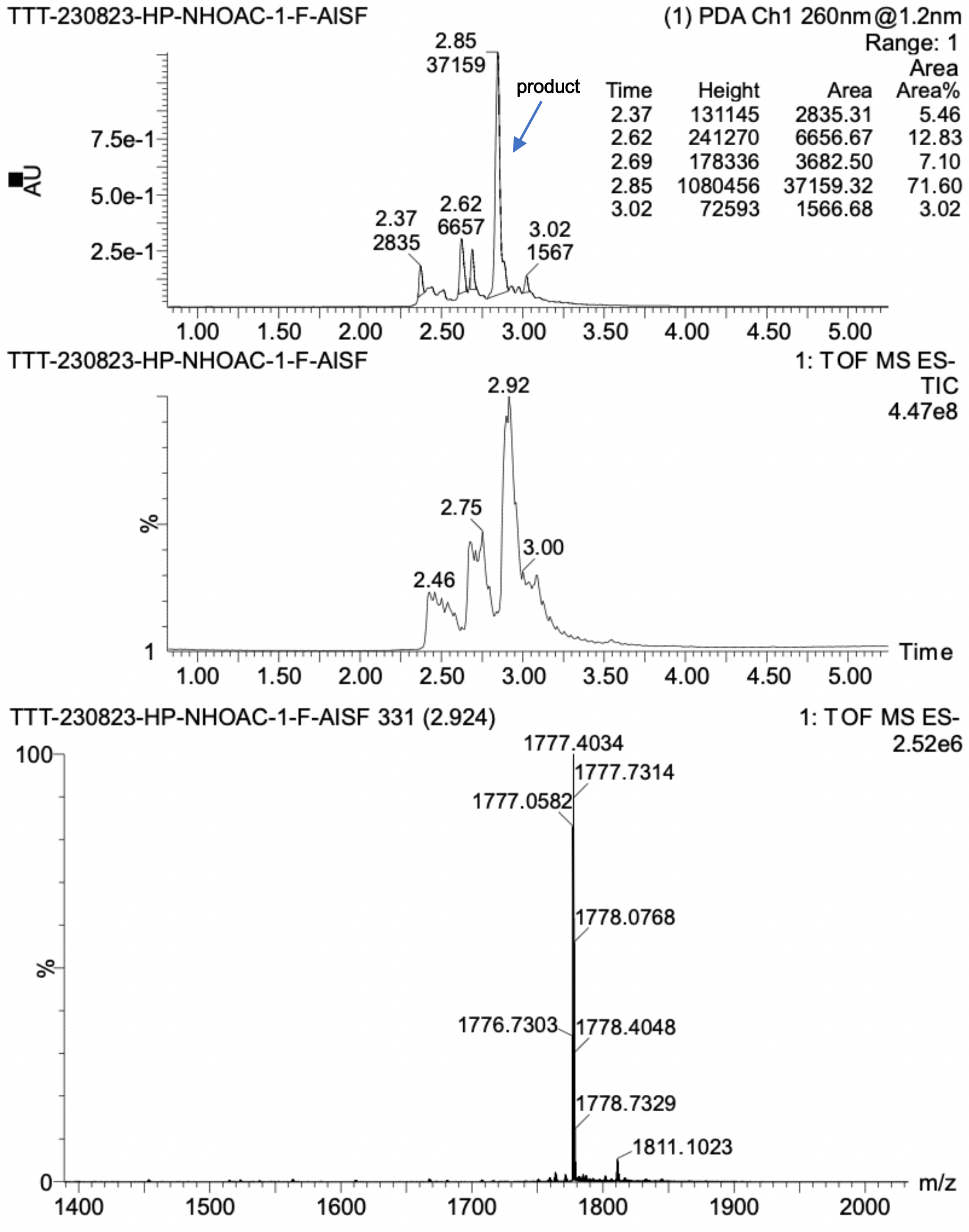

LC Trace and Mass of **N**

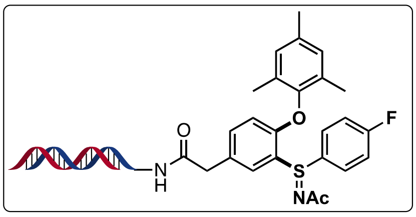

Yield: 77%

Exact mass: 5372.37

Triply charged mass (M-3)/3, calculated 1789.7900; observed 1789.4384.

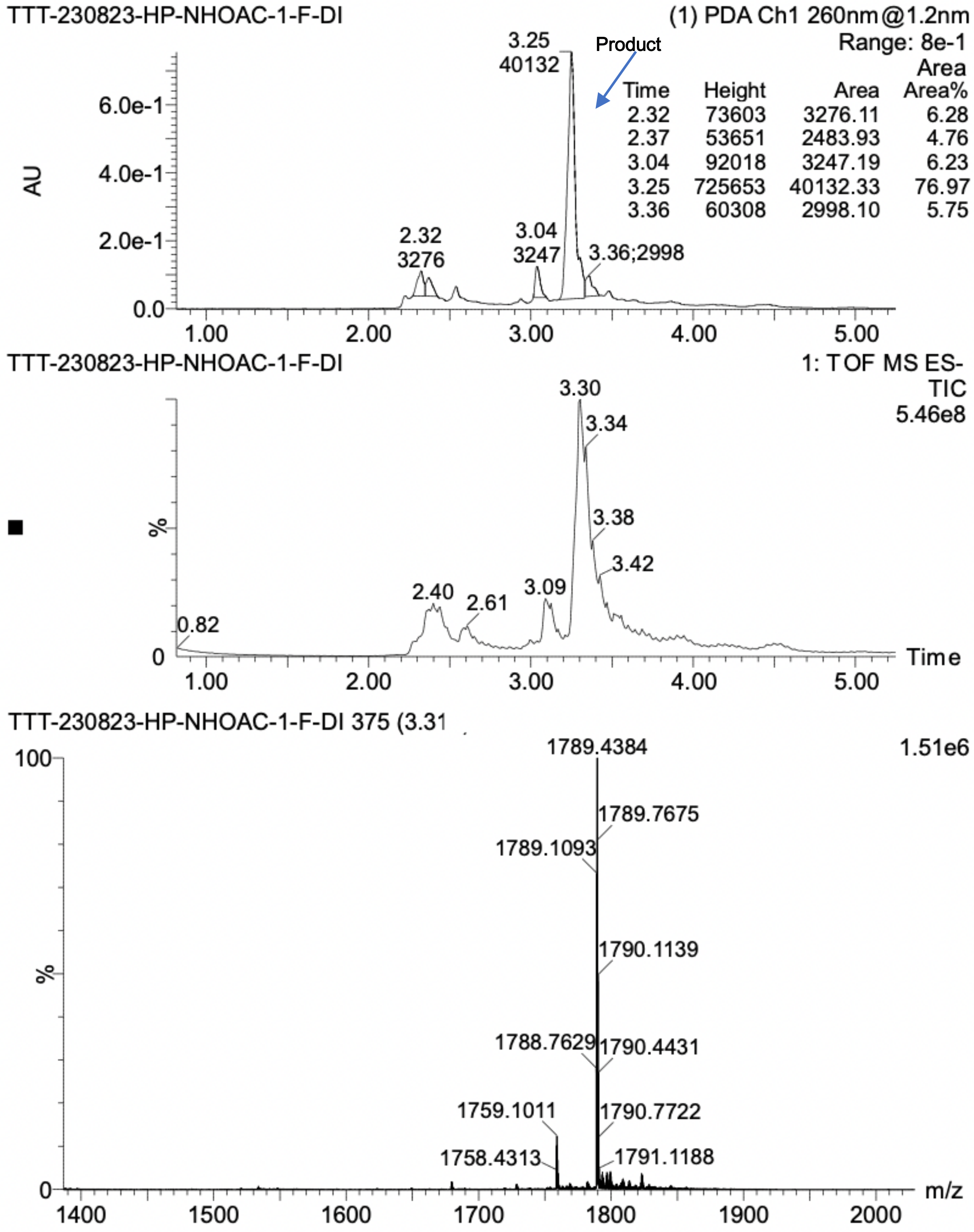

Reference

[S1] (a) Petrassi, H. M.; Sharpless, K. B.; Kelly, J. W. *Org. Lett.*, **2001**, *3*, 139. (b) Takeda, N.; Miyata, O.; Naito, T. *Eur. J. Org. Chem.*, **2007**, 1491. (c) Tang, D.; Gai, Y.; Polemeropoulos, A.; Chen, Z.; Wang, Z. *Bioorg. Med. Chem. Lett.*, **2008**, *18*, 5078. (d) Liu, G.; Shen, Y.; Zhou, Z.; Lu, X. *Angew. Chem. Int. Ed.*, **2013**, *52*, 6033. (e) Li, B.; Lan, J.; Wu, D.; You, J. *Angew. Chem. Int. Ed.*, **2015**, *54*, 14008.

[S2] Kuram, M. R.; Bhanuchandra, M.; Sahoo, A. K. *Angew. Chem. Int. Ed.*, **2013**, *52*, 4607.
